# Estimation of Prenatal Alcohol Exposure: Comparison of Retrospective Survey and Measurement of Fatty Acid Ethyl Esters, Ethyl Sulfate, and Ethyl Glucuronide Concentrations in Neonatal Meconium

**DOI:** 10.3390/toxics14020155

**Published:** 2026-02-04

**Authors:** Marek Wiergowski, Iwona Jańczewska, Jolanta Wierzba, Monika Cichoń-Kotek, Mateusz Kacper Woźniak, Agata Kot-Wasik, Marek Biziuk, Jacek Sein Anand, Daria Barbara Schetz, Małgorzata Glińska, Katarzyna Hinca

**Affiliations:** 1 Department of Forensic Medicine, Faculty of Medicine, Medical University of Gdańsk, Dębowa 23 St., 80-204 Gdańsk, Poland; mateusz.wozniak@gumed.edu.pl; 2 Division of Neonatology, Department of Gynecology, Obstetrics and Neonatology, Faculty of Medicine, 17 Mariana Smoluchowskiego St., 80-214 Gdańsk, Poland; iwona.janczewska@gumed.edu.pl (I.J.); mglinska@uck.gda.pl (M.G.); katarzyna.hinca@gumed.edu.pl (K.H.); 3 Department of Obstetric and Gynaecological Nursing, Institute of Nursing and Midwifery, Medical University of Gdańsk, Dębinki 7 St., 80-214 Gdańsk, Poland; jolanta.wierzba@gumed.edu.pl (J.W.); monika.cichon-kotek@gumed.edu.pl (M.C.-K.); 4 Department of Analytical Chemistry, Faculty of Chemistry, Gdańsk University of Technology, 11/12 Narutowicza St., 80-233 Gdańsk, Poland; agawasik@pg.edu.pl (A.K.-W.); marek.biziuk@pg.edu.pl (M.B.); 5 Division of Clinical Toxicology, Faculty of Health Sciences with the Institute of Maritime and Tropical Medicine, Medical University of Gdansk, 80-211 Gdańsk, Poland; jacek.anand@gumed.edu.pl; 6 Pomeranian Centre of Toxicology, Kartuska 4/6 St., 80-104 Gdańsk, Poland; 7 Department of Pharmacology, Faculty of Medicine, Medical University of Gdańsk, Dębowa 23 St., 80-204 Gdańsk, Poland; dariaschetz@gumed.edu.pl

**Keywords:** alcohol consumption biomarkers, ethyl glucuronide, ethyl sulfate, fatty acid ethyl esters, meconium, fetal alcohol spectrum disorders, prenatal alcohol exposure, liquid chromatography–tandem mass spectrometry, gas chromatography–mass spectrometry

## Abstract

Determining the concentration of fatty acid ethyl esters (FAEEs), ethyl sulfate (EtS), and ethyl glucuronide (EtG) is crucial for establishing the true scale of prenatal alcohol exposure (PAE) and enabling early diagnosis of fetal alcohol spectrum disorders. This study primarily aimed to compare two detection methods: retrospective maternal alcohol consumption surveys and chromatographic analysis of newborn meconium. Among 478 mothers, parallel survey data and meconium samples were collected. Nine FAEEs were measured by gas chromatography–mass spectrometry, and EtG and EtS by liquid chromatography–tandem mass spectrometry. The study also aimed to establish marker cut-offs and evaluate their clinical utility. While only 4% (approximately) of mothers reported alcohol consumption during pregnancy, the biomarker analysis suggested a significant underestimation of the actual PAE scale, highlighting the limitations of self-reported data. Analysis using the cumulative biomarker index for two biomarkers with a threshold of ≥5 indicated that alcohol consumption affected approximately 3% of the studied population, further demonstrating the low reliability of maternal self-reports. Ultimately, this study confirms that the combined EtG and EtS measurements provide the most reliable diagnostic information for PAE and underscores the necessity of objective meconium screening in clinical practice.

## 1. Introduction

A primary challenge in diagnosing fetal alcohol spectrum disorders (FASD) at an early stage is the accurate identification and verification of prenatal alcohol exposure (PAE). Consequently, clinicians must rely on a comprehensive review of the child’s medical history and clinical observations to reach a diagnosis. Prospective studies by Australian pediatricians indicate a median diagnostic age of 3.3 years for children with FASD, with only 6.5% diagnosed at birth and 63% by age 5 [1]. In the US, data from 2011 to 2013 showed that approximately 10% of pregnant women reported alcohol consumption in the last 30 days and that approximately 1–5% of school-age children met the criteria for FASD. In Poland, FASD is rarely diagnosed, yet research indicates a significant prevalence; a study of school children aged 7–9 years estimated a prevalence of at least 2%. Some studies suggest that approximately 10% of Polish women consume alcohol during pregnancy, in line with the global average and relatively low compared to other European countries [2,3].

It is difficult to obtain reliable information about alcohol consumption by women during pregnancy. Many women conceal this fact for fear of stigmatization. For this reason, it is necessary to find objective criteria unrelated to medical history that would indicate PAE (e.g., in the form of PAE biomarkers) [4]. Diagnostic tests related to alcohol consumption can be considered depending on the detection window for ethanol and its oxidative and non-oxidative metabolites. PAE biomarkers are typically ethyl glucuronide (EtG), ethyl sulfate (EtS), and fatty acid ethyl esters (FAEEs), which are measured in biological materials such as a newborn’s meconium, hair, nails, or umbilical cord. In recent years, it has been increasingly suggested that the most reliable information on PAE is provided by combining several selected biomarkers such as EtG, EtS, and FAEEs. Bakhireva et al. [5,6] found that the most common PAE biomarkers were EtS (7.8%) and ethyl oleate (EE 18:1, 6.9%). They also found that 5.4% of meconium samples tested positive for at least two biomarkers. Table 1 presents the results of meconium analyses from previous studies in which PAE biomarkers were determined to estimate the cut-off values differentiating women who did and did not consume alcoholic beverages during pregnancy.

According to Bakhireva et al. [5], an ideal PAE biomarker should have the following characteristics: (1) the ability to detect low-to-moderate alcohol consumption over an extended period following the last episode of alcohol consumption; (2) a high probability of detecting alcohol consumption during pregnancy (i.e., high sensitivity and detectability); and (3) a low false-positive rate (i.e., high specificity). In addition, the authors indicated that the ideal PAE method is one in which: (1) the biological sample is easily accessible through a minimally invasive and clinically acceptable procedure, (2) requires little or no sample preparation for testing, (3) the analytical procedure is simple and relatively inexpensive, and (4) provides rapid results, preferably at the point of care. In practice, however, most current PAE biomarkers meet only one or two of these attributes.

This study aimed to compare two PAE detection methods during the prenatal period through: (1) retrospective questionnaire surveys concerning, among other things, alcohol consumption by women before and during prenatal periods, and (2) chromatographic analysis of newborn meconium and measurement of the concentration of nine FAEEs, EtG, and EtS. In addition, the study aimed to determine the cut-off concentration and usefulness of PAE markers in meconium that would indicate FAE.

## 2. Materials and Methods

### 2.1. Retrospective Survey

Meconium samples were collected, and cross-sectional surveys were conducted among 495 mothers who gave birth between 16 June 2019 and 24 April 2020, at the Neonatology Clinic (NC) of the Medical University of Gdańsk (MUG, Poland) University Clinical Center (UCC). As a Level III reference center, the NC collaborates closely with the Maternity Clinic (MC) of MUG and UCC to provide comprehensive neonatal care for both healthy and high-risk newborns.

The survey (*n* = 495) utilized an original questionnaire (Table S1) for maternal self-assessment of health status, pre-existing and gestational disorders, and the consumption of medications, stimulants, and dietary supplements during pregnancy. To ensure anonymity, each questionnaire and corresponding meconium sample were assigned a unique identification number. All participating mothers provided written informed consent. We analyzed the sociodemographic and economic characteristics, behavioral habits, obstetric history, chronic diseases, and pregnancy complications. The study was limited to singleton pregnancies.

### 2.2. Sampling and Determination of PAE Biomarkers

Meconium samples (*n* = 495) were collected at the NC UCC MUG within 24 h of birth. Samples were collected from diapers using plastic spatulas, transferred to plastic vials (Falcons), and stored at −20 °C to preserve analyte integrity. Analyte enrichment and isolation (FAEEs, EtG, and EtS) were conducted at the Department of Analytical Chemistry (DAC) of Gdańsk University of Technology (GUT, Poland). Subsequent instrumental analyses were performed using gas chromatography–mass spectrometry (GC-MS) at the MUG Department of Forensic Medicine and liquid chromatography–tandem mass spectrometry (LC-MS/MS) at the DAC GUT. Meconium from mothers who reported no exposure to alcohol during pregnancy served as blank samples for validation; chromatographic analysis confirmed these blanks were free of analytes or contained concentrations below the limits of detection (LOD). While certain findings from this survey have been previously published [12], they addressed different research objectives—particularly whether sociodemographic, economic, and lifestyle factors, professional status, or chronic diseases contributed to preterm birth within this Pomeranian cohort.

The diagnostic procedure, including validation of analytical parameters, for determining the concentrations of nine FAEEs, EtS, and EtG in meconium has been described in detail in an earlier publication [13]; key elements of this procedure are summarized below. The nine FAEEs determined were ethyl laurate (EE 12:0), ethyl myristate (EE 14:0), ethyl palmitate (EE 16:0), ethyl stearate (EE 18:0), ethyl oleate (EE 18:1), ethyl linoleate (EE 18:2), ethyl linolenate (EE 18:3), ethyl arachidate (EE 20:0), and ethyl arachidonate (EE 20:4). Given the highly diverse chemical properties of these analytes, two distinct analytical workflows were employed following preliminary extraction: GC-MS for the non-polar, lipophilic FAEEs and LC-MS/MS for the polar, hydrophilic EtS and EtG.

The meconium samples were thawed for 1 h at room temperature and homogenized. Then, 200 ± 5 mg of meconium was weighed, spiked with deuterated internal standards, and stirred. Liquid–liquid extraction was performed using methanol and hexane; the resulting upper hexane layer was transferred to a solid-phase extraction (SPE) column, where FAEEs were eluted with dichloromethane. Following elution from the SPE column, the extract was evaporated under a gentle stream of nitrogen. To prevent the volatilization of FAEEs observed at 35 °C, the nitrogen flow was terminated immediately upon solvent removal. Finally, the dry residue was dissolved in 100 μL of hexane and analyzed via GC-MS (FAEEs).

Acetonitrile and methanol were added to the remaining meconium fraction, and EtG and EtS were isolated using aminopropyl-modified silica weak anion-exchange SPE columns (NH_2_). Following evaporation, the dry residues were dissolved in 100 μL of mobile phase solution and transferred to autosampler vials for LC-MS/MS analysis (EtG and EtS). The LOD values for FAEEs determination by GC-MS ranged from 0.8 to 7.5 ng/g, while the LOD for EtS and EtG by LC-MS/MS were 0.2 ng/g and 0.8 ng/g, respectively. The limit of quantification (LOQ) values for FAEEs ranged from 5 to 25 ng/g, and for EtS and EtG, they were 1 ng/g and 2.5 ng/g, respectively. Precision values ranged from 3.5% to 9.7%, and recovery values ranged from 89.1% to 109%.

### 2.3. Statistical Analysis

Basic statistical analysis of the survey results and PAE biomarkers, including the calculation of means, medians, and standard deviations, alongside the generation of two-parameter and box plots, was performed using Microsoft Excel 365. More complex analyses, including multivariate correlation estimations, were conducted in R (R Core Team) [14] using the RStudio environment (Posit, version 2022.02.3+492.pro3) [15] and the tidyverse suite of packages [16]. Due to the high skewness of the variables, exploratory factor analysis (EFA) used Spearman’s correlation coefficient for calculations. In the first step of cluster analysis, the number of potential clusters was examined and the Euclidean metric was used. Analysis based on the construction of the dependent variable and logical regression was performed using Fisher’s exact test. All tests were performed assuming a statistical significance level of 0.05.

## 3. Results

The results of all surveys conducted among pregnant women at the NC of the MUG are summarized in Tables S2–S8. The results of all GC-MS-SIM and LC-ESI-MS/MS chemical analyses of infant meconium, collected on the first day after birth at the NC of the MUG, are summarized in Tables S9 (mass concentration units, µg/g) and S10 (molar concentration units, nmol/g). To compare the results of the retrospective surveys with the PAE biomarker concentrations in meconium, the total number of compared results was reduced from 495 to 478 due to incomplete survey responses and insufficient biological material. The surveys were conducted among pregnant women aged 14–45 (median age 31, mean age 31) years.

### 3.1. Results of Retrospective Survey

Table S11 summarizes the survey results, indicating the number and percentage share of individual responses. To mitigate bias, questions regarding alcohol consumption were blended with general social queries to minimize participant focus on that specific topic.

**Figure 1:**
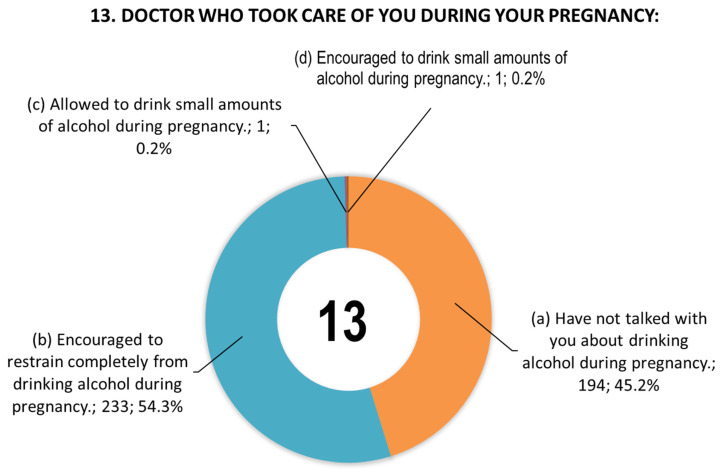
Responses (*n* = 429) to question 13 regarding information provided by doctors during pregnancy. Due to rounding of data to the decimal places, the sum of values may not be exactly 100%.

**Figure 2:**
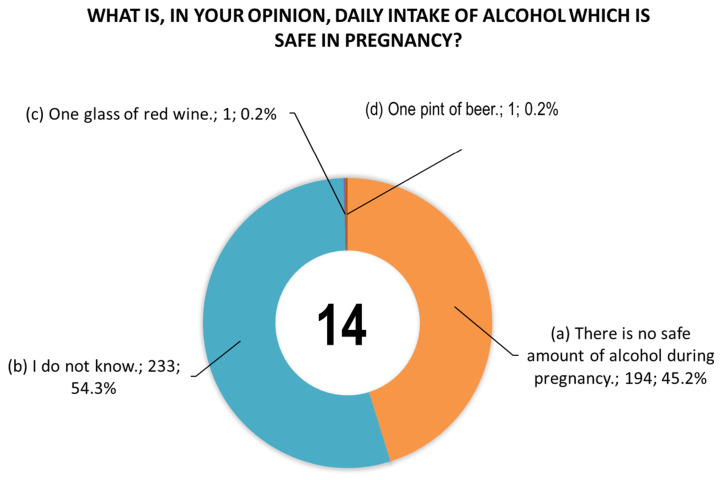
Responses (*n* = 432) to question 14 regarding safe daily alcohol consumption during pregnancy. Due to rounding of data to the decimal places, the sum of values may not be exactly 100%.

Figure 1 shows women’s responses to question 13 concerning information provided by doctors during pregnancy. Figure 2 shows women’s responses to question 14 concerning the daily dose of alcohol that they consider safe for pregnant women.

**Figure 3:**
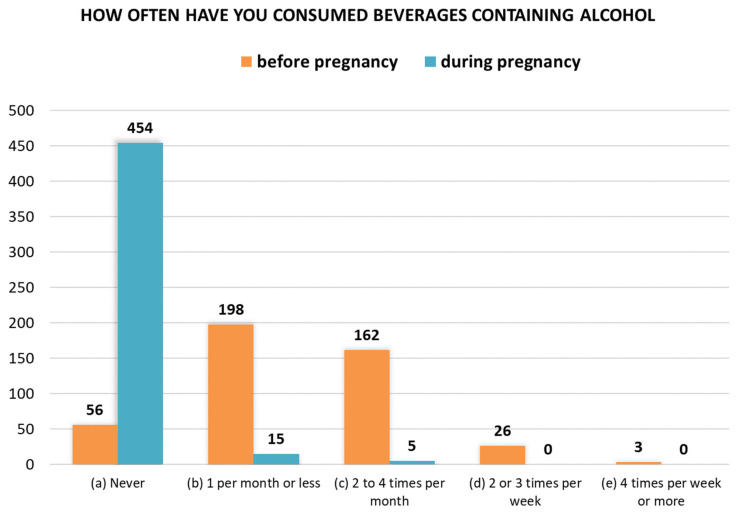
Responses to questions 20 and 21 regarding alcohol consumption frequency before (*n* = 445) and during pregnancy (*n* = 474).

A comparison of women’s responses to questions 20 and 21, regarding the frequency of alcohol consumption before and during pregnancy, is shown in Figure 3. Fifteen women reported consuming alcohol once a month or less during pregnancy, while five women admitted to consuming alcohol 2–4 times a month. In total, 20 women (4.2%) consumed alcohol during pregnancy. Before becoming pregnant, 389 women reported consuming alcohol with varying frequency (87.4%).

**Figure 4:**
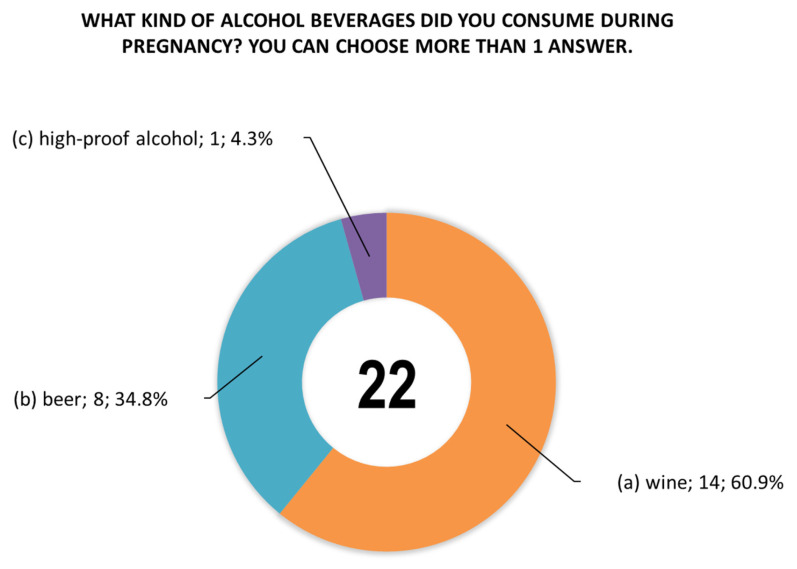
Responses (*n* = 19) to question 22 regarding the type of alcoholic beverage consumed during pregnancy. Woman no. 54 reported consuming all three alcoholic beverages (answers a, b, and c), and women no. 186 and 237 reported consuming beer and wine (answers a and b).

**Figure 5:**
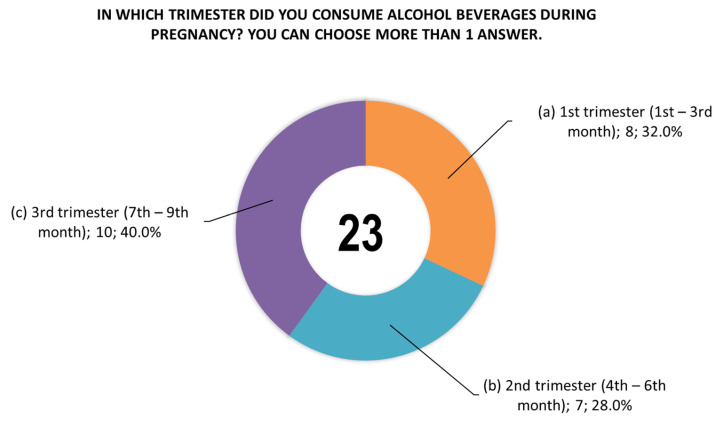
Responses (*n* = 19) to question 23 regarding the time (trimester) of alcohol consumption during pregnancy. Women no. 42 and 363 reported consuming alcohol in all trimesters (answers a, b, and c), and women no. 4 and 244 reported consuming alcohol in the second and third trimesters (answers b and c).

**Figure 6:**
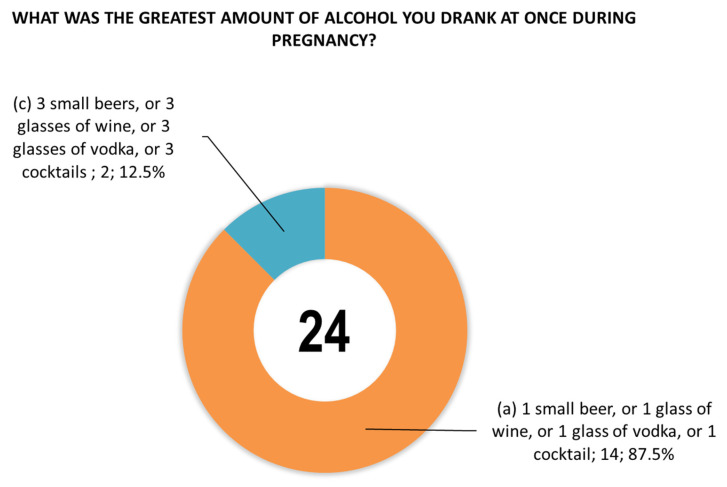
Positive responses to question 24 regarding maximum single-session alcohol consumption during pregnancy. No respondents selected option b (2 small beers, or 2 glasses of wine, or 2 glasses of vodka, or 2 cocktails).

The women were also asked questions about the type of alcoholic beverage consumed during pregnancy (Figure 4), the time (trimester) of alcohol consumption during this period (Figure 5), and the largest amount of alcohol they drank on a single occasion during pregnancy (Figure 6).

**Figure 7:**
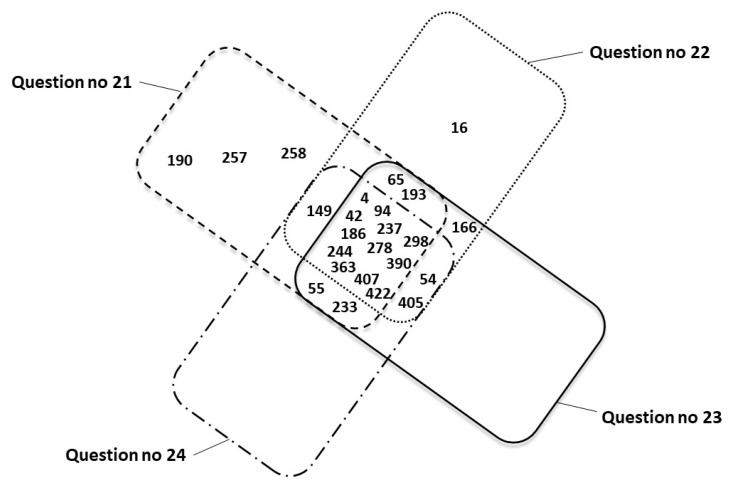
Summary of positive PAE responses (represented by personal identification numbers) across questionnaire items 21 (*n* = 20), 22 (*n* = 19), 23 (*n* = 19), and 24 (*n* = 16). Positive answers for question no 21—dashed line box, question no 22—dotted one, question no 23—solid one and question no 24—dashed-dotted one.

Figure 7 shows a summary of mothers’ responses (represented by personal identification numbers) confirming PAE in four different questionnaire items: 21 (*n* = 21), 22 (*n* = 19), 23 (*n* = 19), and 24 (*n* = 16). Respondents 1 and 360, for whom the questionnaire was incomplete and PAE resulted from the circumstances of childbirth, are excluded.

### 3.2. Results of PAE Biomarker Determination

Chemical test results (Tables S9 and S10) were used to compile a statistical summary of FAEEs, EtS, and EtG biomarkers in children’s meconium (Table 2). In addition, PAE marker concentrations (C_max_, C_min_, SD, C_average_, and C_median_) were compiled for results exceeding the LOQ, assuming LOQ = 3 × LOD (LOD values determined by Woźniak et al. [13]).

**Figure 8:**
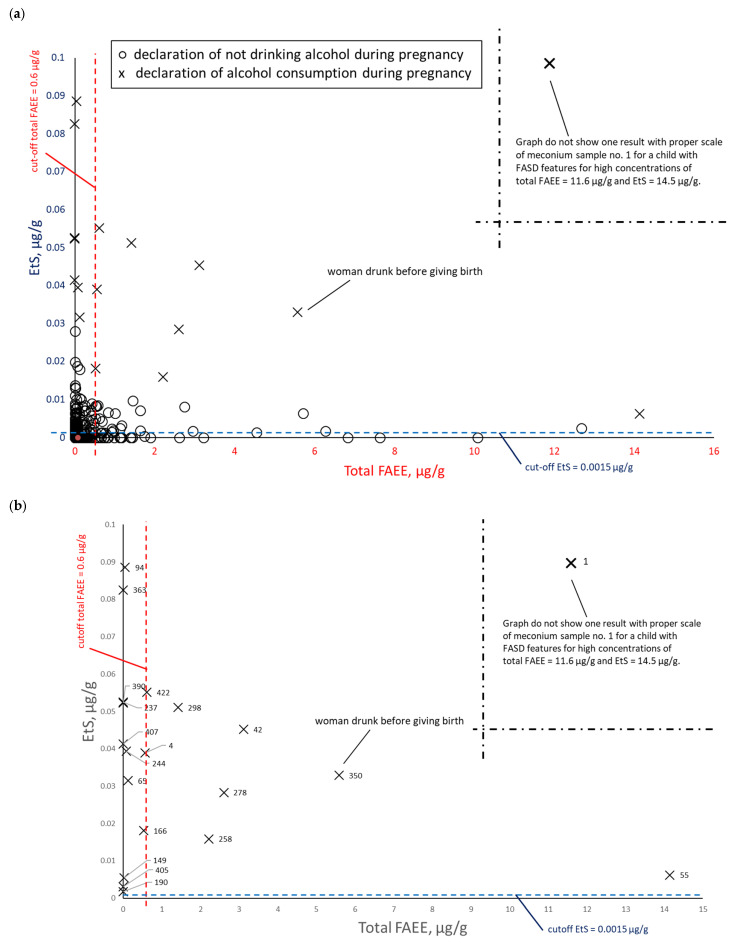
EtS concentrations relative to total FAEEs. (**a**) Comparison between self-reported alcohol consumption (at least one positive response to questions 21, 22, or 23) (×) and self-reported abstinence (○) during pregnancy. (**b**) Subset analysis of the PAE group; self-reported abstinence is excluded to highlight specific sample numbers.

**Figure 9:**
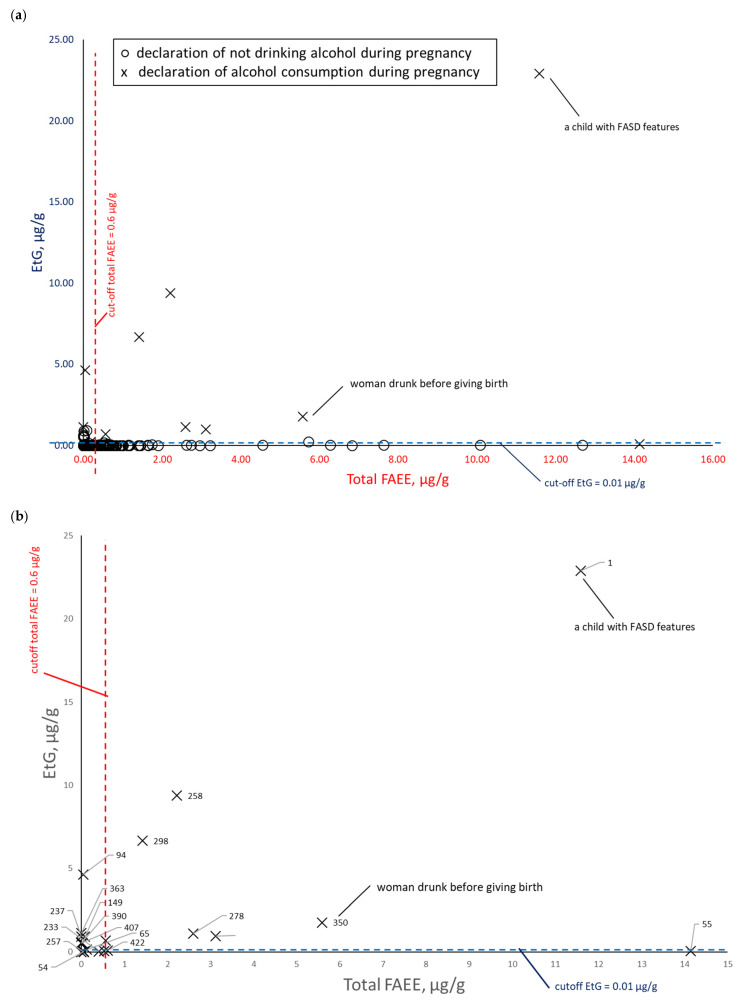
EtG concentrations relative to total FAEEs. (**a**) Comparison between self-reported alcohol consumption (at least one positive response to questions 21, 22, or 23) (×) and self-reported abstinence (○) during pregnancy. (**b**) Subset analysis of the PAE group; self-reported abstinence is excluded to highlight specific sample numbers.

**Figure 10:**
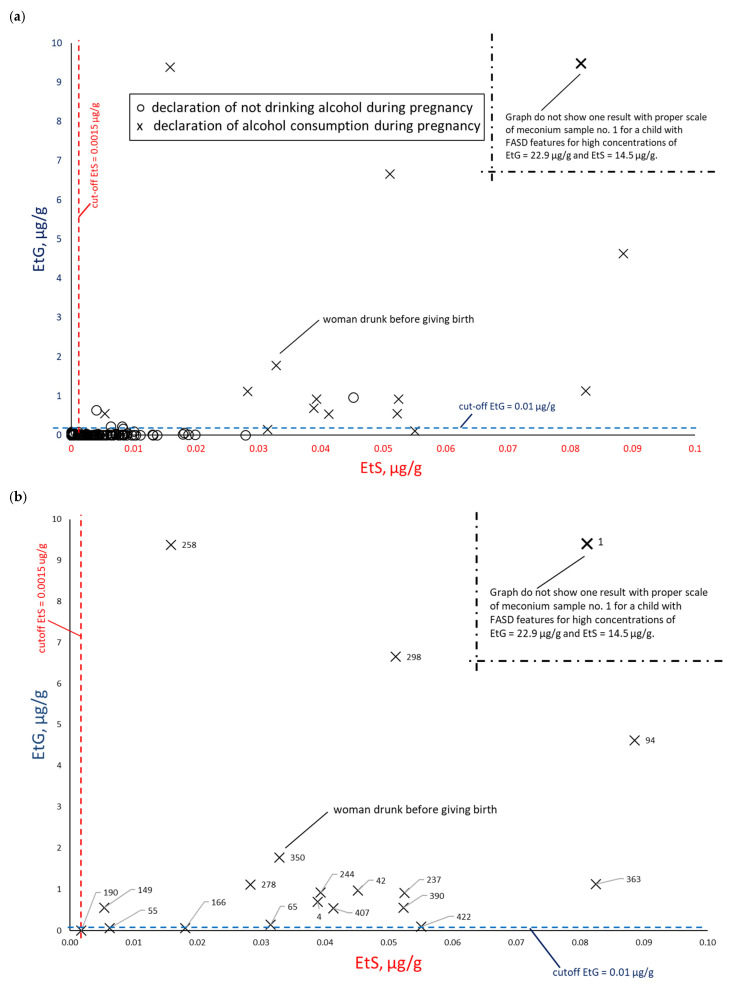
EtS relative to EtG concentrations. (**a**) Comparison between self-reported alcohol consumption (at least one positive answer to questions 21, 22, or 23) (×) and self-reported abstinence (○) during pregnancy. (**b**) Subset analysis of the PAE group; self-reported abstinence is excluded to highlight specific sample numbers.

To assess the correlation between PAE biomarker concentrations in meconium, data were paired and plotted: EtS versus total FAEEs (Figure 8), EtG versus total FAEEs (Figure 9), and EtS versus EtG (Figure 10), incorporating maternal declarations of alcohol consumption during pregnancy. Illustrative cut-off lines were added to each graph, which were initially intended to differentiate between alcohol consumption and abstinence in women during pregnancy:

- Cut-off for total FAEEs of 2 nmol/g [9], which corresponds to approximately 0.6 µg/g (it is assumed that 1 nmol of averaged mass of FAEEs is calculated as 0.3 µg)
- Cut-off for EtS of 0.012 nmol/g, which corresponds to approximately 0.0015 µg/g [7]
- Cut-off for EtG of 0.01 µg/g [6].

### 3.3. Comparison of Retrospective Survey and PAE Biomarkers Results

The maternal survey responses (questions 21–24, Figure 7) are cross-referenced in Table 3 with the corresponding PAE biomarker concentrations and laboratory results.

**Figure 11:**
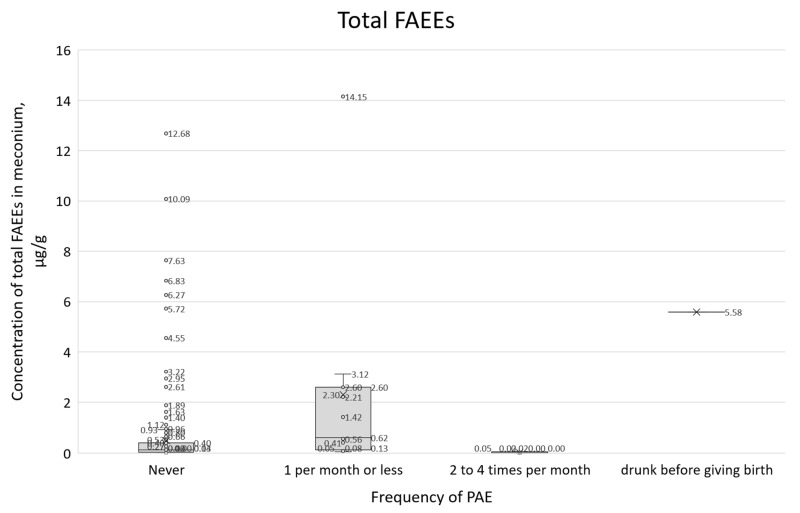
Box plot showing total FAEE concentrations categorized by self-reported consumption frequency (question 21) and cases involving acute maternal intoxication at parturition.

**Figure 12:**
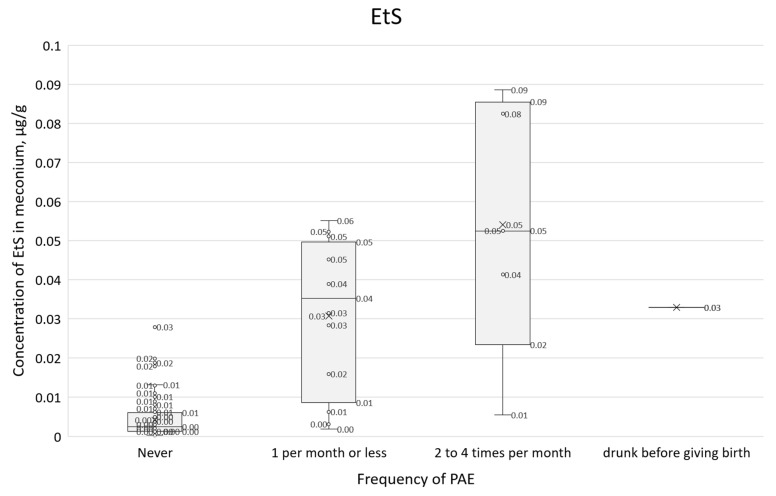
Box plot showing EtS concentrations characterized by self-reported consumption frequency (question 21) and cases involving acute maternal intoxication at parturition.

**Figure 13:**
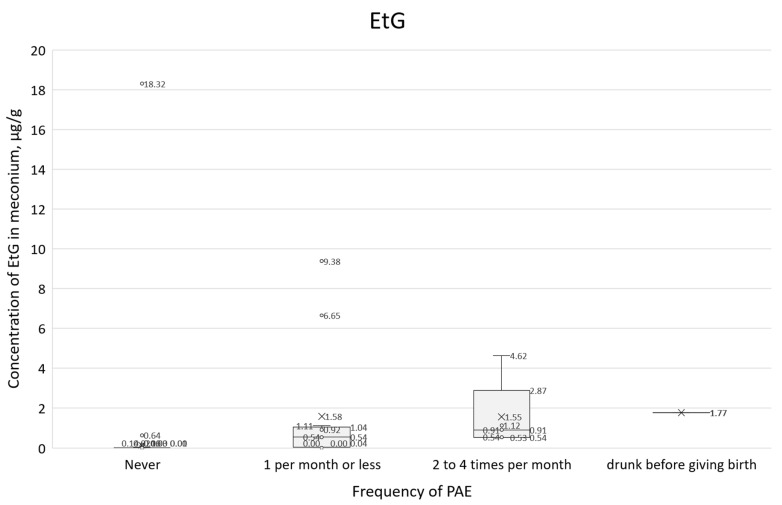
Box plot showing EtG concentrations characterized by self-reported consumption frequency (question 21) and cases involving acute maternal intoxication at parturition.

Concentrations of all PAE biomarkers were correlated with self-reported alcohol consumption frequency (question 21, Figure 11, Figure 12 and Figure 13). In these box plots, the central horizontal line denotes the median, while the lower and upper boundaries of the box represent the first (Q1, 25th percentile) and third (Q3, 75th percentile) quartiles, respectively.

### 3.4. Multiparameter Analysis of Results

#### 3.4.1. Exploratory Factor Analysis (EFA)

The study lacked a naturally occurring objective dependent variable for maternal alcohol consumption. It was not possible to collect objective, measurable data confirming alcohol consumption during pregnancy for most participants, except for women nos. 1 and 360. Instead, the data identified two questionnaire-type variables related to alcohol consumption during pregnancy, including question 21. An attempt was made to identify a threshold among the physiological variables (PVs) that would determine if specific PAE biomarker concentrations in meconium indicated a high probability of maternal alcohol consumption. A total of 12 PVs represented meconium PAE biomarker concentrations: EtS, EtG, and individual FAEE analytes—EE 12:0, EE 14:0, EE 16:0, EE 18:0, EE 18:1, EE 18:2, EE 18:3, EE 20:0, and EE 20:4. Total FAEEs were calculated as a linear combination of nine analytes, resulting in 11 unique variables. Preliminary PV analysis was conducted in two stages:

- Analysis of mutual correlations between PVs to assess their linear relationships
- EFA to identify potential latent variables among PVs

The dataset contained 478 records containing both survey responses and quantified PAE biomarker concentrations (see “Retrospective survey” and “Sampling and determination of PAE biomarkers”). For the PVs, missing data were input as zero, representing concentrations below the LOD. Due to the high skewness of the variable distributions, Spearman’s correlation coefficient was utilized for all analyses. Correlations of PV variables in the form of marked PAE biomarkers, with *p*-values indicating statistical significance, are presented in Table S5. Subsequently, EFA was performed on the 11 unique PVs (excluding the composite total FAEEs) to identify underlying latent factors. Spearman’s rank correlation was again utilized for the matrix calculations. Despite poor goodness-of-fit indices (TLI < 0.09 and RMSEA > 0.10), the analysis suggested a distinct clustering of EtS, EtG, and EE 20:0. While the EFA confirmed a poor overall model fit, it highlighted a clearer division of variables across the range of substances determined: factor loadings for EtS and EtG were significantly lower than for the FAEE analytes. Ultimately, the EFA failed to identify stable or reliable latent variables underlying the PV data.

#### 3.4.2. Cluster Analysis

The next step in the analysis was to identify potential clusters within the PV variables to help determine the dependent variable. A clustering method was applied to the same dataset used for the EFA (data with at least one non-zero observation, 11 PV variables, excluding the FAEEs_total variable). In the first stage, the number of potential clusters was examined using Euclidean metrics.

The analysis in the 2-cluster model was performed with a maximum number of iterations equal to 100 and 50 different random initial conditions. The resulting clustering did not meet the basic criterion for reliability (it is generally accepted that each cluster should contain at least 10% of the sample). Apart from this, the results specifically indicated two variables that differentiate the results for two meconium samples with EE 18:1 and EE 20:4. In the 3-cluster model, an additional cluster indicated 3 meconium samples with the highest PV variable scores. However, the frequencies of individuals assigned to clusters still did not allow this model to be considered a good fit. In the 4-cluster model, an additional cluster indicated another 6 individuals with relatively high PV scores. As with the 3-cluster model, we did not obtain a sufficient number of cases in the selected clusters; therefore, the model did not meet the necessary conditions for a good fit. Cluster analyses were also performed on standardized data, and a similar result was obtained. Neither variable grouping (EFA) nor observation grouping (clustering) allowed us to identify a dependent variable for further analysis.

#### 3.4.3. Analysis Based on the Dependent Variable

The main challenge of the analysis was the lack of a clear, established concentration cut-off point for PV that would allow us to conclude with a high degree of certainty that a woman consumed alcohol during pregnancy. Therefore, a method was employed to create a dependent variable based on the adopted parameter. For the first version of the analysis, the following assumptions were made: the “Exposure” variable would be based on the EtS concentration, with the cut-off point for high-probability prenatal alcohol exposure set at the first quartile of non-zero EtS values. Based on these assumptions, the calculated threshold for the EtS variable was 0.0013 µg/g, with *n* = 135 individuals in the entire dataset exceeding this threshold. Using this model, the relationship between the “Exposure” variable and the age of the subjects was tested using logistic regression. This relationship was found to be borderline statistically significant at a significance level of 0.05 (*p* = 0.0343). The regression coefficient was negative, indicating that as age increases, the probability of a woman being exposed to alcohol decreases.

The data were reanalyzed assuming the validity of the dependent variable “Exposure,” which was derived from a questionnaire item (question 21) asking women, “How often did you consume alcoholic beverages while pregnant?” Assuming that respondents who admitted to consuming alcohol in this variable certainly did so, a new variable, “Drinking during pregnancy,” was created and compared with the “Exposure” variable and PV. Due to the small number of non-trivial responses, a new dichotomous variable was constructed for further analysis: “no consumption” and “consumption” (*n* = 20) of alcohol during pregnancy. Fisher’s test indicated a significant relationship between “Exposure” and “alcohol consumption”; however, this relationship is not of great practical significance. As indicated by the cross-tabulation of the frequencies of both variables (Table 4), the statistical significance arises from the sample size rather than the effect size.

Data with the accepted exposure assessment cut-off indicated that 20% of individuals who declared that they consumed alcohol were not included in the chemical exposure test (EtS), which calls into question the effectiveness of differentiation based on either the EtS cut-off concentration (exposure) or declarations of alcohol consumption.

In view of the above, and recognizing that FAEEs correlate poorly with responses about self-reported alcohol consumption during pregnancy, a Cumulative Biomarker Index for two biomarkers (CBI_2_) as a multiple of exceeding the thresholds (cut-off for EtS = 0.0015 µg/g and cut-off for EtG = 0.01 µg/g) given in the literature [6,7] according to the following Formula (1):

(1) $${\mathrm{CBI}}_{2}=\left(\frac{C_{\mathrm{EtS}}}{0.0015}\right)\times \;0.5+\left(\frac{C_{\mathrm{EtG}}}{0.01}\right)\times \;0.5$$

where C_EtS_ represents EtS concentration in µg/g meconium, and C_EtG_ represents EtG concentration in µg/g meconium.

For concentrations of both biomarkers at the EtS = 0.0015 µg/g limit and the EtG = 0.01 µg/g cut-off, the proposed CBI_2_ value is 1; however, the concentrations of both biomarkers may differ. For the CIB_2_ index, an expert cut-off point of 5 was determined (CBI_2__5), which means that the cut-off concentrations C_EtS_ = 0.0075 µg/g and C_EtG_ = 0.05 µg/g were adopted (assuming an equal share in the concentrations of both biomarkers). The variables thus created were intended to indicate whether a woman was exposed to alcohol during pregnancy.

By taking the CBI_2__5 variable as the dependent variable, we were able to compare it with the other variables in the study. We then analyzed the relationship between the CBI_2__5 variable and the variables and responses indicated in the questionnaires. In some cases, due to the lack of similarity of the distributions to normal, the Mann–Whitney U test was used. The tests indicated a statistical relationship between the variables in the case of

- Answers to question 14, “In your opinion, what is the daily and safe amount of alcohol consumption for women during pregnancy?”;
- Answers to the question 18, “Do you smoke/did you smoke cigarettes?”;
- Answers to the question 20, “How often did you drink any alcoholic beverages before pregnancy?”;
- Answers to question 21, “How often did you consume alcoholic beverages while pregnant?”;
- Answers to question 22, “What type of alcoholic beverage did you drink during pregnancy? (you can select several answers): (a) wine, (b) beer” (strong alcohol was removed from the analysis due to lack of variance);
- Answers to question 23, “When during your pregnancy did you consume alcohol? (you can select several answers): (a) 1st trimester, (b) 2nd trimester, (c) 3rd trimester”;
- Answers to question 24, “What was the maximum amount of alcohol you consumed at one time during your pregnancy?”;
- Answers to question 26, “Where did you get information about toxic factors affecting the fetus? (you can select several answers)–antenatal classes”;
- Answers to the question “What is your level of education?”.

When the absence of a response (n/a) was replaced with a negative response, the result was similar to the one above. Table 5 shows the calculated CBI_2_ score together with positive responses to questions no. 21, 23, and 24 concerning alcohol consumption during pregnancy.

The above analyses of the relationships between the CBI_2__5 variable and the study variables show that only a small number of variables have significant relationships with CBI_2__5. Despite demonstrating significant correlations between these variables and the CBI_2__5 variable, a broader analysis was conducted based on a logistic regression model with Firth’s correction for the analysis of rare events (taking into account the frequencies of the CBI_2__5 variable categories). Since most of the variables in the model proved to be insignificant, it was decided to use the backward model reduction method. One variable at a time was removed from the model, which was assessed as the most burdensome for the model, and the *p*-value was adopted as the selection criterion.

Table 6 summarizes the number of responses with CBI_2_ ≥ 5 and CBI_2_ < 5 for questions 21, 23, and 24.

## 4. Discussion

The mechanism of formation of the biomarkers FAEEs, EtG, and EtS in meconium is based on the non-oxidative metabolism of ethanol, which contributes approximately 0.1–0.2% to total metabolism. When a pregnant woman consumes alcohol, it crosses the placenta into the fetal circulation, and a small portion is then converted to the aforementioned biomarkers. FAEEs are formed by the esterification of ethanol with free fatty acids, a reaction catalyzed by FAEE synthases and acyl-CoA:ethanol acyltransferase. EtG is formed by the conjugation of ethanol with glucuronic acid via UDP-glucuronosyltransferases (UGTs). EtS is formed by the conjugation of ethanol with sulfate (donor: PAPS), catalyzed by sulfotransferases (SULTs). It is assumed that FAEEs, being lipophilic substances, do not cross the placenta, so their presence directly indicates fetal metabolism [17]. In the case of EtG and EtS, hydrophilic substances, it is assumed that they can accumulate in meconium both from fetal metabolism and from the mother via the placenta. Therefore, polar metabolites of EtG and EtS have a shorter detection window, and their presence in meconium may indicate short-term exposure to PAE. However, increased concentrations of nonpolar FAEEs in meconium may indicate longer-term PAE. Meconium begins to accumulate between the 12th and 16th weeks of pregnancy, and the highest biomarker accumulation occurs in the second and third trimesters (especially from approximately the 20th week). Therefore, meconium analysis allows for the assessment of PAE in the last 4–5 months of fetal life. However, it does not reflect alcohol consumption in the first trimester, when meconium is not yet formed. Table 3 shows a correlation for survey question 23: for 4 women (no. 94, 186, 193, 390) who reported alcohol consumption exclusively in the first trimester, PAE biomarker values are below the LOD or relatively low. These results confirm the possibility of detecting PAE biomarkers only from the time of meconium formation.

Regarding FAEEs, the authors of previous studies selected different fatty acid ethyl esters for determination in meconium, so comparison of total FAEE concentrations is limited and difficult. Himes et al.’s study showed that most FAEEs in authentic meconium were degraded after 12 h at room temperature and 72 h at 4 °C, with high interindividual variability [18]. Furthermore, freeze–thaw stability experiments showed greater stability of EtG and EtS concentrations ≤ 11% of preliminary results [18,19]. These results suggest that FAEE may not be a suitable marker of long-term ethyl alcohol concentration due to susceptibility to degradation resulting from environmental conditions. Furthermore, Chan et al. showed that olive oil consumption during pregnancy was associated with increased total FAEE concentrations in meconium [20]. Most authors did not establish concentration thresholds for FAEEs, EtG, and EtS due to the small number of results with the assumed PAE [9,10], therefore the following discussion of the results should also be treated with great caution.

The cutoff values proposed by various authors [7,8,9,10,11] for total FAEE indicating PAE, as shown in Table 1, were in the range of 0.111–0.600 µg/g of meconium. In our study, 12 women admitted to consuming alcohol in the second and third trimester of pregnancy, with total FAEE concentrations (Table 3) ranging from 0.0018 to 14.1468 µg/mL (*n* = 10; for 2 women, concentrations above the LOQ were not found). Additionally, 2 women who were definitely exposed to PAE and who did not complete the questionnaire, no. 350 (drunk before giving birth) and no. 1 (alcohol until the 20th week of pregnancy), had very high total FAEE concentrations of 5.5832 and 11.5971 µg/g, respectively. Four women reported alcohol consumption exclusively in the first trimester, and no total FAEE concentrations exceeding the LOQ were detected, with the exception of one woman who had a concentration of 0.0513 µg/g.

The cutoff values for EtG suggested by various authors [7,8,9,10,11] indicating PAE, as presented in Table 1, ranged from 0.030 to 21.06 µg/g of meconium. In our study, 12 women reported drinking alcohol in the second and third trimesters of pregnancy, with EtG concentrations (Table 3) ranging from 0.0116 to 6.6510 µg/mL (*n* = 12). Additionally, two women who were definitely exposed to PAE and who did not complete the questionnaire, no. 350 (drunk before giving birth) and no. 1 (alcohol until the 20th week of pregnancy) had very high EtG concentrations of 1.7700 and 22.8950 µg/g, respectively. Four women reported drinking alcohol exclusively in the first trimester, and of these, two had no EtG levels exceeding the LOQ, while two had results of 0.5441 µg/g (no. 390 with alcohol consumption 1 per month) and 4.6210 µg/g (no. 94 with alcohol consumption 2–4 times per month).

The cutoff values for EtS proposed by various authors [7,8,9,10,11], indicating PAE, shown in Table 1, were in the range of 0.000182–0.03055 µg/g of meconium. In our study, 12 women admitted to drinking alcohol in the second and third trimester of pregnancy, with EtS concentrations (Table 3) ranging from 0.0062 to 0.00825 µg/mL (*n* = 11), and for 1 woman, the concentration was not above the LOQ. Additionally, 2 women who were definitely exposed to PAE and who did not complete questionnaires no. 350 (drunk before giving birth) and no. 1 (alcohol until the 20th week of pregnancy) had very high EtS concentrations of 0.0329 and 14.4900 µg/g, respectively. Four women reported drinking alcohol exclusively in the first trimester. Two women did not have EtS concentrations exceeding the LOQ, while two women had EtS levels of 0.0523 µg/g (no. 390) and 0.0886 µg/g (no. 94).

The differences between our results and those reported in the literature are not significant, but it must be acknowledged that authors rarely clearly indicate cutoff values for PAE biomarkers, which is also due to the high variability within study groups and the relatively small number of positive PAE results from survey studies.

A comparison of total FAEE and EtS concentrations in meconium, stratified by self-reported alcohol consumption during pregnancy (at least one positive answer to questions 21, 22, or 23) and abstinence (Figure 8a), demonstrated that the proposed cut-off values for these biomarkers were extremely low, resulting in poor discrimination between the exposed and non-exposed groups. Assuming the accuracy of self-reported PAE (positive response to any of the questions 21, 22, and 23), the EtS cut-off should be increased at least five-fold to 0.0075 µg/g. This adjustment is necessary to improve the differentiation between exposed and non-exposed cases and to enhance overall diagnostic specificity. For total FAEEs, the high number of positive and negative results suggested that merely increasing the cut-off concentration would not achieve the desired diagnostic effect. As shown in Figure 8b, excluding cases of declared abstinence revealed greater consistency between self-reports and chemical analysis results for EtS than for total FAEEs.

A comparison of total FAEE and EtG concentrations in meconium, stratified by alcohol consumption during pregnancy (at least one positive answer to questions 21, 22, or 23) and abstinence (Figure 9a), demonstrated that the proposed cut-off values for both biomarkers were also extremely low. As in the case of EtS (Figure 9b), excluding cases of declared abstinence revealed greater consistency between self-reports and chemical analysis results for EtG than for total FAEEs. Increasing the EtG cut-off at least five-fold to 0.05 µg/g improves the differentiation between the exposed and non-exposed groups.

A comparison of EtS and EtG determination (Figure 10a) revealed a greater concentration of results near the origin for both EtS (0 µg/g) and EtG (0 µg/g). This indicated a better correlation with PAE for these two biomarkers relative to total FAEEs. Furthermore, excluding cases of declared abstinence (Figure 10b) confirmed that EtS and EtG best reflected PAE. In this figure, meconium samples from women reporting alcohol consumption only in the first trimester (meconium samples numbered 55, 65, 190, and 422)—or in the case of a woman with conflicting reports (sample 166, reported no alcohol consumption during pregnancy but consumed beer in the third trimester)—showed extremely low EtG but elevated EtS. Notably, this specific, contradictory sample contained EtS and EtG concentrations above the proposed cut-off point.

Four women (no. 16, 54, 166, 405) who responded to question 21 that they did not consume alcohol during pregnancy, in subsequent questions indicated consumption of wine, beer, vodka, or alcohol in the first or third trimester of pregnancy. PAE biomarker results for these four individuals are included in Table 3 but were not included in the statistical analyses and final PAE percentage estimates. This inconsistency in women’s responses is one of the sources of uncertainty in estimating the PAE scale.

A summary of all survey responses (Table 3) in which women indicated alcohol consumption during pregnancy (in at least one of the three questions 21–24) was characterized by inconsistency. The most surprising were the responses of four women (nos. 16, 54, 166, and 405), who answered “No” to question 21 regarding alcohol consumption, yet subsequently indicated consumption of wine, beer, or vodka in the first or third trimester. Based on the survey results and analyzing questions 21–24 separately, the following number of women admitted to consuming alcohol during pregnancy: *n* = 20 (positive answer to question 21), *n* = 19 (positive answer to question 22), *n* = 19 (positive answer to question 23), and *n* = 16 (positive answer to question 24). As shown in Figure 4, question 22 allowed for multiple answers. Woman no. 54 declared that she had consumed all three alcoholic beverages (answers a, b, and c), while women nos. 186 and 237 declared that they had consumed beer and wine (answers a and b). Similarly, in question 23, the number of answers exceeded the number of respondents; women nos. 42 and 363 declared that they consumed alcoholic beverages in all trimesters (answers a, b, and c), and women nos. 4 and 244 declared that they consumed alcohol in the 2nd and 3rd trimesters (answers b and c).

Based on the positive results from questions 21–24 (Figure 7), only 12 women provided consistent answers across all four questions (nos. 4, 42, 94, 186, 237, 244, 278, 298, 363, 390, 407, and 422). The remaining positive responses regarding PAE were inconsistent, appearing in only 1, 2, or 3 of the questions. Therefore, if a positive response to any of the questions 21–24 is assumed to be accurate, a maximum of 24 women consumed alcohol during pregnancy.

The summary of survey results with positive responses to question 21, in conjunction with the total FAEE concentration results in the box-plot (Figure 11), did not correlate with the predicted concentration increase trend. For those who declared that they did not consume alcohol during pregnancy, the median total FAEE was 0.14 µg/g, and the interquartile range (IQR) was 0.03–0.40 µg/g. For those who declared alcohol consumption of 1 per month or less, the median was 0.62 µg/mL, and the IQR was 0.13–2.60, while for those who declared consumption of 2–4 times per month, the median was 0.02 µg/g and the IQR was 0.00–0.05 µg/g.

A similar comparison of survey responses with a positive answer to question 21, in conjunction with the EtS concentration results (Figure 12), showed a clear upward trend, and the IQR range overlapped to a lesser extent than for total FAEEs. For those who declared that they did not consume alcohol during pregnancy, the median EtS was 0.002 µg/g, and the IQR was 0.001–0.006 µg/g. For those who declared alcohol consumption of 1 per month or less, the median was 0.035 µg/mL, and the IQR was 0.009–0.050, while for those who declared consumption of 2–4 times per month, the median EtS was 0.053 µg/g and the IQR was 0.023–0.086 µg/g.

A similar comparison of survey responses with a positive answer to question 21, in conjunction with the EtG concentration results (Figure 13), showed a clear upward trend, and the IQR range overlapped to a lesser extent than for total FAEEs. For those who declared that they did not consume alcohol during pregnancy, the median EtG was 0.008 µg/g, and the IQR range was 0.004–0.016 µg/g. For those who declared alcohol consumption 1 per month or less, the median was 0.54 µg/mL, and the IQR was 0.07–0.97 µg/g, while for those who reported consuming 2–4 times per month, the median EtS was 0.91 µg/g, and the IQR was 0.54–1.12 µg/g.

The declared alcohol consumption in the first trimester of pregnancy (Table 5) resulted in a relatively higher frequency of CBI_2_ scores of 0 or 1 compared to the declared alcohol consumption in the second and third trimesters. This suggests a potential inability to detect PAE based on declared consumption in the first months of pregnancy, likely due to the lack of biomarker accumulation in meconium. In most cases, the calculated CBI_2_ values exceeded 1 several times; however, analysis of the cut-off points for this indicator suggests that PAE can be reliably differentiated from a value of 5.

The following limitations should also be noted in the results of our research work. The grant project initially assumed the collection of approximately 1000 meconium samples for chemical analysis of FAEEs, EtG, and EtS biomarkers. It was initially assumed that FASD in Poland may affect approximately 2–10% of the population (as we mentioned in the Introduction), so 1000 samples could contain 20 to 100 positive results for PAE. Sample calculations for the number of samples for EtG (the most frequently determined PAE biomarker) in meconium using the Receiver Operating Characteristic (ROC) method (2):

(2) $$n=\frac{{\left(Z_{1-\propto }+Z_{1-\beta }\right)}^{2}\times (1+r)}{{\left(AUC-0.5\right)}^{2}\times r}$$

where:

- AUC—expected value under the curve (0.85);
- r—ratio of the number of people exposed to PAE to those not exposed, assumed = 0.1:1;
- β—type II error (power 1 − β = 0.80), hence Z_1−β_ = 0.84;
- α—significance level (0.05), hence Z_1−α_ = 1.96.

Based on the above data, approximately 704 samples would need to be collected, assuming that 10% of the female population was exposed to PAE. If the percentage of women exposed to PAE was five times lower (approximately 2%), this number would be approximately 3264 samples. The project was planned for four years and was implemented over five years (from 5 October 2016 to 4 October 2021), extended due to the delayed purchase of GC-MS apparatus and COVID-19. When we began the project, we did not know the exact scale of the FASD problem in Poland. The number of biomarker results obtained for 478 meconium samples is not sufficient to accurately estimate the cutoff concentrations of FAEES, EtG, and EtS, but it can be approximated in relation to the real scale of the FASD problem and the actual biomarker cutoff values.

The survey questionnaire used in the project was prepared and validated by experienced researchers from our team based on a literature review (expert validation) as stated in the Section 2. A potential source of error during the survey administration phase could have been related to the excessive number of questions used in the questionnaire. These could have been too complex or difficult for postpartum women to answer. The surveys were anonymous, but some women may have feared stigmatization or doubted the anonymity of the research. Polish society is characterized by a relatively low level of trust in institutions, which may impact the accuracy and reliability of the completed surveys. During the survey, women were provided with a safe and comfortable environment to complete the surveys, while ensuring complete anonymity. After collecting meconium samples, they were immediately placed in a −20 °C freezer to minimize degradation of PAE biomarkers. The methodology for determining FAEEe, EtG, and EtS was developed and validated before testing actual samples.

## 5. Conclusions

The study underscores the need to implement meconium biomarker measurement as an objective and independent method for identifying PAE. Survey data suggest a significant underestimation of PAE, as the reported frequency of alcohol consumption during pregnancy was consistently low (approximately 4%) and potentially unreliable. The most reliable PAE information came from combined biomarker analysis, particularly EtG and EtS, which correlated better with reported alcohol consumption, with FAEEs as a secondary marker. Multivariate analyses yielded limited actionable insights due to low case numbers and results often below the LOQ. The proposed cumulative biomarker index, CBI2 ≥ 5, suggests that PAE affected approximately 3% of the study population, highlighting self-report unreliability. Standardization of analytical methods and validation of biomarker cut-off values remain key for implementing PAE biomarkers in routine clinical diagnostics.

## Figures and Tables

**Table 1:** Prevalence of PAE based on meconium biomarkers.

Population (Author, Year)	Meconium Biomarkers	Self-Reported Alcohol Consumption	Results: Cut-off, Range, and Median of Concentrations
108 meconium samples (33 with no PAE): US Northern Plains and Cape Town, South Africa (Himes et al., 2015) [7]	- 9 FAEEs: ethyl linolenate (EE * 18:3), ethyl laurate (EE 12:0), ethyl myristate (EE 14:0), ethyl palmitoleate (EE 16:1), ethyl arachidonate (EE 20:4), ethyl linoleate (EE 18:2), ethyl palmitate (EE 16:0), ethyl oleate (EE 18:1), and ethyl stearate (EE 18:0) - EtG - EtS	TLFB ** interviews	- PAE by EtG: ≥30 ng/g (highest agreement with self-reported PAE in the second half of pregnancy) - No association between EtG and EtS - Negligible association between EtG and individual/summed FAEEs - Moderate-to-strong associations among individual FAEEs - Modest correlations between ethyl oleate/EtG and ethyl oleate/EtS
99 meconium samples: 49 Italy and 50 Spain (Morini et al., 2010) [8]	- 7 FAEEs: EE 16:0, EE 16:1, EE 18:0, EE 18:1, EE 18:2, EE 18:3, and arachidonic acid ethyl ester (EE 20:0) - EtG - EtS	Medical records	- PAE by EtG (>LOD): 82.8% in both cohorts - Alcohol-exposed group (*n* = 49): Cut-off: EtG ≥ 1.5 nmol/g (≥330 ng/g) Range: 4.1–49.0 nmol/g Median: not applicable - PAE by EtS (>LOD): 19.2% in both cohorts - Alcohol-exposed group (*n* = 49): Cut-off: EtS ≥ 0.012 nmol/g (≥1.6 ng/g) Range: 0.0–26.5 nmol/g Median: not applicable - PAE by FAEEs (≥2 nmol/g): 10.4% in Italy and 34% in Spain (22.2% combined) - Significant correlations between EtG and ethyl laurate (EE 12:0), ethyl linolenate (EE 18:3), ethyl linoleate (EE 18:2), ethyl oleate (EE 18:1), and total FAEEs - Significant correlations between EtS and ethyl arachidonate (EE 20:4), ethyl linoleate (EE 18:2), ethyl palmitate (EE 16:0), ethyl oleate (EE 18:1), and total FAEEs
185 meconium samples: 80 Italy and 105 Spain (Morini et al., 2010) [9]	- 7 FAEEs: EE 16:0, EE 16:1, EE 18:0, EE 18:1, EE 18:2, EE 18:3, and EE 20:0 - EtG - EtS	Questionnaire administered at each trimester	- Nonexposed group (*n* = 42) *** by EtG: Cut-off: <2 nmol/g (<440 ng/g) Range: 0.022–1.870 nmol/g Median: 0.100 nmol/g - Nonexposed group (*n* = 52) *** by EtG: Range: 0.022–1.570 nmol/g Median: 0.140 nmol/g - Unconfirmed exposure status group (*n* = 23) *** by EtG: Range: 0.022–0.650 nmol/g Median: 0.160 nmol/g - Unconfirmed exposure status group (*n* = 36) *** by EtG: Range: 0.040–10.500 nmol/g Median: 0.250 nmol/g - Confirmed exposure status group (*n* = 5) *** by EtG: Range: 2.510–95.720 nmol/g Median: 7.240 nmol/g - Nonexposed group (*n* = 8) *** by EtS: Cut-off: not established Range: 0.008–0.020 nmol/g Median: 0.010 nmol/g - Nonexposed group (*n* = 15) *** by EtS Cut-off: not established Range: 0.010–0.030 nmol/g Median: 0.020 nmol/g - Unconfirmed exposure status group (*n* = 12) *** by EtS: Cut-off: not established Range: 0.010–0.520 nmol/g Median 0.020 nmol/g - Unconfirmed exposure status group (*n* = 7) *** by EtS: Cut-off: not established Range: 0.010–0.110 nmol/g Median: 0.020 nmol/g - Confirmed exposure status group (*n* = 5) *** by EtS: Cut-off: not established Range: 0.014–0.235 nmol/g Median: 0.330 nmol/g - Alcohol-exposed group (*n* = 4) *** by FAEEs: Cut-off: ≥2 nmol/g (≥600 ng/g) Range: 0.200–0.370 nmol/g Median: 0.290 nmol/g - Alcohol-exposed group (*n* = 18) *** by FAEEs: Cut-off: ≥2 nmol/g (≥600 ng/g) Range: 0.240–66.020 nmol/g Median: 2.460 nmol/g - Median, min–max, and 97.5 percentile concentrations for each biomarker presented by nonexposed and unconfirmed exposure status groups - Meconium EtG (≥2 nmol/g) showed superior sensitivity and specificity to EtS and FAEEs
177 meconium samples: 96 Italy and 81 Spain (Pichini et al., 2009) [10]	- 7 FAEEs: EE 16:0, EE 16:1, EE 18:0, EE 18:1, EE 18:2, EE 18:3, and EE 20:0 - EtG - EtS	Questionnaire	- PAE by EtG (>5 ng/g): 81.5% in Italy and 95.5% in Spain - PAE by EtS (>1 ng/g): 46.9% in Italy and 51.9% in Spain. - Alcohol-exposed group (*n* = 96) by EtS: Cut-off: not established Range: 1–65.2 ng/g Median: 15.6 ng/g - Alcohol-exposed group (*n* = 81) by EtS: Cut-off: not established Range: 1.1–437.5 ng/g Median: 101.5 ng/g - PAE by FAEEs (≥2 nmol/g): 8% in Italy and 42% in Spain - Alcohol-exposed group (*n* = 96) by FAEEs: Cut-off: ≥2 nmol/g (≥600 ng/g) Range: 2.8–3.5 ng/g Median: Not applicable - Alcohol-exposed group (*n* = 81) by FAEEs: Cut-off: ≥2 nmol/g (≥600 ng/g) Range: 2.2–324.7 ng/g Median: not applicable
Review of PAE studies (through 31 December 2015; PubMed, n = 44) (Dos Santos, 2016) [11]	Most frequently observed combinations of FAEEs in meconium comprise either a 4-compound set (EE 16:1, EE 18:0, EE 18:1, and EE 18:2) or a 7-compound set (EE 16:0, E16:1, EE 18:0, EE 18:1, EE 18:2, EE18:3, and EE 20:4)	Mothers and their newborns, whose meconium was analyzed for FAEEs	- Ethyl oleate (EE 18:1): Probably the most prevalent biomarker - Ethyl linoleate (EE 18:2): Most frequent biomarker with the highest concentrations - Total FAEEs: Summed values are more reliable for PAE interpretation than individual esters - Heavy maternal consumption group by FAEEs: Cut-off: ≥600 ng/g Range: not applicable Median: not applicable

Note: Total FAEE concentrations are based on varying combinations of individual compounds as reported by the respective studies. * EE—ethyl ester; **—TLFB-timeline follow-back method; *** Only samples with measurable concentration (>LLOQ) of EtG and EtS were considered for this calculation. For comparison with the present study, nmol units were converted to µg; 1 nmol of EtG and EtS corresponds to 0.22 and 0.13 µg, respectively. Due to varying molar masses of individual FAEEs, 1 nmol of total FAEEs is estimated as 0.3 µg.

**Table 2:** Summary of chemical analysis results for FAEEs, EtS, and EtG in meconium (*n* = 478).

Parameter	EE 12:0	EE 14:0	EE 16:0	EE 18:2	EE 18:1	EE 18:3	EE 18:0	EE 20:4	EE 20:0	Total FAEEs	EtS	EtG
*n* ≥ LOQ (%)	19 (4.0)	27 (5.6)	92 (19.2)	171 (35.8)	123 (25.7)	78 (16.3)	38 (7.9)	105 (22.0)	12 (2.5)	n/a *	150 (31.4)	154 (32.2)
*n* LOD-LOQ (%)	8 (1.7)	18 (3.8)	77 (16.1)	27 (5.6)	26 (5.4)	16 (3.3)	47 (9.8)	17 (3.6)	15 (3.1)	n/a *	31 (6.5)	22 (4.6)
*n* < LOD (%)	451 (94.4)	433 (90.6)	309 (64.6)	280 (58.6)	329 (68.8)	384 (80.3)	393 (82.2)	356 (74.5)	451 (94.4)	n/a *	297 (62.1)	302 (63.2)
C_min_ (µg/g)	0.0051	0.0050	0.0050	0.0255	0.0075	0.0074	0.0052	0.0253	0.0078	0.0018	0.0010	0.0026
C_max_ (µg/g)	0.6804	1.1210	2.0050	4.8360	8.7170	4.3600	0.7560	6.5000	0.0878	14.1468	14.49	22.90
C_average_ (µg/g)	0.1577	0.1323	0.1244	0.3086	0.3542	0.1620	0.0882	0.3892	0.0302	0.6297	0.1054	0.4842
SD (µg/g)	0.2355	0.2652	0.3074	0.5151	1.1194	0.5680	0.1597	0.8046	0.0260	1.7374	1.1825	2.5403
C_median_ (µg/g)	0.0210	0.0227	0.0222	0.1802	0.0321	0.0228	0.0175	0.1520	0.0192	0.1382	0.0037	0.0123
LOD (µg/g)	0.0010	0.0008	0.0012	0.0075	0.0025	0.0021	0.0013	0.0074	0.0020	n/a *	0.0002	0.0008
LOQ (µg/g)	0.0030	0.0024	0.0036	0.0225	0.0075	0.0063	0.0039	0.0222	0.0060	n/a *	0.0006	0.0024

*—not applicable.

**Table 3:** Summary of maternal self-reported alcohol consumption in questions 21–24 and corresponding PAE biomarker levels in meconium samples. Plus sign (+) means positive answer for the questions no. 22 and 23; blank box means no answer or negative response regarding PAE.

No.	Frequency of Alcohol Consumption or PAE Resulting from Circumstances of Childbirth (No. 21)	Type of Alcohol Consumed During Pregnancy (No. 22)			Trimester of Alcohol Exposure (No. 23)			Quantity Per Occasion (No. 24)	Total FAEEs, µg/g	EtS, µg/g	EtG, µg/g
		Wine (*n* = 14)	Beer (*n* = 8)	Vodka (*n* = 1)	1st (*n* = 8)	2nd (*n* = 7)	3rd (*n* = 10)	(*n* = 16)			
16	Never (*n* = 4)		+						n.d. **	n.d. **	n.d. **
54		+	+	+	+			(c) **	0.06244	n.d. **	0.02102
166			+				+		0.5274	0.0181	0.0647
405			+		+			(a) ***	n.d. **	0.0031	n.d. **
4	1 per month or less (*n* = 15)	+				+	+	(a)	0.5601	0.0389	0.6870
42		+			+	+	+	(a)	3.1193	0.0452	0.9653
55							+		14.1468	0.0062	0.0649
65			+				+		0.1298	0.0315	0.1372
186		+	+		+			(a)	n.d. **	n.d. **	n.d. **
190									n.d. **	0.0018	0.0041
193			+		+				n.d. **	n.d. **	n.d. **
233							+	(a)	0.4082	n.d. **	0.0116
244		+				+	+	(a)	0.0829	0.0394	0.9175
257									0.0522	n.d. **	0.0048
258									2.2148	0.0159	9.3790
278		+					+	(a)	2.6033	0.0283	1.1105
298		+				+		(c)	1.4218	0.0511	6.6510
390		+			+			(a)	n.d. **	0.0523	0.5441
422		+				+		(a)	0.6155	0.0551	0.0968
94	2–4 times per month (*n* = 5)	+			+			(a)	0.0513	0.0886	4.6210
149		+						(a)	0.0211	0.0054	0.5427
237		+	+			+		(a)	0.0018	0.0525	0.9102
363		+			+	+	+	(a)	n.d. **	0.0825	1.1244
407		+					+	(a)	n.d. **	0.0413	0.5315
350	Drunk before giving birth *	n/a ***	n/a ***	n/a ***	n/a ***	n/a ***	n/a ***	n/a ***	5.5832	0.0329	1.7700
1	Exposure through gestational week 20 *	n/a ***	n/a ***	n/a ***	n/a ***	n/a ***	n/a ***	n/a ***	11.5971	14.4900	22.8950

* The questionnaire was incomplete, and PAE resulted from the circumstances of childbirth. ** (c)—3 small beers, or 3 glasses of wine, or 3 glasses of vodka, or 3 cocktails. *** (a)—1 small beer, or 1 glass of wine, or 1 glass of vodka, or 1 cocktail; **—not detected; ***—not applicable.

**Table 4:** Contingency table with the variables “Exposure” (EtS) and self-reported “alcohol consumption” in the questionnaire (question 21).

	No Consumption	Alcohol Consumption
No exposure	336	4
Exposure	118	16

**Table 5:** Comparison of the calculated CBI_2_ score with survey responses on alcohol consumption in questions 21, 23, and 24.

No of Sample	Answer to Question 21 (*n* = 20)	CBI_2_
4	Once a month or less frequently	47
42	Once a month or less frequently	62
55	Once a month or less frequently	5
65	Once a month or less frequently	17
94	2–4/month	259
149	2–4/month	29
186	Once a month or less frequently	0
190	Once a month or less frequently	1
193	Once a month or less frequently	0
233	Once a month or less frequently	1
237	2–4/month	62
244	Once a month or less frequently	58
257	Once a month or less frequently	0
258	Once a month or less frequently	474
278	Once a month or less frequently	64
298	Once a month or less frequently	349
363	2–4/month	82
390	Once a month or less frequently	44
407	2–4/month	39
422	Once a month or less frequently	22
	**Answer to question 23 (*n* = 8)**	
42	Alcohol consumption in the 1st trimester	62
54		1
94		259
186		0
193		0
363		82
390		44
405		1
	**Answer to question 23 (*n* = 7)**	
4	Alcohol consumption in the 2nd trimester	47
42		62
237		62
244		58
298		349
363		82
422		22
	**Answer to question 23 (*n* = 8)**	
4	Alcohol consumption in the 3rd trimester	47
42		62
55		5
65		17
166		9
233		1
244		58
407		39
	**Answer to question 24 (*n* = 16)**	
4	1 small beer or 1 glass of wine, or 1 shot of vodka, or 1 cocktail	47
42	1 small beer or 1 glass of wine, or 1 shot of vodka, or 1 cocktail	62
54	3 small beers of 3 glasses of wine, or 3 shots of vodka, or 3 cocktails	1
94	1 small beer or 1 glass of wine, or 1 shot of vodka, or 1 cocktail	259
149	1 small beer or 1 glass of wine, or 1 shot of vodka, or 1 cocktail	29
186	1 small beer or 1 glass of wine, or 1 shot of vodka, or 1 cocktail	0
233	1 small beer or 1 glass of wine, or 1 shot of vodka, or 1 cocktail	1
237	1 small beer or 1 glass of wine, or 1 shot of vodka, or 1 cocktail	62
244	1 small beer or 1 glass of wine, or 1 shot of vodka, or 1 cocktail	58
278	1 small beer or 1 glass of wine, or 1 shot of vodka, or 1 cocktail	64
298	3 small beers or 3 glasses of wine, or 3 shots of vodka, or 3 cocktails	349
363	1 small beer or 1 glass of wine, or 1 shot of vodka, or 1 cocktail	82
390	1 small beer or 1 glass of wine, or 1 shot of vodka, or 1 cocktail	44
405	1 small beer or 1 glass of wine, or 1 shot of vodka, or 1 cocktail	1
407	1 small beer or 1 glass of wine, or 1 shot of vodka, or 1 cocktail	39
422	“a” or “c” answer to question 24	22

**Table 6:** Summary of chemical analysis results CBI_2_ ≥ 5 and CBI_2_ < 5 and responses to survey questions 21, 23, and 24 (based on Table 5).

Question No	CBI_2_ ≥ 5 (Positive)		CBI_2_ < 5 (Negative)	
	Range	*n*	Range	*n*
21	- 1/month or less frequently: 5–474	10	- 1 month or less: 0–1	5
	- 2–4/month: 29–259	5	- 2–4/month: none	0
23	- PAE in the 1st trimester: 44–259	4	- PAE in the 1st trimester: 0–1	4
	- PAE in the 2nd trimester: 22–349	7	- PAE in the 2nd trimester: none	0
	- PAE in the 3rd trimester: 5–62	7	- PAE in the 3rd trimester: 1	1
24	- 1 small beer or 1 glass of wine, or 1 shot of vodka, or 1 cocktail: 29–259	10	- 1 small beer or 1 glass of wine, or 1 shot of vodka, or 1 cocktail: 0–1	3
	- 3 small beers or 3 glasses of wine, or 3 shots of vodka, or 3 cocktails: 22–349	2	- 3 small beers or 3 glasses of wine, or 3 shots of vodka, or 3 cocktails: 1	1

## Data Availability

The original contributions presented in this study are included in the article/Supplementary Materials. Further inquiries can be directed to the corresponding author.

## Supplementary Materials

The following supporting information can be downloaded at https://www.mdpi.com/article/10.3390/toxics14020155/s1. Table S1: Author’s survey questionnaire (English version); Table S2: Results of survey questions 1–5 (n = 478) in pregnant women at the Neonatology Clinic of the Medical University of Gdańsk, Pomeranian Province (16 June 2019, to 24 April 2020); Table S3: Results of survey questions 6a–6o (n = 478) in pregnant women at the Neonatology Clinic of the Medical University of Gdańsk, Pomeranian Province (16 June 2019, to 24 April 2020); Table S4: Results of survey questions 7–11d (n = 478) in pregnant women at the Neonatology Clinic of the Medical University of Gdańsk, Pomeranian Province (16 June 2019, to 24 April 2020); Table S5: Results of survey questions 12–18 (n = 478) in pregnant women at the Neonatology Clinic of the Medical University of Gdańsk, Pomeranian Province (16 June 2019, to 24 April 2020); Table S6: Results of survey questions 19a–23c (n = 478) in pregnant women at the Neonatology Clinic of the Medical University of Gdańsk, Pomeranian Province (16 June 2019, to 24 April 2020); Table S7: Results of survey questions 24–26 (n = 478) in pregnant women at the Neonatology Clinic of the Medical University of Gdańsk, Pomeranian Province (16 June 2019, to 24 April 2020); Table S8: Demographic data of pregnant women (n = 478) surveyed (1–7 g) at the Neonatology Clinic of the Medical University of Gdańsk, Pomeranian Province (16 June 2019, to 24 April 2020); Table S9: Results of GC-MS-SIM and LC-ESI-MS-MS chemical analyses (mass concentration units, µg/g) for infant meconium (n = 478) collected on the first day after birth at the Neonatology Clinic of the Medical University of Gdańsk, Pomeranian Province (16 June 2019, to 24 April 2020); Table S10: Results of GC-MS-SIM and LC-ESI-MS-MS chemical analyses (molar concentration units, nmol/g) for infant meconium (n = 478) collected on the first day after birth at the Neonatology Clinic of the Medical University of Gdańsk, Pomeranian Province (16 June 2019, to 24 April 2020); Table S11: Summary of survey questionnaire (n = 478, response rates by individual question).

## Abbreviations

The following abbreviations are used in this manuscript:

CBI_2_	Cumulative biomarker index for 2 biomarkers
CBI_2__5	Cumulative biomarker index for 2 biomarkers equals 5
DAC	Department of Analytical Chemistry
EE 12:0	Ethyl laurate
EE 14:0	Ethyl myristate
EE 16:0	Ethyl palmitate
EE 16:1	Ethyl palmitoleate
EE 18:0	Ethyl stearate
EE 18:1	Ethyl oleate
EE 18:2	Ethyl linoleate
EE 18:3	Ethyl linolenate
EE 20:0	Ethyl arachidate
EE 20:4	Ethyl arachidonate
EFA	Exploratory factor analysis
EtG	Ethyl glucuronide
EtS	Ethyl sulfate
FAEEs	Fatty acid ethyl esters
FASD	Fetal alcohol spectrum disorders
GC-MS	Gas chromatography–mass spectrometry
GUT	Gdańsk University of Technology
IQR	Interquartile range
LC-MS/MS	Liquid chromatography–tandem mass spectrometry
LOD	Limits of detection
LOQ	Limits of quantification
MC	Maternity clinic
MUG	Medical University of Gdańsk
NC	Neonatology Clinic
PAE	Prenatal alcohol exposure
PV	Physiological variable
RMSEA	Root mean square error of approximation
SD	Standard deviation
TLI	Tucker–Lewis index
UCC	University Clinical Center
