# Supplementary material for: Estimation of Prenatal Alcohol Exposure: Comparison of Retrospective Survey and Measurement of Fatty Acid Ethyl Esters, Ethyl Sulfate, and Ethyl Glucuronide Concentrations in Neonatal Meconium
Source: Toxics. 2026 Feb 4;14(2):155. doi: 10.3390/toxics14020155 (PMC12944540; doi:10.3390/toxics14020155)
Supplement: Supplementary file 1 [file toxics-14-00155-s001.zip › Table S01 questionnaire.pdf]

Table S1. Author's survey questionnaire (English version)

**Dear Madame,**<br><br>We kindly ask you to participate in the study conducted by Medical University of Gdańsk and Gdańsk University of Technology. The aim of the study is to learn opinions about the types of substances consumed by pregnant women and to learn about women's behavior. The answers to the survey are confidential, obtained information will only be used for the needs of the scientific study and statistical analysis.<br><br>**Thank you very much for participating in the study.**
**1. Which pregnancy does the current baby come from?**<br>a) 1st pregnancy<br>b) 2nd pregnancy<br>c) 3rd pregnancy<br>d) 4th pregnancy<br>e) 5th or more pregnancy
**2. How many times have you given birth so far (except giving birth now)?**<br>a) 0<br>b) 1<br>c) 2<br>d) 3<br>e) 4 or more times
**3. In which week of pregnancy was the current baby born?**<br>a) At term, more than 37 weeks of gestation .<br>b) Preterm, less than 37 weeks of gestation.<br>c) 42 weeks of gestation or more.
**4. What were the baby's measurements at birth?**<br>a) Birth weight ………<br>b) Head circumference ………<br>c) Body length ………
**5. What was the Apgar score in the first minute of life?**<br>a) 8 - 10<br>b) 4 - 7<br>c) 0 - 3
**6. Have you had any chronic diseases during your current pregnancy, such as:**<br>a) pre-pregnancy hypertension<br>b) hypertension developing during pregnancy<br>c) pre-pregnancy diabetes<br>d) gestational diabetes (developing during pregnancy)<br>e) chronic kidney disease<br>f) hypothyroidism<br>g) bronchial asthma<br>h) allergy<br>i) other chronic lung diseases<br>j) other chronic diseases of the cardiovascular system<br>k) anemia<br>l) cancer<br>m) chronic infectious diseases: hepatitis B, hepatitis C, AIDS,

n) depression
o) other

7. Have you had a miscarriage/miscarriages?

a) yes
b) no

8. Have you used any medicine during this pregnancy? If so, please list which ones

a) no
b) yes, as follows: ……………………………………………………

9. Have you colored your hair during this pregnancy?

a) yes
b) no

10. Have you used sweeteners during this pregnancy?

a) yes,
b) no.

11. Are you on a diet or have you been on a diet? You can select more than one answer.

Diet	Yes, pre-pregnancy	Yes, pre-pregnancy, and I stopped during pregnancy	I am still on diet	I am not on a diet
a) lactose-free				
b) gluten-free				
c) vegetarian/vegan				
d) another				

12. Have you used/taken the following medications during your current pregnancy? (you can mark several answers, you can choose not to mark any answer).

a) sedative medicines prescribed by a doctor
b) sedatives available on prescription without the doctor's knowledge
c) over-the-counter sedatives
d) inhalant drugs/spliff
e) drugs by injection
f) the so-called "legal highs"

13. Doctor who took care of you during your pregnancy:

a) Have not talked with you about drinking alcohol during pregnancy.
b) Encouraged to restrain completely from drinking alcohol during pregnancy.
c) Allowed to drink small amounts of alcohol during pregnancy.
d) Encouraged to drink small amounts of alcohol during pregnancy.

14. What is, in your opinion, daily intake of alcohol which is safe in pregnancy?

a) There is no safe amount of alcohol during pregnancy.
b) I do not know.
c) One glass of red wine.
d) One pint of beer.
e) One glass of cognac.

15. Were there/are there any strong sources of electromagnetic radiation, such as:

mobile phone transmitting antennas or high voltage transmission lines in your work environment or in your house surroundings?

a) Yes
b) No
c) I do not know

16. Have you had an X-RAY examination during this pregnancy?

a) yes
b) no

17. Have you drunk caffeinated drinks during this pregnancy? For example: coffee, strong black or green tea, energy drinks, Coca-cola.

a) yes
b) no

18. Have you smoked cigarettes during this pregnancy?

a) I have never smoked (continue to question 20.).
b) I have stopped before pregnancy or earlier.
c) I have stopped during this pregnancy.
d) I still smoke.

19. How many cigarettes have you smoked daily:

a) In the last 3 months before pregnancy? ………
b) In the first 3 months of pregnancy? ………
c) In the last 3 months of pregnancy? ………
d) I still smoke around ……… cigarettes daily.

20. How often have you consumed beverages containing alcohol before pregnancy?

a) Never
b) 1 per month or less
c) 2 to 4 times per month
d) 2 or 3 times per week
e) 4 times per week or more

21. How often did you consume beverages containing alcohol during pregnancy?

a) Never (continue to question 25.)
b) 1 per month or less
c) 2 to 4 times per month
d) 2 or 3 times per week
e) 4 times per week or more

22. What kind of alcohol beverages did you consume during pregnancy? You can choose more than 1 answer.

a) wine
b) beer
c) high-proof alcohol

23. In which trimester did you consume alcohol beverages during pregnancy? You can choose more than 1 answer.

a) 1st trimester (1st - 3rd month)
b) 2nd trimester (4th - 6th month)
c) 3rd trimester (7th - 9th month)

24. What was the greatest amount of alcohol you drank at once during pregnancy?

a) 1 small beer, or 1 glass of wine, or 1 glass of vodka, or 1 cocktail
b) 2 small beers, or 2 glasses of wine, or 2 glasses of vodka, or 2 cocktails
c) 3 small beers, or 3 glasses of wine, or 3 glasses of vodka, or 3 cocktails

25. Can you rate your knowledge on positive and negative factors affecting the course of pregnancy?

a) High
b) Moderate
c) Low
d) No knowledge

26. Where did you look for information on substances toxic to the fetus? You can choose more than 1 answer.

a) internet
b) media (press, radio, TV)
c) doctor
d) midwife
e) birthing classes
f) family
g) friends
h) other.

MATERNAL CHARACTERISTICS

1. How old are you?

a) less than 18 years old
b) 18-24 years old
c) 25-30 years old
d) 31-35 years old
e) more than 35 years old

2. What is your education?

a) Primary school
b) Middle school
c) Basic vocational education
d) Secondary vocational education
e) High school diploma/general secondary schools
f) Bachelor degree/Engineer
g) Master's degree

3. What is your marital status?

a) Single
b) Married
c) Informal relationship
d) Separated
e) Divorced
f) Widow

4. Where do you live?

a) Countryside
b) City < 100.000 inhabitants
c) City >100.000 and <100.000 inhabitants
d) City > 400.000 inhabitants

5. Do you work? What is the type of your profession?

a) Office/administrative work.
b) Physical work.
c) I am a business owner.
d) I do not work.
e) I am a student.

6. Can you rate your social and living conditions?

a) Very good
b) Good

c) Middle
d) Low

7. Who lives with you at one household? You can choose more than 1 answer.

a) Husband
b) Partner
c) Child/children
d) Parents
e) Siblings
f) Other people, who are they? ………

Thank you very much for your time and for participating in the study.
