# Supplementary material for: Estimation of Prenatal Alcohol Exposure: Comparison of Retrospective Survey and Measurement of Fatty Acid Ethyl Esters, Ethyl Sulfate, and Ethyl Glucuronide Concentrations in Neonatal Meconium
Source: Toxics. 2026 Feb 4;14(2):155. doi: 10.3390/toxics14020155 (PMC12944540; doi:10.3390/toxics14020155)
Supplement: Supplementary file 1 [file toxics-14-00155-s001.zip › Table S02 answers 1-5.pdf]

Table S2. Results of survey questions 1 to 5 (n=478) in pregnant women conducted at the Neonatology Clinic of the Medical University of Gdańsk in the Pomeranian Province between June 16, 2019, and April 24, 2020.

**No**	**Date of birth** (DD-MM-YYYY)	**Child's gender** (f-female, m-male)	**Mother's age** (years)	**Week of delivery** (number)	**Answer 1** (a-e)	**Answer 2** (a-e)	**Answer 3** (a-c)	**Answer 4a** (grams)	**Answer 4b** (cm)	**Answer 4c** (cm)	**Answer 5** (Apgar)
1	n/d	n/d	33	38	a	a	a	3000	n/d	53	a
2	16-06-2019	f	31	34	a	a	b	2200	31	51	b
3	16-06-2019	f	26	39	a	a	a	3184	36	53	a
4	16-06-2019	m	34	40	c	a	a	3035	33	52	a
5	16-06-2019	f	36	36	e	e	b	3240	35	52	a
6	17-06-2019	f	30	37	b	b	a	3025	32	49	a
7	17-06-2019	m	31	39	b	b	a	3670	37,5	55	a
8	17-06-2019	f	35	38	a	a	a	2850	37,5	50	a
9	17-06-2019	f	31	41	b	b	a	4330	35	60	a
10	18-06-2019	f	45	38	e	e	a	2935	32	54	a
11	18-06-2019	m	33	39	a	a	a	3000		53	a
12	18-06-2019	f	34	39	b	b	a	3900	36	55	a
13	20-06-2019	m	34	40	b	b	a	3855	35	55	a
14	21-06-2019	m	30	40	a	a	a	4500	36	63	a
15	22-06-2019	f	36	39	c	c	a	3590		57	b

No	Date of birth (DD-MM-YYYY)	Child's gender (f-female, m-male)	Mother's age (years)	Week of delivery (number)	Answer 1 (a-e)	Answer 2 (a-e)	Answer 3 (a-c)	Answer 4a (grams)	Answer 4b (cm)	Answer 4c (cm)	Answer 5 (Apgar)
16	23-06-2019	f	32	39	a	a	a	3600	35	56	a
17	23-06-2019	f	35	41	b	a	a	3570	34	57	a
18	23-06-2019	m	30	39	a	a	a	3460	34	55	a
19	24-06-2019	f	26	39	c	c	a	3570	34	58	a
20	24-06-2019	m	26	39	b	b	a	3570	35	54	a
21	24-06-2019	m	21	36	a	a	b	2560	33	50	a
22	24-06-2019	m	40	41	c	a	a	3720	32,5	59	a
23	25-06-2019	m	30	41	a	a	a	3325	34	53	a
24	25-06-2019	f	34	40	c	c	a	2750	32	50	a
25	25-06-2019	f	25	41	a	a	a	3500	35	56	a
26	25-06-2019	f	20	40	b	b	a	3345		57	a
27	25-06-2019	f	39	38	e	d	a	2200	33	48	b
28	27-06-2019	m	39	28	c	c	b	1160	27	40	c
29	27-06-2019	f	32	40	c	b	a	3495	34	59	b
30	27-06-2019	f	29	40	d	d	a	3550	34,5	55	a
31	09-07-2019	f	34	38	d	d	a	2845		51	a
32	17-07-2019	f	27	41	a	a	a	3405	35	52	a

No	Date of birth (DD-MM-YYYY)	Child's gender (f-female, m-male)	Mother's age (years)	Week of delivery (number)	Answer 1 (a-e)	Answer 2 (a-e)	Answer 3 (a-c)	Answer 4a (grams)	Answer 4b (cm)	Answer 4c (cm)	Answer 5 (Apgar)
33	18-07-2019	f	29	41	a	a	a	3620	33	55	a
34	18-07-2019	m	26	37	a	a	a	3220		50	a
35	18-07-2019	f	30	36	b	b	b	2745		53	a
36	18-07-2019	f	30	36	b	b	b	2570		54	a
37	20-07-2019	f	28		a	a	a	3160	33,5	52	a
38	01-08-2019	f	35	33	c	b	b	1860	32	48	a
39	02-08-2019	f	31	39	b	b	a	3390	32	55	a
40	04-08-2019	m	38	42	b	a	a	3790	35	53	a
41	05-08-2019	f	34	39	b	b	a	2910	34,5	49	a
42	07-08-2019	m	36	38	b	b	a	2975	32	55	a
43	08-08-2019	f	30	40	c	c	a	3660	34	56	a
44	08-08-2019	f	24	39	a	a	a	2440	30	51	a
45	08-08-2019	f	35	39	b	a	a	3545	36	54	a
46	08-08-2019	m	25	40	a	a	a	3880	33	61	a
47	08-08-2019	m	37	42	c	b	a	3665	35	58	a
48	09-08-2019	f	36	35	b	b	b	2480	33	51	a
49	09-08-2019	f	18	40	a	a	a	3300	34	55	a

No	Date of birth (DD-MM-YYYY)	Child's gender (f-female, m-male)	Mother's age (years)	Week of delivery (number)	Answer 1 (a-e)	Answer 2 (a-e)	Answer 3 (a-c)	Answer 4a (grams)	Answer 4b (cm)	Answer 4c (cm)	Answer 5 (Apgar)
50	09-08-2019	m	26	40	a	a	a	3720	35	54	a
51	09-08-2019	f	29	42	a	a	a	3380	35	57	a
52	09-08-2019	f	26	42	a	a	a	3030	32	53	a
53	09-08-2019	f	34	40	c	c	a	3555	34	55	a
54	09-08-2019	m	33	40	b	b	a	4300	35	61	a
55	20-06-2019	m	41	37	c	c	b	3150	34	56	a
56	09-08-2019	f	42	40	a	a	a	3250	36	57	a
57	11-08-2019	f	28	41	b	b	a	3795	34	54	a
58	12-08-2019	f	33	40	b	b	a	3340	31	54	a
59	12-08-2019	m	33	41	b	b	a	3635	36	53	a
60	12-08-2019	m	26	40	b	b	a	4175	36	59	a
61	12-08-2019	f	26	40	a	a	a	3330	33	52	a
62	12-08-2019	m	30	39	b	b	a	4485	35	58	c
63	13-08-2019	m	32	39	b	b	a	4300	34	57	a
64	13-08-2019	f	38	39	c	c	a	3055	32	52	a
65	13-08-2019	m	39	37	c	b	b	3125	35	50	a
66	13-08-2019	m	32	38	a	a	a	3650	36	54	a

No	Date of birth (DD-MM-YYYY)	Child's gender (f-female, m-male)	Mother's age (years)	Week of delivery (number)	Answer 1 (a-e)	Answer 2 (a-e)	Answer 3 (a-c)	Answer 4a (grams)	Answer 4b (cm)	Answer 4c (cm)	Answer 5 (Apgar)
67	13-08-2019	m	29	37	b	a	a	2780	33	51	a
68	14-08-2019	m	31	40	b	b	a	3950	35	54	a
69	14-08-2019	f	29	41	a	a	a	4100		57	a
70	14-08-2019	f	33	38	b	b	a	2050	30	48	a
71	14-08-2019	m	34	41	b	b	a	3600		55	a
72	14-08-2019	f	33	40	b	b	a	3585	34	56	a
73	14-08-2019	m	40	38	d	c	a	3260		56	a
74	14-08-2014	m	27	33	a	a	b	1740	30	45	a
75	14-08-2014	f	27	33	a	a	b	1430	29	43	a
76	19-08-2019	f	24	35	c	b	b	2630	33	52	a
77	19-08-2019	m	23	41	a	a	a	3250	33	52	a
78	20-08-2019	m	28	40	a	a	a	3130	33	50	a
79	20-08-2019	f	36	39	b	b	a	3125	33	53	a
80	20-08-2019	m	27	40	b	b	a	3430	34	56	a
81	20-08-2019	f	28	41	b	b	a	3520	34	52	a
82	20-08-2019	f	34	38	c	c	a	3220	34	54	a
83	20-08-2019	m	27	39	a	a	a	3850	36	56	a

No	Date of birth (DD-MM-YYYY)	Child's gender (f-female, m-male)	Mother's age (years)	Week of delivery (number)	Answer 1 (a-e)	Answer 2 (a-e)	Answer 3 (a-c)	Answer 4a (grams)	Answer 4b (cm)	Answer 4c (cm)	Answer 5 (Apgar)
84	20-08-2019	f	27	40	c	c	a	3220	34	53	a
85	20-08-2019	m	35	41	c	c	a	4250	36	62	a
86	20-08-2019	m	32	41	b	b	a	3985	35	57	a
87	20-08-2019	f	24	41	a	a	a	3805	34	60	a
88	21-08-2019	f	37	41	a	a	a	3090	33	54	a
89	21-08-2014	m	22	36	a	a	36	2970	34	51	a
90	21-08-2019	m	35	27	b	b	b	970		35	b
91	22-08-2019	f	29	40	c	b	a	3275	32	50	a
92	01-09-2019	m	32	34	b	a	b	2640	33	52	a
93	02-09-2019	m	31	38	a	a	a	3295	32	53	a
94	02-09-2019	f	27	34	d	d	b	1470	29	41	
95	02-09-2019	m	27	37	a	a	b	3305	31	55	a
96	02-09-2019	f	38	38	c	c	b	2950	33	53	a
97	02-09-2019	m	37	37	d	d	a	4740	36	56	a
98	02-09-2019	f	24	40	d	b	a	3365	33	52	a
99	02-09-2019	m	29	38	a	a	a	2450	31	50	a
100	02-09-2019	f	26	38	a	a	a	2625	31	51	a

No	Date of birth (DD-MM-YYYY)	Child's gender (f-female, m-male)	Mother's age (years)	Week of delivery (number)	Answer 1 (a-e)	Answer 2 (a-e)	Answer 3 (a-c)	Answer 4a (grams)	Answer 4b (cm)	Answer 4c (cm)	Answer 5 (Apgar)
101	03-09-2019	m	28	40	a	a	a	3950	34	59	a
102	03-09-2019	m	30	41	b	b	a	3610	36	53	a
103	18-09-2019	f	27	40	a	a	a	3020	34	57	a
104	18-09-2019	m	28	41	c	b	a	3710	33	56	a
105	18-09-2019	f	31	41	a	a	a	2700	31	53	a
106	18-09-2019	m	39	40	b	b	a	3170	35,5	52	a
107	18-09-2019	m	23	40	b	a	a	3990	34	56	a
108	18-09-2019	f	29	40	c	b	a	3700	33	54	a
109	19-09-2019	m	23	38	a	a	38	3100	32	51	a
110	19-09-2019	m	31	39	a	a	a	2930	31	58	a
111	19-09-2019	m	35	39	d	b	a	4510		62	a
112	19-09-2019	f	39	36	b	b	b	2820	35	50	a
113	19-09-2019	f	34	38	b	b	38	3425		56	a
114	20-09-2019	m	37	38	b	a	38	3300	33	56	a
115	20-09-2019	f	30	39	b	b	39	3120	35	53	a
116	20-09-2019	m	26	39	a	a	39	3700	36	55	a
117	21-09-2019	m	35	37	a	a	37	2930	33	51	a

No	Date of birth (DD-MM-YYYY)	Child's gender (f-female, m-male)	Mother's age (years)	Week of delivery (number)	Answer 1 (a-e)	Answer 2 (a-e)	Answer 3 (a-c)	Answer 4a (grams)	Answer 4b (cm)	Answer 4c (cm)	Answer 5 (Apgar)
118	24-09-2019	m	31	40	c	c	40	3180	34	54	a
119	24-09-2019	m	25	41	b	b	41	3815	33	55	a
120	24-09-2019	m	33	39	a	a	39	3460		50	a
121	24-09-2019	m	38	38	a	a	38	3655	36	55	a
122	24-09-2016	f	33	38	c	c	a	3340		57	a
123	25-09-2019	m	28	36	a	a	b	3020	36	53	a
124	25-09-2019	f	28	40	a	a	a	2865	33	56	a
125	25-09-2019	m	24	39	a	a	39	3450		53	a
126	25-09-2019	f	29	40	a	a	a	3310		55	a
127	26-09-2019	f	33	37	b	b	a	2825	32	47	a
128	29-09-2019	f	28	37	b	b	a	3430	35	54	a
129	30-09-2019	m	28	40	a	a	a	3510	32	56	a
130	30-09-2019	f	21	39	b	b	a	2590	31	51	a
131	30-09-2019	m	35	39	a	a	39	2800		52	a
132	30-09-2019	m	29	39	b	b	a	3505		56	a
133	01-10-2019	f	20	36	a	a	b	2390		53	a
134	01-10-2019	f	36	34	b	b	34	3160	35	53	a

No	Date of birth (DD-MM-YYYY)	Child's gender (f-female, m-male)	Mother's age (years)	Week of delivery (number)	Answer 1 (a-e)	Answer 2 (a-e)	Answer 3 (a-c)	Answer 4a (grams)	Answer 4b (cm)	Answer 4c (cm)	Answer 5 (Apgar)
135	01-10-2019	m	28	39	b	b	a	3770	35	60	a
136	01-10-2019	f	40	39	a	a	39	4190		58	a
137	01-10-2019	m	35	40	a	a	a	3585	33	54	a
138	01-10-2019	m	28	41	a	a	a	3450	34	55	a
139	01-10-2019	f	28	40	a	a	a	3450	32	57	a
140	02-10-2019	f	42	40	c	c	40	3770		60	a
141	02-10-2019	f	18	33	a	a	b	2115	28	44	a
142	02-10-2019	f	34	38	c	b	a	2840	32	52	a
143	02-10-2019	m	38	40	c	c	a	4025	35	61	a
144	02-10-2019	f	37	38	a	a	a	2185	32	49	a
145	02-10-2019	m	40	39	b	b	a	3870	33	56	a
146	02-10-2019	f	33	40	a	a	a	3185	33	58	a
147	02-10-2019	m	34	39	a	a	a	3735	36	57	a
148	03-10-2019	f	32	40	a	a	a	3825	35	58	a
149	03-10-2019	f	38	39	c	c	a	3185	31	54	a
150	03-10-2019	f	24	39	c	b	a	3065	35	54	a
151	03-10-2019	f	30	38	a	a	a	3850	34	52	a

No	Date of birth (DD-MM-YYYY)	Child's gender (f-female, m-male)	Mother's age (years)	Week of delivery (number)	Answer 1 (a-e)	Answer 2 (a-e)	Answer 3 (a-c)	Answer 4a (grams)	Answer 4b (cm)	Answer 4c (cm)	Answer 5 (Apgar)
152	03-10-2019	m	28	38	b	b	38	3010	34	58	a
153	03-10-2019	m	27	36	a	a	36	2595	33	48	a
154	03-10-2019	m	30								
155	04-10-2019	m	38	38	d	b	38	3475	35	56	a
156	04-10-2019	m	32	40	a	a	40	3485	33	53	a
157	04-10-2019	f	33	38	b	b	38	2980	33	52	a
158	04-10-2019	m	27	39	a	a	39	3850	34	55	a
159	04-10-2019	m	22	38	b	a	38	3400	34	54	a
160	04-10-2019	m	30	40	b	b	40	3285	35	54	a
161	04-10-2019	f	26	38	a	a	38	2370	30	50	b
162	05-10-2019	m	**37**	**34**	**b**	**b**	**34**	**2055**	**33**	**45**	**a**
163	07-10-2019	m	35	37	c	b	37	2330	34	48	a
164	05-10-2019	f	31	40	c	c	40	3180	52	54	a
165	07-10-2019	m	35	37	c	b	37	2330	34	48	a
166	07-10-2019	f	33	41	c	b	41	3900		55	a
167	08-10-2019	f	28	39	b	b	39	3540	34	57	a
168	08-10-2019	f	29	41	a	a	a	3350	34	50	a

No	Date of birth (DD-MM-YYYY)	Child's gender (f-female, m-male)	Mother's age (years)	Week of delivery (number)	Answer 1 (a-e)	Answer 2 (a-e)	Answer 3 (a-c)	Answer 4a (grams)	Answer 4b (cm)	Answer 4c (cm)	Answer 5 (Apgar)
169	08-10-2019	f	26	40	a	a	40	4405	35	61	a
170	08-10-2019	m	30	38	e	e	a	2400		51	b
171	08-10-2019	m	30	38	e	e	a	2470		49	a
172	08-10-2019	f	27	39	a	a	a	3200	34	54	a
173	08-10-2019	f	38	38	d	c	a	2800	34	53	a
174	08-10-2019	m	27	38	a	a	a	3105	32	53	a
175	08-10-2019	f	32	38	a	a	38	3430	33	52	a
176	09-10-2019	m	29	40	c	b	40	4650		60	a
177	09-10-2019	m	33	35	a	a	b	2450	32	50	b
178	09-10-2019	m	26	36	b	b	b	3250		51	a
179	09-10-2019	m	37	38	b	b	38	4030	38	57	a
180	10-10-2019	m	20	39	a	a	a	3070		53	a
181	10-10-2019	m	26	38	a	a	38	3280	34	53	a
182	10-10-2019	m	30	39	b	b	39	3495	37	55	a
183	10-10-2019	m	24	39	a	a	39	4345	38	59	a
184	10-10-2019	m	34	37	b	b	37	3110	32	51	a
185	10-10-2019	m	34	37	b	b	37	2590	33	46	a

**No**	**Date of birth** (DD-MM-YYYY)	**Child's gender** (f-female, m-male)	**Mother's age** (years)	**Week of delivery** (number)	**Answer 1** (a-e)	**Answer 2** (a-e)	**Answer 3** (a-c)	**Answer 4a** (grams)	**Answer 4b** (cm)	**Answer 4c** (cm)	**Answer 5** (Apgar)
186	10-10-2019	f	32	41	a	a	41	3300	32	57	a
187	10-10-2019	m	31	38	d	c	a	3090	33	55	a
188	10-10-2019	f	29	39	a	a	a	2890	34	55	a
189	10-10-2019	f	35	37	d	d	37	3525	33	57	a
190	11-10-2019	f	31	39	d	d	39	3600	35	56	a
191	11-10-2019	m	33	40	d	d	40	4990	37	62	a
192	11-10-2019	f	28	38	b	b	38	2930	32	55	a
193	11-10-2019	f	29	41	a	a	41	4140		59	a
194	13-10-2019	f	26	39	a	a	a	4225	34	60	a
195	14-10-2019	m	33	40	b	b	40	4030	35	59	a
196	14-10-2019	m	22	39	a	a	a	3770	34	56	a
197	15-10-2019	f	34	39	b	b	39	3700	34	54	a
198	15-10-2019	m	34	38	a	a	38	3695	33	55	a
199	15-10-2019	m	28	41	a	a	41	4030	33	58	a
200	15-10-2019	m	34	38	b	a	a	3080	33	53	a
201	15-10-2019	m	25	39	a	a	a	3847		57	a
202	15-10-2020	m	27	38	a	a	38	3250	35	51	a

No	Date of birth (DD-MM-YYYY)	Child's gender (f-female, m-male)	Mother's age (years)	Week of delivery (number)	Answer 1 (a-e)	Answer 2 (a-e)	Answer 3 (a-c)	Answer 4a (grams)	Answer 4b (cm)	Answer 4c (cm)	Answer 5 (Apgar)
203	15-10-2021	m	39	37	a	a	37	2305		50	a
204	15-10-2022	f	31	36	c	b	36	2820		51	a
205	15-10-2023	m	31	36	c	b	36	3080		53	a
206	15-10-2024	m	30	40	a	a	40	3645	35	56	a
207	15-10-2025	m	27	36	b	b	36	3300		57	a
208	16-10-2019	f	33	41	b	c	a	3605		56	a
209	16-10-2019	f	30	39	b	b	a	3080	32	54	a
210	17-10-2019	f	35	40	d	b	a	4040	36	55	a
211	17-10-2019	m	29	39	a	a	a	3530		52	a
212	18-10-2019	m	31	37	b	b	37	3400	33	56	a
213	18-10-2019	m	35	39	b	b	a	4250	35	58	a
214	18-10-2019	m	27	40	b	a	a	3480		52	a
215	18-10-2019	f	36	40	a	a	a	3480	33	54	a
216	18-10-2019	f	24	40	b	b	40	3250	31	54	a
217	18-10-2019	m	34	35	b	a	b	2795	35	53	a
218	19-10-2019	m	37	39	c	c	a	3050	34	52	a
219	19-10-2019	f	39	40	b	b	a	3185	33	55	a

No	Date of birth (DD-MM-YYYY)	Child's gender (f-female, m-male)	Mother's age (years)	Week of delivery (number)	Answer 1 (a-e)	Answer 2 (a-e)	Answer 3 (a-c)	Answer 4a (grams)	Answer 4b (cm)	Answer 4c (cm)	Answer 5 (Apgar)
220	19-10-2019	f	32	39	d	b	a	3400		55	a
221	19-10-2019	f	34	35	c	c	b	2010	31	46	b
222	21-10-2019	m	28	39	b	a	a	3900	34	58	a
223	21-10-2019	f	32	39	b	b	39	3155	34	57	a
224	21-10-2019	m	34	39	a	a	a	3705			a
225	21-10-2019	f	28	39	a	a	a	2590	29	50	a
226	21-10-2019	m	35	39	b	b	a	4535		52	a
227	22-10-2019	f	37	38	a	a	a	3325		54	a
228	23-10-2019	f	28	40	a	a	a	3420	34	56	a
229	23-10-2019	f	29	41	a	a	a	3450	32	53	a
230	23-10-2019	f	30	39	b	b	a	3650	38	55	a
231	24-10-2019	f	31	36	a	a	b	2280	29	48	b
232	24-10-2019	f	22	40	a	a	a	3600	34	56	a
233	24-10-2019	f	35	40	b	b	a	4325	35	56	a
234	24-10-2019	m	16	41	a	a	a	2735	33	55	a
235	24-10-2019	f	30	39	a	a	a	3540	33	57	a
236	24-10-2019	f	35	39	c	c	a	2725		49	a

No	Date of birth (DD-MM-YYYY)	Child's gender (f-female, m-male)	Mother's age (years)	Week of delivery (number)	Answer 1 (a-e)	Answer 2 (a-e)	Answer 3 (a-c)	Answer 4a (grams)	Answer 4b (cm)	Answer 4c (cm)	Answer 5 (Apgar)
237	24-10-2019	f	43	40	b	b	a	3380	32	51	a
238	24-10-2019	m	31	39	a	a	a	3550	34	55	a
239	25-10-2019	f	21	39	a	a	a	2730	32	51	a
240	25-10-2019	m	35	34	a	a	b	2490	31	50	a
241	26-10-2019	m	39	41	e	d	a	3950	34	57	a
242	26-10-2019	m	28	40	a	a	a	3185	33	55	a
243	26-10-2019	f	29	39	a	a	a	2995	33	51	a
244	26-10-2019	f	26	42	a	a	a	3695	34	56	a
245	27-10-2019	m	31	41	a	a	a	2780	33	53	a
246	27-10-2019	f	32	40	a	a	a	2865	31	52	a
247	27-10-2019	m	36	36	c	b	b	3200	36	55	a
248	27-10-2019	m	20	39	a	a	a	3450	34	53	a
249	27-10-2019	f	28	39	a	a	a	3555	33	55	a
250	28-10-2019	m	24	38	e	b	a	4110	34	57	a
251	28-10-2019	m	33	40	b	b	a	3055	33	54	a
252	29-10-2019	m	35	38	d	e	a	3225	33	55	a
253	29-10-2019	f	32	38	c	c	a	2860	34	52	a

No	Date of birth (DD-MM-YYYY)	Child's gender (f-female, m-male)	Mother's age (years)	Week of delivery (number)	Answer 1 (a-e)	Answer 2 (a-e)	Answer 3 (a-c)	Answer 4a (grams)	Answer 4b (cm)	Answer 4c (cm)	Answer 5 (Apgar)
254	29-10-2019	m	38	39	b	b	a	3350	34	52	a
255	29-10-2019	m	23	42	a	a	a	3475	34	56	a
256	29-10-2019	m	39	38	d	d	a	2710		50	a
257	29-10-2020	f	37	40	a	a	a	4240	35	59	a
258	30-10-2019	m	21	38	a	a	a	3255		55	a
259	30-10-2019	f	36	40	b	b	a	5050	37	64	a
260	30-10-2019	f	33	39	b	b	a	3225	34	56	a
261	30-10-2019	f	27	38	a	a	a	1865	31	46	a
262	04-08-2019	m	42	32	c	c	b	2290	33	54	a
263	31-10-2019	m	33	34	c	b	b	2055	30	46	b
264	31-10-2019	m	33	34	c	b	b	1675	30	47	a
265	01-11-2019	m	30	42	a	a	a	4120	34	55	a
266	01-12-2019	f	31	39	b	b	a	3680	32	54	b
267	01-11-2019	m	33	41	a	a	a	3895	33	49	a
268	01-11-2019	m	37	39	a	a	a	3230	35	51	a
269	01-11-2019	m	37	39	a	a	a	2810	33,5	51	a
270	02-11-2019	f	36	38	c	b	a	3255	34	54	a

No	Date of birth (DD-MM-YYYY)	Child's gender (f-female, m-male)	Mother's age (years)	Week of delivery (number)	Answer 1 (a-e)	Answer 2 (a-e)	Answer 3 (a-c)	Answer 4a (grams)	Answer 4b (cm)	Answer 4c (cm)	Answer 5 (Apgar)
271	02-11-2019	f	39	40	a	a	a	3725	33	57	a
272	02-11-2019	f	34	42	b	a	a	3910	34	56	a
273	03-11-2019	f	35	31	a	a	b	1545	29	44	a
274	04-11-2019	f	31	37	a	a	a	2825		52	a
275	04-11-2019	f	37	34	a	a	b	2575	34	52	b
276	05-11-2019	m	34	40	b	b	a	3350	32	55	a
277	05-11-2019	f	38	40	e	d	a	3720	34	55	a
278	05-11-2019	m	36	40	c	c	a	3465	35	54	a
279	05-11-2019	f	35	40	c	c	a	3310	34	55	a
280	05-11-2019	f	37	39	c	c	a	3455		56	a
281	05-11-2019	m	28	40	a	a	a	3135	32	52	a
282	05-11-2019	f	33	37	a	a	a	2105	33	43	a
283	05-11-2019	m	24	37	b	b	a	3445	34	54	a
284	06-11-2019	m	34	40	b	b	a	3350	33	53	a
285	06-11-2019	m	30	41	a	a	a	4000	34	57	a
286	07-11-2019	m	28	40	a	a	a	3035	34	52	a
287	09-11-2019	f	26	36	b	b	b	2115		46	a

**No**	**Date of birth** (DD-MM-YYYY)	**Child's gender** (f-female, m-male)	**Mother's age** (years)	**Week of delivery** (number)	**Answer 1** (a-e)	**Answer 2** (a-e)	**Answer 3** (a-c)	**Answer 4a** (grams)	**Answer 4b** (cm)	**Answer 4c** (cm)	**Answer 5** (Apgar)
288	09-11-2019	f	26	36	b	b	b	2000		46	a
289	09-11-2019	f	30	41	b	b	a	3460	32	56	a
290	09-11-2019	m	30	39	a	a	a	3570	35	52	a
291	09-11-2019	m	36	41	a	a	a	3320	36	51	a
292	12-11-2019	m	35	40	e	c	a	4760	37	60	a
293	12-11-2019	f	31	42	b	b	a	3110	33	48	a
294	13-11-2019	m	29	36	a	a	b	2935	32	52	a
295	14-11-2019	m	29	38	a	a	b	2660	33	53	a
296	14-11-2019	f	30	39	c	b	a	2920	33	55	b
297	14-11-2019	f	28	37	a	a	a	3365	33	55	a
298	14-11-2019	f	31	39	d	b	a	3050	34	54	a
299	14-11-2019	m	35	39	b	b	a	4250		59	a
300	18-11-2019	m	19	41	a	a	a	3620	34	58	a
301	18-11-2019	m	33	40	b	b	a	4035	34	55	a
302	19-11-2019	f	31	36	e	c	b	2600		48	a
303	19-11-2019	f	26	37	a	a	a	2705	34	49	a
304	19-11-2019	f	26	37	a	a	a	2380	34	50	a

No	Date of birth (DD-MM-YYYY)	Child's gender (f-female, m-male)	Mother's age (years)	Week of delivery (number)	Answer 1 (a-e)	Answer 2 (a-e)	Answer 3 (a-c)	Answer 4a (grams)	Answer 4b (cm)	Answer 4c (cm)	Answer 5 (Apgar)
305	19-11-2019	m	25	38	a	a	a	3230	33	51	a
306	19-11-2019	f	27	41	b	b	a	3475	32	58	a
307	20-11-2019	m	29	41	b	b	a	3830	33	59	a
308	21-11-2019	m	38	40	b	b	a	3235	34	54	a
309	21-11-2019	m	39	30	b	a	b	1675		48	a
310	21-11-2019	m	28	36	a	a	b	3150	30	54	a
311	21-11-2019	m	30	39	b	b	a	3800		55	a
312	21-11-2019	f	25	41	a	a	a	4070		55	a
313	23-11-2019	f	29	40	a	a	a	4040	36	57	a
314	23-11-2019	m	36	42	c	b	a	3800	34	58	a
315	24-11-2019	m	39	37	a	a	a	2860		52	a
316	24-11-2019	f	37	40	a	a	a	2925	34	55	b
317	24-11-2019	m	36	37	c	b	a	3390		57	a
318	25-11-2019	m	33	34	c	c	b	2880			a
319	25-11-2019	m	23	31			b	1680	29	43	b
320	25-11-2019	m	23	31			b	1770	29	45	b
321	26-11-2019	f	36	37	e	e	a	2750	34	52	a

No	Date of birth (DD-MM-YYYY)	Child's gender (f-female, m-male)	Mother's age (years)	Week of delivery (number)	Answer 1 (a-e)	Answer 2 (a-e)	Answer 3 (a-c)	Answer 4a (grams)	Answer 4b (cm)	Answer 4c (cm)	Answer 5 (Apgar)
322	26-11-2019	m	28	41	a	a	a	3845	35	59	a
323	26-11-2019	f	34	38	b	b	a	3170	33	56	a
324	26-11-2019	f	28	40	a	a	a	3300	35	55	a
325	27-11-2019	m	30	40	d	b	a	3640	36	57	a
326	27-11-2019	m	29	39	d	d	a	2735	33	51	a
327	28-11-2019	m	28	39	a	a	a	3570	38	57	a
328	28-11-2019	f	34	38	a	a	a	3170	33	50	a
329	02-12-2019	f	32	40	a	a	a	4325		62	a
330	03-12-2019	m	36	40	b	b	a	3530	36	58	a
331	03-12-2019	f	28	39	a	a	a	3235	33	54	a
332	05-12-2019	f	28	37	b	b	a	3345		55	a
333	05-12-2019	f	32	39	b	b	a	3325	35	56	a
334	05-12-2019	m	43	34	e	d	b	2780	35	54	a
335	05-12-2019	f	45	38	d	d	a	3180	34	53	a
336	06-12-2019	m	31	41	b	b	a	3430	35	52	a
337	06-12-2019	f	34	39	a	a	a	2925	30	27	a
338	10-12-2019	m	23	39	b	b	a	3150	34	53	a

No	Date of birth (DD-MM-YYYY)	Child's gender (f-female, m-male)	Mother's age (years)	Week of delivery (number)	Answer 1 (a-e)	Answer 2 (a-e)	Answer 3 (a-c)	Answer 4a (grams)	Answer 4b (cm)	Answer 4c (cm)	Answer 5 (Apgar)
339	10-12-2019	m	37	41	b	b	a	3660	35	53	a
340	10-12-2019	m	28	39	a	a	a	4000	36	55	a
341	10-12-2019	m	28	39	a	a	a	2975	32	54	a
342	11-12-2019	m	23	40	b	a	a	3055	34	54	a
343	11-12-2019	f	29	38	b	a	a	2415	30	48	a
344	11-12-2019	f	21	40	b	b	a	2570	32	52	a
345	13-12-2019	m	27	42	b	b	a	3740	33	58	a
346	13-12-2019	f	32	39	c	b	a	3250	33	51	a
347	20-12-2019	m	42	34	a	a	b	2770		48	a
348	27-12-2019	f	31	40	b	b	a	3590	35	49	a
349	27-12-2019	f	38	38	e	c	a	3660	35	51	a
350		n/d	27	38	a	a	38	2500		52	a
351	03-01-2020	f	34	40	a	a	a	3855		55	a
352	07-01-2020	f	28	40	c	a	a	3425	34	52	a
353	20-01-2020	f	37	39	b	b	a	3640	35	53	a
354	20-01-2020	f	31	41	a	a	a	3715	33	60	a
355	20-01-2020	f	34	39	e	e	a	3745	37	53	a

No	Date of birth (DD-MM-YYYY)	Child's gender (f-female, m-male)	Mother's age (years)	Week of delivery (number)	Answer 1 (a-e)	Answer 2 (a-e)	Answer 3 (a-c)	Answer 4a (grams)	Answer 4b (cm)	Answer 4c (cm)	Answer 5 (Apgar)
356	20-01-2020	f	27	40	a	a	a	3580	34	53	a
357	28-01-2020	m	31	38	b	b	a	2070	35	46	a
358	28-01-2020	f	31	38	b	b	a	2785	38	52	a
359	28-01-2020	m	31	40	a	a	a	3300	33	56	a
360	28-01-2020	f	29	36	b	a	b	2485		51	a
361	11-02-2020	m	33	40	d	b	a	4385	38	56	a
362	11-02-2020	f	30	40	a	a	a	4015		55	a
363	11-02-2020	m	23	41	a	a	a	3390		55	a
364	11-02-2020	m	26	40	a	a	a	3855	34	56	a
365	11-02-2020	f	31	40	a	a	a	3435	32	53	a
366	13-02-2020	f	29	36	c	c	b	2980	35	53	a
367	13-02-2020	f	29	36	c	c	b	2460	33	52	a
368	13-02-2020	f	32	40	b	b	a	3475	36	56	a
369	13-02-2020	m	22	38	a	a	a	3120	35	55	a
370	14-02-2020	f	26	39	c	b	a	2650	32	48	a
371	14-02-2020	f	35	37	b	b	a	3600	36	56	a
372	14-02-2020	f	36	39	b	b	a	3600	34	52	a

No	Date of birth (DD-MM-YYYY)	Child's gender (f-female, m-male)	Mother's age (years)	Week of delivery (number)	Answer 1 (a-e)	Answer 2 (a-e)	Answer 3 (a-c)	Answer 4a (grams)	Answer 4b (cm)	Answer 4c (cm)	Answer 5 (Apgar)
373	14-02-2020	m	41	38	d	c	a	3160	37	51	a
374	15-02-2020	f	27	40	a	a	a	4015	37	54	a
375	15-02-2020	f	29	39	a	a	a	3515	33	56	a
376	16-02-2020	m	37	31	a	a	b	3160	38	51	a
377	17-02-2020	f	27	38	b	a	a	3210	32	50	a
378	17-02-2020	f	31	40	b	b	a	3670	33,5	53	a
379	17-02-2020	f	37	35	c	b	b	3035	35	51	a
380	18-02-2020	f	31	39	c	d	a	2930	32	52	a
381	18-02-2020	m	35	39	d	c	a	3045	35	56	a
382	18-02-2020	m	41	36	c	b	b	2810	33	54	a
383	18-02-2020	f	29	33	a	a	33	2015	31	49	b
384	19-02-2020	f	26	34	c	b	34	2300	32	51	b
385	20-20-2020	m	27	40	a	a	40	3025	35	54	a
386	20-20-2020	m	33	39	d	e	a	3530	35	54	a
387	20-02-2020	m	36	41	e	c	a	4135	37	56	a
388	20-20-2020	f	29	36	a	a	36	1760	30,5	45	a
389	20-02-2020	m	34	39	b	a	a	3700	34	55	a

No	Date of birth (DD-MM-YYYY)	Child's gender (f-female, m-male)	Mother's age (years)	Week of delivery (number)	Answer 1 (a-e)	Answer 2 (a-e)	Answer 3 (a-c)	Answer 4a (grams)	Answer 4b (cm)	Answer 4c (cm)	Answer 5 (Apgar)
390	23-02-2020	m	30	36	a	a	b	2740	31	51	a
391	23-02-2020	f	32	38	c	c	a	3145	33	54	a
392	23-02-2020	m	36	40	c	c	a	4090	36	55	a
393	23-02-2020	f	36	39	c	b	a	3200	34	52	a
394	23-02-2020	f	35	40	c	c	a	3350	32	54	a
395	23-02-2020	f	30	38	a	a	a	3865	36	60	a
396	23-02-2020	f	35	35	c	c	b	2555	32	48	b
397	24-02-2020	m	32	33	b	b	b	2670	31	47	a
398	25-02-2020	m	27	39	a	a	a	3780	34	54	a
399	25-02-2020	f	37	34	a	a	b	2260	32	47	a
400	25-02-2020	f	28	40	a	a	a	3550	36	54	a
401	25-02-2020	f	32	38	c	b	a	4415	33	61	a
402	25-02-2020	f	30	38	a	a	a	2720	30	47	a
403	25-02-2020	m	32	39	a	a	a	2820	31	51	a
404	28-02-2020	f	35	39	c	c	a	3570	35	54	a
405	28-02-2020	f	42	41	d	b	a	3570	33	59	a
406	28-02-2020	m	38	40	a	a	a	4540	35	59	a

No	Date of birth (DD-MM-YYYY)	Child's gender (f-female, m-male)	Mother's age (years)	Week of delivery (number)	Answer 1 (a-e)	Answer 2 (a-e)	Answer 3 (a-c)	Answer 4a (grams)	Answer 4b (cm)	Answer 4c (cm)	Answer 5 (Apgar)
407	28-02-2020	f	35	39	c	c	a	4155	35	56	a
408	28-02-2020	m	30	39	b	b	a	3640		53	a
409	28-02-2020	m	35	40	a	a	a	3390	34	57	b
410	29-02-2020	m	31	37	b	a	b	2800	32	51	a
411	29-02-2020	m	36	39	d	a	a	3540	35	54	a
412	02-03-2020	m	32	40	e	e	a	2900	32	50	a
413	02-03-2020	m	36	41	b	b	c	3490	33	59	a
414	02-03-2020	f	37	38	d	c	a	2420	32	50	a
415	02-03-2020	f	33	38	b	b	a	3660		54	a
416	02-03-2020	m	32	39	b	b	a	3430	33	53	a
417	02-03-2020	f	24	41	a	a	a	3510	34	58	a
418	02-03-2020	f	39	38	b	b	a	3015	31	50	a
419	02-03-2020	f	26	37	a	a	a	1745	30	42	a
420	02-03-2020	f	25	36	a	a	b	2330	32	50	a
421	02-03-2020	m	25	36		a	b	2550	34	49	a
422	04-03-2020	m	35	40	c	c	a	3500	34	57	a
423	04-03-2020	m	26	37	c	c	a	2500	34	48	a

No	Date of birth (DD-MM-YYYY)	Child's gender (f-female, m-male)	Mother's age (years)	Week of delivery (number)	Answer 1 (a-e)	Answer 2 (a-e)	Answer 3 (a-c)	Answer 4a (grams)	Answer 4b (cm)	Answer 4c (cm)	Answer 5 (Apgar)
424	04-03-2020	f	26	37	c	c	a	2690	32	49	a
425	05-03-2020	f	38	39	c	c	a	3545	36	57	a
426	05-03-2020	f	25	38	a	a	a	2415	32	52	a
427	06-03-2020	f	28	39	c	d	a	3645	33	53	a
428	07-03-2020	f	28	40	b	b	a	3390	32	55	a
429	07-03-2020	f	30	37	a	a	a	2370	32	49	a
430	07-03-2020	f	27	40	b	a	a	3655	35	57	a
431	07-03-2020	f	34	42	a	a	c	4835	39	58	a
432	08-03-2020	f	38	39	d	d	a	3915	33	56	a
433	08-03-2020	f	34	40	b	b	a	3385	32	56	a
434	08-03-2020	m	27	37	a	a	a	3020	34	54	b
435	08-03-2020	m	28	39	c	b	a	3910	34	56	a
436	09-03-2020	f	25	29	d	d	29	1400	26	43	b
437	10-03-2020	f	31	39	a	a	a	3640	32	57	a
438	10-03-2020	m	29	39	a	a	a	3995	35	55	a
439	11-03-2020	m	26	40	a	a	a	2995	32	55	a
440	11-03-2020	m	38	40	c	c	a	3200	34	55	a

No	Date of birth (DD-MM-YYYY)	Child's gender (f-female, m-male)	Mother's age (years)	Week of delivery (number)	Answer 1 (a-e)	Answer 2 (a-e)	Answer 3 (a-c)	Answer 4a (grams)	Answer 4b (cm)	Answer 4c (cm)	Answer 5 (Apgar)
441	12-03-2020	f	30		d	d	a	3570	34	56	a
442	12-03-2020	m	28	37	c	c	a	1810	32	46	a
443	12-03-2020	f	28	41	a	a	a	3460	35	56	a
444	12-03-2020	f	22	36	a	a	b	2760	33	50	a
445	12-03-2020	f	22	36	a	a	b	2715	33,5	50	a
446	17-03-2020	f	35	38	a	a	a	3680	36	58	a
447	20-03-2020	f	14	39	a	a	39	3735	33	55	a
448	21-03-2020	f	27	38	a	a	38	2945	32	55	a
449	21-03-2020	m	27	39	c	c	39	3580	36	53	a
450	30-03-2020	f	40	36	b	b	36	2315	32	48	a
451	30-03-2020	f	40	36	b	b	36	2380	33	47	a
452	12-04-2020	f	24	36	c	a	36	3490	33	56	a
453	12-04-2020	m	35	40	d	a	40	3880	32	56	a
454	13-04-2020	f	29	34	b	a	34	3520	33	56	a
455	14-04-2020	m	24	34	b	b	34	2520	32,5	52	a
456	14-04-2020	m	34	39	b	b	39	3215	32	53	a
457	14-04-2020	m	27	35	c	a	35	2980	36	54	a

No	Date of birth (DD-MM-YYYY)	Child's gender (f-female, m-male)	Mother's age (years)	Week of delivery (number)	Answer 1 (a-e)	Answer 2 (a-e)	Answer 3 (a-c)	Answer 4a (grams)	Answer 4b (cm)	Answer 4c (cm)	Answer 5 (Apgar)
458	14-04-2020	m	29	37	a	a	37	3750	34	57	a
459	14-04-2020	m	30	40	a	a	40	3875	36	57	a
460	16-04-2020	m	28	40	a	a	40	3630	35	57	a
461	16-04-2020	m	33	38	b	b	38	3620	36	57	a
462	16-04-2020	f	44	37	c	b	37	2750	34	50	a
463	17-04-2020	m	31	36	b	b	36	2475	32	51	a
464	17-04-2020	f	31	36	b	b	36	2690	34	53	a
465	17-04-2020	m	45	36	c	c	36	2880	32	50	a
466	18-04-2020	m	39	39	d	d	39	2885	34	46	a
467	18-04-2020	m	29	39	b	a	39	3650	33	52	a
468	21-04-2020	f	26	40	a	a	a	2850	34	51	a
469	21-04-2020	m	30	39	c	b	a	4515	33	58	a
470	21-04-2020	m	36	41	c	b	a	2930	33	57	a
471	21-04-2020	f	22	40	a	a	a	3900	35	53	a
472	21-04-2020	m	30	33	a	a	b	1800	31	45	a
473	21-04-2020	m	30	33	a	a	b	1695	30	48	a
474	24-04-2020	m	26	39	a	a	a	3360	34	55	a

No	Date of birth (DD-MM-YYYY)	Child's gender (f-female, m-male)	Mother's age (years)	Week of delivery (number)	Answer 1 (a-e)	Answer 2 (a-e)	Answer 3 (a-c)	Answer 4a (grams)	Answer 4b (cm)	Answer 4c (cm)	Answer 5 (Apgar)
475	18-04-2020	f	28	40	b	b	40	3200	32	54	a
476	12-03-2020	f	28	38	a	a	a	2920	33	52	a
477	24-04-2020	m	33	41	a	a	a	4100		59	a
478	n/d	n/d	30	40	b	a	40	3080	30	52	a
