# Supplementary material for: Estimation of Prenatal Alcohol Exposure: Comparison of Retrospective Survey and Measurement of Fatty Acid Ethyl Esters, Ethyl Sulfate, and Ethyl Glucuronide Concentrations in Neonatal Meconium
Source: Toxics. 2026 Feb 4;14(2):155. doi: 10.3390/toxics14020155 (PMC12944540; doi:10.3390/toxics14020155)
Supplement: Supplementary file 1 [file toxics-14-00155-s001.zip › Table S03 answers 6a-6o.pdf]

Table S3. Results of the survey questions 6a to 6o (n=478) in pregnant women conducted at the Neonatology Clinic of the Medical University of Gdańsk in the Pomeranian Province between June 16, 2019, and April 24, 2020.

**No**	**Answer 6a** (yes/no)	**Answer 6b** (yes/no)	**Answer 6c** (yes/no)	**Answer 6d** (yes/no)	**Answer 6e** (yes/no)	**Answer 6f** (yes/no)	**Answer 6g** (yes/no)	**Answer 6h** (yes/no)	**Answer 6i** (yes/no)	**Answer 6j** (yes/no)	**Answer 6k** (yes/no)	**Answer 6l** (yes/no)	**Answer 6m** (yes/no)	**Answer 6n** (yes/no)	**Answer 6o** (yes/no)
1	no	yes	no	no	no	no	no	no	no	no	no	no	no	no	no
2	no	no	no	no	no	yes	no	no	no	no	no	no	no	no	no
3	no	no	no	yes	no	yes	no	no	no	no	no	no	no	no	no
4	no	no	no	no	no	no	no	no	no	no	no	no	no	no	no
5	no	no	no	no	no	no	no	no	no	no	no	no	no	no	no
6	no	no	no	no	no	yes	no	no	no	no	no	no	no	no	no
7	no	no	no	yes	no	no	no	no	no	no	no	no	no	no	yes
8	no	no	no	no	no	yes	no	no	no	no	yes	no	no	no	no
9	no	no	no	no	no	yes	yes	yes	no	no	no	no	no	no	no
10	no	no	no	yes	no	yes	no	no	no	no	no	no	no	no	no
11	no	yes	no	no	no	no	no	no	no	no	no	no	no	no	no
12	no	no	no	yes	no	yes	no	no	no	no	no	no	no	no	no
13	no	no	no	no	no	yes	no	no	no	no	no	no	no	no	no
14	no	no	no	no	no	no	no	no	no	no	no	no	no	no	no
15	no	no	no	no	no	no	no	no	no	no	no	no	no	no	no
16	no	no	no	no	no	yes	no	no	no	no	no	no	no	no	no
17	no	no	no	yes	no	yes	no	no	no	no	yes	no	no	no	no
18	no	yes	no	no	no	no	no	no	no	no	no	no	no	no	no
19	no	no	no	no	no	no	no	no	no	no	no	no	no	no	no
20	no	no	no	yes	no	no	no	no	no	no	no	no	no	no	no
21	no	no	no	no	no	no	no	no	no	no	no	no	no	no	no
22	no	no	no	no	no	no	no	no	no	no	no	no	no	no	no

No	**Answer 6a** (yes/no)	**Answer 6b** (yes/no)	**Answer 6c** (yes/no)	**Answer 6d** (yes/no)	**Answer 6e** (yes/no)	**Answer 6f** (yes/no)	**Answer 6g** (yes/no)	**Answer 6h** (yes/no)	**Answer 6i** (yes/no)	**Answer 6j** (yes/no)	**Answer 6k** (yes/no)	**Answer 6l** (yes/no)	**Answer 6m** (yes/no)	**Answer 6n** (yes/no)	**Answer 6o** (yes/no)
23	no	no	no	no	no	yes	no	no	no	no	no	no	no	no	no
24	no	no	no	no	no	yes	no	no	no	no	yes	no	no	no	no
25	no	no	no	no	no	no	no	no	no	no	no	no	no	no	no
26	no	no	no	no	no	no	no	no	no	no	no	no	no	no	no
27	no	no	no	yes	no	no	no	no	no	no	no	no	no	no	no
28	no	no	no	yes	no	no	no	no	no	no	no	no	no	no	no
29	no	no	no	no	no	yes	no	no	no	no	yes	no	no	no	no
30	no	no	no	no	no	yes	no	no	no	no	no	no	no	no	no
31	no	no	no	no	no	no	no	no	no	no	no	no	no	no	no
32	no	no	no	no	no	no	no	no	no	no	no	no	no	no	no
33	no	no	no	no	no	yes	no	no	no	no	no	no	no	no	no
34	no	no	no	no	no	no	no	no	no	no	no	no	no	no	no
35	no	no	no	no	no	no	no	no	no	no	no	no	no	no	no
36	no	no	no	no	no	no	no	no	no	no	no	no	no	no	no
37	no	no	no	no	no	yes	no	no	no	no	no	no	no	no	yes
38	no	no	no	no	no	yes	no	no	no	no	no	no	no	no	no
39	no	no	no	no	no	no	no	no	no	no	no	no	no	no	no
40	no	no	no	no	no	no	no	no	no	no	no	no	no	no	no
41	no	no	no	no	no	no	no	no	no	no	no	no	no	no	no
42	yes	no	no	no	no	no	no	no	no	no	no	no	no	no	no
43	no	no	no	no	no	yes	no	no	no	no	no	no	no	no	no
44	no	no	no	no	no	yes	no	no	no	no	no	no	no	no	no
45	no	no	no	no	no	no	no	no	no	no	no	no	no	no	no
46	no	no	no	no	no	no	no	no	no	no	no	no	no	no	no
47	no	no	no	no	no	yes	no	no	no	no	no	no	no	no	no

No	**Answer 6a** (yes/no)	**Answer 6b** (yes/no)	**Answer 6c** (yes/no)	**Answer 6d** (yes/no)	**Answer 6e** (yes/no)	**Answer 6f** (yes/no)	**Answer 6g** (yes/no)	**Answer 6h** (yes/no)	**Answer 6i** (yes/no)	**Answer 6j** (yes/no)	**Answer 6k** (yes/no)	**Answer 6l** (yes/no)	**Answer 6m** (yes/no)	**Answer 6n** (yes/no)	**Answer 6o** (yes/no)
48	no	no	no	no	no	no	no	no	no	no	no	no	no	no	no
49	no	no	no	no	no	yes	no	no	no	no	yes	no	no	no	no
50	no	no	no	no	no	no	no	no	no	no	no	no	no	no	no
51	no	no	no	no	no	yes	no	no	no	no	no	no	no	no	no
52	no	no	no	no	no	yes	no	no	no	no	no	no	no	no	no
53	no	no	no	no	no	no	no	no	no	no	no	no	no	no	no
54	no	no	no	no	no	no	no	no	yes	no	no	no	no	no	no
55	no	no	no	no	no	no	no	no	no	no	no	no	no	no	no
56	no	yes	no	no	no	yes	no	no	no	no	no	no	no	no	no
57	no	no	no	no	no	yes	no	no	no	no	no	no	no	no	no
58	no	no	no	yes	no	no	no	no	no	no	no	no	no	no	no
59	no	no	no	no	no	yes	no	yes	no	no	no	no	no	no	no
60	no	no	no	no	no	no	no	no	no	no	no	no	no	no	no
61	no	no	no	no	no	no	no	no	no	no	no	no	no	yes	no
62	no	no	no	no	no	yes	no	no	no	no	no	no	no	no	no
63	no	no	no	no	no	no	no	no	no	no	no	no	no	no	no
64	no	no	no	no	no	no	no	no	no	no	no	no	no	no	no
65	no	no	no	no	no	no	no	no	no	no	no	no	no	no	yes
66	no	no	no	no	no	no	no	no	no	no	no	no	no	no	no
67	no	yes	no	no	no	no	no	no	no	no	no	no	no	no	no
68	no	no	no	no	no	no	no	no	no	no	no	no	no	no	no
69	no	no	no	no	no	no	no	no	no	no	no	no	no	no	no
70	no	no	no	no	no	no	no	no	no	no	no	no	no	no	no
71	no	no	no	no	no	yes	no	no	no	no	no	no	no	no	no
72	no	no	no	no	no	no	no	no	no	no	no	no	no	no	yes

No	**Answer 6a** (yes/no)	**Answer 6b** (yes/no)	**Answer 6c** (yes/no)	**Answer 6d** (yes/no)	**Answer 6e** (yes/no)	**Answer 6f** (yes/no)	**Answer 6g** (yes/no)	**Answer 6h** (yes/no)	**Answer 6i** (yes/no)	**Answer 6j** (yes/no)	**Answer 6k** (yes/no)	**Answer 6l** (yes/no)	**Answer 6m** (yes/no)	**Answer 6n** (yes/no)	**Answer 6o** (yes/no)
73	yes	yes	no	no	no	no	no	no	no	no	no	no	no	no	no
74	no	no	no	no	no	no	no	no	no	no	no	no	no	no	no
75	no	no	no	no	no	no	no	no	no	no	no	no	no	no	no
76	no	no	no	no	no	no	no	no	no	no	no	no	no	no	no
77	no	no	no	no	no	yes	no	no	no	no	no	no	no	no	no
78	no	no	no	no	no	yes	no	yes	no	no	no	no	no	no	no
79	no	no	no	no	no	yes	no	no	no	no	yes	no	no	no	no
80	no	no	no	no	no	no	no	no	no	no	no	no	no	no	no
81	no	no	no	no	no	no	no	no	no	no	no	no	no	no	no
82	no	no	no	no	no	no	no	no	no	no	no	no	no	no	no
83	no	no	no	no	no	yes	no	no	no	no	no	no	no	no	no
84	no	no	no	no	no	no	no	no	no	no	no	no	no	no	no
85	no	no	no	no	no	no	no	no	no	no	no	no	no	no	no
86	no	no	no	no	no	yes	no	no	no	no	no	no	no	no	no
87	no	no	no	no	no	no	no	no	no	no	no	no	no	no	no
88	no	no	no	no	no	no	no	no	no	no	no	no	no	no	no
89	no	no	no	yes	no	yes	no	no	yes	no	no	no	no	no	no
90	no	no	no	no	no	no	no	no	no	no	no	no	no	no	no
91	no	no	no	no	no	yes	no	no	no	no	no	no	no	no	no
92	yes	no	no	yes	no	yes	no	no	no	no	no	no	no	no	yes
93	no	no	no	no	no	no	no	no	no	no	no	no	no	no	no
94	no	no	no	no	no	yes	no	no	no	no	no	no	no	no	no
95	no	no	no	no	no	yes	no	no	no	no	yes	no	no	no	no
96	no	no	no	no	no	yes	no	no	no	no	no	no	no	no	no
97	no	no	yes	no	yes	no	no	yes	no	no	no	no	no	no	no

No	**Answer 6a** (yes/no)	**Answer 6b** (yes/no)	**Answer 6c** (yes/no)	**Answer 6d** (yes/no)	**Answer 6e** (yes/no)	**Answer 6f** (yes/no)	**Answer 6g** (yes/no)	**Answer 6h** (yes/no)	**Answer 6i** (yes/no)	**Answer 6j** (yes/no)	**Answer 6k** (yes/no)	**Answer 6l** (yes/no)	**Answer 6m** (yes/no)	**Answer 6n** (yes/no)	**Answer 6o** (yes/no)
98	no	no	no	no	no	yes	no	no	no	yes	yes	no	no	no	no
99	no	no	no	yes	no	yes	no	no	no	no	no	no	no	no	no
100	no	no	no	no	no	no	yes	yes	no	no	no	no	no	no	no
101	no	no	no	no	no	yes	no	no	no	no	no	no	no	no	no
102	no	no	no	no	no	no	no	no	no	yes	no	no	no	no	no
103	no	no	no	no	no	no	no	no	no	no	no	no	no	no	no
104	no	no	no	no	no	no	no	no	no	no	yes	no	no	no	no
105	no	no	no	no	no	no	no	no	no	no	yes	no	no	no	no
106	no	no	no	no	no	yes	no	no	no	no	no	no	no	no	no
107	no	no	no	no	no	no	no	no	no	no	no	no	no	no	no
108	no	no	no	no	no	yes	no	no	no	no	no	no	no	no	no
109	no	no	no	no	no	no	no	no	no	no	no	no	no	no	no
110	no	no	no	no	no	yes	no	no	no	no	no	no	no	no	no
111	no	no	no	yes	no	no	no	no	no	no	no	no	no	no	no
112	no	no	no	no	no	no	no	no	no	no	no	no	no	no	no
113	no	no	no	no	no	no	no	no	no	no	no	no	no	no	no
114	no	no	no	yes	yes	yes	no	no	no	no	no	no	no	no	no
115	no	no	no	no	no	yes	no	no	no	no	no	no	no	no	no
116	no	no	no	no	no	yes	no	no	no	no	no	no	no	no	no
117	no	no	no	no	no	yes	no	no	no	no	no	no	no	no	no
118	no	no	no	no	no	no	no	no	no	no	no	no	no	no	no
119	no	no	no	no	no	no	no	no	no	no	no	no	no	no	no
120	no	no	no	no	no	no	no	yes	no	no	no	no	no	no	no
121	no	no	no	no	no	yes	no	no	no	no	no	no	no	no	no
122	no	no	no	yes	no	yes	no	no	no	no	no	no	no	no	no

No	**Answer 6a** (yes/no)	**Answer 6b** (yes/no)	**Answer 6c** (yes/no)	**Answer 6d** (yes/no)	**Answer 6e** (yes/no)	**Answer 6f** (yes/no)	**Answer 6g** (yes/no)	**Answer 6h** (yes/no)	**Answer 6i** (yes/no)	**Answer 6j** (yes/no)	**Answer 6k** (yes/no)	**Answer 6l** (yes/no)	**Answer 6m** (yes/no)	**Answer 6n** (yes/no)	**Answer 6o** (yes/no)
123	no	no	no	no	no	no	no	no	no	no	no	no	no	no	yes
124	no	no	no	yes	no	yes	no	no	no	no	no	no	no	no	no
125	no	no	no	no	no	no	no	no	no	no	no	no	no	no	no
126	no	no	no	no	no	no	no	no	no	no	no	no	no	no	no
127	no	no	no	yes	no	yes	no	no	no	no	no	no	no	no	no
128	no	no	no	no	no	yes	no	no	no	no	yes	no	no	no	no
129	no	no	no	no	no	no	no	no	no	no	no	no	no	no	yes
130	no	no	no	no	no	no	no	no	no	no	no	no	no	no	no
131	no	no	no	yes	no	yes	no	no	no	no	no	no	no	no	no
132	no	no	no	no	no	no	no	no	no	no	yes	no	no	no	no
133	no	no	no	no	no	yes	no	no	no	no	no	no	no	no	no
134	yes	yes	no	yes	no	no	no	no	no	no	no	no	no	no	no
135	no	no	no	no	no	no	no	no	no	no	no	no	no	no	no
136	no	no	no	no	no	no	no	no	no	no	no	no	no	no	yes
137	no	no	no	no	no	no	no	no	no	no	no	no	no	no	no
138	no	no	no	no	no	no	no	no	no	no	no	no	no	no	no
139	no	no	no	no	no	yes	no	no	no	no	no	no	no	no	no
140	no	no	no	no	no	no	no	no	no	no	no	no	no	no	yes
141	no	no	no	no	no	no	no	no	no	no	no	no	no	no	no
142	no	yes	no	no	no	no	no	no	no	no	no	no	no	no	no
143	no	no	no	no	no	yes	no	no	no	no	no	no	no	no	no
144	no	no	no	no	no	no	no	no	no	no	no	no	no	no	yes
145	no	no	no	no	no	no	no	no	no	no	no	no	no	no	no
146	no	yes	no	no	no	yes	no	no	no	no	no	no	no	no	no
147	no	no	no	no	no	no	no	no	no	no	yes	no	no	no	no

No	Answer 6a (yes/no)	Answer 6b (yes/no)	Answer 6c (yes/no)	Answer 6d (yes/no)	Answer 6e (yes/no)	Answer 6f (yes/no)	Answer 6g (yes/no)	Answer 6h (yes/no)	Answer 6i (yes/no)	Answer 6j (yes/no)	Answer 6k (yes/no)	Answer 6l (yes/no)	Answer 6m (yes/no)	Answer 6n (yes/no)	Answer 6o (yes/no)
148	no	no	no	no	no	yes	no	no	no	no	no	no	no	no	no
149	no	no	no	no	no	no	no	no	no	no	no	no	no	no	no
150	no	yes	no	no	no	no	no	no	no	no	no	no	no	no	no
151	no	no	no	no	no	no	no	no	no	no	yes	no	no	no	no
152	no	no	no	no	no	yes	no	no	no	no	no	no	no	no	no
153	no	no	no	no	no	no	no	no	no	no	yes	no	no	no	no
154	no	no	no	no	no	no	no	no	no	no	no	no	no	no	no
155	no	no	no	no	no	no	no	yes	no	no	no	no	no	no	no
156	no	no	no	no	no	yes	no	no	no	no	no	no	no	no	no
157	yes	no	no	no	no	no	no	no	no	no	no	no	no	no	no
158	no	no	no	no	no	no	no	no	no	no	no	no	no	no	no
159	no	no	no	no	no	yes	no	no	no	no	yes	no	no	no	no
160	no	no	no	no	no	yes	no	no	no	no	no	no	no	no	no
161	no	no	no	no	no	yes	no	no	no	no	yes	no	no	no	no
162	no	no	no	yes	no	yes	no	no	no	no	no	no	no	no	no
163	no	no	no	yes	no	no	no	no	no	no	no	no	no	no	no
164	no	no	no	no	no	no	no	no	no	no	no	no	no	no	no
165	no	no	no	yes	no	no	no	no	no	no	no	no	no	no	no
166	no	no	no	no	no	yes	no	no	no	no	no	no	no	yes	no
167	no	no	no	no	no	no	no	no	no	no	yes	no	no	no	no
168	no	no	no	no	no	no	no	no	no	no	yes	no	no	no	no
169	no	no	no	no	no	no	no	no	no	no	no	no	no	no	no
170	no	no	no	no	no	no	no	no	no	yes	yes	no	no	yes	yes
171	no	no	no	no	no	no	no	no	no	yes	yes	no	no	yes	yes
172	no	yes	no	no	no	no	yes	yes	no	no	no	no	no	no	no

No	**Answer 6a** (yes/no)	**Answer 6b** (yes/no)	**Answer 6c** (yes/no)	**Answer 6d** (yes/no)	**Answer 6e** (yes/no)	**Answer 6f** (yes/no)	**Answer 6g** (yes/no)	**Answer 6h** (yes/no)	**Answer 6i** (yes/no)	**Answer 6j** (yes/no)	**Answer 6k** (yes/no)	**Answer 6l** (yes/no)	**Answer 6m** (yes/no)	**Answer 6n** (yes/no)	**Answer 6o** (yes/no)
173	yes	no	no	yes	no	no	no	no	no	no	no	no	no	no	no
174	no	no	no	no	yes	no	no	no	no	no	no	no	no	no	no
175	no	no	no	no	no	no	no	no	no	no	no	no	no	no	no
176	no	no	no	no	no	yes	no	no	no	no	no	no	no	no	no
177	no	no	no	no	no	yes	no	no	no	no	yes	no	no	no	no
178	no	no	yes	no	no	no	no	no	no	no	no	no	no	no	no
179	no	no	no	yes	no	no	no	no	no	yes	no	no	no	no	yes
180	no	no	no	no	no	no	no	no	no	no	no	no	no	no	no
181	no	no	no	no	no	no	no	no	no	no	no	no	no	no	no
182	no	no	no	no	no	no	no	no	no	no	no	no	no	no	no
183	no	no	no	yes	no	no	no	no	no	no	yes	no	no	no	no
184	no	no	no	yes	no	no	no	no	no	no	no	no	no	no	no
185	no	no	no	yes	no	no	no	no	no	no	no	no	no	no	no
186	no	no	no	no	no	no	no	no	no	no	no	no	no	no	no
187	no	no	no	no	no	no	no	no	no	no	no	no	no	no	no
188	no	no	no	no	no	yes	no	no	no	no	no	no	no	no	no
189	no	no	no	no	no	no	no	no	no	no	no	no	no	no	no
190	no	no	no	no	no	yes	no	no	no	no	no	no	no	no	no
191	no	no	no	yes	no	no	no	no	no	no	no	no	no	no	no
192	no	no	no	no	no	no	no	no	no	no	no	no	no	no	no
193	no	no	no	no	no	yes	no	no	no	no	yes	no	no	no	yes
194	no	no	no	no	no	yes	no	no	no	no	no	no	no	no	no
195	no	no	no	yes	no	no	no	no	no	no	no	no	no	no	no
196	no	no	no	no	no	yes	no	no	no	no	no	no	no	no	no
197	no	no	no	no	no	yes	no	no	no	no	no	no	no	no	no

No	**Answer 6a** (yes/no)	**Answer 6b** (yes/no)	**Answer 6c** (yes/no)	**Answer 6d** (yes/no)	**Answer 6e** (yes/no)	**Answer 6f** (yes/no)	**Answer 6g** (yes/no)	**Answer 6h** (yes/no)	**Answer 6i** (yes/no)	**Answer 6j** (yes/no)	**Answer 6k** (yes/no)	**Answer 6l** (yes/no)	**Answer 6m** (yes/no)	**Answer 6n** (yes/no)	**Answer 6o** (yes/no)
198	no	no	no	no	no	no	no	no	no	yes	no	no	no	no	no
199	no	no	no	no	no	yes	no	no	no	no	no	no	no	no	no
200	no	no	no	no	no	no	no	no	no	no	yes	no	no	no	no
201	no	no	no	no	no	no	no	no	no	no	yes	no	no	no	no
202	no	no	no	no	no	yes	no	no	no	no	no	no	no	no	no
203	no	yes	no	no	no	yes	no	no	no	no	no	no	no	no	no
204	no	no	no	no	no	no	no	no	no	no	no	no	no	no	yes
205	no	no	no	no	no	no	no	no	no	no	no	no	no	no	yes
206	no	no	no	no	no	yes	no	no	no	no	no	no	no	no	no
207	no	no	no	no	no	no	no	no	no	no	no	no	no	no	yes
208	no	no	no	no	no	no	no	no	no	no	no	no	no	no	no
209	no	no	no	no	no	no	no	no	no	no	yes	no	no	no	no
210	no	no	no	no	no	no	no	no	no	no	no	yes	no	no	no
211	no	no	no	no	no	no	no	no	no	no	no	no	no	no	no
212	no	no	no	no	no	no	no	yes	no	no	yes	no	no	no	no
213	no	no	no	yes	no	no	no	no	no	no	no	no	no	no	no
214	no	no	no	no	no	yes	yes	no	no	no	no	no	no	no	no
215	no	no	no	no	no	no	no	no	no	no	no	no	no	no	no
216	no	no	no	yes	no	yes	no	no	no	no	no	no	no	no	no
217	no	no	yes	no	no	yes	no	no	no	no	no	no	no	no	no
218	no	no	no	no	no	yes	no	no	no	no	no	no	no	no	no
219	no	no	no	no	no	yes	no	no	no	no	no	no	no	no	no
220	no	no	no	yes	no	no	no	no	no	no	no	no	no	no	no
221	no	no	no	yes	no	no	no	no	no	no	no	no	no	no	no
222	no	no	no	yes	no	no	no	no	no	no	no	no	no	no	no

No	Answer 6a (yes/no)	Answer 6b (yes/no)	Answer 6c (yes/no)	Answer 6d (yes/no)	Answer 6e (yes/no)	Answer 6f (yes/no)	Answer 6g (yes/no)	Answer 6h (yes/no)	Answer 6i (yes/no)	Answer 6j (yes/no)	Answer 6k (yes/no)	Answer 6l (yes/no)	Answer 6m (yes/no)	Answer 6n (yes/no)	Answer 6o (yes/no)
223	no	no	no	no	no	no	no	no	no	no	no	no	no	no	no
224	no	no	no	no	no	yes	no	no	no	no	no	no	no	no	no
225	no	no	no	no	no	yes	no	no	no	no	yes	no	no	no	no
226	no	no	no	no	no	no	no	no	no	no	no	no	no	no	no
227	no	no	yes	no	no	yes	no	no	no	no	no	no	no	no	no
228	no	no	no	no	no	yes	no	no	no	no	yes	no	no	no	no
229	no	no	no	yes	no	no	no	no	no	no	no	no	no	no	no
230	no	no	no	no	no	yes	no	no	no	no	no	no	no	no	no
231	no	no	no	no	no	no	no	no	no	no	no	no	no	no	no
232	no	no	no	no	no	no	no	no	no	no	no	no	no	no	no
233	no	no	no	no	no	yes	no	no	no	no	yes	no	no	no	no
234	no	no	no	no	no	no	no	no	no	no	no	no	no	no	no
235	no	no	no	no	no	no	no	no	no	no	no	no	no	no	no
236	no	no	no	no	no	no	no	no	no	no	no	no	no	no	no
237	no	no	no	no	no	yes	no	no	no	no	no	no	no	no	no
238	no	no	no	no	no	no	no	yes	no	no	no	no	no	no	no
239	no	no	no	no	no	no	no	no	no	no	no	no	no	no	no
240	no	no	no	no	no	no	no	yes	no	no	no	no	no	no	no
241	no	no	no	no	no	no	no	no	no	no	no	no	no	no	no
242	no	no	no	no	no	yes	no	no	no	no	no	no	no	no	no
243	no	no	no	no	no	no	no	no	no	no	no	no	no	no	no
244	no	no	no	no	no	yes	no	no	no	no	no	no	no	no	no
245	no	no	no	no	no	no	no	yes	no	no	no	no	no	no	no
246	no	no	no	yes	no	yes	no	no	no	no	no	no	no	no	no
247	no	no	yes	no	no	yes	no	no	no	no	no	no	no	no	no

No	**Answer 6a** (yes/no)	**Answer 6b** (yes/no)	**Answer 6c** (yes/no)	**Answer 6d** (yes/no)	**Answer 6e** (yes/no)	**Answer 6f** (yes/no)	**Answer 6g** (yes/no)	**Answer 6h** (yes/no)	**Answer 6i** (yes/no)	**Answer 6j** (yes/no)	**Answer 6k** (yes/no)	**Answer 6l** (yes/no)	**Answer 6m** (yes/no)	**Answer 6n** (yes/no)	**Answer 6o** (yes/no)
248	no	no	no	yes	no	no	no	no	no	no	no	no	no	no	no
249	no	no	yes	no	no	yes	no	no	no	no	no	no	no	no	no
250	no	no	yes	no	no	yes	no	no	no	no	no	no	no	no	yes
251	no	no	no	no	no	yes	no	no	no	no	no	no	no	no	no
252	no	no	no	no	no	no	no	no	no	no	no	no	no	no	yes
253	no	no	no	no	no	no	no	no	no	no	no	no	no	no	no
254	no	no	no	no	no	yes	no	no	no	no	no	no	no	no	no
255	no	no	no	no	no	no	no	no	no	no	no	no	no	no	no
256	no	no	no	no	no	no	no	no	no	no	no	no	no	no	no
257	no	no	no	no	no	no	no	no	no	no	no	no	no	no	no
258	no	no	no	no	no	no	no	no	no	no	no	no	no	no	no
259	no	yes	yes	no	no	yes	no	no	no	no	no	no	no	no	no
260	no	no	no	yes	no	yes	no	no	no	no	no	no	no	yes	no
261	no	no	no	no	no	no	no	no	no	no	no	no	no	no	no
262	yes	yes	no	no	no	yes	no	no	no	no	no	no	no	no	no
263	no	no	no	no	no	yes	no	no	no	no	no	no	no	no	no
264	no	no	no	no	no	yes	no	no	no	no	no	no	no	no	no
265	no	no	no	no	no	no	no	no	no	no	no	no	no	no	no
266	no	yes	no	no	no	no	no	no	no	no	no	no	no	no	yes
267	no	yes	no	yes	no	yes	no	no	no	no	no	no	no	no	no
268	no	no	no	no	no	no	no	no	no	no	yes	no	no	no	no
269	no	no	no	no	no	no	no	no	no	no	yes	no	no	no	no
270	no	no	no	no	no	yes	no	no	no	no	no	no	no	no	no
271	no	no	no	yes	no	yes	no	no	no	no	no	no	no	no	no
272	no	no	no	no	no	no	no	no	no	no	no	no	no	no	no

No	**Answer 6a** (yes/no)	**Answer 6b** (yes/no)	**Answer 6c** (yes/no)	**Answer 6d** (yes/no)	**Answer 6e** (yes/no)	**Answer 6f** (yes/no)	**Answer 6g** (yes/no)	**Answer 6h** (yes/no)	**Answer 6i** (yes/no)	**Answer 6j** (yes/no)	**Answer 6k** (yes/no)	**Answer 6l** (yes/no)	**Answer 6m** (yes/no)	**Answer 6n** (yes/no)	**Answer 6o** (yes/no)
273	no	yes	no	no	no	yes	no	no	no	no	no	no	no	no	no
274	no	yes	no	no	no	no	no	no	no	yes	no	no	no	no	no
275	no	no	no	no	no	yes	no	no	no	no	no	no	no	no	no
276	no	no	no	no	no	no	no	no	no	no	no	no	no	no	no
277	no	no	no	no	no	no	no	no	no	no	no	no	no	no	no
278	no	no	no	no	no	no	no	no	no	no	no	no	no	yes	no
279	no	no	no	no	no	no	no	no	no	no	no	no	no	no	no
280	no	no	no	no	no	yes	no	yes	no	no	no	no	no	no	no
281	no	no	no	yes	no	no	no	no	no	no	no	no	no	no	no
282	no	no	no	yes	no	no	no	yes	no	no	yes	no	no	no	no
283	no	no	no	no	no	yes	no	no	no	no	no	no	no	no	no
284	no	no	no	no	no	no	no	no	no	no	no	no	no	no	no
285	no	no	no	no	no	no	no	no	no	no	no	no	no	no	no
286	no	yes	no	yes	no	yes	no	no	no	no	no	no	no	no	no
287	no	no	no	no	no	yes	no	no	no	no	no	no	no	no	no
288	no	no	no	no	no	yes	no	no	no	no	no	no	no	no	no
289	no	no	no	no	no	no	no	no	no	no	no	no	no	no	yes
290	no	no	no	yes	no	no	no	no	no	no	no	no	no	no	no
291	no	no	no	no	no	no	no	no	no	no	no	no	no	no	no
292	no	no	no	no	no	yes	no	no	no	no	no	no	no	no	no
293	no	no	no	no	no	yes	no	no	no	no	no	no	no	no	no
294	no	no	no	no	no	yes	yes	yes	no	no	no	no	no	no	no
295	no	no	no	no	no	no	no	no	no	no	no	no	no	no	no
296	no	no	no	no	no	no	no	no	no	no	no	no	no	no	no
297	no	no	no	no	no	no	no	no	no	no	yes	no	no	no	no

No	**Answer 6a** (yes/no)	**Answer 6b** (yes/no)	**Answer 6c** (yes/no)	**Answer 6d** (yes/no)	**Answer 6e** (yes/no)	**Answer 6f** (yes/no)	**Answer 6g** (yes/no)	**Answer 6h** (yes/no)	**Answer 6i** (yes/no)	**Answer 6j** (yes/no)	**Answer 6k** (yes/no)	**Answer 6l** (yes/no)	**Answer 6m** (yes/no)	**Answer 6n** (yes/no)	**Answer 6o** (yes/no)
298	no	no	no	yes	no	yes	no	no	no	no	no	no	no	no	no
299	no	no	no	no	no	yes	no	no	no	no	no	no	no	no	no
300	no	no	no	no	no	no	no	no	no	no	no	no	no	no	no
301	no	no	no	no	no	no	no	no	no	no	no	no	no	no	no
302	no	no	no	no	no	no	no	no	no	no	no	no	no	no	no
303	no	yes	no	yes	no	no	no	no	no	no	no	no	no	no	no
304	no	yes	no	yes	no	no	no	no	no	no	no	no	no	no	no
305	no	no	no	no	no	no	no	no	no	no	no	no	no	yes	no
306	no	no	no	no	no	no	no	no	no	no	no	no	no	no	no
307	no	no	no	no	no	yes	no	no	no	no	no	no	no	no	no
308	no	no	no	no	no	no	no	no	no	no	no	no	no	no	no
309	no	no	no	no	no	no	no	no	no	no	no	no	no	no	no
310	no	no	no	no	no	yes	no	no	no	no	no	no	no	no	no
311	no	no	no	no	no	yes	no	no	no	no	yes	no	no	no	no
312	no	no	no	no	no	yes	no	no	no	no	no	no	no	no	no
313	no	no	no	no	no	no	no	no	no	no	no	no	no	no	yes
314	no	no	no	no	no	no	no	no	no	no	no	no	no	no	no
315	no	no	no	no	no	no	no	no	no	no	no	no	no	no	no
316	no	no	no	yes	no	yes	no	no	no	no	no	no	no	no	no
317	no	no	no	no	no	no	no	no	no	no	no	no	no	no	no
318	no	no	no	no	no	yes	no	no	no	no	no	no	no	no	no
319	no	no	no	no	no	no	no	no	no	no	no	no	no	yes	no
320	no	no	no	no	no	no	no	no	no	no	no	no	no	yes	no
321	no	yes	no	no	no	no	no	no	no	no	no	no	no	no	no
322	no	no	no	no	no	no	no	no	no	no	no	no	no	no	no

No	Answer 6a (yes/no)	Answer 6b (yes/no)	Answer 6c (yes/no)	Answer 6d (yes/no)	Answer 6e (yes/no)	Answer 6f (yes/no)	Answer 6g (yes/no)	Answer 6h (yes/no)	Answer 6i (yes/no)	Answer 6j (yes/no)	Answer 6k (yes/no)	Answer 6l (yes/no)	Answer 6m (yes/no)	Answer 6n (yes/no)	Answer 6o (yes/no)
323	no	no	no	yes	no	yes	no	no	no	no	no	no	no	no	no
324	no	no	no	no	no	yes	no	no	no	no	no	no	no	no	no
325	no	no	no	no	no	no	no	no	no	no	no	no	no	no	no
326	no	no	no	no	no	no	no	no	no	no	no	no	no	no	no
327	no	no	no	no	no	no	no	no	no	no	no	no	no	no	no
328	no	no	no	no	no	yes	no	no	no	no	no	no	no	no	no
329	no	no	no	no	no	no	no	no	no	no	no	no	no	no	no
330	no	no	no	yes	no	yes	no	no	no	no	no	no	no	no	no
331	no	no	no	no	no	yes	no	no	no	no	no	no	no	no	no
332	no	no	yes	no	no	yes	no	no	no	no	no	no	no	no	no
333	no	no	no	no	no	no	no	no	no	no	yes	no	no	no	no
334	no	no	no	no	no	no	yes	no	no	yes	no	no	no	no	yes
335	no	no	no	no	no	yes	no	no	no	no	no	no	no	no	no
336	no	no	no	no	no	no	no	no	no	no	no	no	no	no	yes
337	no	no	no	no	no	yes	no	no	no	no	no	no	no	no	no
338	no	no	no	no	no	no	no	no	no	no	no	no	no	no	no
339	no	no	no	no	no	yes	no	no	no	no	no	no	no	no	no
340	no	no	no	no	no	no	no	no	no	no	no	no	no	no	no
341	no	no	no	no	no	no	no	no	no	no	no	no	no	no	no
342	no	no	no	no	no	no	no	no	no	no	no	no	no	no	no
343	no	no	no	no	no	no	no	no	no	no	no	no	no	no	yes
344	no	no	no	no	no	no	no	no	no	no	no	no	no	no	no
345	no	no	no	no	no	no	no	no	no	no	no	no	no	no	no
346	no	no	no	no	no	no	no	no	no	no	no	no	no	no	no
347	no	yes	no	yes	no	yes	no	no	no	no	no	no	no	no	no

No	**Answer 6a** (yes/no)	**Answer 6b** (yes/no)	**Answer 6c** (yes/no)	**Answer 6d** (yes/no)	**Answer 6e** (yes/no)	**Answer 6f** (yes/no)	**Answer 6g** (yes/no)	**Answer 6h** (yes/no)	**Answer 6i** (yes/no)	**Answer 6j** (yes/no)	**Answer 6k** (yes/no)	**Answer 6l** (yes/no)	**Answer 6m** (yes/no)	**Answer 6n** (yes/no)	**Answer 6o** (yes/no)
348	no	no	no	no	no	yes	no	no	no	no	no	no	no	no	no
349	no	no	no	no	no	yes	no	no	no	no	no	no	no	no	no
350	no	no	no	no	no	no	no	no	no	no	no	no	no	no	no
351	no	no	no	no	no	no	no	no	no	no	no	no	no	no	no
352	no	no	no	no	no	yes	no	no	no	no	no	no	no	no	no
353	no	no	no	no	no	no	no	no	no	no	no	no	no	no	no
354	no	no	no	no	no	no	no	no	no	no	yes	no	no	no	no
355	no	no	no	no	no	no	no	no	no	no	no	no	no	no	no
356	no	no	no	no	no	no	no	no	no	no	no	no	no	no	no
357	no	yes	no	no	no	no	no	no	no	no	no	no	no	yes	no
358	no	yes	no	no	no	no	no	no	no	no	no	no	no	yes	no
359	no	no	no	yes	no	yes	no	no	no	no	no	no	no	no	no
360	no	no	no	no	no	yes	no	no	no	no	no	no	no	no	no
361	no	no	no	yes	no	yes	no	no	no	no	no	no	no	no	no
362	no	no	no	no	no	no	no	no	no	no	no	no	no	no	no
363	no	no	no	no	no	yes	no	no	no	no	no	no	no	no	no
364	no	yes	no	no	no	no	no	no	no	no	no	no	no	no	no
365	no	no	no	no	no	no	no	no	no	no	no	no	no	no	no
366	no	no	no	no	no	no	no	no	no	no	no	no	no	no	no
367	no	no	no	no	no	no	no	no	no	no	no	no	no	no	no
368	no	no	no	no	no	yes	no	no	no	no	no	no	no	no	no
369	no	no	no	no	no	no	no	no	no	no	no	no	no	no	no
370	no	no	no	no	no	no	no	no	no	no	no	no	no	no	no
371	no	no	yes	no	no	yes	no	no	no	no	no	no	no	no	no
372	no	no	no	no	no	yes	no	no	no	no	no	no	no	no	no

No	**Answer 6a** (yes/no)	**Answer 6b** (yes/no)	**Answer 6c** (yes/no)	**Answer 6d** (yes/no)	**Answer 6e** (yes/no)	**Answer 6f** (yes/no)	**Answer 6g** (yes/no)	**Answer 6h** (yes/no)	**Answer 6i** (yes/no)	**Answer 6j** (yes/no)	**Answer 6k** (yes/no)	**Answer 6l** (yes/no)	**Answer 6m** (yes/no)	**Answer 6n** (yes/no)	**Answer 6o** (yes/no)
373	yes	no	no	no	no	no	no	no	no	no	no	no	no	no	no
374	no	no	no	no	no	yes	no	no	no	no	no	no	no	no	no
375	no	no	no	no	no	yes	no	no	no	no	no	no	no	no	no
376	no	no	no	yes	no	yes	no	no	no	no	no	no	no	no	no
377	no	no	no	no	no	no	no	no	no	no	yes	no	no	no	no
378	no	no	no	no	no	yes	no	no	no	no	no	no	no	no	no
379	no	no	no	no	no	yes	no	no	no	no	no	no	no	no	no
380	yes	no	no	yes	no	no	no	no	no	no	no	no	no	no	no
381	no	no	no	yes	no	no	no	no	no	no	no	no	no	no	no
382	no	no	no	no	no	yes	no	no	no	no	no	no	no	no	no
383	no	no	no	no	no	no	no	no	no	no	no	no	no	no	yes
384	no	no	no	yes	no	yes	no	no	no	no	no	no	no	no	no
385	no	no	no	no	no	no	no	no	no	no	no	no	yes	no	no
386	no	no	no	yes	no	no	no	no	no	no	no	no	no	no	no
387	no	no	no	no	no	no	no	no	no	no	no	no	no	no	no
388	no	yes	no	yes	no	no	no	no	no	no	no	no	no	no	no
389	no	no	no	no	no	no	no	no	no	no	no	no	no	no	no
390	no	no	no	no	no	yes	no	no	no	no	no	no	no	no	no
391	no	no	no	yes	no	no	no	no	no	no	no	no	no	no	no
392	no	no	no	no	no	yes	no	no	no	no	no	no	no	no	no
393	no	no	no	no	no	no	no	no	no	no	no	no	no	no	no
394	no	no	no	no	no	no	no	no	no	no	yes	no	no	no	no
395	no	no	no	yes	no	yes	no	no	no	no	no	no	no	no	no
396	no	yes	no	yes	no	yes	no	no	no	no	no	no	no	no	no
397	no	yes	no	no	no	yes	no	no	no	no	yes	no	no	no	no

No	**Answer 6a** (yes/no)	**Answer 6b** (yes/no)	**Answer 6c** (yes/no)	**Answer 6d** (yes/no)	**Answer 6e** (yes/no)	**Answer 6f** (yes/no)	**Answer 6g** (yes/no)	**Answer 6h** (yes/no)	**Answer 6i** (yes/no)	**Answer 6j** (yes/no)	**Answer 6k** (yes/no)	**Answer 6l** (yes/no)	**Answer 6m** (yes/no)	**Answer 6n** (yes/no)	**Answer 6o** (yes/no)
398	no	no	no	no	no	yes	no	no	no	no	no	no	no	no	no
399	no	no	no	no	no	no	no	no	no	no	no	no	no	no	no
400	no	no	no	yes	no	yes	no	no	no	no	no	no	no	yes	no
401	no	no	no	no	no	no	no	no	no	no	no	no	no	no	no
402	no	no	no	yes	no	no	no	no	no	no	no	no	no	no	no
403	no	no	no	no	no	yes	no	no	no	no	no	no	no	no	no
404	no	no	no	no	no	no	no	no	no	no	no	no	no	no	no
405	no	no	no	no	no	no	no	no	no	no	no	no	no	no	yes
406	no	no	no	yes	no	no	no	no	no	no	no	no	no	no	no
407	no	no	no	yes	no	no	no	no	no	no	no	no	no	no	no
408	no	no	no	no	no	yes	no	no	no	no	no	no	no	no	no
409	no	yes	no	yes	no	yes	no	no	no	no	no	no	no	no	no
410	no	yes	no	yes	no	no	no	no	no	no	no	no	no	no	no
411	no	no	no	yes	no	yes	no	yes	no	no	no	no	no	yes	yes
412	no	no	no	no	no	no	no	no	no	no	no	no	no	no	no
413	no	no	no	no	no	no	no	no	no	no	no	no	no	no	no
414	no	no	no	no	no	no	no	no	no	no	no	no	no	no	yes
415	no	yes	no	no	no	yes	no	no	no	no	no	no	no	no	no
416	no	yes	no	no	no	yes	no	no	no	no	no	no	no	no	no
417	no	no	no	no	no	no	no	no	no	no	no	no	no	no	no
418	no	no	no	no	no	no	no	no	no	no	no	no	no	no	no
419	no	yes	no	yes	no	yes	no	no	no	no	no	no	no	no	no
420	no	no	no	no	no	no	no	no	no	no	no	no	no	no	no
421	no	no	no	no	no	no	no	no	no	no	no	no	no	no	no
422	no	no	no	no	no	no	no	no	no	no	no	no	no	no	no

No	**Answer 6a** (yes/no)	**Answer 6b** (yes/no)	**Answer 6c** (yes/no)	**Answer 6d** (yes/no)	**Answer 6e** (yes/no)	**Answer 6f** (yes/no)	**Answer 6g** (yes/no)	**Answer 6h** (yes/no)	**Answer 6i** (yes/no)	**Answer 6j** (yes/no)	**Answer 6k** (yes/no)	**Answer 6l** (yes/no)	**Answer 6m** (yes/no)	**Answer 6n** (yes/no)	**Answer 6o** (yes/no)
423	no	no	no	no	no	yes	no	no	no	no	no	no	no	no	no
424	no	no	no	no	no	yes	no	no	no	no	no	no	no	no	no
425	no	no	no	no	no	yes	no	no	no	no	no	no	no	no	no
426	no	no	no	no	no	no	no	no	no	no	no	no	no	no	no
427	no	no	no	no	no	no	no	no	no	no	no	no	no	no	no
428	no	no	no	no	no	yes	no	no	no	no	no	no	no	no	no
429	no	no	no	no	no	yes	no	no	no	no	no	no	no	no	no
430	no	no	no	no	no	yes	no	no	no	no	no	no	no	no	no
431	no	yes	no	yes	no	no	no	no	no	no	no	no	no	no	no
432	no	no	no	no	no	yes	no	no	no	no	no	no	no	no	no
433	no	no	no	no	no	yes	no	no	no	no	no	no	no	no	no
434	no	no	no	no	no	yes	no	no	no	no	no	no	no	no	no
435	no	no	no	yes	no	yes	no	no	no	no	no	no	no	no	no
436	no	no	no	no	no	no	no	no	no	no	no	no	no	no	no
437	no	no	no	no	no	yes	no	no	no	no	no	no	no	no	no
438	no	no	no	no	no	no	no	no	no	no	no	no	no	no	no
439	no	no	no	no	no	no	no	no	no	no	no	no	no	no	no
440	no	no	no	no	no	no	no	no	no	no	no	no	no	no	no
441	no	yes	no	no	no	no	no	no	no	no	no	no	no	no	no
442	no	no	no	no	no	no	no	no	no	no	no	no	no	no	no
443	no	no	no	no	no	yes	no	no	no	no	no	no	no	no	no
444	no	no	no	no	no	yes	no	no	no	no	no	no	no	no	no
445	no	no	no	no	no	yes	no	no	no	no	no	no	no	no	no
446	no	no	no	no	no	yes	no	no	no	no	no	no	no	no	no
447	no	no	no	yes	no	no	no	no	no	no	no	no	no	no	no

No	**Answer 6a** (yes/no)	**Answer 6b** (yes/no)	**Answer 6c** (yes/no)	**Answer 6d** (yes/no)	**Answer 6e** (yes/no)	**Answer 6f** (yes/no)	**Answer 6g** (yes/no)	**Answer 6h** (yes/no)	**Answer 6i** (yes/no)	**Answer 6j** (yes/no)	**Answer 6k** (yes/no)	**Answer 6l** (yes/no)	**Answer 6m** (yes/no)	**Answer 6n** (yes/no)	**Answer 6o** (yes/no)
448	no	no	no	no	no	no	no	no	no	no	no	no	no	no	no
449	no	no	no	no	no	no	no	no	no	no	no	no	no	no	no
450	no	no	no	no	no	yes	no	no	no	no	no	no	no	no	no
451	no	no	no	no	no	yes	no	no	no	no	no	no	no	no	no
452	no	no	no	no	no	yes	no	no	no	no	no	no	no	no	no
453	no	no	no	no	no	yes	no	no	no	no	no	no	no	no	no
454	no	yes	yes	no	no	yes	no	no	no	no	no	no	no	no	no
455	no	no	no	no	no	yes	no	no	no	no	no	no	no	no	no
456	no	no	no	no	no	yes	no	no	no	no	no	no	no	no	no
457	no	no	no	no	no	yes	no	no	no	no	no	no	no	no	no
458	no	no	no	no	no	no	no	no	no	no	no	no	no	no	no
459	no	no	no	no	no	yes	no	no	no	no	no	no	no	no	no
460	no	no	no	yes	no	no	no	no	no	no	no	no	no	no	no
461	no	no	no	no	no	no	no	no	no	no	no	no	no	no	no
462	yes	no	no	no	yes	no	no	no	no	no	yes	no	yes	no	no
463	no	no	no	no	no	no	no	no	no	no	no	no	no	no	no
464	no	no	no	no	no	no	no	no	no	no	no	no	no	no	no
465	no	yes	no	yes	no	yes	no	no	no	no	yes	no	no	no	no
466	no	yes	no	yes	no	no	no	no	no	no	no	no	no	no	no
467	no	no	no	no	no	no	no	no	no	no	no	no	no	no	no
468	no	no	no	no	no	yes	no	no	no	no	no	no	no	no	no
469	no	no	no	no	no	no	no	no	no	no	yes	no	no	no	no
470	no	no	no	no	no	no	no	no	no	no	no	no	no	no	no
471	no	no	no	no	no	yes	no	no	no	no	no	no	no	no	no
472	no	no	no	no	no	yes	no	no	no	no	no	no	no	no	no

**No**	**Answer 6a** (yes/no)	**Answer 6b** (yes/no)	**Answer 6c** (yes/no)	**Answer 6d** (yes/no)	**Answer 6e** (yes/no)	**Answer 6f** (yes/no)	**Answer 6g** (yes/no)	**Answer 6h** (yes/no)	**Answer 6i** (yes/no)	**Answer 6j** (yes/no)	**Answer 6k** (yes/no)	**Answer 6l** (yes/no)	**Answer 6m** (yes/no)	**Answer 6n** (yes/no)	**Answer 6o** (yes/no)
473	no	no	no	no	no	yes	no	no	no	no	no	no	no	no	no
474	no	no	no	no	no	yes	no	no	no	no	no	no	no	no	yes
475	no	no	no	no	no	yes	no	no	no	no	no	no	no	no	no
476	no	no	no	no	no	yes	no	no	no	yes	no	no	no	no	no
477	no	no	no	no	no	no	no	no	no	no	no	no	no	no	no
478	no	no	no	no	no	yes	yes	yes	no	no	no	no	no	no	no
