# Supplementary material for: Estimation of Prenatal Alcohol Exposure: Comparison of Retrospective Survey and Measurement of Fatty Acid Ethyl Esters, Ethyl Sulfate, and Ethyl Glucuronide Concentrations in Neonatal Meconium
Source: Toxics. 2026 Feb 4;14(2):155. doi: 10.3390/toxics14020155 (PMC12944540; doi:10.3390/toxics14020155)
Supplement: Supplementary file 1 [file toxics-14-00155-s001.zip › Table S04 answers 7-11d.pdf]

Table S4. Results of the survey questions 7 to 11d (n=478) in pregnant women conducted at the Neonatology Clinic of the Medical University of Gdańsk in the Pomeranian Province between June 16, 2019, and April 24, 2020

No	Answer 7 (a-b)	Answer 8 (a-b)	Answer 8 (medicines)	Answer 8b (medicines: yes/no)	Answer 8b (progesterone: yes/no)	Answer 8b (hypertension medicines: yes/no)	Answer 9 (a-b)	Answer 10 (a-b)	Answer 11a (yes/no)	Answer 11b (yes/no)	Answer 11c (yes/no)	Answer 11d (yes/no)
1	b	b	Methyldopa	no	no	yes	b	b	no	no	no	no
2	b	b	Levothyroxine, Heparin, Iron	yes	yes	no	b	b	no	no	no	no
3	b	b	Levothyroxine, Insulin	yes	no	no	a	b	no	no	yes	no
4	a	a		no	no	no	a	b	no	no	no	no
5	b	a	No	no	no	no	a	b	no	no	no	no
6	b	b	Levothyroxine	yes	no	no	b	b	no	no	no	no
7	b	a		no	no	no	a	b	no	no	no	no
8	b	b	Levothyroxine	yes	no	no	a		no	yes	no	no
9	b	b	Levothyroxine, Omeprazole, Cetirizine	yes	no	no	b	b	no	no	no	no
10	a	b	Levothyroxine, Insulin	yes	no	no	a	b	no	no	no	yes
11	b	b	Methyldopa	no	no	yes	b	b	no	no	no	no
12	b	b	Levothyroxine, Insulin	yes	no	no	a	b	no	no	no	yes
13	b	b	Levothyroxine	yes	no	no	b	b	no	no	no	no
14	b	a		no	no	no	b	b	no	no	no	no
15	b	b	n/d	no	no	no			no	no	no	no
16	b	b	Levothyroxine	yes	no	no	a	b	no	no	no	no

No	Answer 7 (a-b)	Answer 8 (a-b)	Answer 8 (medicines)	Answer 8b (medicines: yes/no)	Answer 8b (progesterone: yes/no)	Answer 8b (hypertension medicines: yes/no)	Answer 9 (a-b)	Answer 10 (a-b)	Answer 11a (yes/no)	Answer 11b (yes/no)	Answer 11c (yes/no)	Answer 11d (yes/no)
17	a	b	Levothyroxine, Heparin, Iron	yes	no	no	a	b	no	no	no	yes
18	b	b	Methyldopa	no	no	yes	a	b	no	no	no	no
19	b	a		no	no	no	a	a	no	no	no	no
20	b	a		no	no	no	b	b	no	no	no	no
21	b	a	no	no	no	no			no	no	no	no
22	b	a		no	no	no	a	b	no	no	no	no
23	b	a		yes	no	no	b	b	no	no	no	no
24	b	b	Levothyroxine, Iron	yes	no	no	a	b	no	no	no	no
25	b	a		no	no	no	a	b	no	no	yes	no
26	b	b	Progesterone, Methyldopa	no	yes	no			no	no	no	no
27	a	b	n/d	no	no	no	a	b	no	no	no	no
28	b	b	Insulin	no	no	no	b	b				
29	a	b	Enoxaparin, Iron, Progesterone, Drotaverine	yes	yes	no	a	b	no	no	no	no
30	b	b	Levothyroxine	yes	no	no	b	b	no	no	no	no
31	b	b	n/d	no	no	no			yes	no	yes	no
32	b	a		no	no	no	a	b	no	no	no	no
33	b	b	Levothyroxine	yes	no	no	b	b	no	no	no	no
34	b	b	Progesterone	no	yes	no	b	b	no	no	no	no
35	b	a	Progesterone	no	yes	no			no	no	no	no
36	b	a	Progesterone	no	yes	no			no	no	no	no

No	Answer 7 (a-b)	Answer 8 (a-b)	Answer 8 (medicines)	Answer 8b (medicines: yes/no)	Answer 8b (progesterone: yes/no)	Answer 8b (hypertension medicines: yes/no)	Answer 9 (a-b)	Answer 10 (a-b)	Answer 11a (yes/no)	Answer 11b (yes/no)	Answer 11c (yes/no)	Answer 11d (yes/no)
37	b	b	Levothyroxine	yes	no	no	a	a	no	no	no	no
38	a	b	Levothyroxine	yes	no	no	a	b	no	no	no	no
39	b	a		no	no	no	a	a	no	no	no	no
40	a	a		no	no	no	b	b	no	no	no	no
41	b	a		no	no	no	a	b	no	no	no	no
42	b	b	Methyldopa	no	no	yes	a	b	no	no	no	no
43	b	b	Levothyroxine, Iron	yes	no	no	a	b	no	no	no	no
44	b	b	Levothyroxine	yes	no	no		b	no	no	no	no
45	a	a		no	no	no	b	b	no	no	no	no
46	b	a		no	no	no	a	b	no	no	no	no
47	a	b	Levothyroxine, Olanzapine, Quetiapine	yes	no	no	b	a	no	no	yes	no
48	b	a		no	no	no	a	b	no	no	no	no
49	b	a		yes	no	no	a	b	no	no	no	no
50	b	a		no	no	no	a	b	no	no	no	no
51	b	b	levothyroxine	yes	no	no	b	b	no	no	no	no
52	b	a		yes	no	no	a	b	no	no	no	no
53	b	a		no	no	no	a	b	no	no	no	no
54	b	a		no	no	no	a	b	no	no	no	yes
55	b	a	no	no	no	no	a	b	no	no	no	no
56	b	b	Levothyroxine, Enoxaparin, Methyldopa	yes	no	yes	a	b	no	no	no	yes
57	b	b	Levothyroxine	yes	no	no	b	b	no	no	no	no

No	Answer 7 (a-b)	Answer 8 (a-b)	Answer 8 (medicines)	Answer 8b (medicines: yes/no)	Answer 8b (progesterone: yes/no)	Answer 8b (hypertension medicines: yes/no)	Answer 9 (a-b)	Answer 10 (a-b)	Answer 11a (yes/no)	Answer 11b (yes/no)	Answer 11c (yes/no)	Answer 11d (yes/no)
58	b	b	Progesterone, Drotaverine	no	yes	no	a	b	no	no	no	no
59	b	b	Levothyroxine	yes	no	no	a	b	no	no	no	no
60	b	b	Iron	no	no	no	a	a	no	no	no	no
61	b	b	Venlafaxine	no	no	no	a	b	no	no	no	no
62	b	b	Levothyroxine, Progesterone	yes	yes	no	a	b	no	no	no	
63	b	a		no	no	no	a	b	no	no	no	no
64	b	a		no	no	no	a	b	no	no	no	no
65	a	a	n/d	no	no	no	a	a	no	no	no	no
66	b	a		no	no	no	b	b	no	no	no	no
67	a	b	Enoxaparin	no	no	no	a	b	no	no	no	no
68	b	b	Progesterone	no	yes	no	b	b	no	no	no	no
69	b	a		no	no	no	b	b	no	no	no	no
70	b	a		no	no	no	b	b	no	no	no	no
71	b	b	Levothyroxine	yes	no	no	b	b	no	no	no	no
72	b	b	Levetiracetam, Oxcarbazepine	no	no	no	b	b	no	no	no	no
73	a	b	Methylodopa, Acyclovir, Progesterone	no	yes	yes	a	b	no	no	no	no
74	b	a		no	no	no			no	no	no	no
75	b	a		no	no	no			no	no	no	no
76	a	b	Progesterone	no	yes	no	a	b	no	no	no	no
77	b	b	Levothyroxine	yes	no	no	a	b	no	no	no	no
78	a	b	Levothyroxine	yes	no	no	b	b	no	no	no	no

No	Answer 7 (a-b)	Answer 8 (a-b)	Answer 8 (medicines)	Answer 8b (medicines: yes/no)	Answer 8b (progesterone: yes/no)	Answer 8b (hypertension medicines: yes/no)	Answer 9 (a-b)	Answer 10 (a-b)	Answer 11a (yes/no)	Answer 11b (yes/no)	Answer 11c (yes/no)	Answer 11d (yes/no)
79	b	a	Levothyroxine	yes	no	no	b	b	no	no	no	no
80	b	a		no	no	no	b	b	no	no	no	no
81	b	a		no	no	no	a	b	no	no	no	no
82	a	a		no	no	no	a	b	no	no	no	no
83	b	b	Levothyroxine	yes	no	no	b	b	no	no	no	no
84	a	a		no	no	no	a	b	no	no	no	no
85	b	a		no	no	no	b	b	no	no	no	no
86	b	b	Levothyroxine	yes	no	no	a	b	no	no	no	yes
87	b	a		no	no	no	a	b	no	no	no	no
88	b	a		no	no	no	b	b	no	no	no	no
89	b	b	Levothyroxine	yes	no	no	b	b				
90	b	a	no	no	no	no	a	b	no	no	no	no
91	a	b	Levothyroxine, Iron	yes	no	no	a	b	no	no	no	no
92	a	b	Levothyroxine, Progesterone, Ursodeoxycholic Acid, Zinc Sulfate	yes	yes	no			yes	no	no	yes
93	b	a		no	no	no	a	b	no	no	no	no
94	b	b	Levothyroxine	yes	no	no	a	b	no	no	no	no
95	b	a	Levothyroxine, Methyldopa	yes	no	yes	a	b	no	no	no	no
96	b	b	Levothyroxine	yes	no	no	b	b	no	no	no	no
97	b	b	n/d	no	no	no	a	b	no	no	no	no

No	Answer 7 (a-b)	Answer 8 (a-b)	Answer 8 (medicines)	Answer 8b (medicines: yes/no)	Answer 8b (progesterone: yes/no)	Answer 8b (hypertension medicines: yes/no)	Answer 9 (a-b)	Answer 10 (a-b)	Answer 11a (yes/no)	Answer 11b (yes/no)	Answer 11c (yes/no)	Answer 11d (yes/no)
98	a	b	Levothyroxine, Enoxaparin	yes	no	no	a	a	no	no	yes	no
99	b	b	Levothyroxine	yes	no	no	a	b	no	no	no	
100	b	b	Digoxin, Potassium	no	no	no	b	b	no	no	no	no
101	b	b	Levothyroxine	yes	no	no	a	b	no	no	no	no
102	b	b	Enoxaparin	no	no	no	b	b	no	no	no	no
103	b	b	Corticosteroids	no	no	no	a	a	no	no	no	no
104	a	b	Iron	no	no	no	a	b	no	no	no	no
105	b	b	Iron, Mometasone	no	no	no	b	b	no	no	no	no
106	b	b	Levothyroxine	yes	no	no	a	b	no	no	no	no
107	a	a		no	no	no	b	b	no	no	no	no
108	a	b	Levothyroxine	yes	no	no	b	b	no	no	no	no
109	b	b	0	no	no	no	b	b	no	no	no	no
110	b	b	Levothyroxine	yes	no	no	a	b	no	no	no	no
111	a	b	Enoxaparin, Folic acid	no	no	no	a	b	no	no	no	yes
112	b	a	no	no	no	no	a	b	no	no	no	no
113	b	a		no	no	no	a	b	no	no	no	no
114	a	b	Levothyroxine	yes	no	no	a	b	no	no	no	no
115	b	b	Levothyroxine	yes	no	no	b	b	no	no	no	no
116	b	b	Levothyroxine	yes	no	no	b	b	no	no	no	no
117	b	b	Levothyroxine	yes	no	no	b	b	no	no	no	no
118	b	b	Paracetamol	no	no	no	a	a	no	no	yes	no
119	b	a		no	no	no	a	b	no	no	no	no

No	Answer 7 (a-b)	Answer 8 (a-b)	Answer 8 (medicines)	Answer 8b (medicines: yes/no)	Answer 8b (progesterone: yes/no)	Answer 8b (hypertension medicines: yes/no)	Answer 9 (a-b)	Answer 10 (a-b)	Answer 11a (yes/no)	Answer 11b (yes/no)	Answer 11c (yes/no)	Answer 11d (yes/no)
120	b	a		no	no	no	a	b	no	no	no	no
121	b	b	Levothyroxine, Enoxaparin	yes	no	no	a	b	no	no	no	no
122	b	b	Levothyroxine	yes	no	no	a	b	no	no	no	yes
123	b	a	No	no	no	no	b	b	no	no	no	no
124	b	b	Levothyroxine	yes	no	no	b	b	no	no	no	no
125	b	a		no	no	no	b	b	no	no	no	no
126	b	a	Progesterone	no	yes	no			no	no	no	no
127	b	b	Levothyroxine, Insulin	yes	no	no	b	b	no	no	no	no
128	b	b	Levothyroxine, Iron, Progesterone	yes	yes	no	a	b	no	no	no	no
129	b	b	Rovamycin	no	no	no	a	b	no	no	no	no
130	b	a		no	no	no	a	b	no	no	no	no
131	b	b	Levothyroxine	yes	no	no	b	b	yes	no	no	no
132	b	b	Enoxaparin	no	no	no	a	b	no	no	no	no
133	b	b	Levothyroxine	yes	no	no			no	no	no	no
134	b	b	Methyldopa, Calcium Channel Blocker, Insulin, Diazepam	no	no	yes	a	b	no	no	no	yes
135	b	a		no	no	no	a	b	no	no	no	no

No	Answer 7 (a-b)	Answer 8 (a-b)	Answer 8 (medicines)	Answer 8b (medicines: yes/no)	Answer 8b (progesterone: yes/no)	Answer 8b (hypertension medicines: yes/no)	Answer 9 (a-b)	Answer 10 (a-b)	Answer 11a (yes/no)	Answer 11b (yes/no)	Answer 11c (yes/no)	Answer 11d (yes/no)
136	b	b	Acetylsalicylic acid, Metformin, Enoxaparin	no	no	no	a		yes	yes	no	yes
137	b	a		no	no	no	a	b	no	no	no	no
138	b	a		no	no	no	b	b	no	no	no	no
139	b	b	Levothyroxine	yes	no	no	b	b	no	no	no	no
140	b	a	no	no	no	no	b	b	no	no	no	no
141	b	a		no	no	no	a	a	no	no	no	no
142	a	a		no	no	no	a	b	no	no	no	no
143	a	b	Levothyroxine	yes	no	no	a	b	no	no	no	no
144	b	b	'after in vitro'	no	no	no	a	b	no	no	no	no
145	b	a		no	no	no	b	b	yes	no	no	no
146	b	b	Levothyroxine	yes	no	no	b	b	no	no	yes	no
147	b	a		no	no	no	b	b	no	no	no	no
148	b	b	Levothyroxine	yes	no	no	a	b	no	no	no	no
149	a	a		no	no	no	a	b	no	no	no	no
150	a	b	Methyldopa	no	no	yes	a	a	no	no	no	no
151	b	a		no	no	no	a	b	no	no	no	no
152	b	b	Levothyroxine	yes	no	no	a	b	no	no	no	no
153	b	b	Acetylsalicylic Acid, Iron	no	no	no	b	b	no	no	no	no
154	b	b	Progesterone	no	yes	no	b	b	no	no	no	no
155	b	a		no	no	no	a	b	no	no	no	no
156	b	b	Levothyroxine	yes	no	no	b	b	no	no	no	no

No	Answer 7 (a-b)	Answer 8 (a-b)	Answer 8 (medicines)	Answer 8b (medicines: yes/no)	Answer 8b (progesterone: yes/no)	Answer 8b (hypertension medicines: yes/no)	Answer 9 (a-b)	Answer 10 (a-b)	Answer 11a (yes/no)	Answer 11b (yes/no)	Answer 11c (yes/no)	Answer 11d (yes/no)
157	b	a		no	no	no	b	b	no	no	no	no
158	b	b	Iron	no	no	no	b	b	no	no	no	no
159	a	b	Levothyroxine, Iron	yes	no	no	b	b	no	no	no	no
160	b	b	Levothyroxine	yes	no	no	b	b	no	no	no	no
161	b	b	Levothyroxine, Iron	yes	no	no	b	b	no	no	no	no
162	b	a	Levothyroxine	yes	no	no	a	b	no	no	no	no
163	a	a	Progesterone	no	no	no	b	b	no	no	no	no
164	b	a		no	no	no	b	b	no	no	no	no
165	a	a	Progesterone	no	no	no	b	b	no	no	no	no
166	a	a	Levothyroxine	yes	no	no	a	a	no	no	no	no
167	b	a		no	no	no	b		no	no	no	no
168	b	b	Acetylsalicylic acid	no	no	no	b	b	no	no	no	no
169	b	a		no	no	no	b	b	no	no	no	no
170	a	b	Acetylsalicylic acid, Heparin	no	no	no			no	no	no	no
171	a	b	Acetylsalicylic acid, Heparin	no	no	no			no	no	no	no
172	b	a		no	no	no	b	b	no	no	no	no
173	a	b	Bisoprolol	no	no	yes	a	b	no	no	no	yes
174	b	a		no	no	no	a	b	no	no	no	no
175	b	a		no	no	no	a	b	no	no	no	no
176	a	b	Levothyroxine	yes	no	no	a	b	no	no	no	no

No	Answer 7 (a-b)	Answer 8 (a-b)	Answer 8 (medicines)	Answer 8b (medicines: yes/no)	Answer 8b (progesterone: yes/no)	Answer 8b (hypertension medicines: yes/no)	Answer 9 (a-b)	Answer 10 (a-b)	Answer 11a (yes/no)	Answer 11b (yes/no)	Answer 11c (yes/no)	Answer 11d (yes/no)
177	b	b	Levothyroxine, Heparin	yes	no	no	a	b	no	no	no	no
178	b	b	Insulin	no	no	no			yes	no	no	yes
179	b	b	Calcium channel blocker, Insulin, Enoxaparin	no	no	no	b	b	no	no	no	yes
180	b	b	n/d	no	no	no			no	no	no	no
181	b	b	No	no	no	no	a	b	no	no	no	no
182	b	a	No	no	no	no	a	b	no	no	no	no
183	b	b	Insulin, Iron	no	no	no	b	b	no	no	no	yes
184	b	b	Progesterone	no	yes	no	b	b	no	no	no	yes
185	b	b	Progesterone	no	yes	no	b	b	no	no	no	yes
186	b	b	no	no	no	no	b	b	no	no	no	no
187	a	a		no	no	no	a	b	no	no	no	no
188	b	b	Levothyroxine	yes	no	no			no	no	no	no
189	a	a	no	no	no	no	a	b	no	no	no	no
190	b	a	no	no	no	no	b	b	no	no	no	no
191	a	a	no	no	no	no	a	b	no	no	no	no
192	b	b	no	no	no	no	b	b	no	no	no	no
193	b	b	Levothyroxine	yes	no	no	b	b	no	no	no	no
194	b	b	Levothyroxine	yes	no	no			no	no	no	no
195	b	a	no	no	no	no	a	b	no	no	no	no
196	b	a		no	no	no	a	b	no	no	no	no

No	Answer 7 (a-b)	Answer 8 (a-b)	Answer 8 (medicines)	Answer 8b (medicines: yes/no)	Answer 8b (progesterone: yes/no)	Answer 8b (hypertension medicines: yes/no)	Answer 9 (a-b)	Answer 10 (a-b)	Answer 11a (yes/no)	Answer 11b (yes/no)	Answer 11c (yes/no)	Answer 11d (yes/no)
197	b	b	Levothyroxine, Iron, Drotaverine	yes	no	no	a	b	no	no	no	no
198	b	a		no	no	no	a	b	yes	no	no	no
199	b	a		no	no	no	b	b	no	no	no	no
200	a	a		no	no	no	a	b	yes	no	no	no
201	b	b	Iron	no	no	no	a	b	no	no	no	no
202	b	b	Levothyroxine	yes	no	no	a	b	no	no	no	no
203	b	b	Levothyroxine, Methyldopa, Beta Blocker, Enoxaparin, Prednisone	yes	no	yes			no	yes	no	no
204	a	b	Iron	no	no	no	a	b	no	no	no	no
205	a	b	Iron	no	no	no	a	b	no	no	no	no
206	b	b	Levothyroxine	yes	no	no	b	b	no	no	no	no
207	b	a	no	no	no	no	b	b	no	no	no	no
208	b	a		no	no	no	a	b	no	no	no	no
209	b	a		no	no	no	b	b	no	no	no	no
210	a	a		no	no	no	b	b	no	no	no	no
211	b	a		0	0	0	0	0	0	0	0	0
212	b	a	Progesterone, Iron	no	yes	no	b	b	no	no	no	no
213	b	a		no	no	no	b	b	no	no	no	yes
214	a	b	Levothyroxine	yes	no	no			no	no	no	no
215	b	a		no	no	no	b	b	no	no	no	no

No	Answer 7 (a-b)	Answer 8 (a-b)	Answer 8 (medicines)	Answer 8b (medicines: yes/no)	Answer 8b (progesterone: yes/no)	Answer 8b (hypertension medicines: yes/no)	Answer 9 (a-b)	Answer 10 (a-b)	Answer 11a (yes/no)	Answer 11b (yes/no)	Answer 11c (yes/no)	Answer 11d (yes/no)
216	b	b	Levothyroxine, Insulin	yes	no	no	b	b	no	no	no	no
217	a	b	Levothyroxine Acetylsalicylic acid, Heparin	yes	no	no	a	b	no	no	no	yes
218	b	b	Levothyroxine	yes	no	no	a	b	yes	yes	no	no
219	b	b	Levothyroxine	yes	no	no	a	b	no	no	no	no
220	a	b	Insulin	no	no	no			yes	no	no	yes
221	b	b	Progesterone	no	yes	no	a	b	no	no	no	no
222	a	b	Insulin	no	no	no			yes	no	no	yes
223	b	a		no	no	no	a	b	no	no	no	yes
224	b	b	Levothyroxine	yes	no	no	b	b	no	no	no	no
225	b	b	Levothyroxine, Iron, Heparin	yes	no	no	b	a	no	no	yes	yes
226	b	a		no	no	no	a	b	no	no	no	no
227	b	b	Levothyroxine, Insulin, Heparin	yes	no	no	a	a	no	no	no	yes
228	b	b	Levothyroxine	yes	no	no	a	b	no	no	no	no
229	b	a		no	no	no	b	b	no	no	no	no
230	b	b	Levothyroxine	yes	no	no	b	b	no	no	no	yes
231	b	a	Progesterone, Drotaverine	no	no	no	b	b	yes	no	no	no
232	b	a		no	no	no	a	b	no	no	no	no
233	b	b	Levothyroxine	yes	no	no			no	no	no	no
234	b	a		no	no	no	b	a	no	no	no	no

No	Answer 7 (a-b)	Answer 8 (a-b)	Answer 8 (medicines)	Answer 8b (medicines: yes/no)	Answer 8b (progesterone: yes/no)	Answer 8b (hypertension medicines: yes/no)	Answer 9 (a-b)	Answer 10 (a-b)	Answer 11a (yes/no)	Answer 11b (yes/no)	Answer 11c (yes/no)	Answer 11d (yes/no)
235	b	a		no	no	no	a	b	no	no	no	no
236	b	a		no	no	no			no	no	no	no
237	b	b	Levothyroxine	yes	no	no	a	a	no	no	no	no
238	b	b	Levocetirizine	no	no	no	a	b	no	no	no	no
239	b	b	Rovamycin	no	no	no	a	a	no	no	yes	no
240	b	b	Progesterone, Drotaverine	no	yes	no	a	b	no	no	no	no
241	a	a		no	no	no	b	b	no	no	no	no
242	b	b	Levothyroxine	yes	no	no	a	b	no	no	no	no
243	b	a		no	no	no	a	b	no	no	no	no
244	b	b	Levothyroxine	yes	no	no	b	a	no	no	no	no
245	b	a		no	no	no	b	b	no	no	no	no
246	b	b	Insulin, Levothyroxine	yes	no	no	a	b	no	no	no	yes
247	a	b	Levothyroxine	yes	no	no	a	b	no	no	no	yes
248	b	b	Insulin	no	no	no	b	b	no	no	no	no
249	b	b	Levothyroxine	yes	no	no	a	b	no	no	no	yes
250	a	b	Insulin	no	no	no	a	b	no	no	no	yes
251	b	b	Levothyroxine	yes	no	no	a	b	no	no	no	no
252	b	b	dietary supplement	no	no	no	a	b	no	no	no	no
253	b	a		no	no	no	a	b	no	no	no	no
254	b	b	Levothyroxine	yes	no	no	a	b	no	no	no	no
255	b	a		no	no	no	b	b	no	no	no	no
256	b	a		no	no	no	a	b	no	no	no	no

No	Answer 7 (a-b)	Answer 8 (a-b)	Answer 8 (medicines)	Answer 8b (medicines: yes/no)	Answer 8b (progesterone: yes/no)	Answer 8b (hypertension medicines: yes/no)	Answer 9 (a-b)	Answer 10 (a-b)	Answer 11a (yes/no)	Answer 11b (yes/no)	Answer 11c (yes/no)	Answer 11d (yes/no)
257	b	a		no	no	no	a	b	no	no	no	yes
258	b	a		no	no	no			no	no	no	no
259	b	b	Levothyroxine, Insulin	yes	no	yes	a	b	no	no	no	yes
260	b	b	Levothyroxine	yes	no	no	b	b	no	no	no	yes
261	b	a		no	no	no	b	b	yes	yes	yes	no
262	b	b	Levothyroxine, Methyldopa	yes	no	yes			no	no	no	no
263	a	b	Levothyroxine, Heparin	yes	no	no	a	b	no	no	no	no
264	a	b	Levothyroxine, Heparin	yes	no	no	a	b	no	no	no	no
265	b	a		no	no	no	b	b	no	no	no	no
266	b	a		no	no	no	b	b	no	no	no	yes
267	b	b	Levothyroxine, Methyldopa, Metformin, Progesterone	yes	yes	yes	a	a	no	no	no	no
268	b	b	Iron	no	no	no	a	b	no	no	no	no
269	b	b	Iron	no	no	no	a	b	no	no	no	no
270	a	b	Levothyroxine	yes	no	no	a	b	no	no	no	no
271	b	b	Levothyroxine	yes	no	no	a	b	no	no	no	no
272	a	a		no	no	no	a	b	yes	yes	no	no
273	b	b	Levothyroxine, Progesterone	yes	yes	yes	b	b	no	no	no	no
274	b	b	Methyldopa	no	no	yes			no	no	no	no

No	Answer 7 (a-b)	Answer 8 (a-b)	Answer 8 (medicines)	Answer 8b (medicines: yes/no)	Answer 8b (progesterone: yes/no)	Answer 8b (hypertension medicines: yes/no)	Answer 9 (a-b)	Answer 10 (a-b)	Answer 11a (yes/no)	Answer 11b (yes/no)	Answer 11c (yes/no)	Answer 11d (yes/no)
275	b	b	Levothyroxine, Iron	yes	no	no			no	no	no	no
276	b	b	Progesterone, Drotaverine	no	yes	no	b	b	no	no	no	no
277	a	a		no	no	no	b	b	no	no	no	no
278	b	a		no	no	no	a	b	no	no	no	no
279	b	a		no	no	no	b	b	no	no	yes	no
280	b	b	Levothyroxine	yes	no	no	b	b	yes	no	no	no
281	b	a		no	no	no	b	a	no	no	no	no
282	b	b	Iron	no	no	no	b	b	no	no	no	yes
283	b	b	Levothyroxine, Cabergoline	yes	no	no	b	b	no	no	no	no
284	b	a		no	no	no	a	b	no	no	no	no
285	b	b	Iron, Vitamin B12	no	no	no	a	b	no	no	yes	no
286	b	b	Levothyroxine	yes	no	no	a	a	no	no	no	no
287	b	b	Levothyroxine	yes	no	no			no	no	no	no
288	b	b	Levothyroxine	yes	no	no			no	no	no	no
289	a	a		no	no	no	a	a	no	no	no	no
290	b	b	Insulin	no	no	no	a	b	no	no	no	no
291	b	a		no	no	no	a	b	no	no	no	no
292	a	b	Levothyroxine	yes	no	no	a	b	no	no	no	no
293	b	b	Levothyroxine	yes	no	no	b	b	no	no	no	no
294	b	b	Levothyroxine	yes	no	no	b	b	no	no	no	no
295	b	a		no	no	no	b	b	no	no	no	no

No	Answer 7 (a-b)	Answer 8 (a-b)	Answer 8 (medicines)	Answer 8b (medicines: yes/no)	Answer 8b (progesterone: yes/no)	Answer 8b (hypertension medicines: yes/no)	Answer 9 (a-b)	Answer 10 (a-b)	Answer 11a (yes/no)	Answer 11b (yes/no)	Answer 11c (yes/no)	Answer 11d (yes/no)
296	a	b	Acetylsalicylic acid, Progesterone	no	yes	no	b	b	no	no	no	no
297	b	b	Iron	no	no	no	b	b	no	no	no	no
298	a	b	Sertraline, Lorazepam, Chloroprothixene, Levothyroxine	yes	no	no	a	b	no	no	no	no
299	b	b	Levothyroxine	yes	no	no	b	b	no	no	no	no
300	b	a		no	no	no	a	a	no	no	no	no
301	b	a		no	no	no	a	b	no	no	no	no
302	a	b	Progesterone	no	yes	no			no	no	no	no
303	b	b	Methyldopa	no	no	yes	b	b	no	no	no	no
304	b	b	Methyldopa	no	no	yes	b	b	no	no	no	no
305	b	a		no	no	no	b	a	no	no	no	no
306	b	a		no	no	no	a	b	no	no	no	no
307	b	b	Levothyroxine	yes	no	no	b	b	no	no	no	no
308	b	a		no	no	no	a	b	no	no	no	no
309	a	b	Progesterone	no	yes	no	b	b	no	no	yes	yes
310	b	b	Levothyroxine	yes	no	no	a	b	no	no	no	no
311	b	b	Lewotyroksyn, Iron	yes	no	no	b	b	no	no	no	no
312	b	b	Levothyroxine	yes	no	no			no	no	no	no
313	b	b	Mesalazine	no	no	no	a	b	no	no	no	no
314	a	a		no	no	no	b	b	no	no	yes	no

No	Answer 7 (a-b)	Answer 8 (a-b)	Answer 8 (medicines)	Answer 8b (medicines: yes/no)	Answer 8b (progesterone: yes/no)	Answer 8b (hypertension medicines: yes/no)	Answer 9 (a-b)	Answer 10 (a-b)	Answer 11a (yes/no)	Answer 11b (yes/no)	Answer 11c (yes/no)	Answer 11d (yes/no)
315	b	a	no	no	no	no	b	b	no	no	no	no
316	b	b	Levothyroxine	yes	no	no	b	b	no	no	no	yes
317	a	b	Progesterone, Drotaverine	no	yes	no	b	b	no	no	no	no
318	b	b	Levothyroxine, Progesterone	yes	yes	no			yes	no	no	yes
319	n/d	n/d	n/d	n/d	0	0	n/d	n/d	0	0	0	0
320	n/d	n/d	n/d	n/d	0	0		n/d	0	0	0	0
321	b	b	Methyldopa, Acetylsalicylic acid	no	no	yes	a	a		no	no	no
322	b	a		no	no	no	b	b	yes	no	no	no
323	b	b	Levothyroxine	yes	no	no	a	b	no	no	no	yes
324	b	b	Levothyroxine	yes	no	no	a	b	no	no	no	no
325	a	a		no	no	no	b	b	no	no	no	no
326	b	a		no	no	no	b	b	no	no	no	no
327	b	a		no	no	no	b	b	no	no	no	no
328	b	b	Levothyroxine, Progesterone	yes	yes	no	a	b	no	no	no	no
329	b	a		no	no	no	b	b	no	no	no	no
330	b	b	Levothyroxine	yes	no	no	b	b	no	no	no	no
331	b	b	Levothyroxine	yes	no	no	b	b	no	no	no	no
332	b	b	Levothyroxine, Insulin	yes	no	no			yes	no	no	yes
333	b	a		no	no	no	b	b	no	no	no	no

No	Answer 7 (a-b)	Answer 8 (a-b)	Answer 8 (medicines)	Answer 8b (medicines: yes/no)	Answer 8b (progesterone: yes/no)	Answer 8b (hypertension medicines: yes/no)	Answer 9 (a-b)	Answer 10 (a-b)	Answer 11a (yes/no)	Answer 11b (yes/no)	Answer 11c (yes/no)	Answer 11d (yes/no)
334	a	b	Calcium channel blocker, Heparin, Drotaverine	no	no	no			yes	no	no	yes
335	b	b	Levothyroxine	yes	no	no	a	b	no	no	yes	no
336	b	b	Heparin, Progesterone	no	yes	no	a	b	no	no	no	no
337	b	a	Levothyroxine	yes	no	no						
338	b	a		no	no	no	a	a	no	no	no	no
339	b	b	Levothyroxine	yes	no	no	a	b	no	no	no	no
340	b	a		no	no	no	a	b	no	no	no	no
341	b	a		no	no	no	a	b	no	no	no	no
342	a	a		no	no	no	a	b	no	no	no	no
343	a	b	Heparin	no	no	no			no	no	no	no
344	b	a		no	no	no	b	b	no	no	no	no
345	b	a		no	no	no	a	b	no	no	no	no
346	b	a		no	no	no	a	b	no	no	no	no
347	b	b	Methyldopa, Calcium channel blocker, Beta blocker, Levothyroxine, Insulin	yes	no	yes			yes	no	no	yes
348	b	b	Levothyroxine	yes	no	no	a	b	no	no	no	no

No	Answer 7 (a-b)	Answer 8 (a-b)	Answer 8 (medicines)	Answer 8b (medicines: yes/no)	Answer 8b (progesterone: yes/no)	Answer 8b (hypertension medicines: yes/no)	Answer 9 (a-b)	Answer 10 (a-b)	Answer 11a (yes/no)	Answer 11b (yes/no)	Answer 11c (yes/no)	Answer 11d (yes/no)
349	a	b	Levothyroxine, Heparin	yes	no	no	a	b	no	no	no	no
350	b	a		no	no	no	b	b	no	no	no	no
351	b	b	Progesterone	no	yes	no			no	no	no	no
352	b	b	Levothyroxine	yes	no	no	a	b	no	no	no	no
353	b	a		no	no	no	a	b	no	no	no	no
354	b	b	Iron	no	no	no	a	a	no	no	no	no
355	a	a		no	no	no	b	b	no	no	no	no
356	b	a		no	no	no	a	b	no	no	no	no
357	b	b	Progesterone, Drotaverine, Iron	no	yes	no	a	b	no	no	no	no
358	b	b	Progesterone, Drotaverine, Iron	no	yes	no	a	b	no	no	no	no
359	b	b	Levothyroxine, Insulin	yes	no	no	a	b	no	no	no	no
360	a	b	Levothyroxine, Progesterone, Corticosteroids	yes	yes	no			no	no	no	no
361	a	b	Levothyroxine	yes	no	no	b	b	no	no	no	no
362	b	a		no	no	no	b	b	no	no	no	no
363	b	a		yes	no	no	a	b	no	no	no	no
364	b	b	Methyldopa	no	no	yes	a	b	no	no	no	no
365	b	a		no	no	no	a	a	yes	no	yes	no
366	b	a	Iron	no	no	no	b	b	no	no	no	no

No	Answer 7 (a-b)	Answer 8 (a-b)	Answer 8 (medicines)	Answer 8b (medicines: yes/no)	Answer 8b (progesterone: yes/no)	Answer 8b (hypertension medicines: yes/no)	Answer 9 (a-b)	Answer 10 (a-b)	Answer 11a (yes/no)	Answer 11b (yes/no)	Answer 11c (yes/no)	Answer 11d (yes/no)
367	b	a	Progesterone, Drotaverine	no	no	no	b	b	no	no	no	no
368	b	b	Levothyroxine	yes	no	no	b	b	no	no	no	yes
369	b	a		no	no	no	a	b	no	no	no	no
370	a	b	Heparin	no	no	no	b	b	no	no	no	no
371	b	b	Levothyroxine, Insulin	yes	no	no	a	b	no	no	no	no
372	b	b	Levothyroxine	yes	no	no	a	b	yes	yes	no	no
373	a	a		no	no	no	a	b	no	no	no	no
374	b	b	Levothyroxine	yes	no	no	b	b	no	no	no	no
375	b	b	Levothyroxine	yes	no	no	a	b	no	no	yes	no
376	b	b	Levothyroxine	yes	no	no	b	b	no	no	no	yes
377	a	b	Iron	no	no	no	b	b	no	no	no	no
378	b	b	Levothyroxine	yes	no	no	b	b	no	no	no	no
379	a	b	Levothyroxine	yes	no	no	a	b	no	no	no	no
380	b	b	Calcium channel blocker (Amlodipine), Methyldopa		no	yes	a	b	no	no	no	yes
381	a	a		no	no	no	a	a	no	no	no	no
382	a	b	Levothyroxine	yes	no	no	b	b	no	yes	no	no
383	b	a	Progesterone, Drotaverine, Magnesium	no	yes	no	b	b	no	no	no	no
384	a	b	Levothyroxine, Progesterone	yes	yes	no	a	b	no	no	no	no

No	Answer 7 (a-b)	Answer 8 (a-b)	Answer 8 (medicines)	Answer 8b (medicines: yes/no)	Answer 8b (progesterone: yes/no)	Answer 8b (hypertension medicines: yes/no)	Answer 9 (a-b)	Answer 10 (a-b)	Answer 11a (yes/no)	Answer 11b (yes/no)	Answer 11c (yes/no)	Answer 11d (yes/no)
385	b	a		no	no	no	b	b	no	no	no	no
386	b	b	Insulin	no	no	no	b	a	no	no	no	no
387	a	a		no	no	no	a	b	no	no	no	no
388	b	b	Methyldopa, Progesterone	no	yes	yes	b	b	no	no	no	no
389	a	b	Iron	no	no	no	b	b	no	no	no	no
390	b	b	Levothyroxine	yes	no	no	b	b	no	no	no	no
391	b	b	Progesterone, Estradiol, Heparin, Iron, Folic acid, Vitamin D3	no	yes	no	a	b	no	no	no	no
392	b	b	Levothyroxine, Heparin	yes	no	no	a	b	no	no	no	no
393	a	a		no	no	no	a	b	no	no	no	no
394	b	a		0	0	0	b	b	no	no	no	no
395	b	b	Levothyroxine, Acetylsalicylic acid	yes	no	no	a	b	no	no	no	yes
396	b	b	Levothyroxine	yes	no	yes	b	b	no	yes	no	no
397	b	b	Levothyroxine, Iron	yes	no	yes	a	b	no	no	no	no
398	a	b	Levothyroxine	yes	no	no	a	a	no	no	no	no
399	b	a	Progesterone, Drotaverine	no	no	no	a	b	no	no	no	no

No	Answer 7 (a-b)	Answer 8 (a-b)	Answer 8 (medicines)	Answer 8b (medicines: yes/no)	Answer 8b (progesterone: yes/no)	Answer 8b (hypertension medicines: yes/no)	Answer 9 (a-b)	Answer 10 (a-b)	Answer 11a (yes/no)	Answer 11b (yes/no)	Answer 11c (yes/no)	Answer 11d (yes/no)
400	b	b	Levothyroxine, Lutein	yes	yes	no	a	b	no	no	no	yes
401	a	a		no	no	no	b	b	no	no	no	no
402	b	a		no	no	no	b	a	no	no	no	yes
403	b	b	Levothyroxine	yes	no	no	a	b	no	no	no	no
404	b	b	Progesterone, Drotaverine, Magnesium	no	no	no	a	b	no	no	no	no
405	a	b	n/d	no	no	no	b	a	no	yes	no	no
406	b	a		no	no	no	b	a	no	no	no	yes
407	b	a		no	no	no	a	b	no	no	no	no
408	b	b	Levothyroxine	yes	no	no	a	b	no	no	no	no
409	b	b	Levothyroxine	yes	no	yes	b	b	no	no	no	no
410	a	a	Methyldopa	no	no	yes	a	b	no	no	no	no
411	a	b	Acetylsalicylic acid	no	no	no	a	b	yes	yes	no	no
412	a	b	n/d	no	no	no	b	b	no	no	no	no
413	b	a		no	no	no	b	b	no	no	no	no
414	a	b	Enoxaparin	no	no	no	b	b	no	no	no	no
415	b	b	Levothyroxine, Progesterone, Methyldopa	yes	yes	yes			no	no	no	no
416	b	a		yes	no	yes	b	b	no	no	no	no
417	b	a		no	no	no	b	b	no	no	no	no
418	b	a		no	no	no	b	b	no	yes	no	no

No	Answer 7 (a-b)	Answer 8 (a-b)	Answer 8 (medicines)	Answer 8b (medicines: yes/no)	Answer 8b (progesterone: yes/no)	Answer 8b (hypertension medicines: yes/no)	Answer 9 (a-b)	Answer 10 (a-b)	Answer 11a (yes/no)	Answer 11b (yes/no)	Answer 11c (yes/no)	Answer 11d (yes/no)
419	b	b	Levothyroxine, Enoxaparin, Acetylsalicylic acid	yes	no	yes	a	a	no	no	no	yes
420	b	a	no	no	no	no	b	b	no	no	no	no
421	b	a	no	no	no	no	b	b	no	no	no	no
422	b	a		no	no	no	a	b	no	no	no	no
423	b	b	Levothyroxine	yes	no	no	b	b	no	no	no	no
424	b	b	Levothyroxine	yes	no	no	b	b	no	no	no	no
425	b	b	Levothyroxine	yes	no	no	b	b	no	no	no	no
426	b	a		no	no	no	a	a	no	no	no	no
427	b	a		no	no	no	a	a	no	no	no	no
428	b	b	Levothyroxine	yes	no	no	b	b	no	no	no	no
429	b	b	Levothyroxine, Ursodeoxycholic acid	yes	no	no	a	b	no	no	no	no
430	a	b	Levothyroxine	yes	no	no	b	b	no	no	no	no
431	b	a		no	no	no	a	b	no	no	no	no
432	b	b	Levothyroxine	yes	no	no	b	b	no	no	no	no
433	b	b	Levothyroxine	yes	no	no	b	b	no	no	no	no
434	b	b	Levothyroxine	yes	no	no	a	b	no	no	no	no
435	a	b	Levothyroxine	yes	no	no	b	b	no	no	no	yes
436	b	a	no	no	no	no	b	b	no	no	no	no
437	b	b	Levothyroxine, Acetylsalicylic acid	yes	no	no	b	b	no	no	no	no

No	Answer 7 (a-b)	Answer 8 (a-b)	Answer 8 (medicines)	Answer 8b (medicines: yes/no)	Answer 8b (progesterone: yes/no)	Answer 8b (hypertension medicines: yes/no)	Answer 9 (a-b)	Answer 10 (a-b)	Answer 11a (yes/no)	Answer 11b (yes/no)	Answer 11c (yes/no)	Answer 11d (yes/no)
438	b	b	Progesterone, Drotaverine	no	yes	no	a	b	no	no	no	no
439	b	a		no	no	no	a	b	no	no	no	no
440	b	a		no	no	no	a	b	no	no	no	no
441	a	b	Progesterone, Drotaverine, Methyldopa	no	yes	yes	b	b	no	no	no	no
442	b	a	Progesterone	no	no	no	b	a	no	no	no	no
443	b	b	Levothyroxine, Heparin	yes	no	no	a	b	no	no	no	no
444	b	b	Levothyroxine, Progesterone	yes	yes	no	a	b	no	no	no	no
445	b	b	Levothyroxine, Progesterone	yes	yes	no	a	b	no	no	no	no
446	b	b	Levothyroxine, Acetylsalicylic acid	yes	no	no	a	b	no	no	no	no
447	b	a		no	no	no	b	b	no	no	no	no
448	b	a		no	no	no	b	b	no	no	no	no
449	b	b	Progesterone	no	yes	no	a	b	yes	no	no	no
450	b	b	Levothyroxine	yes	no	no	a	b	no	no	no	no
451	b	b	Levothyroxine	yes	no	no	a	b	no	no	no	no
452	a	b	Levothyroxine	yes	no	no	a	b	no	no	no	no
453	a	b	Levothyroxine	yes	no	no	b	b	no	no	no	no
454	a	b	Levothyroxine, Methyldopa,	yes	no	yes	a	b	no	no	no	yes

No	Answer 7 (a-b)	Answer 8 (a-b)	Answer 8 (medicines)	Answer 8b (medicines: yes/no)	Answer 8b (progesterone: yes/no)	Answer 8b (hypertension medicines: yes/no)	Answer 9 (a-b)	Answer 10 (a-b)	Answer 11a (yes/no)	Answer 11b (yes/no)	Answer 11c (yes/no)	Answer 11d (yes/no)
			Beta blocker, Insulin									
455	b	b	Levothyroxine	yes	no	no	a	b	no	no	no	no
456	b	b	Levothyroxine	yes	no	no	b	a	no	no	no	no
457	a	b	Levothyroxine, Metyhlpredniso lone, Insulin, Acetylsalicylic acid, Lutein	yes	yes	no	b	b	no	no	no	no
458	b	b	Iron	no	no	no	b	b	no	no	no	no
459	b	b	Levothyroxine	yes	no	no	a	b	no	no	no	no
460	b	a	no	no	no	no	b	b	no	no	no	no
461	b	a	no	no	no	no	b	b	no	no	no	no
462	a	b	Methyldopa, Allopurinol, Alfacalcidol, Beta blocker	no	no	yes	a	b	yes	yes	no	no
463	b	a	no	no	no	no	b	b	no	no	no	no
464	b	a	No	no	no	no	b	b	no	no	no	no
465	b	b	Levothyroxine, Insulin	yes	no	no	b	b	no	no	no	yes
466	b	b	Methyldopa, Insulin	no	no	yes	a	b	no	no	no	yes
467	b	b	Progesterone	no	yes	no	b	b	no	no	no	no
468	b	b	Levothyroxine	yes	no	no	b	b	no	no	no	no
469	a	b	Iron	no	no	no	b	a	no	no	no	no

No	Answer 7 (a-b)	Answer 8 (a-b)	Answer 8 (medicines)	Answer 8b (medicines: yes/no)	Answer 8b (progesterone: yes/no)	Answer 8b (hypertension medicines: yes/no)	Answer 9 (a-b)	Answer 10 (a-b)	Answer 11a (yes/no)	Answer 11b (yes/no)	Answer 11c (yes/no)	Answer 11d (yes/no)
470	a	b	Lutein	no	yes	no	b	b	no	no	no	no
471	b	b	Levothyroxine	yes	no	no	b	b	no	no	no	no
472	b	b	Levothyroxine	yes	no	no	a	a	no	no	no	no
473	b	b	Levothyroxine	yes	no	no	a	a	no	no	no	no
474	b	b	Levothyroxine, Metformin	yes	no	no	b	b	no	no	no	no
475	a	b	Levothyroxine	yes	no	no	a	b	no	no	no	no
476	b	b	Levothyroxine	yes	no	no	a	b	yes	no	no	no
477	b	a		0	0	0			no	no	no	no
478	a	b	Levothyroxine, Budesonide, Formoterol, Salbutamol	yes	no	no	b	a	no	no	no	no
