# Supplementary material for: Estimation of Prenatal Alcohol Exposure: Comparison of Retrospective Survey and Measurement of Fatty Acid Ethyl Esters, Ethyl Sulfate, and Ethyl Glucuronide Concentrations in Neonatal Meconium
Source: Toxics. 2026 Feb 4;14(2):155. doi: 10.3390/toxics14020155 (PMC12944540; doi:10.3390/toxics14020155)
Supplement: Supplementary file 1 [file toxics-14-00155-s001.zip › Table S05 answers 12-18.pdf]

Table S5. Results of survey questions 12 to 18 (n=478) in pregnant women conducted at the Neonatology Clinic of the Medical University of Gdańsk in the Pomeranian Province between June 16, 2019, and April 24, 2020.

No	Answer 12a (yes/no)	Answer 12b (yes/no)	Answer 12c (yes/no)	Answer 12d (yes/no)	Answer 12e (yes/no)	Answer 12f (yes/no)	Answer 13 (a-d)	Answer 14 (a-e)	Answer 15 (a-c)	Answer 16 (a-b)	Answer 17 (a-b)	Answer 18 (a-d)
1	no	no	no	no	no	no	a	a	b	b	b	a
2	no	no	no	no	no	no	a	b	c	b	b	a
3	no	no	no	no	no	no	b	a	c	b	a	c
4	no	no	no	no	no	no	b	a	b	b	a	c
5	no	no	no	no	no	no	a	a	b	b	a	c
6	no	no	no	no	no	no	b	a	b	b	a	b
7	no	no	no	no	no	no	b	a	b	b	a	a
8	no	no	no	no	no	no	a	a	b	b	a	a
9	no	no	no	no	no	no	b	a	b	b	a	a
10	no	no	no	no	no	no	b	a	b	b	b	a
11	no	no	no	no	no	no	a	a	b	b	b	a
12	no	no	no	no	no	no	b	a	a	b	a	a
13	no	no	no	no	no	no	a	a	c	b	a	a
14	no	no	no	no	no	no	b	a	b	b	a	a
15	no	no										a
16	no	no	no	no	no	no	a	a	b	b	b	b
17	no	no	no	no	no	no	a	a	b	b	a	b
18	no	no	no	no	no	no	b	a	b	b	a	a
19	no	no	no	no	no	no	b	a	b	b	a	a
20	no	no	no	no	no	no	b	a	b	b	a	b
21	no	no										a
22	no	no	no	no	no	no	b	b	b	b	a	a

No	Answer 12a (yes/no)	Answer 12b (yes/no)	Answer 12c (yes/no)	Answer 12d (yes/no)	Answer 12e (yes/no)	Answer 12f (yes/no)	Answer 13 (a-d)	Answer 14 (a-e)	Answer 15 (a-c)	Answer 16 (a-b)	Answer 17 (a-b)	Answer 18 (a-d)
23	no	no	no	no	no	no	b	a	b	b	a	a
24	no	no	no	no	no	no	a	a	b	b	a	a
25	no	no	no	no	no	no	a	a	b	b	a	a
26	no	no										a
27	no	no	no	no	no	no	a	a	b	b	b	a
28												a
29	no	no	yes	no	no	no	b	a	b	b	a	a
30	no	no	no	no	no	no	a	a	c	b	a	b
31	no	no										a
32	no	no	no	no	no	no	a	a	b	b	a	a
33	no	no	no	no	no	no	b	a	b	b	a	a
34	no	no	no	no	no	no						a
35	no	no										a
36	no	no										a
37	no	no	no	no	no	no	a	a	a	b	a	a
38	no	no	no	no	no	no	a	a	b	b	a	a
39	no	no	no	no	no	no	a	a	c	b	a	c
40	no	no	no	no	no	no	a	a	b	b	b	a
41	no	no	no	no	no	no	b	a	b	b	a	b
42	no	no	no	no	no	no	a	a	b	b	a	b
43	no	no	no	no	no	no	a	a	c	b	a	a
44	no	no	no	no	no	no	b	a	b	b	a	a
45	no	no	no	no	no	no	b	a	b	a	b	a
46	no	no	no	no	no	no	b	a	a	b	a	c
47	yes	no	no	no	no	no	a	a	b	b	b	a

No	Answer 12a (yes/no)	Answer 12b (yes/no)	Answer 12c (yes/no)	Answer 12d (yes/no)	Answer 12e (yes/no)	Answer 12f (yes/no)	Answer 13 (a-d)	Answer 14 (a-e)	Answer 15 (a-c)	Answer 16 (a-b)	Answer 17 (a-b)	Answer 18 (a-d)
48	no	no	no	no	no	no	b	a	b	b	a	a
49	no	no	no	no	no	no	b	a	c	b	b	c
50	yes	no	no	no	no	no	b	a	b	b	a	a
51	no	no	no	no	no	no	b	a	b	b	a	a
52	no	no	no	no	no	no	c	a	b	b	a	a
53	no	no	no	no	no	no	b	a	b	b	a	a
54	no	no	no	no	no	no	a	a	c	a	a	a
55	no	no	no	no	no	no	a	b	a	b	a	a
56	no	no	no	no	no	no	a	a	c	b	a	a
57	no	no	no	no	no	no	a	a	b	b	a	a
58	no	no	no	no	no	no	b	a	b	b	a	a
59	no	no	no	no	no	no	a	a	a	a	a	a
60	no	no	no	no	no	no	b	a	b	b	a	a
61	yes	no	no	no	no	no	d	a	b	b	a	b
62	no	no	no	no	no	no	b	a	b	b	a	b
63	no	no	no	no	no	no	b	a	b	b	a	a
64	no	no	no	no	no	no	b	a	b	b	a	b
65	no	no	no	no	no	no	a	c	c	b	a	a
66	no	no	no	no	no	no	b	c	c	b	a	a
67	no	no	no	no	no	no	b	a	c	b	a	a
68	no	no	no	no	no	no	a	a	b	b	a	a
69	no	no	no	no	no	no	b	a	b	b	a	a
70	no	no	no	no	no	no	a	a	b	b	a	a
71	no	no	no	no	no	no	b	a	a	b	a	a
72	no	no	no	no	no	no	a	a	c	b	a	a

No	Answer 12a (yes/no)	Answer 12b (yes/no)	Answer 12c (yes/no)	Answer 12d (yes/no)	Answer 12e (yes/no)	Answer 12f (yes/no)	Answer 13 (a-d)	Answer 14 (a-e)	Answer 15 (a-c)	Answer 16 (a-b)	Answer 17 (a-b)	Answer 18 (a-d)
73	yes	no	yes	no	no	no	b	a	b	b	a	b
74	no	no										a
75	no	no										a
76	no	no	no	no	no	no	b	a	b	b	b	c
77	no	no	no	no	no	no	a	a	b	b	a	a
78	no	no	no	no	no	no	b	a	c	b	a	a
79	no	no	no	no	no	no	b	a	b	b	a	a
80	no	no	no	no	no	no	a	a	a	b	a	a
81	no	no	no	no	no	no	a	a	b	b	a	a
82	no	no	no	no	no	no	b	a	b		b	a
83	no	no	no	no	no	no	b	a	b	b	a	a
84	no	no	no	no	no	no	b	a	b	b	b	a
85	no	no	no	no	no	no	b	a	b	b	a	a
86	no	no	no	no	no	no	a	a	b	b	a	b
87	no	no	no	no	no	no	a	a	b	b	a	a
88	no	no	no	no	no	no	b	a	b	b	a	b
89	no	no	no	no	no	no	b	b	b	b	b	a
90	no	no	no	no	no	no	b	a	b	b	b	a
91	no	no	no	no	no	no	b	a	c	b	a	c
92	no	no										a
93	no	no	no	no	no	no	b	a	c	b	a	b
94	no	no	no	no	no	no		c		b	a	d
95	no	no	no	no	no	no	a	a	b	b	a	b
96	no	no	no	no	no	no	b	a	b	b	b	a
97	no	no	no	no	no	no	a	a	b	b	a	a

No	Answer 12a (yes/no)	Answer 12b (yes/no)	Answer 12c (yes/no)	Answer 12d (yes/no)	Answer 12e (yes/no)	Answer 12f (yes/no)	Answer 13 (a-d)	Answer 14 (a-e)	Answer 15 (a-c)	Answer 16 (a-b)	Answer 17 (a-b)	Answer 18 (a-d)
98	no	no	no	no	no	no	a	a	b	b	a	a
99	no	no	no	no	no	no	b	a	b	b	a	a
100	no	no	no	no	no	no	a	a	b	b	a	a
101	no	no	no	no	no	no	b	a	a	b	a	b
102	no	no	no	no	no	no	a	a	b	b	a	b
103	no	no	no	no	no	no	b	a	c	b	a	a
104	no	no	no	no	no	no	b	a	b	a	a	b
105	no	no	no	no	no	no	a	a	b	b	a	a
106	no	no	no	no	no	no	a	a	b	b	a	a
107	no	no	no	no	no	no	b	a	b	b	a	b
108	no	no	no	no	no	no	b	a	b	b	a	c
109	no	no	no	no	no	no	b	a	b	b	a	a
110	no	no	no	no	no	no	b	a	c	b	a	a
111	no	no	no	no	no	no		a	b	b	a	a
112	no	no	no	no	no	no		a	b	b	a	a
113	no	no	no	no	no	no	a	a	c	b	a	a
114	no	no	no	no	no	no	b	a	c	b	a	a
115	no	no	no	no	no	no	b	a	b	b	a	a
116	no	no	no	no	no	no	a	a	c	b	a	a
117	no	no	no	no	no	no	b	a	b	b	a	a
118	no	no	no	no	no	no	b	a	b	b	a	c
119	no	no	no	no	no	no	a	a	b	b	a	a
120	no	no	no	no	no	no	a	a	b	b	a	a
121	no	no	no	no	no	no	a	a	b	b	b	a
122	no	no	no	no	no	no	a	a	b	b	a	c

No	Answer 12a (yes/no)	Answer 12b (yes/no)	Answer 12c (yes/no)	Answer 12d (yes/no)	Answer 12e (yes/no)	Answer 12f (yes/no)	Answer 13 (a-d)	Answer 14 (a-e)	Answer 15 (a-c)	Answer 16 (a-b)	Answer 17 (a-b)	Answer 18 (a-d)
123	no	no	no	no	no	no	b	a	b	b	b	a
124	no	no	no	no	no	no	b	a	c	b	a	a
125	no	no	no	no	no	no	a	a	b	b	a	c
126	no	no										a
127	no	no	no	no	no	no	b	a	c	b	b	a
128	no	no	no	no	no	no	b	a	b	b	a	a
129	no	no	no	no	no	no	b	a	c	b	a	a
130	no	no	no	no	no	no	a	a	c	b	a	c
131	no	no	no	no	no	no	b	a	b	b	b	a
132	no	no	no	no	no	no	a	a	b	b	a	a
133	no	no										a
134	no	no	no	no	no	no	b	a	b	b	a	a
135	no	no	no	no	no	no	b	a	c	b	a	a
136	yes	no	no	no	no	no	b	a	c	b	a	b
137	no	no	no	no	no	no	a	a	b	b	a	b
138	no	no	no	no	no	no	b	a	b	b	a	a
139	no	no	no	no	no	no	b	a	c	b	a	a
140	no	no	no	no	no	no	a	a	a	b	a	a
141	no	no	no	no	no	no	b	a	b	b	b	c
142	no	no	no	no	no	no	b	a	b	a	a	a
143	no	no	no	no	no	no	b	a	a	b	b	a
144	no	no	no	no	no	no	b	a	b	b	a	a
145	no	no	no	no	no	no	a	a	c	b	a	a
146	no	no	no	no	no	no	a	a	b	b	a	a
147	no	no	no	no	no	no	b	a	b	b	a	b

No	Answer 12a (yes/no)	Answer 12b (yes/no)	Answer 12c (yes/no)	Answer 12d (yes/no)	Answer 12e (yes/no)	Answer 12f (yes/no)	Answer 13 (a-d)	Answer 14 (a-e)	Answer 15 (a-c)	Answer 16 (a-b)	Answer 17 (a-b)	Answer 18 (a-d)
148	no	no	no	no	no	no	a	a	b	b	a	b
149	no	no	no	no	no	no	a	b	a	b	a	c
150	no	no	no	no	no	no	b	a	c	b	a	a
151	no	no	no	no	no	no	b	a	b	b	a	a
152	no	no	no	no	no	no	a	a	c	b	a	a
153	no	no	no	no	no	no	a	a	b	b	a	a
154	no	no	no	no	no	no	a	a	c	b	a	n/d
155	no	no	no	no	no	no	a	a	b	b	a	a
156	no	no	no	no	no	no	a	a	c	b	b	a
157	no	no	no	no	no	no	b	a	a	b	b	a
158	no	no	no	no	no	no	a	a	b	b	a	a
159	no	no	no	no	no	no	b	a	c	b	a	b
160	no	no	no	no	no	no	b	a	b	b	a	a
161	no	no	no	no	no	no	b	a	a	b	a	a
162	no	no	no	no	no	no	b	a	b	b	b	a
163	no	no	no	no	no	no	b	a	b	b	a	a
164	no	no	no	no	no	no	a	a	b	b	b	c
165	no	no	no	no	no	no	b	a	b	b	a	a
166	no	no	no	no	no	no	b	c	b	b	a	c
167	no	no	no	no	no	no	a	a	a	b	a	a
168	no	no	no	no	no	no	b	a	b	b	a	a
169	no	no	no	no	no	no	b	a	a	b	a	c
170	no	no										a
171	no	no										a
172	no	no	no	no	no	no	b	a	b	b	a	c

No	Answer 12a (yes/no)	Answer 12b (yes/no)	Answer 12c (yes/no)	Answer 12d (yes/no)	Answer 12e (yes/no)	Answer 12f (yes/no)	Answer 13 (a-d)	Answer 14 (a-e)	Answer 15 (a-c)	Answer 16 (a-b)	Answer 17 (a-b)	Answer 18 (a-d)
173	no	no	no	no	no	no	b	a	b	b	a	a
174	no	no	no	no	no	no	b	a	b	b	a	b
175	no	no	no	no	no	no	a	a	b	b	a	c
176	no	no	no	no	no	no	b	a	b	b	a	a
177	no	no	no	no	no	no	a	a	c	b	a	b
178	no	no										a
179	no	no	no	no	no	no	a	a	b	b	b	b
180	no	no										d
181	no	no	no	no	no	no	a	a	b	b	a	b
182	no	no	no	no	no	no	b	a	c	b	a	a
183	no	no	no	no	no	no	b	a	b	b	a	c
184	no	no	no	no	no	no	b	a	b	b	a	a
185	no	no	no	no	no	no	b	a	b	b	a	a
186	no	no	no	no	no	no	b	b	b	b	a	b
187	no	no	no	no	no	no	b	a	b	b	a	c
188	no	no										b
189	no	no	no	no	no	no	b	a	c	b	a	a
190	no	no	no	no	no	no	a	a	b	b	a	a
191	no	no	no	no	no	no	a	a	c	b	a	b
192	no	no	no	no	no	no	a	a	c	b	a	a
193	no	no	no	no	no	no	a	a	b	b	a	b
194	no											a
195	no	no	no	no	no	no	a	a	b	b	a	a
196	no	no	no	no	no	no	b	a	b	b	a	c
197	no	no	no	no	no	no	a	a	a	b	a	a

No	Answer 12a (yes/no)	Answer 12b (yes/no)	Answer 12c (yes/no)	Answer 12d (yes/no)	Answer 12e (yes/no)	Answer 12f (yes/no)	Answer 13 (a-d)	Answer 14 (a-e)	Answer 15 (a-c)	Answer 16 (a-b)	Answer 17 (a-b)	Answer 18 (a-d)
198	no	no	no	no	no	no	a	a	b	b	a	c
199	no	no	no	no	no	no	a	a	b	b	a	b
200	no	no	no	no	no	no	a	a	b	b	a	a
201	no	no	no	no	no	no	b	a	b	b	a	c
202	no	no	no	no	no	no	b	a	b	b	a	c
203	no	no	no	no	no	no	b	a	c	b	a	a
204	no	no	no	no	no	no	a	a	b		a	a
205	no	no	no	no	no	no	a	a	b		a	a
206	no	no	no	no	no	no	a	a	a	b	a	c
207	no	no	no	no	no	no	a	a	b	b	a	a
208	no	no	no	no	no	no	b	a	b	b	b	a
209	no	no	no	no	no	no	a	a	b	b	a	a
210	no	no	no	no	no	no	a	a	c	b	a	b
211	0	0										a
212	no	no	no	no	no	no	b	a	a	b	a	a
213	no	no	no	no	no	no	a	a	b	b	b	a
214	no	no										a
215	no	no	no	no	no	no	b	a	b	b	a	a
216	no	no	no	no	no	no	b	a	b	b	a	a
217	no	no	no	no	no	no	b	a	b	b	a	a
218	no	no	no	no	no	no	a	a	b	b	a	a
219	yes	no	no	no	no	no	b	a	b	b	a	b
220	no	no										a
221	no	no	no	no	no	no	a	a	c	b	a	a
222	no	no										a

No	Answer 12a (yes/no)	Answer 12b (yes/no)	Answer 12c (yes/no)	Answer 12d (yes/no)	Answer 12e (yes/no)	Answer 12f (yes/no)	Answer 13 (a-d)	Answer 14 (a-e)	Answer 15 (a-c)	Answer 16 (a-b)	Answer 17 (a-b)	Answer 18 (a-d)
223	no	no	no	no	no	no	b	a	b	b	a	a
224	no	no	no	no	no	no	a	a	b	b	b	a
225	no	no	no	no	no	no	b	a	b	b	a	a
226	no	no	no	no	no	no	a	a	b	b	a	a
227	no	no	no	no	no	no	b	a	b	b	b	a
228	no	no	no	no	no	no	a	a	c	b	a	b
229	no	no	no	no	no	no	b	a	b	b	a	c
230	no	no	no	no	no	no	a	a	a	b	a	a
231	no	no	no	no	no	no	b	a	a	b	a	a
232	no	no	no	no	no	no	b	a	b	b	a	c
233	no	no	n	n	n	n	a	a	b	b	a	a
234	no	no	no	no	no	no	b	a	c	b	b	a
235	no	no	no	no	no	no	b	a	b	b	a	a
236	no	no										a
237	no	no	no	no	no	no	a	a	b	b	a	a
238	no	no	no	no	no	no	b	a	b	b	b	a
239	no	no	no	no	no	no	b	a	b	b	a	a
240	no	no	no	no	no	no	a	a	b	b	a	b
241	no	no	no	no	no	no	a	a	b	b	a	a
242	no	no	no	no	no	no	b	a	a	b	a	a
243	no	no	no	no	no	no	b	a	a	b	a	a
244	no	no	no	no	no	no	a	a	b	b	a	c
245	no	no	no	no	no	no	b	a	c	b	b	a
246	no	no	no	no	no	no	b	a	b	b	a	b
247	no	no	no	no	no	no	a	a	a	b	a	a

No	Answer 12a (yes/no)	Answer 12b (yes/no)	Answer 12c (yes/no)	Answer 12d (yes/no)	Answer 12e (yes/no)	Answer 12f (yes/no)	Answer 13 (a-d)	Answer 14 (a-e)	Answer 15 (a-c)	Answer 16 (a-b)	Answer 17 (a-b)	Answer 18 (a-d)
248	no	no	no	no	no	no	b	a	b	a	a	a
249	no	no	no	no	no	no	a	a	a	b	a	a
250	no	no	no	no	no	no	b	a	b	b	b	a
251	no	no	no	no	no	no	a	a	b	b	a	a
252	no	no	no	no	no	no	a	a	c	b	a	b
253	no	no	no	no	no	no	a	a	b	b	a	a
254	no	no	no	no	no	no	a	a	c	b	a	a
255	no	no	no	no	no	no	b	c	b	b	a	a
256	no	no	no	no	no	no	b	a	b	b	a	c
257	no	no	no	no	no	no	a	a	b	b	a	b
258	no	no	no	no	no	no						d
259	no	no	no	no	no	no	a	a	c	b	a	b
260	no	no	yes	no	no	no	a	a	a	b	a	a
261	no	no	no	no	no	no	a	a	a	b	a	a
262	no	no										a
263	yes	no	no	no	no	no	a	a	b	b	b	a
264	yes	no	no	no	no	no	a	a	b	b	b	a
265	no	no	no	no	no	no	b	a	c	b	a	a
266	no	no	no	no	no	no	a	a	a	b	a	b
267	no	no	no	no	no	no	a	a	c	b	a	a
268	no	no	no	no	no	no	a	b	c	b	a	a
269	no	no	no	no	no	no	a	b	c	b	a	a
270	no	no	no	no	no	no	a	a	b	b	a	b
271	no	no	no	no	no	no	a	a	b	b	a	b
272	no	no	no	no	no	no	b	a	b	b	a	a

No	Answer 12a (yes/no)	Answer 12b (yes/no)	Answer 12c (yes/no)	Answer 12d (yes/no)	Answer 12e (yes/no)	Answer 12f (yes/no)	Answer 13 (a-d)	Answer 14 (a-e)	Answer 15 (a-c)	Answer 16 (a-b)	Answer 17 (a-b)	Answer 18 (a-d)
273	no	no	no	no	no	no	b	a	b	b	a	a
274	no	no										a
275	no											a
276	no	no	no	no	no	no	a	a	a	b	a	a
277	no	no	no	no	no	no	a	a	b	b	a	a
278	no	no	no	no	no	no	a	a	b	a	a	b
279	no	no	no	no	no	no	a	a	a	b	a	a
280	no	no	no	no	no	no	b	a	b	b	a	a
281	no	no	no	no	no	no	b	a	b	b	a	b
282	no	no	no	no	no	no	b	a	b	b	b	c
283	no	no	no	no	no	no	b	a	b	b	a	a
284	no	no	no	no	no	no	a	a	b	b	a	a
285	no	no	no	no	no	no	a	a	b	b	b	a
286	no	no	no	no	no	no	a	a	a	b	a	b
287	no	no										a
288	no	no										a
289	no	no	no	no	no	no	b	a	b	b	a	c
290	no	no	no	no	no	no	b	a	b	b	a	a
291	no	no	no	no	no	no	b	a	b	b	a	a
292	no	no	no	no	no	no	a	a	b	b	a	b
293	no	no	no	no	no	no	a	a	b	b	a	b
294	no	no	no	no	no	no	b	a	b	b	a	a
295	no	no	no	no	no	no	a	a	b	b	a	b
296	no	no	no	no	no	no	a	a	a	b	a	a
297	no	no	no	no	no	no	a	a	b	b	b	a

No	Answer 12a (yes/no)	Answer 12b (yes/no)	Answer 12c (yes/no)	Answer 12d (yes/no)	Answer 12e (yes/no)	Answer 12f (yes/no)	Answer 13 (a-d)	Answer 14 (a-e)	Answer 15 (a-c)	Answer 16 (a-b)	Answer 17 (a-b)	Answer 18 (a-d)
298	yes	no	no	no	no	no	a	a	c	b	a	c
299	no	no	no	no	no	no	a	a	a	b	a	b
300	no	no	no	yes	no	no	b	a	c	b	a	c
301	no	no	no	no	no	no	b	a	c	b	b	a
302	no	no										a
303	no	no	no	no	no	no	b	a	b	b	a	a
304	no	no	no	no	no	no	b	a	b	b	a	a
305	no	no	no	no	no	no	a	b	a	b	a	a
306	no	no	no	no	no	no	b	a	a	b	a	a
307	no	no	no	no	no	no	a	a	b	b	b	a
308	no	no	no	no	no	no	b	a	b	b	b	b
309	no	no	no	yes	no	no	a	b	b	b	a	c
310	no	no	no	no	no	no	b	a	b	b	a	c
311	no	no	no	no	no	no	a	a	b	b	a	a
312	no	no										a
313	no	no	no	no	no	no	b	a	b	b	a	c
314	no	no	no	no	no	no	a	a	b	b	b	a
315	no	no	no	no	no	no	b	a	b	b	b	a
316	no	no	no	no	no	no	a	a	b	b	b	a
317	no	no	no	no	no	no	b	a	b	b	b	a
318	no	no										a
319	0	n/d	n/d	n/d	n/d	n/d	n/d	n/d	n/d	n/d	n/d	n/d
320	0	n/d	n/d	n/d	n/d	n/d	n/d	n/d	n/d	n/d	n/d	n/d
321	no	no	no	no	no	no	a	a	c	b	a	c
322	no	no	no	no	no	no	a	a	b	a	b	b

No	Answer 12a (yes/no)	Answer 12b (yes/no)	Answer 12c (yes/no)	Answer 12d (yes/no)	Answer 12e (yes/no)	Answer 12f (yes/no)	Answer 13 (a-d)	Answer 14 (a-e)	Answer 15 (a-c)	Answer 16 (a-b)	Answer 17 (a-b)	Answer 18 (a-d)
323	no	no	no	no	no	no	b	a	b	b	a	a
324	no	no	no	no	no	no	b	a	a	b	a	a
325	yes	no	no	no	no	no	a	a	b	b	a	c
326	no	no	no	no	no	no	b	a	b	b	b	a
327	no	no	no	no	no	no	b	a	b	b	a	a
328	no	no	no	no	no	no	b	a	b	b	a	a
329	no	no	no	no	no	no	a	a	c	b	a	a
330	no	no	no	no	no	no	b	a	c	b	b	a
331	no	no	no	no	no	no	a	a	b	b	a	a
332	no	no										a
333	no	no	no	no	no	no	b	a	b	b	a	a
334	no	no										a
335	no	no	no	no	no	no	a	a	a	b	b	a
336	no	no	no	no	no	no	b	a	b	b	a	a
337												a
338	no	no	no	no	no	no	b	a	b	b	a	c
339	no	no	no	no	no	no	a	a	b	b	a	a
340	no	no	no	no	no	no	b	a	c	b	a	a
341	no	no	no	no	no	no	b	a	b	b	a	a
342	no	no	no	no	no	no	b	a	c	b	a	c
343	no	no										a
344	no	no	no	no	no	no	b	a	a	b	a	c
345	no	no	no	no	no	no	a	a	b	b	a	a
346	no	no	no	no	no	no	a	a	b	b	a	b
347	no	no										a

No	Answer 12a (yes/no)	Answer 12b (yes/no)	Answer 12c (yes/no)	Answer 12d (yes/no)	Answer 12e (yes/no)	Answer 12f (yes/no)	Answer 13 (a-d)	Answer 14 (a-e)	Answer 15 (a-c)	Answer 16 (a-b)	Answer 17 (a-b)	Answer 18 (a-d)
348	no	no	no	no	no	no	b	a	b	b	a	d
349	no	no	no	no	no	no	a	a	c	b	b	a
350	no	no	no	no	no	no	b	a	c	b	a	b
351	no	no										a
352	no	no	no	no	no	no	a	a	b	b	a	a
353	no	no	no	no	no	no	a	a	b	b	a	a
354	no	no	no	no	no	no	b	a	c	b	a	a
355	no	no	no	no	no	no	a	a	b	b	a	a
356	no	no	no	no	no	no	b	a	c	b	a	b
357	no	no	no	no	no	no	b	a	b	b	a	a
358	no	no	no	no	no	no	b	a	b	b	a	a
359	no	no	no	no	no	no	b	a	b	b	a	a
360	no	no										a
361	no	no	no	no	no	no	b	a	b	b	a	a
362	no	no	no	no	no	no	b	a	b	b	a	a
363	no	no	no	no	no	no	a	a	b	b	a	a
364	no	no	no	no	no	no	a	a	b	b	a	b
365	no	no	no	no	no	no	a	a	c	b	a	b
366	no	no	no	no	no	no	a	a	b	b	a	a
367	no	no	no	no	no	no	a	a	b	b	a	a
368	no	no	no	no	no	no	a	a	c	b	a	a
369	no	no	no	no	no	no	b	a	b	b	a	a
370	no	no	no	no	no	no	b	a	c	b	a	a
371	no	no	no	no	no	no	a	a	a	b	a	b
372	no	no	no	no	no	no	b	a	a	b	a	a

No	Answer 12a (yes/no)	Answer 12b (yes/no)	Answer 12c (yes/no)	Answer 12d (yes/no)	Answer 12e (yes/no)	Answer 12f (yes/no)	Answer 13 (a-d)	Answer 14 (a-e)	Answer 15 (a-c)	Answer 16 (a-b)	Answer 17 (a-b)	Answer 18 (a-d)
373	no	no	no	no	no	no	a	a	b	b	a	a
374	no	no	no	no	no	no	b	a	c	b	a	a
375	no	no	no	no	no	no	a	a	b	b	a	a
376	no	no	no	no	no	no	b	a	a	a	a	a
377	no	no	no	no	no	no	b	a	b	b	b	b
378	no	no	no	no	no	no	b	a	b	b	a	a
379	no	no	no	no	no	no	b	a	c	b	a	a
380	no	no	no	no	no	no	b	b	b	b	b	a
381	no	no	no	no	no	no		a	c	b	a	b
382	no	no	no	no	no	no	b	a	b	b	a	a
383	no	no	no	no	no	no	a	a	c	b	b	a
384	no	no	no	no	no	no	a	a	c	b	a	a
385	no	no	no	no	no	no	a	b	c	b	a	d
386	no	no	no	no	no	no	b	a	a	b	a	a
387	no	no	no	no	no	no	a	a	b	b	a	a
388	no	no	no	no	no	no	b	a	c	b	a	a
389	no	no	no	no	no	no	b	a	b	b	b	a
390	no	no	no	no	no	no	a	a	b	b	b	a
391	no	no	no	no	no	no	b	a	a	b	a	a
392	no	no	no	no	no	no	b	a	c	b	a	a
393	no	no	no	no	no	no	b	a	b	b	a	a
394	no	no	no	n	no	no	a	a	a	b	b	a
395	no	no	no	no	no	no	b	a	a	b	a	a
396	no	no	no	no	no	no	a	a	c	b	a	a
397	no	no	no	no	no	no	b	a	b	b	a	a

No	Answer 12a (yes/no)	Answer 12b (yes/no)	Answer 12c (yes/no)	Answer 12d (yes/no)	Answer 12e (yes/no)	Answer 12f (yes/no)	Answer 13 (a-d)	Answer 14 (a-e)	Answer 15 (a-c)	Answer 16 (a-b)	Answer 17 (a-b)	Answer 18 (a-d)
398	no	no	no	no	no	no	b	a	c	b	a	b
399	no	no	no	no	no	no	a	a	b	b	a	a
400	no	no	no	no	no	no	b	a	a	b	a	b
401	no	no	no	no	no	no	b	a	c	b	b	a
402	no	no	no	no	no	no	b	a	b	b	b	c
403	no	no	no	no	no	no	b	a	a	b	a	a
404	no	no	no	no	no	no	a	a	a	b	a	a
405	no	no	no	no	no	no	b	a	b	b	a	b
406	no	no	no	no	no	no	b	b	b	b	a	c
407	no	no	no	no	no	no	a	a	a	a	a	a
408	no	no	no	no	no	no	b	a	b	b	a	b
409	no	no	no	no	no	no	a	a	b	b	a	a
410	no	no	no	no	no	no	b	a	b	b	a	b
411	no	no	no	no	no	no	b	a	b	b	a	a
412	no	no	no	no	no	no	b	b	b	b	a	d
413	no	no	no	no	no	no	a	a	c	b	a	b
414	no	no	no	no	no	no	b	a	b	b	b	a
415	no	no										a
416	no	no	no	no	no	no	a	a	c	b	a	a
417	no	no	no	no	no	no	b	a	c	b	a	a
418	no	no	no	no	no	no	a	a	b	b	a	a
419	no	no	no	no	no	no	b	a	a	b	a	a
420	no	no	no	no	no	no	b	a	b	b	a	a
421	no	no	no	no	no	no	b	a	b	b	a	a
422	no	no	no	no	no	no	b	a	b	b	a	a

No	Answer 12a (yes/no)	Answer 12b (yes/no)	Answer 12c (yes/no)	Answer 12d (yes/no)	Answer 12e (yes/no)	Answer 12f (yes/no)	Answer 13 (a-d)	Answer 14 (a-e)	Answer 15 (a-c)	Answer 16 (a-b)	Answer 17 (a-b)	Answer 18 (a-d)
423	no	no	no	no	no	no	b	a	b	b	a	a
424	no	no	no	no	no	no	b	a	b	b	a	a
425	no	no	no	no	no	no	a	a	b	a	a	b
426	no	no	no	no	no	no	a	a	b	b	a	a
427	no	no	no	no	no	no	b	a	b	b	a	a
428	no	no	no	no	no	no	a	a	c	b	a	a
429	no	no	no	no	no	no	a	a	c	b	a	a
430	no	no	no	no	no	no	a	a	b	b	b	a
431	no	no	no	no	no	no	a	a	b	b	a	b
432	no	no	no	no	no	no	a	a	c	b	a	a
433	no	no	no	no	no	no	a	a	c	b	a	a
434	no	no	no	no	no	no	b	a	b	b	a	a
435	no	no	no	no	no	no	b	a	b	b	a	a
436	no	no	no	no	no	no	b			b	a	d
437	no	no	no	no	no	no	a	a	c	b	b	a
438	no	no	no	no	no	no	a	c	b	b	a	c
439	no	no	no	no	no	no	b	a	c	b	b	a
440	no	no	no	no	no	no	b	a	b	b	a	a
441	no	no	no	no	no	no	a	a	b	b	a	a
442	no	no	no	no	no	no	a	a	c	b	a	a
443	no	no	no	no	no	no	a	a	b	b	a	a
444	no	no	no	no	no	no	a	a	b	b	a	a
445	no	no	no	no	no	no	a	a	b	b	a	a
446	no	no	no	no	no	no	b	a	b	b	a	a
447	no	no	no	no	no	no	b	a	c	a	a	a

No	Answer 12a (yes/no)	Answer 12b (yes/no)	Answer 12c (yes/no)	Answer 12d (yes/no)	Answer 12e (yes/no)	Answer 12f (yes/no)	Answer 13 (a-d)	Answer 14 (a-e)	Answer 15 (a-c)	Answer 16 (a-b)	Answer 17 (a-b)	Answer 18 (a-d)
448	no	no	no	no	no	no	a	a	b	b	b	b
449	no	no	no	no	no	no	b	a	b	b	b	a
450	no	no	yes	no	no	no	b	a	b	b	a	b
451	no	no	yes	no	no	no	b	a	b	b	a	b
452	no	no	no	no	no	no	a	a	c	b	a	a
453	no	no	no	no	no	no	b	a	b	b	a	a
454	no	no	no	no	no	no	a	b	b	b	a	a
455	no	no	no	no	no	no	a	a	b	b	b	a
456	no	no	no	no	no	no	a	a	b	b	a	a
457	no	no	no	no	no	no	b	a	c	b	a	a
458	no	no	no	no	no	no	b	a	b	b	a	a
459	no	no	no	no	no	no	b	a	c	b	a	a
460	no	no	no	no	no	no	a	a	b	b	a	a
461	no	no	no	no	no	no	a	a	b	b	a	a
462	no	no	no	no	no	no	b	b	b	b	b	a
463	no	no	no	no	no	no	b	a	a	b	a	a
464	no	no	no	no	no	no	b	a	a	b	a	a
465	no	no	no	no	no	no	b	a	b	b	a	a
466	no	no	no	no	no	no	a	a	c	b	a	d
467	no	no	no	no	no	no	b	a	b	b	a	a
468	no	no	no	no	no	no	a	a	c	b	a	a
469	no	no	no	no	no	no	a	a	b	b	a	b
470	no	no	no	no	no	no	b	a	c	b	a	b
471	no	no	no	no	no	no	b	a	a	b	a	a
472	no	no	no	no	no	no	b	a	c	b	a	b

No	Answer 12a (yes/no)	Answer 12b (yes/no)	Answer 12c (yes/no)	Answer 12d (yes/no)	Answer 12e (yes/no)	Answer 12f (yes/no)	Answer 13 (a-d)	Answer 14 (a-e)	Answer 15 (a-c)	Answer 16 (a-b)	Answer 17 (a-b)	Answer 18 (a-d)
473	no	no	no	no	no	no	b	a	c	b	a	b
474	no	no	no	no	no	no	b	a	c	b	a	a
475	no	no	no	no	no	no	a	a	b	b	a	a
476	no	no	no	no	no	no	b	a	c	b	b	a
477	no	no	no									a
478	no	no	no	no	no	no	b	a	b	b	a	a
