# Supplementary material for: Estimation of Prenatal Alcohol Exposure: Comparison of Retrospective Survey and Measurement of Fatty Acid Ethyl Esters, Ethyl Sulfate, and Ethyl Glucuronide Concentrations in Neonatal Meconium
Source: Toxics. 2026 Feb 4;14(2):155. doi: 10.3390/toxics14020155 (PMC12944540; doi:10.3390/toxics14020155)
Supplement: Supplementary file 1 [file toxics-14-00155-s001.zip › Table S06 answers 19a-23c.pdf]

Table S6. Results of survey questions 19a to 23c (n=478) in pregnant women conducted at the Neonatology Clinic of the Medical University of Gdańsk in the Pomeranian Province between June 16, 2019, and April 24, 2020.

No	Answer 19a (cigarettes)	Answer 19b (cigarettes)	Answer 19c (cigarettes)	Answer 19d (cigarettes)	Answer 20 (a-e)	Answer 21 (a-e)	Answer 22a (yes/no)	Answer 22b (yes/no)	Answer 22c (yes/no)	Answer 23a (yes/no)	Answer 23b (yes/no)	Answer 23c (yes/no)
1					b	a						
2				0	b	a						
3	10	0	0	0	c	a						
4	1	0	0	0	d	b	yes	no	no	no	yes	yes
5	0	2	0	0	a	a						
6	0	0	0	0	b	a						
7	0	0	0	0	c	a						
8	0	0	0	0	c	a						
9	0	0	0	0	c	a						
10	0	0	0	0	b	a						
11					b	a						
12	0	0	0	0	c	a						
13	0	0	0	0	c	a						
14	0	0	0	0	b	a						
15					b	a						
16	2	0	0	0	c	a	no	yes	no			
17	0	0	0	0	c	a						
18	0	0	0	0	d	a						
19	0	0	0	0	b	a						
20	10	0	0	0	c	a						
21					a	a						

No	Answer 19a (cigarettes)	Answer 19b (cigarettes)	Answer 19c (cigarettes)	Answer 19d (cigarettes)	Answer 20 (a-e)	Answer 21 (a-e)	Answer 22a (yes/no)	Answer 22b (yes/no)	Answer 22c (yes/no)	Answer 23a (yes/no)	Answer 23b (yes/no)	Answer 23c (yes/no)
22	0	0	0	0	b	a						
23	0	0	0	0	c	a						
24	0	0	0	0	c	a						
25	0	0	0	0	b	a						
26					a	a						
27	0	0	0	0	a	a						
28					b	a						
29	0	0	0	0	a	a						
30	0	0	0	0	c	a						
31					b	a						
32	0	0	0	0	c	a						
33	0	0	0	0	b	a						
34						a						
35					b	a						
36					b	a						
37	0	0	0	0	b	a						
38	0	0	0	0	c	a						
39	15	1	0	0	b	a						
40	0	0	0	0	e	a						
41	0	0	0	0	b	a						
42	0	0	0	0	d	b	yes	no	no	yes	yes	yes
43	0	0	0	0	a	a						
44	0	0	0	0	c	a						
45	0	0	0	0	a	a						

No	Answer 19a (cigarettes)	Answer 19b (cigarettes)	Answer 19c (cigarettes)	Answer 19d (cigarettes)	Answer 20 (a-e)	Answer 21 (a-e)	Answer 22a (yes/no)	Answer 22b (yes/no)	Answer 22c (yes/no)	Answer 23a (yes/no)	Answer 23b (yes/no)	Answer 23c (yes/no)
46	15	0	0	0	b	a						
47	0	0	0	0	a	a						
48	0	0	0	0	c	a						
49	9	0	0	0	b	a						
50	0	0	0	0	c	a						
51	0	0	0	0	c	a						
52	0	0	0	0	c	a						
53	0	0	0	0	c	a						
54	0	0	0	0	c	a	yes	yes	yes	yes	no	no
55	0	0	0	0	c	b	no	152	no	no	no	yes
56	0	0	0	0	c	a						
57	0	0	0	0	a	a						
58	0	0	0	0	b	a						
59	0	0	0	0	b	a						
60	0	0	0	0	c	a						
61	5	0	0	0	e	a						
62	2	0	0	0	c	a						
63	0	0	0	0	c	a						
64	0	0	0	0	b	a						
65	0	0	0	0	d	b	no	yes	no	no	no	yes
66	0	0	0	0	c	a						
67	0	0	0	0	c	a						
68	0	0	0	0	c	a						
69	0	0	0	0	b	a						

No	Answer 19a (cigarettes)	Answer 19b (cigarettes)	Answer 19c (cigarettes)	Answer 19d (cigarettes)	Answer 20 (a-e)	Answer 21 (a-e)	Answer 22a (yes/no)	Answer 22b (yes/no)	Answer 22c (yes/no)	Answer 23a (yes/no)	Answer 23b (yes/no)	Answer 23c (yes/no)
70	0	0	0	0	b	a						
71	0	0	0	0	b	a						
72	0	0	0	0	b	a						
73	0	0	0	0	c	a						
74					b	a						
75					b	a						
76	15	0	0	0	b	a						
77	0	0	0	0	b	a						
78	0	0	0	0	c	a						
79	0	0	0	0	c	a						
80	0	0	0	0	b	a						
81	0	0	0	0	c	a						
82	0	0	0	0		a						
83	0	0	0	0	b	a						
84	0	0	0	0	b	a						
85	0	0	0	0	b	a						
86	0	0	0	0	c	a						
87	0	0	0	0	b	a						
88	0	0	0	0	c	a						
89					a	a						
90	0	0	0	0	b	a						
91	5	0	0	0	c	a						
92					a	a						
93	0	0	0	0	b	a						

No	Answer 19a (cigarettes)	Answer 19b (cigarettes)	Answer 19c (cigarettes)	Answer 19d (cigarettes)	Answer 20 (a-e)	Answer 21 (a-e)	Answer 22a (yes/no)	Answer 22b (yes/no)	Answer 22c (yes/no)	Answer 23a (yes/no)	Answer 23b (yes/no)	Answer 23c (yes/no)
94				10	c	c	yes	no	no	yes	no	no
95	0	0	0	0	c	a						
96	0	0	0	0	b	a						
97	0	0	0	0	a	a						
98	0	0	0	0	a	a						
99	0	0	0	0	b	a						
100	0	0	0	0	b	a						
101	1	0	0	0	c	a						
102	0	0	0	0	c	a						
103	0	0	0	0	c	a						
104	0	0	0	0	c	a						
105	0	0	0	0	c	a						
106	0	0	0	0	c	a						
107	0	0	0	0	b	a						
108	5	0	0	0	c	a						
109					b	a						
110	0	0	0	0	b	a						
111	0	0	0	0	b	a						
112	0	0	0	0	a	a						
113					c	a						
114					c	a						
115					b	a						
116					b	a						
117					c	a						

No	Answer 19a (cigarettes)	Answer 19b (cigarettes)	Answer 19c (cigarettes)	Answer 19d (cigarettes)	Answer 20 (a-e)	Answer 21 (a-e)	Answer 22a (yes/no)	Answer 22b (yes/no)	Answer 22c (yes/no)	Answer 23a (yes/no)	Answer 23b (yes/no)	Answer 23c (yes/no)
118	5	2	0	0	c	a						
119					b	a						
120					a	a						
121					b	a						
122	4	0	0	0	b	a						
123	0	0	0	0	b	a						
124	0	0	0	0	c	a						
125	5				a	a						
126					a	a						
127	0	0	0	0	a	a						
128	0	0	0	0	a	a						
129	0	0	0	0	a	a						
130	0	0	0	0	b	a						
131					a	a						
132	0	0	0	0		a						
133					b	a						
134					b	a						
135	0	0	0	0	b	a						
136	0	0		0	c	a						
137	0	0	0	0	b	a						
138	0	0	0	0	c	a						
139	0	0	0	0	b	a						
140					b	a						
141	0	4	0	0	a	a						

No	Answer 19a (cigarettes)	Answer 19b (cigarettes)	Answer 19c (cigarettes)	Answer 19d (cigarettes)	Answer 20 (a-e)	Answer 21 (a-e)	Answer 22a (yes/no)	Answer 22b (yes/no)	Answer 22c (yes/no)	Answer 23a (yes/no)	Answer 23b (yes/no)	Answer 23c (yes/no)
142	0	0	0	0	c	a						
143	0	0	0	0	b	a						
144	0	0	0	0	a	a						
145	0	0	0	0	b	a						
146	0	0	0	0	b	a						
147	0	0	0	0	c	a						
148	20	0	0	0	b	a						
149	20	5	0	0	c	c	yes	no	no			
150	0	0	0	0	c	a						
151	0	0	0	0	b	a						
152					a	a						
153					b	a						
154	n/d	n/d	n/d	n/d	b	a						
155					b	a						
156					c	a						
157					b	a						
158					c	a						
159					b	a						
160					c	a						
161					d	a						
162					b	a						
163					a	a						
164		3			a	a						
165					a	a						

No	Answer 19a (cigarettes)	Answer 19b (cigarettes)	Answer 19c (cigarettes)	Answer 19d (cigarettes)	Answer 20 (a-e)	Answer 21 (a-e)	Answer 22a (yes/no)	Answer 22b (yes/no)	Answer 22c (yes/no)	Answer 23a (yes/no)	Answer 23b (yes/no)	Answer 23c (yes/no)
166	1				c	a		yes				yes
167					c	a						
168	0	0	0	0	c	a						
169	7	3	0	0	c	a						
170						a						
171						a						
172	10	0	0	0	b	a						
173	0	0	0	0	b	a						
174	2	0	0	0	c	a						
175	4	0	0	0	d	a						
176					c	a						
177	0	0	0	0	b	a						
178						a						
179					a	a						
180	20	20	20	20		a						
181					b	a						
182					b	a						
183	10				c	a						
184					c	a						
185					c	a						
186					c	b	yes	yes		yes		
187	5	5	5	0	b	a						
188	0	0	0	0		a						
189					b	a						

No	Answer 19a (cigarettes)	Answer 19b (cigarettes)	Answer 19c (cigarettes)	Answer 19d (cigarettes)	Answer 20 (a-e)	Answer 21 (a-e)	Answer 22a (yes/no)	Answer 22b (yes/no)	Answer 22c (yes/no)	Answer 23a (yes/no)	Answer 23b (yes/no)	Answer 23c (yes/no)
190					a	b						
191					c	a						
192					b	a						
193					c	b		yes		yes		
194						a						
195					c	a						
196	10	2	0	0	b	a						
197					b	a						
198	15	4	0	0	c	a						
199					e	a						
200	0	0	0	0	c	a						
201	5	0	0	0	b	a						
202		4			b	a						
203					b	a						
204					c	a						
205					c	a						
206	5				c	a						
207					c	a						
208	0	0	0	0	c	a						
209	0	0	0	0	c	a						
210	0	0	0	0	b	a						
211						a						
212					b	a						
213	0	0	0	0	a	a						

No	Answer 19a (cigarettes)	Answer 19b (cigarettes)	Answer 19c (cigarettes)	Answer 19d (cigarettes)	Answer 20 (a-e)	Answer 21 (a-e)	Answer 22a (yes/no)	Answer 22b (yes/no)	Answer 22c (yes/no)	Answer 23a (yes/no)	Answer 23b (yes/no)	Answer 23c (yes/no)
214						a						
215	0	0	0	0	b	a						
216					c	a						
217	0	0	0	0	d	a						
218	0	0	0	0	c	a						
219	3	0	0	0	c	a						
220						a						
221	0	0	0	0	a	a						
222						a						
223					b	a						
224	0	0	0	0	b	a						
225	0	0	0	0	b	a						
226	0	0	0	0	b	a						
227	0	0	0	0	a	a						
228	0	0	0	0	c	a						
229	5	2	0	0	c	a						
230	0	0	0	0	b	a						
231	0	0	0	0	c	a						
232	0	0	0	0	c	a						
233					b	b	a					yes
234	0	0	0	0	a	a						
235	0	0	0	0	b	a						
236						a						
237	0	0	0	0	c	c	yes	yes	no	no	yes	no

No	Answer 19a (cigarettes)	Answer 19b (cigarettes)	Answer 19c (cigarettes)	Answer 19d (cigarettes)	Answer 20 (a-e)	Answer 21 (a-e)	Answer 22a (yes/no)	Answer 22b (yes/no)	Answer 22c (yes/no)	Answer 23a (yes/no)	Answer 23b (yes/no)	Answer 23c (yes/no)
238	0	0	0	0	c	a						
239	0	0	0	0	b	a						
240	0	0	0	0	c	a						
241	0	0	0	0	a	a						
242	0	0	0	0	b	a						
243	0	0	0	0	d	a						
244	8	0	0	0	c	b	yes	no	no	no	yes	yes
245	0	0	0	0	b	a						
246	7	0	0	0	d	a						
247	0	0	0	0	d	a						
248	0	0	0	0	b	a						
249	0	0	0	0	a	a						
250	0	0	0	0	b	a						
251	0	0	0	0	b	a						
252	0	0	0	0	b	a						
253	0	0	0	0	c	a						
254	0	0	0	0	b	a						
255	0	0	0	0	b	a						
256	0	0	0	0	b	a						
257	0	0	0	0	b	b						
258					c	b						
259	0	0	0	0	a	a						
260	0	0	0	0	b	a						
261	0	0	0	0	c	a						

No	Answer 19a (cigarettes)	Answer 19b (cigarettes)	Answer 19c (cigarettes)	Answer 19d (cigarettes)	Answer 20 (a-e)	Answer 21 (a-e)	Answer 22a (yes/no)	Answer 22b (yes/no)	Answer 22c (yes/no)	Answer 23a (yes/no)	Answer 23b (yes/no)	Answer 23c (yes/no)
262					b	a						
263	0	0	0	0	c	a						
264	0	0	0	0	c	a						
265	0	0	0	0	c	a						
266	2	0	0	0	b	a						
267	0	0	0	0	c	a						
268	0	0	0	0	c	a						
269		0	0	0	c	a						
270	3	0	0	0	d	a						
271	15	0	0	0	b	a						
272	0	0	0	0	b	a						
273	0	0	0	0	b	a						
274						a						
275												
276	0	0	0	0	b	a						
277	0	0	0	0	b	a						
278	0	0	0	0	d	b	yes	no	no	no	no	yes
279	0	0	0	0	b	a						
280	0	0	0	0	b	a						
281	5	0	0	0	c	a						
282	2	0	0	0	c	a						
283	0	0	0	0	b	a						
284	0	0	0	0	b	a						
285	0	0	0	0	b	a						

No	Answer 19a (cigarettes)	Answer 19b (cigarettes)	Answer 19c (cigarettes)	Answer 19d (cigarettes)	Answer 20 (a-e)	Answer 21 (a-e)	Answer 22a (yes/no)	Answer 22b (yes/no)	Answer 22c (yes/no)	Answer 23a (yes/no)	Answer 23b (yes/no)	Answer 23c (yes/no)
286	10	1	0	0	b	a						
287						a						
288						a						
289	10	0	0	0	b	a						
290	0	0	0	0	c	a						
291	0	0	0	0	a	a						
292	10	0	0	0	c	a						
293	0	0	0	0	c	a						
294	0	0	0	0	b	a						
295	14	0	0	0	d	a						
296	0	0	0	0	b	a						
297	0	0	0	0	b	a						
298	10	0	0	0	c	b	yes	no	no	no	yes	no
299	0	0	0	0	b	a						
300	3	3	0	0	b	a						
301	0	0	0	0	b	a						
302						a						
303	0	0	0	0	b	a						
304	0	0	0	0	b	a						
305	0	0	0	0	a	a						
306	0	0	0	0	a	a						
307	0	0	0	0	b	a						
308	0	0	0	0	b	a						
309	3	1	0	0	b	a						

No	Answer 19a (cigarettes)	Answer 19b (cigarettes)	Answer 19c (cigarettes)	Answer 19d (cigarettes)	Answer 20 (a-e)	Answer 21 (a-e)	Answer 22a (yes/no)	Answer 22b (yes/no)	Answer 22c (yes/no)	Answer 23a (yes/no)	Answer 23b (yes/no)	Answer 23c (yes/no)
310	1	0	0	0	d	a						
311	0	0	0	0	b	a						
312						a						
313	0	0	0	0	b	a						
314	0	0	0	0	b	a						
315	0	0	0	0	d	a						
316	0	0	0	0	b	a						
317	0	0	0	0	b	a						
318						a						
319	n/d	n/d	n/d	n/d	n/d	n/d	n/d	n/d	n/d	n/d	n/d	n/d
320	n/d	n/d	n/d	n/d	n/d	n/d	n/d	n/d	n/d	n/d	n/d	n/d
321	15	0	0	0	b	a						
322	3	0	0	0	c	a						
323	0	0	0	0	a	a						
324	0	0	0	0	b	a						
325	10	2	0	0	c	a						
326	0	0	0	0	a	a						
327	0	0	0	0	d	a						
328	0	0	0	0	b	a						
329	0	0	0	0	c	a						
330	0	0	0	0	b	a						
331	0	0	0	0	c	a						
332						a						
333	0	0	0	0	b	a						

No	Answer 19a (cigarettes)	Answer 19b (cigarettes)	Answer 19c (cigarettes)	Answer 19d (cigarettes)	Answer 20 (a-e)	Answer 21 (a-e)	Answer 22a (yes/no)	Answer 22b (yes/no)	Answer 22c (yes/no)	Answer 23a (yes/no)	Answer 23b (yes/no)	Answer 23c (yes/no)
334						a						
335	0	0	0	0	b	a						
336	0	0	0	0	c	a						
337						a						
338	12	0	0	0	b	a						
339	0	0	0	0	b	a						
340					d	a						
341	0	0	0	0	b	a						
342	0	5	0	0	b	a						
343						a						
344	0	0	0	0	b	a						
345	0	0	0	0	b	a						
346	15	0	0	0	b	a						
347						a						
348	15	0	0	0	a	a						
349	0	0	0	0	b	a						
350					d	a						
351						a						
352	0	0	0	0	d	a						
353	0	0	0	0	b	a						
354	0	0	0	0	c	a						
355	0	0	0	0	b	a						
356	1	0	0	0	c	a						
357	0	0	0	0	c	a						

No	Answer 19a (cigarettes)	Answer 19b (cigarettes)	Answer 19c (cigarettes)	Answer 19d (cigarettes)	Answer 20 (a-e)	Answer 21 (a-e)	Answer 22a (yes/no)	Answer 22b (yes/no)	Answer 22c (yes/no)	Answer 23a (yes/no)	Answer 23b (yes/no)	Answer 23c (yes/no)
358	0	0	0	0	c	a						
359	0	0	0	0	b	a						
360						a						
361	0	0	0	0	b	a						
362	0	0	0	0	b	a						
363	0	0	0	0	c	c	yes	no	no	yes	yes	yes
364	2	0	0	0	b	a						
365	0	0	0	0	b	a						
366	0	0	0	0	c	a						
367	0	0	0	0	c	a						
368	0	0	0	0	a	a						
369	0	0	0	0	c	a						
370	0	0	0	0	c	a						
371	0	0	0	0	c	a						
372	0	0	0	0	d	a						
373	0	0	0	0	b	a						
374	0	0	0	0	c	a						
375	0	0	0	0	c	a						
376	0	0	0	0	d	a						
377	8	0	0	0	b	a						
378	0	0	0	0	b	a						
379	0	0	0	0	c	a						
380	0	0	0	0	b	a						
381	1	0	0	0	c	a						

No	Answer 19a (cigarettes)	Answer 19b (cigarettes)	Answer 19c (cigarettes)	Answer 19d (cigarettes)	Answer 20 (a-e)	Answer 21 (a-e)	Answer 22a (yes/no)	Answer 22b (yes/no)	Answer 22c (yes/no)	Answer 23a (yes/no)	Answer 23b (yes/no)	Answer 23c (yes/no)
382	0	0	0	0	c	a						
383					b	a						
384					b	a						
385				10	b	a						
386	0	0	0	0	a	a						
387	0	0	0	0	c	a						
388					b	a						
389	0	0	0	0	b	a						
390	0	0	0	0	b	b	yes	no	no	yes	no	no
391	0	0	0	0	b	a						
392	0	0	0	0	c	a						
393	0	0	0	0	b	a						
394					c	a						
395	0	0	0	0	c	a						
396	0	0	0	0	b	a						
397	0	0	0	0	b	a						
398	0	0	0	0	c	a						
399	0	0	0	0	b	a						
400	0	0	0	0	c	a						
401	0	0	0	0	a	a						
402	10	0	0	0	b	a						
403	0	0	0	0	a	a						
404	0	0	0	0	c	a						
405	0	0	0	0	a	a	no	yes	no	yes	no	no

No	Answer 19a (cigarettes)	Answer 19b (cigarettes)	Answer 19c (cigarettes)	Answer 19d (cigarettes)	Answer 20 (a-e)	Answer 21 (a-e)	Answer 22a (yes/no)	Answer 22b (yes/no)	Answer 22c (yes/no)	Answer 23a (yes/no)	Answer 23b (yes/no)	Answer 23c (yes/no)
406	1	0	0	0	b	a						
407	0	0	0	0	c	c	yes	no	no	no	no	yes
408	2	0	0	0	c	a						
409	0	0	0	0	b	a						
410	0	0	0	0	b	a						
411	0	0	0	0	c	a						
412	6	4	3	0	a	a						
413	0	0	0	0	a	a						
414	0	0	0	0	c	a						
415						a						
416	0	0	0	0	b	a						
417	0	0	0	0	c	a						
418	0	0	0	0	c	a						
419	0	0	0	0	c	a						
420	0	0	0	0	c	a						
421	0	0	0	0	c	a						
422	0	0	0	0	c	b	yes	no	no	no	yes	no
423	0	0	0	0	c	a						
424	0	0	0	0	c	a						
425	5	0	0	0	c	a						
426	0	0	0	0	b	a						
427	0	0	0	0	b	a						
428	0	0	0	0	b	a						
429	0	0	0	0	d	a						

No	Answer 19a (cigarettes)	Answer 19b (cigarettes)	Answer 19c (cigarettes)	Answer 19d (cigarettes)	Answer 20 (a-e)	Answer 21 (a-e)	Answer 22a (yes/no)	Answer 22b (yes/no)	Answer 22c (yes/no)	Answer 23a (yes/no)	Answer 23b (yes/no)	Answer 23c (yes/no)
430	0	0	0	0	c	a						
431	5	0	0	0	b	a						
432	0	0	0	0	b	a						
433	0	0	0	0	b	a						
434	0	0	0	0	c	a						
435	0	0	0	0	b	a						
436			15		b	a						
437	0	0	0	0	b	a						
438	10	0	0	0	c	a						
439	0	0	0	0	d	a						
440	0	0	0	0	b	a						
441	0	0	0	0	b	a						
442	0	0	0	0	a	a						
443	0	0	0	0	c	a						
444	0	0	0	0	b	a						
445	0	0	0	0	b	a						
446	0	0	0	0	b	a						
447					a	a						
448					a	a						
449					b	a						
450					d	a						
451					d	a						
452					c	a						
453					c	a						

No	Answer 19a (cigarettes)	Answer 19b (cigarettes)	Answer 19c (cigarettes)	Answer 19d (cigarettes)	Answer 20 (a-e)	Answer 21 (a-e)	Answer 22a (yes/no)	Answer 22b (yes/no)	Answer 22c (yes/no)	Answer 23a (yes/no)	Answer 23b (yes/no)	Answer 23c (yes/no)
454					c	a						
455					a	a						
456					b	a						
457					c	a						
458					b	a						
459					c	a						
460					c	a						
461					b	a						
462					a	a						
463					a	a						
464					a	a						
465					b	a						
466	10				b	a						
467					c	a						
468	0	0	0	0	b	a						
469	0	0	0	0	b	a						
470	10	0	0	0	c	a						
471	0	0	0	0	c	a						
472	0	0	0	0	c	a						
473	0	0	0	0	c	a						
474	0	0	0	0	b	a						
475					b	a						
476					c	a						
477						a						

No	Answer 19a (cigarettes)	Answer 19b (cigarettes)	Answer 19c (cigarettes)	Answer 19d (cigarettes)	Answer 20 (a-e)	Answer 21 (a-e)	Answer 22a (yes/no)	Answer 22b (yes/no)	Answer 22c (yes/no)	Answer 23a (yes/no)	Answer 23b (yes/no)	Answer 23c (yes/no)
478					d	a						
