# Supplementary material for: Estimation of Prenatal Alcohol Exposure: Comparison of Retrospective Survey and Measurement of Fatty Acid Ethyl Esters, Ethyl Sulfate, and Ethyl Glucuronide Concentrations in Neonatal Meconium
Source: Toxics. 2026 Feb 4;14(2):155. doi: 10.3390/toxics14020155 (PMC12944540; doi:10.3390/toxics14020155)
Supplement: Supplementary file 1 [file toxics-14-00155-s001.zip › Table S07 answers 24-26.pdf]

Table S7. Results of survey questions 24 to 26 (n=478) in pregnant women conducted at the Neonatology Clinic of the Medical University of Gdańsk in the Pomeranian Province between June 16, 2019, and April 24, 2020.

No	Answer 24 (a-c)	Answer 25 (a-d)	Answer 26a (yes/no)	Answer 26b (yes/no)	Answer 26c (yes/no)	Answer 26d (yes/no)	Answer 26e (yes/no)	Answer 26f (yes/no)	Answer 26g (yes/no)	Answer 26h (yes/no)
1		a	yes	no	no	no	no	no	no	no
2		a	yes	yes	no	no	yes	yes	no	no
3		b	yes	no	no	yes	no	no	no	no
4	a	b	yes	no	no	no	no	no	no	no
5		b	yes	no	no	no	no	no	no	no
6		a	yes	yes	no	no	no	yes	yes	no
7		a	yes	no	yes	no	no	no	no	no
8		b	yes	yes	no	no	no	yes	yes	no
9		a	no	no	yes	no	no	no	no	yes
10		b	yes	no	no	no	no	no	no	no
11		a	yes	no	no	no	no	no	no	no
12		a	yes	no	yes	no	no	no	no	yes
13		b	yes	no	no	yes	no	no	no	no
14		b	yes	no	yes	yes	yes	yes	yes	no
15										
16		b	yes	no	no	no	yes	no	no	no
17		b	no	yes	yes	no	no	yes	yes	no
18		a	yes	no	yes	no	yes	yes	yes	no
19		b	yes	yes	yes	yes	no	no	yes	no
20		b	yes	no	no	yes	no	no	no	no
21										
22		b	yes	no	no	no	no	no	no	no

No	Answer 24 (a-c)	Answer 25 (a-d)	Answer 26a (yes/no)	Answer 26b (yes/no)	Answer 26c (yes/no)	Answer 26d (yes/no)	Answer 26e (yes/no)	Answer 26f (yes/no)	Answer 26g (yes/no)	Answer 26h (yes/no)
23		b	yes	no	yes	yes	yes	yes	no	no
24		b	yes	yes	no	no	no	no	no	yes
25		b	yes	no	yes	yes	yes	no	no	no
26										
27		b	yes	yes	no	no	no	no	no	no
28										
29		a	yes	no	yes	yes	yes	no	no	no
30		a	yes	yes	yes	yes	no	no	yes	no
31										
32		b	yes	no	yes	yes	no	no	no	yes
33		a	yes	no	no	no	no	yes	yes	no
34										
35										
36										
37		b	yes	no	yes	yes	yes	yes	no	no
38		b	no	no	yes	no	no	no	no	no
39		b	yes	yes	yes	no	no	yes	yes	no
40		b	yes	no	no	no	no	no	no	no
41		a	yes	yes	yes	no	no	no	no	no
42	a	b	yes	no	no	no	no	yes	yes	no
43		b	yes	no	yes	yes	no	yes	no	yes
44		b	yes	no	yes	yes	no	yes	no	no
45		a	yes	no	yes	no	yes	no	no	no
46		b	yes	yes	yes	no	no	no	yes	no
47		a	yes	no	yes	no	no	yes	yes	no

No	Answer 24 (a-c)	Answer 25 (a-d)	Answer 26a (yes/no)	Answer 26b (yes/no)	Answer 26c (yes/no)	Answer 26d (yes/no)	Answer 26e (yes/no)	Answer 26f (yes/no)	Answer 26g (yes/no)	Answer 26h (yes/no)
48		b	yes	no	yes	yes	no	yes	yes	no
49		b	no	no	no	no	no	yes	no	no
50		b	yes	no	yes	yes	yes	yes	yes	no
51		b	yes	no	yes	yes	no	yes	no	no
52		b	yes	yes	no	no	no	no	no	no
53		a	yes	no	yes	no	yes	no	no	no
54	c	a	yes	yes	yes	yes	no	yes	yes	no
55		b	no	no	no	no	no	no	no	yes
56		b	yes	yes	no	no	yes	no	yes	no
57		b	yes	no	no	no	no	yes	no	no
58		b	yes	yes	yes	yes	no	yes	yes	no
59		a	yes	yes	yes	no	yes	no	no	no
60		b	yes	no	yes	no	no	yes	yes	no
61		a	yes	no	no	no	no	no	no	yes
62		a	yes	no	yes	no	no	yes	yes	no
63		b	yes	no	no	no	no	yes	no	no
64		a	yes	no	no	no	no	yes	no	no
65		b	yes	yes	yes	yes	no	yes	yes	no
66		a	yes	no	no	yes	yes	no	yes	no
67		b	yes	yes	yes	no	no	no	no	no
68		b	yes	yes	yes	yes	yes	yes	yes	no
69		a	yes	no	no	no	no	no	no	yes
70		a	yes	yes	yes	yes	yes	yes	yes	no
71		a	yes	yes	yes	yes	yes	yes	yes	yes
72		a	yes	yes	yes	yes	yes	yes	yes	yes

No	Answer 24 (a-c)	Answer 25 (a-d)	Answer 26a (yes/no)	Answer 26b (yes/no)	Answer 26c (yes/no)	Answer 26d (yes/no)	Answer 26e (yes/no)	Answer 26f (yes/no)	Answer 26g (yes/no)	Answer 26h (yes/no)
73		a	yes	yes	yes	no	no	yes	no	no
74										
75										
76		b	yes	no	yes	yes	no	no	no	no
77		b	yes	no	yes	no	no	yes	no	no
78		a	yes	yes	yes	yes	yes	yes	no	no
79		a	yes	no	no	no	no	yes	no	no
80		a	yes	no	no	no	no	no	no	yes
81		b	yes	no	no	no	yes	no	no	yes
82		a	yes	yes	yes	yes	no	yes	no	no
83		b	yes	no	yes	yes	yes	yes	no	no
84		b	yes	yes	yes	no	no	no	no	no
85		b	yes	no	yes	no	no	no	yes	no
86		a	yes	no	yes	no	yes	no	no	no
87		b	yes	yes	yes	no	no	yes	yes	no
88		b	no	no	yes	no	no	yes	no	no
89		b	no	no	yes	no	no	no	no	no
90		b	yes	no	yes	no	no	no	no	no
91			yes	no	no	no	no	no	no	no
92										
93		b	yes	no	no	no	no	yes	yes	no
94	a	b	yes	no	no	no	no	yes	no	no
95		b	yes	no	yes	no	no	yes	yes	no
96		a	yes	yes	yes	yes	no	no	no	no
97		a	no	no	yes	no	no	no	no	no

No	Answer 24 (a-c)	Answer 25 (a-d)	Answer 26a (yes/no)	Answer 26b (yes/no)	Answer 26c (yes/no)	Answer 26d (yes/no)	Answer 26e (yes/no)	Answer 26f (yes/no)	Answer 26g (yes/no)	Answer 26h (yes/no)
98		b	yes	yes	yes	no	no	yes	no	no
99		a	yes	yes	yes	yes	yes	yes	yes	no
100		b	yes	no	no	yes	no	no	no	no
101		a	no	no	no	yes	yes	no	no	no
102		a	yes	yes	yes	no	no	yes	yes	yes
103		b	yes	yes	yes	yes	yes	yes	yes	no
104		a	yes	no	no	no	no	no	no	yes
105		b	yes	no	no	yes	no	no	no	no
106		b	yes	no	no	no	yes	yes	no	no
107		a	yes	no	yes	yes	yes	yes	yes	no
108		a	yes	no	yes	no	no	no	no	no
109		b	yes	no	yes	no	yes	yes	no	no
110		a	yes	no	yes	no	yes	yes	yes	no
111		a	yes	yes	yes	no	no	no	no	no
112		a	yes	no	no	no	no	no	no	no
113		b	yes	no	no	no	no	no	no	no
114		b	yes	yes	yes	no	yes	no	no	no
115		a	yes	no	yes	no	yes	no	no	no
116		b	yes	no	no	no	no	no	no	yes
117		b	yes	no	yes	yes	no	no	no	no
118		b	yes	no	yes	yes	no	no	yes	no
119		a	yes	no	no	yes	no	yes	yes	no
120		a	yes	no	yes	yes	no	no	yes	no
121		a	yes	no	no	no	no	yes	no	no
122		a	yes	yes	yes	no	no	no	no	yes

No	Answer 24 (a-c)	Answer 25 (a-d)	Answer 26a (yes/no)	Answer 26b (yes/no)	Answer 26c (yes/no)	Answer 26d (yes/no)	Answer 26e (yes/no)	Answer 26f (yes/no)	Answer 26g (yes/no)	Answer 26h (yes/no)
123		b	yes	no	no	no	yes	yes	no	no
124		b	yes	no	yes	no	yes	no	no	no
125		b	yes	no	no	no	no	yes	no	no
126										
127		b	no	no	no	no	no	no	no	yes
128		b	no	no	no	yes	yes	yes	no	no
129		b	yes	no	yes	yes	no	yes	no	no
130		b	yes	no	yes	no	no	yes	no	no
131		a	yes	no	yes	no	no	no	no	no
132		b	yes	yes	no	no	yes	no	no	no
133										
134		a	yes	no	no	no	no	no	no	no
135		b	yes	no	yes	no	yes	yes	no	no
136		b	yes	no	yes	no	no	no	no	no
137		b	yes	yes	no	no	yes	no	no	no
138		a	no	no	yes	no	no	no	no	yes
139		b	yes	no	yes	no	yes	no	no	no
140		a	yes	yes	yes	no	yes	yes	no	no
141		b	yes	no	yes	no	no	no	no	no
142		b	yes	yes	yes	no	no	yes	yes	no
143		b	yes	no	yes	yes	no	yes	yes	no
144		b	yes	no	yes	yes	no	no	yes	no
145		b	yes	yes	yes	yes	yes	yes	yes	no
146		b	yes	no	no	no	yes	no	no	no
147		b	yes	yes	yes	yes	yes	yes	yes	no

No	Answer 24 (a-c)	Answer 25 (a-d)	Answer 26a (yes/no)	Answer 26b (yes/no)	Answer 26c (yes/no)	Answer 26d (yes/no)	Answer 26e (yes/no)	Answer 26f (yes/no)	Answer 26g (yes/no)	Answer 26h (yes/no)
148		a	yes	no	no	no	no	yes	yes	no
149	a	b	no	no	no	no	no	yes	no	no
150		b	yes	no	yes	yes	yes	no	no	no
151		a	yes	yes	yes	yes	no	no	no	no
152		b	yes	no	no	yes	yes	no	no	no
153		b	yes	no	yes	no	no	yes	no	no
154		n/d	yes	no	no	no	no	no	no	no
155		a	yes	no	yes	yes	no	no	no	no
156		a	yes	yes	no	no	no	no	no	no
157		a	yes	no	yes	yes	no	no	no	no
158		b	yes	no	no	yes	no	yes	no	yes
159		b	yes	yes	yes	no	no	no	no	no
160		b	yes	no	yes	no	yes	no	no	no
161		c	yes	yes	yes	yes	yes	yes	yes	no
162		b								
163			yes	yes	yes	yes	yes	yes	yes	no
164		b	no	no	no	no	no	no	no	yes
165			yes	yes	yes	yes	yes	yes	yes	no
166		b	yes	yes	yes	no	no	no	no	no
167		b	yes	no	yes	no	no	no	yes	no
168		a	yes	yes	yes	no	yes	yes	yes	no
169		b	yes	yes	yes	yes	yes	yes	no	no
170										
171										
172		b	yes	no	yes	no	yes	no	no	no

No	Answer 24 (a-c)	Answer 25 (a-d)	Answer 26a (yes/no)	Answer 26b (yes/no)	Answer 26c (yes/no)	Answer 26d (yes/no)	Answer 26e (yes/no)	Answer 26f (yes/no)	Answer 26g (yes/no)	Answer 26h (yes/no)
173		a	yes	no	yes	no	no	yes	yes	no
174		b	yes	no	yes	no	no	no	no	no
175		b	yes	yes	yes	yes	yes	yes	yes	yes
176		b	yes	no	yes	no	no	yes	no	no
177		b	yes	no	yes	yes	no	yes	no	no
178										
179		b	yes	yes	yes	no	no	yes	yes	no
180										
181		a	yes	no	no	yes	yes	no	no	no
182			no	no	yes	no	no	no	no	no
183		b	yes	yes	yes	no	yes	yes	yes	no
184		b	yes	no	yes	no	no	yes	no	no
185		b	yes	no	yes	no	no	yes	no	no
186	a	b	no	no	yes	yes	yes	no	yes	yes
187		b	yes	no	yes	yes	no	yes	yes	no
188										
189		b	yes	no	yes	no	no	yes	no	no
190		b	yes	no	yes	no	no	no	no	no
191		b	yes	yes	no	no	no	yes	yes	no
192		a	yes	yes	no	no	no	no	no	no
193	yes	a	yes	yes	yes	yes	yes	yes	yes	no
194										
195		b	yes	yes	yes	no	no	no	no	no
196		b	yes	yes	yes	yes	no	yes	yes	no
197		b	no	no	yes	yes	yes	yes	no	no

No	Answer 24 (a-c)	Answer 25 (a-d)	Answer 26a (yes/no)	Answer 26b (yes/no)	Answer 26c (yes/no)	Answer 26d (yes/no)	Answer 26e (yes/no)	Answer 26f (yes/no)	Answer 26g (yes/no)	Answer 26h (yes/no)
198		b	yes	yes	no	no	no	no	no	no
199		b	yes	yes	no	yes	yes	no	yes	no
200		a	yes	yes	yes	no	yes	yes	yes	no
201		a	yes	no	yes	yes	yes	no	no	no
202		b	yes	no	no	no	no	no	no	no
203		b	yes	no	no	yes	yes	no	no	yes
204		b	yes	yes	no	no	no	yes	no	no
205		b	yes	yes	no	no	no	yes	no	no
206		b	yes	yes	yes	no	yes	no	yes	no
207		b	yes	no	yes	no	yes	no	yes	no
208		a	yes	no	no	no	no	no	no	no
209		a	yes	yes	yes	no	yes	yes	no	no
210		b	yes	no	yes	yes	yes	yes	yes	no
211										
212		a	yes	no	yes	no	no	yes	yes	yes
213		b	no	no	no	yes	no	no	no	no
214										
215		b	yes	yes	yes	yes	yes	no	no	no
216		b	yes	yes	yes	yes	yes	yes	no	no
217		a	yes	no	yes	no	no	no	yes	no
218		a	no	no	no	no	no	no	no	yes
219		b	yes	no	yes	no	no	no	no	no
220										
221		b	yes	no	yes	no	no	no	no	no
222										

No	Answer 24 (a-c)	Answer 25 (a-d)	Answer 26a (yes/no)	Answer 26b (yes/no)	Answer 26c (yes/no)	Answer 26d (yes/no)	Answer 26e (yes/no)	Answer 26f (yes/no)	Answer 26g (yes/no)	Answer 26h (yes/no)
223		b	yes	no	yes	no	no	no	yes	no
224		b	yes	yes	yes	yes	yes	yes	yes	no
225		b	yes	yes	yes	yes	yes	yes	yes	yes
226		a	no	yes	yes	no	no	no	no	no
227		b	yes	no	yes	yes	yes	yes	yes	no
228		b	yes	no	yes	no	no	yes	yes	no
229		b	yes	no	no	no	no	no	no	no
230		a	yes	yes	no	no	no	no	no	no
231		a	yes	yes	yes	yes	no	no	no	no
232		b	yes	no	no	no	no	no	no	no
233	a	b	yes	yes	no	no	yes	no	no	no
234		b	yes	yes	yes	no	no	yes	yes	no
235		a	yes	yes	yes	yes	yes	no	no	yes
236										
237	a	b	yes	no	no	no	no	no	yes	no
238		a	yes	no	yes	yes	no	no	no	no
239		a	yes	no	yes	yes	yes	no	no	no
240		b	yes	no	yes	yes	yes	yes	no	no
241		a	yes	yes	no	no	yes	no	no	no
242		b	yes	no	no	no	yes	yes	no	no
243		b	yes	no	yes	no	yes	no	no	no
244	a	b	yes	no	yes	no	yes	yes	no	no
245		b	yes	yes	yes	yes	yes	yes	yes	no
246		b	yes	no	yes	no	yes	yes	yes	no
247		a	yes	yes	yes	yes	no	yes	yes	no

No	Answer 24 (a-c)	Answer 25 (a-d)	Answer 26a (yes/no)	Answer 26b (yes/no)	Answer 26c (yes/no)	Answer 26d (yes/no)	Answer 26e (yes/no)	Answer 26f (yes/no)	Answer 26g (yes/no)	Answer 26h (yes/no)
248		b	yes	no	yes	yes	no	no	no	no
249		b	yes	no	yes	no	no	yes	no	no
250		a	no	no	yes	no	no	yes	yes	no
251		b	yes	yes	no	no	no	no	no	no
252		b	yes	yes	no	no	no	no	no	no
253		a	yes	no	no	no	no	no	no	no
254		b	yes	no	yes	no	no	no	no	no
255		b	yes	no	yes	no	yes	yes	yes	no
256		b	yes	no	no	no	no	no	no	no
257		b	yes	no	yes	no	no	yes	yes	no
258										
259		a	yes	yes	no	no	no	no	no	no
260		a	yes	yes	no	no	no	no	no	no
261		c	yes	no	no	no	no	no	no	no
262										
263		a	yes	no	yes	no	no	no	no	no
264		a	yes	no	yes	no	no	no	no	no
265		b	yes	no	no	no	yes	yes	no	no
266		b	yes	no	no	no	yes	yes	yes	no
267		b	yes	yes	no	no	no	no	no	no
268		b	yes	no	yes	no	no	yes	yes	no
269		b	yes	no	yes	no	no	yes	yes	no
270		b	yes	no	yes	no	no	no	yes	no
271		b	yes	yes	yes	no	yes	yes	no	no
272		a	yes	no	yes	yes	yes	yes	yes	no

No	Answer 24 (a-c)	Answer 25 (a-d)	Answer 26a (yes/no)	Answer 26b (yes/no)	Answer 26c (yes/no)	Answer 26d (yes/no)	Answer 26e (yes/no)	Answer 26f (yes/no)	Answer 26g (yes/no)	Answer 26h (yes/no)
273		a	yes	yes	yes	yes	yes	no	no	no
274										
275										
276		b	yes	no	yes	no	no	no	no	no
277		a	no	no	no	no	no	no	no	no
278	a	b	yes	no	yes	yes	no	yes	no	no
279		b	yes	no	no	no	yes	yes	yes	no
280		a	yes	no	yes	no	no	no	no	no
281		b	yes	no	yes	no	yes	no	no	no
282		a	yes	no	yes	no	yes	yes	yes	no
283		b	yes	yes	yes	yes	no	yes	no	no
284		a	yes	no	yes	no	no	no	no	no
285		b	no	yes	yes	yes	yes	yes	yes	no
286		b	yes	no	no	no	no	yes	yes	no
287										
288										
289		b	yes	no	yes	yes	yes	no	no	no
290		b	yes	yes	yes	no	no	no	no	no
291		a	yes	no	yes	no	yes	yes	no	no
292		b	yes	yes	yes	yes	no	yes	yes	no
293		b	yes	no	yes	no	yes	no	yes	no
294		b	yes	no	no	no	no	no	no	no
295		a	yes	no	no	no	no	no	no	yes
296		b	yes	no	no	no	no	no	no	no
297		b	yes	no	no	no	no	no	yes	no

No	Answer 24 (a-c)	Answer 25 (a-d)	Answer 26a (yes/no)	Answer 26b (yes/no)	Answer 26c (yes/no)	Answer 26d (yes/no)	Answer 26e (yes/no)	Answer 26f (yes/no)	Answer 26g (yes/no)	Answer 26h (yes/no)
298	c	c	yes	no	no	no	no	no	no	no
299		a	yes	no	no	no	no	no	no	no
300		b	yes	no	yes	yes	no	yes	no	no
301		b	yes	yes	yes	yes	yes	no	no	no
302										
303		b	yes	no	no	no	no	no	no	no
304		b	yes	no	no	no	no	no	no	no
305		b	yes	yes	yes	no	no	yes	no	no
306		a	yes	no	yes	yes	no	no	no	no
307		b	yes	no	yes	no	no	no	no	no
308		b	yes	no	yes	no	no	no	no	no
309		b	yes	yes	yes	no	no	yes	no	no
310		b	yes	no	yes	no	yes	no	yes	no
311		b	yes	yes	no	no	no	yes	no	no
312										
313		a	yes	yes	yes	yes	yes	no	no	no
314		b	yes	no	yes	no	no	no	no	yes
315		a	yes	yes	yes	yes	yes	yes	yes	no
316		b	yes	no	yes	no	no	yes	yes	no
317		a	yes	no	no	no	yes	no	no	no
318										
319	n/d	n/d	n/d	n/d	n/d	n/d	n/d	n/d	n/d	n/d
320	n/d	n/d	n/d	n/d	n/d	n/d	n/d	n/d	n/d	n/d
321		c	yes	no	no	no	no	no	no	yes
322		b	no	no	yes	yes	yes	yes	no	no

No	Answer 24 (a-c)	Answer 25 (a-d)	Answer 26a (yes/no)	Answer 26b (yes/no)	Answer 26c (yes/no)	Answer 26d (yes/no)	Answer 26e (yes/no)	Answer 26f (yes/no)	Answer 26g (yes/no)	Answer 26h (yes/no)
323		a	yes	no	yes	no	no	no	no	yes
324		a	yes	no	yes	yes	yes	no	no	no
325		a	yes	no	no	yes	yes	no	no	no
326		a	no	no	yes	yes	no	no	no	no
327		b	yes	no	no	no	no	no	no	no
328		a	yes	yes	yes	yes	no	yes	yes	no
329		b	yes	yes	no	no	no	no	no	yes
330		b	no	no	yes	no	yes	yes	yes	no
331		b	yes	no	yes	no	no	yes	yes	no
332										
333		a	yes	no	yes	no	yes	no	no	no
334										
335		a	yes	yes	yes	yes	no	no	no	no
336		b	yes	no	yes	no	no	no	no	no
337										
338		a	yes	no	yes	no	no	yes	yes	yes
339		b	yes	yes	yes	no	no	no	no	no
340		a	yes	yes	no	no	yes	no	yes	no
341		b	yes	no	yes	yes	no	yes	no	no
342		a	yes	yes	yes	no	no	yes	yes	no
343										
344		b	yes	no	no	yes	no	yes	no	no
345		b	yes	yes	yes	yes	no	no	no	no
346		b	yes	no	no	no	no	no	no	no
347										

No	Answer 24 (a-c)	Answer 25 (a-d)	Answer 26a (yes/no)	Answer 26b (yes/no)	Answer 26c (yes/no)	Answer 26d (yes/no)	Answer 26e (yes/no)	Answer 26f (yes/no)	Answer 26g (yes/no)	Answer 26h (yes/no)
348			yes	no	yes	no	no	no	yes	no
349		b	yes	yes	yes	yes	yes	yes	yes	yes
350		b	yes	no	yes	no	yes	no	no	no
351										
352		b	yes	no	no	no	no	no	no	no
353		a	yes	no	yes	no	no	no	no	yes
354		a	no	no	yes	yes	yes	yes	no	no
355		b	yes	yes	no	no	no	no	no	no
356		a	yes	no	yes	no	yes	no	no	no
357		b	yes	no	no	no	no	no	no	no
358		b	yes	no	no	no	no	no	no	no
359		a	yes	yes	yes	no	no	no	no	yes
360										
361		a	no	no	yes	no	no	no	no	no
362		b	yes	no	yes	no	no	yes	yes	no
363	a	b	yes	no	yes	no	no	yes	yes	no
364		b	yes	no	no	no	yes	no	no	no
365		b	yes	no	yes	yes	yes	no	yes	yes
366		b	yes	no	yes	no	no	no	no	no
367		b	yes	no	no	no	no	no	no	no
368		b	yes	no	yes	no	no	yes	no	no
369		a	yes	no	no	yes	yes	no	no	no
370		b	yes	no	yes	no	yes	no	no	no
371		b	yes	no	yes	yes	yes	no	no	no
372		b	no	yes	yes	no	no	no	no	no

No	Answer 24 (a-c)	Answer 25 (a-d)	Answer 26a (yes/no)	Answer 26b (yes/no)	Answer 26c (yes/no)	Answer 26d (yes/no)	Answer 26e (yes/no)	Answer 26f (yes/no)	Answer 26g (yes/no)	Answer 26h (yes/no)
373		a	yes	yes	no	yes	no	yes	no	no
374		b	yes	yes	yes	yes	yes	no	yes	no
375		a	yes	no	no	no	no	no	no	yes
376		b	yes	no	yes	yes	yes	no	no	no
377		a	yes	yes	yes	yes	no	yes	yes	yes
378		b	yes	no	yes	no	no	no	no	no
379		b	yes	no	yes	no	yes	yes	no	no
380		b	yes	no	no	no	no	yes	yes	no
381		b	no	yes	yes	yes	no	yes	yes	no
382		b	no	no	no	no	no	no	no	yes
383		a	yes	no	no	yes	yes	no	no	no
384		b	yes	no	no	yes	yes	yes	no	no
385		b	yes	no	no	yes	yes	no	no	no
386		b	yes	yes	yes	yes	yes	no	no	no
387		b	yes	no	yes	no	no	no	yes	no
388		a	yes	no	yes	yes	yes	no	no	yes
389		a	no	yes	yes	yes	no	no	no	yes
390	a	a	yes	no	no	no	no	no	no	no
391		b	yes	yes	yes	yes	no	no	no	no
392		b	yes	yes	yes	yes	yes	no	no	no
393		b	yes	yes	yes	no	no	yes	yes	no
394		a	yes	no	yes	no	no	yes	no	no
395		b	yes	yes	yes	yes	yes	yes	no	no
396		a	no	no	yes	no	no	no	no	no
397		b	yes	no	no	no	no	no	yes	no

No	Answer 24 (a-c)	Answer 25 (a-d)	Answer 26a (yes/no)	Answer 26b (yes/no)	Answer 26c (yes/no)	Answer 26d (yes/no)	Answer 26e (yes/no)	Answer 26f (yes/no)	Answer 26g (yes/no)	Answer 26h (yes/no)
398		b	yes	no	no	yes	yes	yes	yes	no
399		b	yes	yes	no	no	no	no	no	no
400		a	yes	no	yes	yes	yes	no	yes	no
401		b	no	yes	yes	no	yes	no	no	no
402		b	yes	no	yes	no	yes	yes	no	no
403		a	yes	no	yes	no	yes	yes	no	no
404		a	yes	no	yes	no	no	no	no	no
405	a	a	yes	yes	no	no	no	no	no	no
406		b	yes	no	yes	no	no	yes	yes	no
407	a	b	yes	yes	no	no	no	no	no	no
408		a	yes	no	yes	yes	yes	no	yes	yes
409		b	yes	yes	no	yes	yes	yes	yes	no
410		b	yes	yes	yes	yes	yes	yes	yes	no
411		a	yes	yes	yes	yes	yes	yes	yes	yes
412		b	no	no	yes	yes	no	no	no	no
413		a	no	no	no	no	no	yes	no	no
414		a	yes	yes	no	no	no	no	no	no
415										
416		a	yes	no	yes	no	no	no	no	yes
417		b	yes	no	yes	yes	yes	yes	yes	no
418		a	yes	yes	yes	yes	yes	yes	yes	no
419		b	yes	no	no	no	yes	no	no	no
420		a	yes	no	yes	yes	yes	yes	yes	yes
421		a	yes	no	yes	yes	yes	yes	yes	yes
422	a	b	no	no	yes	yes	no	no	no	no

No	Answer 24 (a-c)	Answer 25 (a-d)	Answer 26a (yes/no)	Answer 26b (yes/no)	Answer 26c (yes/no)	Answer 26d (yes/no)	Answer 26e (yes/no)	Answer 26f (yes/no)	Answer 26g (yes/no)	Answer 26h (yes/no)
423		b	yes	no	yes	yes	no	no	no	yes
424		b	yes	no	yes	yes	no	no	no	yes
425		b	yes	no	no	no	no	no	no	no
426		a	yes	yes	no	no	yes	yes	no	no
427		b	yes	no	yes	no	no	yes	yes	no
428		b	yes	no	no	yes	yes	no	no	no
429		b	yes	yes	yes	no	yes	yes	yes	no
430		b	yes	no	yes	no	yes	yes	yes	no
431		a	yes	yes	no	no	no	yes	yes	no
432		a	yes	no	yes	yes	yes	no	no	no
433		b	yes	no	yes	no	no	no	yes	no
434		b	yes	no	yes	yes	no	no	no	no
435		b	yes	no	yes	no	no	no	no	no
436		a	no	no	no	no	no	no	no	yes
437		b	yes	no	yes	no	yes	no	yes	no
438		b	yes	no	no	no	yes	no	yes	no
439		a	no	no	no	yes	yes	yes	yes	no
440		b	yes	yes	yes	no	no	no	no	yes
441		a	yes	no	yes	yes	no	yes	yes	no
442		b	yes	no	no	no	no	no	yes	no
443		b	yes	no	yes	no	no	no	no	no
444		b	yes	no	no	no	no	no	yes	no
445		b	yes	no	no	no	no	no	yes	no
446		a	no	no	no	no	no	no	no	yes
447		b	yes	no	yes	no	no	yes	no	no

No	Answer 24 (a-c)	Answer 25 (a-d)	Answer 26a (yes/no)	Answer 26b (yes/no)	Answer 26c (yes/no)	Answer 26d (yes/no)	Answer 26e (yes/no)	Answer 26f (yes/no)	Answer 26g (yes/no)	Answer 26h (yes/no)
448		b	yes	no	no	no	yes	no	no	no
449		b	yes	no	yes	no	yes	no	yes	no
450		b	yes	no	no	yes	no	yes	yes	no
451		b	yes	no	no	yes	no	yes	yes	no
452		b	yes	no	no	yes	no	no	no	no
453		a	yes	no	yes	no	no	no	no	no
454		b	yes	no	no	yes	no	no	yes	no
455		d	yes	no	no	no	no	no	no	no
456		b	yes	yes	yes	yes	yes	yes	yes	yes
457		a	yes	yes	yes	yes	yes	no	no	yes
458		b	yes	no	yes	yes	yes	no	no	no
459		a	yes	no	no	no	no	no	no	no
460		b	yes	no	yes	yes	yes	yes	yes	no
461		a	yes	yes	no	no	no	no	no	yes
462		b	yes	no	yes	no	no	no	yes	no
463		b	yes	no	yes	no	no	yes	yes	no
464		b	yes	no	yes	no	no	yes	yes	no
465		b	yes	yes	yes	yes	yes	yes	yes	yes
466		b	no	no	no	no	no	no	no	yes
467										
468		c	yes	no	yes	no	yes	yes	yes	no
469		a	yes	yes	no	no	no	no	no	yes
470		b	yes	no	yes	yes	yes	yes	no	no
471		b	yes	no	yes	yes	no	yes	yes	no
472		b	yes	yes	yes	no	yes	no	no	no

No	Answer 24 (a-c)	Answer 25 (a-d)	Answer 26a (yes/no)	Answer 26b (yes/no)	Answer 26c (yes/no)	Answer 26d (yes/no)	Answer 26e (yes/no)	Answer 26f (yes/no)	Answer 26g (yes/no)	Answer 26h (yes/no)
473		b	yes	yes	yes	no	yes	no	no	no
474		a	yes	no	yes	yes	yes	no	no	no
475		b	yes	yes	yes	yes	yes	yes	no	no
476		a	yes	yes	yes	yes	yes	yes	yes	yes
477										
478		b	yes	no	yes	yes	yes	yes	yes	no
