# Supplementary material for: Estimation of Prenatal Alcohol Exposure: Comparison of Retrospective Survey and Measurement of Fatty Acid Ethyl Esters, Ethyl Sulfate, and Ethyl Glucuronide Concentrations in Neonatal Meconium
Source: Toxics. 2026 Feb 4;14(2):155. doi: 10.3390/toxics14020155 (PMC12944540; doi:10.3390/toxics14020155)
Supplement: Supplementary file 1 [file toxics-14-00155-s001.zip › Table S08 metric data 1-7g.pdf]

Table S8. Data on pregnant women (personal details) from 1 to 7g (n=478) in a survey conducted at the Neonatology Clinic of the Medical University of Gdańsk in the Pomeranian Province between June 16, 2019, and April 24, 2020.

No	Metric 1 (a-e)	Metric 2 (a-g)	Metric 3 (a-f)	Metric 4 (a-d)	Metric 5 (a-e)	Metric 5e (yes/no)	Metric 6 (a-d)	Metric 7a (yes/no)	Metric 7b (yes/no)	Metric 7c (yes/no)	Metric 7d (yes/no)	Metric 7e (yes/no)	Metric 7f (yes/no)	Metric 7g (yes/no)
1	d	g	b	d	a	no	b	yes	no	no	no	no	no	no
2	d	g	b	a	a	no	b	yes	no	no	no	no	no	no
3	c	e	c	d	d	no	a	no	yes	no	no	no	no	no
4	d	g	b	d	d	no	b	yes	no	no	no	no	no	no
5	e	c	b	a	d	no	b	yes	no	yes	no	no	no	no
6	c	g	b	d	a	no	a	yes	no	yes	no	no	no	no
7	d	f	b	d	a	no	b	yes	no	yes	no	no	no	no
8	d	g	b	d	a	no	b	yes	no	no	no	no	no	no
9	d	g	b	d	a	no	a	yes	no	yes	no	no	no	no
10	e	d	b	a	b	no	b	yes	no	yes	no	no	no	no
11	d	g	b	d	a	no	b	yes	no	no	no	no	no	no
12	d	g	b	a	a	no	a	yes	no	yes	no	no	no	no
13	d	g	b	a	d	no	b	yes	no	yes	no	no	no	no
14	c	g	b	c	a	no	b	yes	no	no	no	no	no	no
15	e	c	e	d		no		no	yes	yes				
16	d	g	b	c	a	no	b	yes	no	no	no	no	no	no
17	d	g	c	d	a	no	a	no	yes	no	no	no	no	no
18	c	g	c	d	a	no	b	no	yes	no	no	no	no	no
19	c	c	b	b	d	no	a	yes	no	yes	yes	no	no	no
20	c	c	a	c	b	no	a	no	yes	yes	no	no	no	no
21	b	n/d	n/d	b	n/d	n/d	n/d	n/d	n/d	n/d	n/d	n/d	n/d	n/d
22	e	c	b	d	b	no	b	yes	no	yes	no	no	no	no

No	Metric 1 (a-e)	Metric 2 (a-g)	Metric 3 (a-f)	Metric 4 (a-d)	Metric 5 (a-e)	Metric 5e (yes/no)	Metric 6 (a-d)	Metric 7a (yes/no)	Metric 7b (yes/no)	Metric 7c (yes/no)	Metric 7d (yes/no)	Metric 7e (yes/no)	Metric 7f (yes/no)	Metric 7g (yes/no)
23	c	g	b	c	a	no	a	yes	no	no	no	no	no	no
24	d	g	b	c	a	no	a	yes	no	yes	no	no	no	no
25	c	e	b	d	e	yes	b	no	yes	no	no	no	no	no
26	b		a	a		yes								
27	e	g	b	a	a	no	b	yes	no	yes	no	no	no	no
28	e	b	b	c	d	no	c	yes	no	yes				
29	d	g	b	b	a	no	a	yes	no	no	no	no	no	no
30	c	g	b	a	e	yes	a	yes	no	yes	no	no	no	no
31	d		b	d		no		yes	no	yes				
32	c	g	b	b	a	no	a	yes	no	no	no	no	no	no
33	c	g	b	d	a	no	a	yes	no	no	no	no	no	no
34	c	f	b	d	a	no		yes	no	no				
35	c	d	a	d	b	no		yes	no	yes				
36	c		a	d				yes	no	yes				
37	c	g	b	b	b	no	a	yes	no	no	no	no	yes, teściowie	no
38	d	g	b	c	a	no	a	yes	no	yes	no	no	no	no
39	d	e	c	d	b	no	b	no	yes	yes	no	no	no	no
40	e	g	b	a	a	no	a	yes	no	no	no	no	no	no
41	d	g	b	d	c	no	a	yes	no	yes	no	no	no	no
42	e	e	b	c	a	no	a	yes	no	yes	no	no	no	no
43	c	c	c	c	b	no	b	no	yes	yes	no	no	no	no
44	b	e	b	b	c	no	a	yes	no	no	no	no	no	no
45	d	g	b	d	a	no	a	yes	no	no	no	no	no	no
46	c	d	c	c	b	no	b	no	yes	no	no	yes	no	no

No	Metric 1 (a-e)	Metric 2 (a-g)	Metric 3 (a-f)	Metric 4 (a-d)	Metric 5 (a-e)	Metric 5e (yes/no)	Metric 6 (a-d)	Metric 7a (yes/no)	Metric 7b (yes/no)	Metric 7c (yes/no)	Metric 7d (yes/no)	Metric 7e (yes/no)	Metric 7f (yes/no)	Metric 7g (yes/no)
47	e	g	b	b	a	no	b	yes	no	yes	no	no	no	no
48	e	g	c	d	a	no	a	no	yes	yes	no	no	no	no
49	b	c	a	c	e	yes	b	no	yes	no	yes	no	no	no
50	c	g	b	a	a	no	b	yes	no	no	no	no	no	no
51	c	f	b	c	d	no	b	yes	no	no	no	no	no	no
52	c	g	b	d	a	no	b	yes	no	no	no	no	no	no
53	d	g	b	c	a	no	b	yes	no	yes	no	no	no	no
54	d	g	b	d	a	no	a	yes	no	yes	no	no	no	no
55	e	f	b	a	c	no	a	yes	no	yes	no	no	no	no
56	c	g	c	d	a	no	b	no	yes	no	no	no	no	no
57	c	g	b	d	a	no	b	yes	no	yes	no	no	no	no
58	d	d	b	a	b	no	b	yes	no	yes	no	no	no	no
59	d	g	b	d	a	no	a	yes	no	yes	no	no	no	no
60	c	e	b	a	d	no	a	yes	no	yes	no	no	no	no
61	c	e	b	d	e	yes	a	yes	no	no	no	no	no	no
62	c	g	b	d	a	no	a	yes	no	yes	no	no	no	no
63	d	g	b	c	a	no	a	yes	no	yes	no	no	no	no
64	e	g	b	b	a	no	a	yes	no	yes	no	no	no	no
65	e	g	a	c	a	no	b	no	yes	yes	no	no	no	no
66	d	g	c	d	a	no	a	no	yes	no	no	no	no	no
67	c	b	b	d	c	no	b	yes	no	no	no	no	no	no
68	d	g	b	c	a	no	b	yes	no	yes	no	no	no	no
69	c	g	b	d	a	no	a	yes	no	no	no	no	no	no
70	d	d	b	b	c	no	a	yes	no	yes	no	no	no	no
71	d	g	b	c	c	no	b	yes	no	yes	no	no	no	no

No	Metric 1 (a-e)	Metric 2 (a-g)	Metric 3 (a-f)	Metric 4 (a-d)	Metric 5 (a-e)	Metric 5e (yes/no)	Metric 6 (a-d)	Metric 7a (yes/no)	Metric 7b (yes/no)	Metric 7c (yes/no)	Metric 7d (yes/no)	Metric 7e (yes/no)	Metric 7f (yes/no)	Metric 7g (yes/no)
72	d	g	b	d	a	no	b	yes	no	yes	no	no	no	no
73	e	g	b	d	a	no	a	yes	no	yes	no	no	no	no
74	c	g	b	a	a	no		yes						
75	c	g	b	a	a	no		yes	no					
76	b	a	a	a	d	no	a	no	yes	yes	no	no	no	no
77	b	f	a	b	e	yes	a	no	yes	no	yes	no	no	no
78	c	g	c											
79	e	g	b	a	a	no	a	yes	no	yes	no	no	no	no
80	c	f	b	c	a	no	a	yes	no	yes	no	no	no	no
81	c	g	b	b	a	no	a	yes	no	yes	no	no	no	no
82	d	f	b	b	b	no	a	yes	no	yes	no	no	no	no
83	c	e	b	d	b	no	c	yes	no	no	no	no	no	no
84	c	g	c	d	a	no	a	no	yes	yes	no	no	no	no
85	d	g	b	c	a	no	a	yes	no	yes	no	no	no	no
86	d	g	b	d	a	no	a	yes	no	yes	no	no	no	no
87	b	f	b	a	e	yes	a	yes	no	no	no	no	no	no
88	e	g	b	a	a	no	b	yes	no	no	no	no	no	no
89	B	d	c	a	d	no	a	no	yes	no	yes	no	no	no
90	d	d	b	a	c	no	a	yes	no	yes	no	no	no	no
91	c	f	b	a		no	a	yes	no	yes	no	no	no	no
92	d	d	b	a	a	no		yes	no					
93	d	g	b	b	a	no	a	yes	no	no	no	no	no	no
94	c	a	b	c	n/d	no	b	no	yes	yes	no	no	no	no
95	c	e	c	d	a	no	b	no	yes	no	yes	no	no	no
96	e	g	b	c	a	no	a	yes	no	yes	no	no	no	no

No	Metric 1 (a-e)	Metric 2 (a-g)	Metric 3 (a-f)	Metric 4 (a-d)	Metric 5 (a-e)	Metric 5e (yes/no)	Metric 6 (a-d)	Metric 7a (yes/no)	Metric 7b (yes/no)	Metric 7c (yes/no)	Metric 7d (yes/no)	Metric 7e (yes/no)	Metric 7f (yes/no)	Metric 7g (yes/no)
97	e	d	b	d	b	no	b	yes	no	yes	no	no	no	no
98	b	d	b	a	d	no	a	yes	no	yes	yes	no	no	no
99	c	g	b	d	a	no	a	yes	no	no	no	no	no	no
100	c	g	b	b	a	no	b	yes	no	no	no	no	no	no
101	c	g	c	b	b	no	a	no	yes	yes	no	no	no	no
102	c	g	b	c	a	no	a	yes	no	yes	no	no	no	no
103	c	g	b	a	a	no	a	yes	no	no	no	no	no	no
104	c	g	b	d	a	no	a	yes	no	yes	no	no	no	no
105	c	g	b	d	a	no	b	yes	no	no	no	no	no	no
106	e	g	b	a	c	no	b	yes	no	yes	no	no	no	no
107	b	f	b	c	e	yes	a	yes	no	no	no	no	no	no
108	c	d	b	a	d	no	a	yes	no	yes	no	no	no	no
109	B	f	b	d	a	no	a	yes	no	no	no	no	no	no
110	d	g	b	d	a	no	a	yes	no	no	no	no	no	no
111	d	g	b	a	a	no	a	yes	no	yes	no	no	no	no
112	e	g	b	c	c	no	a	yes	no	yes	no	no	no	no
113	D	g	b	a	a	no	a	yes	no	yes	no	no	no	no
114	E	g	b	d	a	no	b	yes	no	no	no	no	no	no
115	C	g	b	d	a	no	a	yes	no	yes	yes	yes	no	no
116	C	g	b	d	d	no	b	yes	no	no	no	no	no	no
117	D	e	b	c	a	no	b	yes	no	no	no	no	no	no
118	D	f	b	a	a	no	c	yes	no	yes	no	no	no	no
119	C	e	b	d	d	no	a	yes	no	yes	no	no	no	no
120	D	g	b	a	a	no	b	yes	no	no	no	no	no	no
121	E	g	c	b	d	no	a	no	yes	no	no	no	no	no

No	Metric 1 (a-e)	Metric 2 (a-g)	Metric 3 (a-f)	Metric 4 (a-d)	Metric 5 (a-e)	Metric 5e (yes/no)	Metric 6 (a-d)	Metric 7a (yes/no)	Metric 7b (yes/no)	Metric 7c (yes/no)	Metric 7d (yes/no)	Metric 7e (yes/no)	Metric 7f (yes/no)	Metric 7g (yes/no)
122	d	e	b	d	b	no	b	yes	no	yes	no	no	no	no
123	c	e	b	d	a	no	a	yes	no	no	yes	yes	no	no
124	c	f	b	d	a	no	a	yes	no	no	no	no	no	no
125	B	g	a	d	a	no	b	no	yes	no	no	no	no	no
126	c		a	d		no			yes	no				
127	d	c	a	b	b	no	b	no	yes	yes	no	no	no	no
128	c	f	b	d	a	no	b	yes	no	yes	no	no	no	no
129	c	d	b	a	a	no	a	yes	no	no	no	no	yes (babcia i siostra mamy)	no
130	b	b	c	b	e	yes	a	no	yes	yes	yes	no	no	no
131	D	e	b	c	d	no	c	yes	no	no	no	no	no	no
132	c	g	b	b	a	no	a	yes	no	yes	no	no	no	no
133	b	n/d	a	b	n/d									
134	E	g	b	d	a	no	a	yes	no	yes	no	no	no	no
135	c	g	b	d	a	no	a	yes	no	yes	no	no	no	no
136	E	f	a	d	a	no	a	no	yes	yes	no	no	no	no
137	d	g	c	d	a	no	a	no	yes	no	no	no	no	no
138	c	g	c	d	a	no	a	no	yes	no	no	no	no	no
139	c	g	a	d	a	no	b	no	yes	no	no	no	no	no
140	E	g	b	b	a	no	b	yes	no	yes	no	no	no	no
141	b	c	a	b	e	yes	a	no	yes	no	yes	no	no	no
142	d	g	c	d	a	no	a	no	yes	yes	no	no	no	no
143	e	d	c	d	b	no	b	no	yes	yes	no	no	no	no

No	Metric 1 (a-e)	Metric 2 (a-g)	Metric 3 (a-f)	Metric 4 (a-d)	Metric 5 (a-e)	Metric 5e (yes/no)	Metric 6 (a-d)	Metric 7a (yes/no)	Metric 7b (yes/no)	Metric 7c (yes/no)	Metric 7d (yes/no)	Metric 7e (yes/no)	Metric 7f (yes/no)	Metric 7g (yes/no)
144	e	g	b	d	a	no	a	yes	no	no	no	no	no	no
145	e	g	b	d	a	no	a	yes	no	yes	no	no	no	no
146	d	g	c	d	c	no	a	no	yes	no	no	no	no	no
147	d	g	b	d	c	no	a	yes	no	no	no	no	no	no
148	d	e	e	b	d	no	b	no	yes	no	no	no	no	no
149	e	c	c	c	b	no	c	no	yes	yes	yes	no	no	no
150	b	g	b	d	a	no	a	yes	no	yes	no	no	no	no
151	c	g	b	a	a	no	b	yes	no	no	no	no	no	no
152	C	g	b	d	a	no	a	yes	no	yes	no	no	no	no
153	C	g	b	b	a	no	a	yes	no	yes	yes	yes	no	no
154	C	d	a	d	b	no	d	no	yes	no	no	no	no	no
155	E	g	b	b	a	no	a	yes	no	yes	no	no	no	no
156	D	g	b	d	a	no	a	yes	no	no	no	no	no	no
157	D	d	a	c	a	no	b	no	yes	yes	no	no	no	no
158	C	e	c	a	b	no	a	no	yes	no	yes	no	no	no
159	B	e	c	b	d	no	b	no	no	no	yes	no	no	no
160	C	g	b	a	a	no	a	yes	no	yes	no	no	no	no
161	C	g	b	b	b	no	a	yes	no	no	no	no	no	no
162	E	c	b	a	d	no	b	yes	no	yes	yes	no	no	no
163	D	g	b	d	a,c	no	b	yes	no	yes	no	no	no	no
164	D	b	c	a	d	no	b	no	no	yes	yes	yes	no	no
165	D	g	b	d	a,c	no	b	yes	no	yes	no	no	no	no
166	D	e	b	a	d	no	a	yes	no	yes	no	no	no	no
167	C	e	b	a	b	no	a	yes	no	yes	yes	no	no	no
168	c	g	b	d	a	no	a	yes	no	no	no	no	no	no

No	Metric 1 (a-e)	Metric 2 (a-g)	Metric 3 (a-f)	Metric 4 (a-d)	Metric 5 (a-e)	Metric 5e (yes/no)	Metric 6 (a-d)	Metric 7a (yes/no)	Metric 7b (yes/no)	Metric 7c (yes/no)	Metric 7d (yes/no)	Metric 7e (yes/no)	Metric 7f (yes/no)	Metric 7g (yes/no)
169	C	g	b	d	b	no	b	yes	no	no	no	no	no	no
170	c		a	d	d	no		no	yes					
171	c		a	d	d	no		no	yes					
172	c	g	a	c	a	no	c	no	yes	yes	no	no	no	no
173	e	g	b	d	a	no	a	yes	no	yes	no	no	no	no
174	c	g	c	d	a	no	a	no	yes	yes	no	no	no	no
175	D	d	a	d	b	no	b	no	yes	no	no	no	no	no
176	C	g	b	c	c	no	a	yes	no	yes	yes	no	no	no
177	d	e	b	d	a	no	b	yes	no	no	yes	no	no	no
178	c	g	b	c	a	no		yes	no	yes				
179	E	g	b	d	a	no	a	yes	no	yes	no	no	no	no
180	c		a	d	d	no		no	yes	no				
181	C	g	c	d	a	no	a	no	yes	no	no	no	no	no
182	C	g	a	a	a	no	a	no	yes	yes	no	no	no	no
183	B	f	a	b	a	no	a	no	yes	no	no	no	no	no
184	D	g	b	a	a	no	a	yes	no	yes	no	no	no	no
185	D	g	b	a	a	no	a	yes	no	yes	no	no	no	no
186	D	g	d	d	a	no	c	no	yes	yes	no	no	no	no
187	d	e	e	d	b	no	b	no	yes	yes	no	no	no	no
188	c	g	b	d	a	no		yes	no	no				
189	D	c	b	a	b	no	b	yes	no	yes	yes	no	no	no
190	D	g	b	a	c	no	a	yes	no	yes	no	no	no	no
191	D	d	b	a	a	no	b	yes	no	yes	no	no	no	no
192	C	f	a	b	c	no	a	no	yes	yes	no	no	no	no
193	C	g	b	d	a	no	a	yes	no	no	no	no	no	no

No	Metric 1 (a-e)	Metric 2 (a-g)	Metric 3 (a-f)	Metric 4 (a-d)	Metric 5 (a-e)	Metric 5e (yes/no)	Metric 6 (a-d)	Metric 7a (yes/no)	Metric 7b (yes/no)	Metric 7c (yes/no)	Metric 7d (yes/no)	Metric 7e (yes/no)	Metric 7f (yes/no)	Metric 7g (yes/no)
194	c	g	b	d	a	no		yes	no	no				
195	D	g	b	d	a	no	a	yes	no	yes	no	no	no	no
196	b	d	c	d	b	no	a	no	yes	no	no	no	no	no
197	D	g	b	a	a	no	a	yes	no	yes	no	no	no	no
198	D	g	b	d	a	no	a	yes	no	no	no	no	no	no
199	C	e	b	a	c	no	a	yes	no	no	no	no	no	no
200	d	g	b	d	a	no	a	yes	no	no	no	no	no	no
201	c	g	b	d	a	no	b	yes	no	no	no	no	no	no
202	C	d	b	c	a	no	b	yes	no	no	no	no	no	no
203	E	e	b	d	a	no	b	yes	no	no	no	no	no	no
204	D	f	b	b	c	no	a	yes	no	yes	no	no	no	no
205	D	f	b	b	c	no	a	yes	no	yes	no	no	no	no
206	C	f	c	d	b	no	b	no	yes	no	no	no	no	no
207	C	g	b	d	a	no	a	yes	no	yes	no	no	no	no
208	d	g	b	d	a	no	a	yes	no	yes	no	no	no	no
209	c	g	b	d	a	no	a	yes	no	yes	no	no	no	no
210	d	g	b	a	a	no	a	yes	no	yes	no	no	no	no
211	c	c	b	d	b	no		yes	no	no				
212	D	g	b	a	a	no	b	yes	no	yes	no	no	no	no
213	d	d	b	a	d	no	a	yes	no	yes	no	no	no	no
214	c	c	a	d	a	no		no	yes	no				
215	e	g	b	d	a	no	a	yes	no	no	no	no	no	no
216	B	f	b	d	a	no	a	yes	no	yes	yes	no	no	no
217	d	g	b	d	a	no	a	yes	no	no	no	no	no	no
218	e	g	b	d	a	no	a	yes	no	yes	no	no	no	no

No	Metric 1 (a-e)	Metric 2 (a-g)	Metric 3 (a-f)	Metric 4 (a-d)	Metric 5 (a-e)	Metric 5e (yes/no)	Metric 6 (a-d)	Metric 7a (yes/no)	Metric 7b (yes/no)	Metric 7c (yes/no)	Metric 7d (yes/no)	Metric 7e (yes/no)	Metric 7f (yes/no)	Metric 7g (yes/no)
219	e	f	a	d	c	no	a	no	yes	yes	no	no	no	no
220	d		a	d	a	no		no	yes	yes				
221	d	g	b	d	a	no	a	yes	no	yes	no	no	no	no
222	c	g	b	d	a	no		yes	no	no				
223	D	g	b	d	a	no	b	yes	no	yes	no	no	no	no
224	d	g	c	d	a	no	a	no	yes	no	no	no	no	no
225	c	g	b	d	d	no	a	yes	no	no	no	no	no	no
226	d	g	b	b	a	no	b	yes	no	yes	no	no	no	no
227	e	g	b	b	a	no	b	yes	no	no	no	no	no	no
228	c	g	b	d	a	no	a	yes	no	no	no	yes	no	no
229	c	g	b	a	a,b	no	b	yes	no	no	no	no	no	no
230	c	g	b	d	e	yes	b	yes	no	yes	no	no	no	no
231	d	g	b	d	a	no	a	yes	no	yes	no	no	no	no
232	b	d	a	b	b	no	b	no	yes	no	no	no	no	no
233	d	g	c	d	d	no	c	no	yes	yes	no	no	no	no
234	a	b	c	b	d	yes	a	no	yes	no	yes	yes	no	no
235	c	g	c	d	a	no	b	no	yes	no	no	no	no	no
236	d	g	a	d	a	no		no	yes	yes				
237	e	g	a	d	a	no	b	no	yes	yes	no	no	no	no
238	d	g	a	d	a	no	b	no	yes	no	no	no	no	no
239	b	e	c	d	e	yes	b	no	yes	no	no	no	no	no
240	d	f	a	d	c	no	a	no	yes	no	no	no	no	no
241	e	g	b	d	b	no	a	yes	no	yes	no	no	no	no
242	c	g	c	d	a	no	b	no	yes	no	no	no	no	no
243	c	g	b	d	c	no	a	yes	no	no	no	no	no	no

No	Metric 1 (a-e)	Metric 2 (a-g)	Metric 3 (a-f)	Metric 4 (a-d)	Metric 5 (a-e)	Metric 5e (yes/no)	Metric 6 (a-d)	Metric 7a (yes/no)	Metric 7b (yes/no)	Metric 7c (yes/no)	Metric 7d (yes/no)	Metric 7e (yes/no)	Metric 7f (yes/no)	Metric 7g (yes/no)
244	c	g	b	d	a	no	a	yes	no	no	no	no	no	no
245	d	e	c	b	b	no	b	no	yes	no	no	no	no	no
246	d	e	b	b	a	no	a	yes	no	yes	no	no	no	no
247	e	d	b	a	a	no	a	yes	no	yes	no	no	no	no
248	b	e	c	a	b	no	a	no	yes	no	no	no	no	no
249	c	g	b	c	a	no	a	yes	no	no	no	no	no	no
250	b	d	b	a	a	no	b	yes	no	yes	no	no	no	no
251	d	g	b	d	a	no	a	yes	no	yes	no	no	no	no
252	d	g	b	d	d	no	c	yes	no	yes	no	no	no	no
253	d	g	b	d	c	no	a	yes	no	yes	no	no	no	no
254	e	g	b	b	a	no	a	yes	no	yes	yes	no	no	no
255	b	d	c	c	d	no	c	no	yes	no	no	no	no	no
256	e	d	b	c	d	no	b	yes	no	yes	no	no	no	no
257	e	f	c	d	c	no	b	no	yes	no	no	no	no	no
258	b	f	a	c		yes			yes	no	no			
259	e	e	b	d	d	no	b	yes	no	yes	no	no	no	no
260	d	g	b	c	b	no	a	yes	no	yes	no	no	no	no
261	c	g	b	a	a	no	a	yes	no	no	no	no	no	no
262	e	d	b	a		no		yes	no	yes				
263	d	g	b	b	a	no	b	yes	no	yes	no	no	no	no
264	d	g	b	b	a	no	b	yes	no	yes	no	no	no	no
265	c	g	c	d	a	no	b	no	yes	no	no	no	no	no
266	d	f	b	d	a	no	a	yes	no	yes	no	no	no	no
267	d	g	b	d	c	no	a	yes	no	no	no	no	no	no
268	e	g	a	d	a	no	a	no	yes	no	no	no	no	no

No	Metric 1 (a-e)	Metric 2 (a-g)	Metric 3 (a-f)	Metric 4 (a-d)	Metric 5 (a-e)	Metric 5e (yes/no)	Metric 6 (a-d)	Metric 7a (yes/no)	Metric 7b (yes/no)	Metric 7c (yes/no)	Metric 7d (yes/no)	Metric 7e (yes/no)	Metric 7f (yes/no)	Metric 7g (yes/no)
269	e	g	a	d	a	no	a	no	yes	no	no	no	no	no
270	e	g	b	a	a	no	a	yes	no	yes	no	no	no	no
271	e	e	e	c	b	no	a	no	no	no	yes	no	no	no
272	d	f	b	d	a	no	a	yes	no	no	no	no	no	no
273	d	g	b	d	a	no	a	yes	no	no	no	no	no	no
274	d	d	b	b	b	no		yes	no	no				
275	e	f	a	c	n/d									
276	d	g	b	d	a	no	a	yes	no	yes	no	no	no	no
277	e	e	b	a	c	no	b	yes	no	yes	no	no	no	no
278	e	g	b	d	a	no	a	yes	no	yes	no	no	no	no
279	d	g	b	d	a	no	b	yes	no	yes	no	no	no	no
280	e	g	c	d	a	no	a	no	yes	yes	no	no	no	no
281	c	f	b	a	a	no	b	yes	no	yes	no	no	yes	no
282	d	g	a	d	a	no	b	no	yes	no	no	no	no	no
283	b	g	b	a	a	no	b	yes	no	yes	yes	no	no	no
284	d	g	b	a	b	no	a	yes	no	yes	no	no	no	no
285	c	f	b	d	a	no	a	yes	no	no	no	no	no	no
286	c	d	b	d	a	no	b	no	yes	no	no	no	no	no
287	c	n/d	a	d	d	no		no	yes	yes				
288	c	n/d	a	d	d	no		no	yes	yes				
289	c	f	b	c	a	no	a	yes	no	no	yes	no	no	no
290	c	g	b	a	a	no	a	yes	no	no	no	no	no	no
291	e	d	b	c	b	no	b	yes	no	no	no	no	no	no
292	d	f	b	c	d	no	a	yes	no	yes	no	no	no	no
293	d	g	b	d	a	no	a	yes	no	yes	no	no	no	no

No	Metric 1 (a-e)	Metric 2 (a-g)	Metric 3 (a-f)	Metric 4 (a-d)	Metric 5 (a-e)	Metric 5e (yes/no)	Metric 6 (a-d)	Metric 7a (yes/no)	Metric 7b (yes/no)	Metric 7c (yes/no)	Metric 7d (yes/no)	Metric 7e (yes/no)	Metric 7f (yes/no)	Metric 7g (yes/no)
294	c	e	b	b	a	no	b	yes	no	no	no	no	no	no
295	c	g	c	c	a	no	a	no	yes	no	no	no	no	no
296	c	g	b	d	a	no	a	yes	no	yes	no	no	no	no
297	c	e	b	d	b	no	b	yes	no	no	no	no	no	no
298	d	e	b	b	d	no	a	yes	no	yes	no	no	no	no
299	d	g	b	d	a	no	a	yes	no	yes	no	no	no	no
300	b	b	c	c	e	yes	b	no	yes	no	no	no	no	no
301	d	g	b	a	a	no	a	yes	no	yes	no	no	no	no
302	e	g	b	a	a	no		yes	no	yes				
303	c	d	b	d	b	no	a	yes	no	no	no	no	no	no
304	c	d	b	d	b	no	a	yes	no	no	no	no	no	no
305	c	g	c	b	b	no	c	no	yes	yes	no	no	yes	no
306	c	d	b	a	d	no	a	yes	no	yes	no	no	no	no
307	c	g	c	c	a	no	b	no	yes	yes	no	no	no	no
308	e	e	b	b	a	no	a	yes	no	yes	no	no	no	no
309	e	g	b	a	a	no	a	yes	no	no	no	no	no	no
310	c	g	b	d	c	no	b	yes	no	no	no	no	no	no
311	c	f	c	d	a	no	a	no	yes	yes	no	no	no	no
312	c	c	a	d	b	no		no	yes	no				
313	c	g	b	c	a	no	a	yes	no	no	no	no	no	no
314	e	g	b	b	c	no	b	yes	no	yes	no	no	no	no
315	e	g	b	d	a	no	a	yes	no	no	no	no	no	no
316	e	g	b	c	a	no	a	yes	no	no	no	no	no	no
317	e	g	b	d	a	no	a	yes	no	yes	no	no	no	no
318	d	c	b	b	b	no		yes	no	yes				

No	Metric 1 (a-e)	Metric 2 (a-g)	Metric 3 (a-f)	Metric 4 (a-d)	Metric 5 (a-e)	Metric 5e (yes/no)	Metric 6 (a-d)	Metric 7a (yes/no)	Metric 7b (yes/no)	Metric 7c (yes/no)	Metric 7d (yes/no)	Metric 7e (yes/no)	Metric 7f (yes/no)	Metric 7g (yes/no)
319	n/d	n/d	n/d	b	n/d	n/d	n/d	n/d	n/d	n/d	n/d	n/d	n/d	
320	n/d	n/d	n/d	b	n/d	n/d	n/d	n/d	n/d	n/d	n/d	n/d	n/d	
321	e	g	b	c	b	no	c	yes	no	yes	no	no	no	no
322	c	g	b	d	a	no	a	yes	no	no	no	no	no	no
323	d	g	b	c	c	no	a	yes	no	no	no	no	no	no
324	c	g	b	d	a	no	a	yes	no	no	no	no	no	no
325	c	d	b	d	b	no	b	yes	no	yes	yes	no	no	no
326	c	c	b	b	d	no	b	yes	no	yes	no	no	no	no
327	c	f	b	b	c	no	a	yes	no	no	no	no	no	no
328	d	g	b	c	c	no	a	yes	no	no	no	no	no	no
329	d	g	b	c	a	no	b	yes	no	no	no	no	no	no
330	e	g	c	d	a	no	b	no	yes	yes	yes	no	no	no
331	c	g	b	a	a	no	a	yes	no	no	no	no	no	no
332	c	e	b	d	a	no		yes	no	yes				
333	d	g	b	d	a	no	b	yes	no	yes	no	no	no	no
334	e	n/d	b	d	d	no		yes	no	yes				
335	e	g	b	c	a	no	a	yes	no	yes	no	no	no	no
336	d	f	b	d	a	no	b	yes	no	yes	no	no	no	no
337	d	f	a	b										
338	b	b	c	d	d	no	a	no	yes	yes	no	no	no	no
339	e	g	b	d	a	no	b	yes	no	yes	no	no	no	no
340	c	g	b	b	a	no	a	yes	no	no	no	no	no	no
341	c	g	b	d	a	no	b	yes	no	no	no	no	no	no
342	b	d	a	d	c	no	b	no	no	yes	no	no	no	no
343	c	f	a	c	a	no		no	yes	no				

No	Metric 1 (a-e)	Metric 2 (a-g)	Metric 3 (a-f)	Metric 4 (a-d)	Metric 5 (a-e)	Metric 5e (yes/no)	Metric 6 (a-d)	Metric 7a (yes/no)	Metric 7b (yes/no)	Metric 7c (yes/no)	Metric 7d (yes/no)	Metric 7e (yes/no)	Metric 7f (yes/no)	Metric 7g (yes/no)
344	b	c	c	c	d	no	b	no	yes	yes	no	no	no	no
345	c	g	b	c	a	no	b	yes	no	yes	no	no	no	no
346	d	g	b	d	c	no	b	yes	no	yes	no	no	no	no
347	e	d	a	d	b	no		no	yes	no				
348	d	g	b	a	a	no	a	yes	no	yes	no	no	no	no
349	e	g	b	b	a	no	b	yes	no	yes	no	no	no	no
350	C	g	b	d	a	no	b	yes	no	no	yes	yes	no	no
351	d	f	b	d	c	no		yes	no	no				
352	c	g	b	d	a	no	a	yes	no	yes	no	no	no	no
353	e	g	b	a	a	no	b	yes	no	yes	no	no	no	no
354	d	f	c	d	d	no	a	no	yes	no	no	no	no	no
355	d	e	b	a	c	no	c	yes	no	yes	no	no	no	no
356	c	e	b	d	a	no	a	yes	no	no	no	no	no	no
357	d	d	b	c	d	no	a	yes	no	yes	no	no	no	no
358	d	d	b	c	d	no	a	yes	no	yes	no	no	no	no
359	d	g	a	c	a	no	a	no	yes	no	no	no	no	no
360	c	e	a	d	a	no		no	yes	no				
361	d	g	b	c	a	no	b	yes	no	yes	no	no	no	no
362	c	e	a	d	a	no	a	no	yes	no	yes	no	no	no
363	b	e	a	a	a	no	a	no	yes	no	no	no	no	no
364	c	g	c	d	b	no	b	no	yes	yes	no	no	no	no
365	d	g	c	d	a	no	a	no	yes	no	no	no	no	no
366	c	c	b	a	d	no	c	yes	no	yes	no	no	no	no
367	c	c	b	a	d	no	c	yes	no	yes	no	no	no	no
368	d	g	b	c	c	no	b	yes	no	yes	no	no	no	no

No	Metric 1 (a-e)	Metric 2 (a-g)	Metric 3 (a-f)	Metric 4 (a-d)	Metric 5 (a-e)	Metric 5e (yes/no)	Metric 6 (a-d)	Metric 7a (yes/no)	Metric 7b (yes/no)	Metric 7c (yes/no)	Metric 7d (yes/no)	Metric 7e (yes/no)	Metric 7f (yes/no)	Metric 7g (yes/no)
369	b	d	b	d	d	no	b	yes	no	no	no	no	no	no
370	c	g	b	d	a	no	a	yes	no	yes	no	no	no	no
371	d	d	b	b	b	no	a	yes	no	no	no	no	no	no
372	e	g	b	d	a	no	a	yes	no	yes	no	no	no	no
373	e	c	b	c	b	no	a	yes	no	yes	no	no	no	no
374	c	g	a	d	a	no	b	no	yes	no	no	no	no	no
375	c	e	b	b	b/c	no	a	yes	no	no	no	no	no	no
376	d	c	b	b	b	no	b	yes	no	no	no	no	no	no
377	c	g	c	b	a	no	a	no	yes	no	no	no	no	no
378	d	g	b	d	a,c	no	b	yes	no	yes	no	no	no	no
379	e	g	b	c	a	no	b	yes	no	yes	no	no	no	no
380	d	c	b	a	d	no	b	yes	no	yes	no	no	no	no
381	d	g	d	b	c	no	a	no	yes	yes	no	no	no	no
382	e	g	b	d	a	no	a	yes	no	yes	no	no	no	no
383	C	f	b	d	d	no	b	yes	no	no	no	no	no	no
384	C	e	b	b	a	no	b	yes	no	no	yes	no	no	no
385	C	f	c	d	d	no	c	no	yes	no	no	no	no	no
386	d	d	b	b	d	no	b	yes	no	yes	yes	no	no	no
387	e	g	b	d	a	no	a	yes	no	yes	no	no	no	no
388	C	f	b	d	a	no	b	yes	no	no	no	no	no	no
389	d	g	b	d	a	no	a	yes	no	no	no	no	no	no
390	c	g	b	d	c	no	a	yes	no	no	no	no	no	no
391	d	d	b	a	c	no	a	yes	no	yes	no	no	no	no
392	e	g	b	d	a	no	a	yes	no	yes	no	no	no	no
393	e	f	b	c	a	no	b	yes	no	yes	no	no	no	no

No	Metric 1 (a-e)	Metric 2 (a-g)	Metric 3 (a-f)	Metric 4 (a-d)	Metric 5 (a-e)	Metric 5e (yes/no)	Metric 6 (a-d)	Metric 7a (yes/no)	Metric 7b (yes/no)	Metric 7c (yes/no)	Metric 7d (yes/no)	Metric 7e (yes/no)	Metric 7f (yes/no)	Metric 7g (yes/no)
394	d	g	b	d	c	no	b	yes	no	yes	no	no	no	no
395	c	e	b	d	b	no	a	yes	no	no	no	no	no	no
396	d	g	b	d	c	no	b	yes	no	yes	no	no	no	no
397	d	g	b	d	a	no	a	yes	no	yes	no	no	no	no
398	c	d	b	a	a	no	b	yes	no	no	no	no	no	no
399	e	g	b	a	a	no	b	yes	no	no	no	no	no	no
400	c	g	b	d	a	no	a	yes	no	no	no	no	no	no
401	d	g	b	a	d	no	a	yes	no	yes	no	no	no	no
402	c	g	b	d	a	no	a	yes	no	no	no	no	no	no
403	d	d	c	c	b	no	b	no	yes	no	no	no	no	no
404	e	g	b	d	a	no	a	yes	no	yes	no	no	no	no
405	e	g	b	d	d	no	b	yes	no	no	no	no	no	no
406	e	g	b	d	a	no	a	yes	no	no	no	no	no	no
407	e	g	b	d	a	no	b	yes	no	yes	no	no	no	no
408	c	f	a	a	d	no	a	no	yes	yes	no	no	no	no
409	d	g	b	d	a	no	a	yes	no	no	no	no	no	no
410	d	g	b	d	a	no	a	yes	no	no	no	no	no	no
411	e	e	b	c	c	yes	a	yes	no	no	no	no	no	no
412	d	a	c	a	d	no	b	no	yes	yes	no	no	yes	no
413	e	g	b	c	a,c	no	b	yes	no	yes	no	no	no	no
414	e	g	b	b	a	a		no	yes	no	no	no	no	no
415	d	d	b	a	c	no		yes	no	yes				
416	d	d	b	a	a	yes	a	yes	no	yes	no	no	no	no
417	b	g	b	d	a	no	a	yes	no	no	no	no	no	no
418	e	g	b	a	a	no	b	yes	no	yes	no	no	no	no

No	Metric 1 (a-e)	Metric 2 (a-g)	Metric 3 (a-f)	Metric 4 (a-d)	Metric 5 (a-e)	Metric 5e (yes/no)	Metric 6 (a-d)	Metric 7a (yes/no)	Metric 7b (yes/no)	Metric 7c (yes/no)	Metric 7d (yes/no)	Metric 7e (yes/no)	Metric 7f (yes/no)	Metric 7g (yes/no)
419	c	g	b	d	a,b	no	a	yes	no	no	no	no	no	no
420	c	f	a	d	a	no	b	no	yes	no	no	no	no	no
421	c	f	a	d	a	no	b	no	yes	no	no	no	no	no
422	e	g	b	a	a,c	no	a	yes	no	yes	no	no	no	no
423	c	g	b	b	d	no	b	yes	no	yes	yes	no	yes	no
424	c	g	b	b	d	no	b	yes	no	yes	yes	no	yes	no
425	e	g	b	d	a	no	b	yes	no	yes	no	no	no	no
426	c	c	a	c	b	no	a	no	yes	no	no	no	no	no
427	c	d	b	a	d	no	b	yes	no	yes	no	no	no	no
428	c	g	b	d	a	no	a	yes	no	yes	no	no	no	no
429	c	g	b	b	a	no	c	yes	no	no	no	no	no	no
430	c	g	b	a	a	no	b	yes	no	no	no	no	no	no
431	d	g	c	c	c	no	a	no	yes	no	no	no	no	no
432	c	g	b	d	a	no	a	yes	no	yes	no	no	no	no
433	d	g	b	a	a	no	a	yes	no	yes	yes	no	no	no
434	c	g	b	b	a	no	a	yes	no	no	no	no	no	no
435	c	g	b	d	d	no	a	yes	no	yes	no	no	no	no
436	C	b	b	a	d	no	b	yes	no	yes	no	no	no	no
437	d	g	b	d	a	no	b	yes	no	yes	no	no	no	no
438	c	g	a	d	c	no	b	no	no	no	no	no	no	yes
439	c	g	c	c	d	no	c	no	yes	no	no	no	no	no
440	e	g	b	a	d	no	b	yes	no	yes	no	no	no	no
441	c	g	b	a	a	no	a	yes	no	yes	yes	no	no	no
442	c	c	b	a	d	no	a	yes	no	yes	no	no	no	no
443	c	g	b	d	e	yes	a	yes	no	no	no	no	no	no

No	Metric 1 (a-e)	Metric 2 (a-g)	Metric 3 (a-f)	Metric 4 (a-d)	Metric 5 (a-e)	Metric 5e (yes/no)	Metric 6 (a-d)	Metric 7a (yes/no)	Metric 7b (yes/no)	Metric 7c (yes/no)	Metric 7d (yes/no)	Metric 7e (yes/no)	Metric 7f (yes/no)	Metric 7g (yes/no)
444	b	e	b	a	b	no	b	yes	no	no	no	no	no	no
445	b	e	b	a	b	no	b	yes	no	no	no	no	no	no
446	e	g	a	d	a	no	a	no	yes	no	no	no	no	no
447	A	b	a	b	d	yes	b	no	no	no	yes	yes	no	no
448	C	g	a	d	d	no	b	no	yes	no	no	no	no	no
449	C	g	b	d	a	no	a	yes	no	yes	no	no	no	no
450	E	g	b	a	c	no	b	yes	no	yes	no	no	no	no
451	E	g	b	a	c	no	b	yes	no	yes	no	no	no	no
452	B	d	a	b	b	no	b	no	yes	no	yes	no	no	no
453	D	g	b	d	a	no	a	yes	no	no	no	no	no	no
454	C	e	b	b	b	no	b	yes	no	no	no	no	no	no
455	B	c	b	a	d	no	a	yes	no	yes	yes	yes	no	no
456	D	f	b	a	b	no	a	yes	no	yes	yes	yes	yes	no
457	C	g	b	a	a	no	a	yes	no	no	no	no	no	no
458	C	g	b	d	a	no	a	yes	no	no	no	no	no	no
459	C	f	b	c	a	no	a	yes	no	no	no	no	no	no
460	C	g	b	d	a	no	b	yes	no	no	no	no	no	no
461	D	g	b	d	a	no	a	yes	no	yes	no	no	no	no
462	E	c	b	b	d	no	b	yes	no	no	no	no	no	no
463	D	e	b	a	c	no	b	yes	no	yes	no	no	no	no
464	D	e	b	a	c	no	b	yes	no	yes	no	no	no	no
465	E	g	d	a	a	no	b	no	yes	no	no	no	no	no
466	E	c	c	d	d	no	b	no	yes	yes	no	no	no	no
467	C	g	b			no	b	yes	no	no	no	no	no	no
468	c	g	b	d	a	no		no	no	no	no	no	no	no

No	Metric 1 (a-e)	Metric 2 (a-g)	Metric 3 (a-f)	Metric 4 (a-d)	Metric 5 (a-e)	Metric 5e (yes/no)	Metric 6 (a-d)	Metric 7a (yes/no)	Metric 7b (yes/no)	Metric 7c (yes/no)	Metric 7d (yes/no)	Metric 7e (yes/no)	Metric 7f (yes/no)	Metric 7g (yes/no)
469	c	g	b	d	a,b,c	no	a	yes	no	yes	no	no	no	no
470	e	c	a	a	b	no	b	no	yes	no	no	no	no	no
471	b	d	b	a	b	no	a	yes	no	no	yes	no	yes	no
472	c	f	b	c	a	no	b	yes	no	no	no	no	no	no
473	c	f	b	c	a	no	b	yes	no	no	no	no	no	no
474	c	g	b	d	a	no	a	yes	no	no	no	no	no	no
475	C	g	b	c	a	no	a	yes	no	no	no	no	no	no
476	c	g	c	d	a	no	a	no	yes	no	no	no	no	no
477	d	f	a	d	a	no		no	yes	no	no	no	no	no
478	C	g	c	d	a	no	a	no	yes	no	no	no	no	no
