# Supplementary material for: Estimation of Prenatal Alcohol Exposure: Comparison of Retrospective Survey and Measurement of Fatty Acid Ethyl Esters, Ethyl Sulfate, and Ethyl Glucuronide Concentrations in Neonatal Meconium
Source: Toxics. 2026 Feb 4;14(2):155. doi: 10.3390/toxics14020155 (PMC12944540; doi:10.3390/toxics14020155)
Supplement: Supplementary file 1 [file toxics-14-00155-s001.zip › Table S09 chemical analysis results in mass concentration.pdf]

Table S9. Results of GC-MS-SIM and LC-ESI-MS-MS chemical analyses (in mass concentration units - µg/g) for infant tar (n=478) collected on the first day after birth at the Neonatology Clinic of the Medical University of Gdańsk in the Pomeranian Province between June 16, 2019, and April 24, 2020.

No	EE 12:0 (µg/g)	EE 14:0 (µg/g)	EE 16:0 (µg/g)	EE 18:2 (µg/g)	EE 18:1 (µg/g)	EE 18:3 (µg/g)	EE 18:0 (µg/g)	EE 20:4 (µg/g)	EE 20:0 (µg/g)	Total FAEE (µg/g)	EtS (µg/g)	EtG (µg/g)
1										0,00	0,00	
2	0,65	0,73	2,01		2,00	4,36	0,26		0,09	10,09		0,03
3				0,14						0,14		0,00
4	0,01	0,07	0,90	0,54	0,16		0,09	0,84		2,61		0,01
5				0,03						0,03		
6										0,00		
7			0,01	0,05	0,01					0,07	0,00	0,00
8		0,01	0,14	0,09	0,02		0,02	0,15		0,43	0,01	0,05
9										0,00		
10										0,00	0,00	
11										0,00	0,00	
12								0,03		0,03	0,00	
13										0,00	0,00	
14		0,11		0,40		0,03			0,01	0,54	0,01	0,02
15										0,00		
16	0,01	0,05	0,29	0,97	1,46	0,11	0,02	0,20	0,00	3,12	0,05	0,97
17			0,00							0,00		0,01
18			0,00				0,00			0,00	0,00	
19										0,00		
20				0,06						0,06		
21		0,01	0,03	0,34	0,02	0,02	0,01	0,35		0,77	0,00	0,00
22										0,00		
23										0,00		
24				0,11	0,01					0,12	0,01	0,00

No	EE 12:0 (µg/g)	EE 14:0 (µg/g)	EE 16:0 (µg/g)	EE 18:2 (µg/g)	EE 18:1 (µg/g)	EE 18:3 (µg/g)	EE 18:0 (µg/g)	EE 20:4 (µg/g)	EE 20:0 (µg/g)	Total FAEE (µg/g)	EtS (µg/g)	EtG (µg/g)
25										0,00		
26				0,04	0,01					0,05	0,01	
27										0,00		
28				0,03				0,03		0,06		0,02
29	0,68	0,52	1,65		8,72	2,33	0,22		0,03	14,15	0,01	0,06
30										0,00		
31			0,01	0,09	0,01			0,06		0,17		
32				0,29				0,11		0,40		0,00
33										0,00		
34				0,29	0,00					0,30		
35			0,01	0,21			0,00	0,07		0,30		
36										0,00	0,02	0,01
37										0,00		0,00
38										0,00		
39				0,12		0,01				0,13	0,03	0,14
40				0,21						0,21		
41										0,00	0,00	0,64
42										0,00		
43										0,00	0,00	
44	0,01		0,02		0,00	0,02		0,22		0,28	0,00	
45										0,00		
46				0,53	0,04	0,01		0,10		0,68	0,00	0,00
47										0,00	0,00	
48										0,00	0,00	
49										0,00	0,00	
50										0,00		

No	EE 12:0 (µg/g)	EE 14:0 (µg/g)	EE 16:0 (µg/g)	EE 18:2 (µg/g)	EE 18:1 (µg/g)	EE 18:3 (µg/g)	EE 18:0 (µg/g)	EE 20:4 (µg/g)	EE 20:0 (µg/g)	Total FAEE (µg/g)	EtS (µg/g)	EtG (µg/g)
51				0,70	0,03			0,40		1,12		0,01
52				0,05						0,05		
53				0,03	0,00			0,03		0,06	0,02	0,01
54				0,26						0,26		
55				0,44				0,69		1,14	0,00	0,01
56										0,00		
57		0,01	0,06	0,54	0,05	0,00		0,47	0,02	1,15		0,02
58										0,00		0,01
59										0,00	0,01	0,01
60	0,45	0,44	0,70	2,28	0,69	0,37	0,04	1,85		6,83		0,01
61				0,28	0,00	0,01		0,08	0,00	0,37		0,00
62				0,08	0,01	0,00				0,09		
63										0,00		
64	0,08	0,05	0,07	2,09	0,21	0,29	0,02	0,42		3,22		
65				0,23		0,01				0,24		
66		0,00	0,07	1,33	0,87	0,17	0,01	0,29		2,74	0,01	0,01
67										0,00		
68			0,01		0,01		0,03		0,00	0,05	0,09	4,62
69					0,01					0,01	0,00	
70										0,00	0,00	
71				0,01						0,01		
72										0,00		
73										0,00	0,00	
74			0,01	0,03	0,08	0,01	0,00	0,03		0,16	0,00	0,01
75			0,01	0,67	0,03	0,02		0,01		0,74		0,01
76			0,01	0,09	0,03	0,02				0,14	0,00	

No	EE 12:0 (µg/g)	EE 14:0 (µg/g)	EE 16:0 (µg/g)	EE 18:2 (µg/g)	EE 18:1 (µg/g)	EE 18:3 (µg/g)	EE 18:0 (µg/g)	EE 20:4 (µg/g)	EE 20:0 (µg/g)	Total FAEE (µg/g)	EtS (µg/g)	EtG (µg/g)
77								0,05		0,05		0,01
78		0,00	0,05	0,24	0,41	0,03			0,00	0,73		0,00
79										0,00		
80										0,00		
81			0,00							0,00	0,00	0,01
82										0,00		
83		0,00	0,03	0,62	0,18	0,05		2,07		2,95	0,00	
84								0,17		0,17	0,00	
85				0,44	0,01			0,38		0,83		
86										0,00		
87				0,01						0,01		0,00
88				0,04				0,04		0,08	0,00	
89			0,00				0,00			0,00	0,01	0,00
90				0,21		0,01				0,22		0,02
91			0,03	0,23	0,09	0,02				0,37	0,00	
92		0,00	0,01	0,06	0,22		0,01	0,31		0,60		0,09
93			0,01	0,31	0,06			0,14		0,51	0,00	0,01
94			0,01	0,53	0,03			0,09		0,66	0,00	0,00
95										0,00		
96		0,01	0,11	0,40	0,57	0,06	0,01	0,25		1,40		0,01
97			0,00		0,01					0,01	0,00	0,01
98				0,03				0,17		0,20		
99										0,00		0,00
100										0,00		
101										0,00		0,01
102										0,00		

No	EE 12:0 (µg/g)	EE 14:0 (µg/g)	EE 16:0 (µg/g)	EE 18:2 (µg/g)	EE 18:1 (µg/g)	EE 18:3 (µg/g)	EE 18:0 (µg/g)	EE 20:4 (µg/g)	EE 20:0 (µg/g)	Total FAEE (µg/g)	EtS (µg/g)	EtG (µg/g)
103			0,05	0,30	0,24		0,00	0,24		0,83	0,01	0,01
104										0,00		
105					0,01	0,03		0,16		0,21		
106								0,26		0,26		
107										0,00	0,00	
108										0,00		
109										0,00		
110			0,03	0,07	0,11		0,01	0,15		0,38		0,00
111			0,01	0,24		0,01	0,00			0,25	0,00	
112	0,02	0,05	0,53	2,23	2,14	0,26	0,15	0,83	0,05	6,27	0,00	0,02
113		0,02	0,12	0,35	0,87	0,08	0,01	0,43	0,01	1,89		
114				0,01	0,01		0,00	0,14		0,16		0,01
115										0,00		
116										0,00		
117				0,06				0,86		0,92	0,00	
118										0,00		
119			0,01	0,04	0,02			0,04		0,11		
120			0,01	0,08	0,02	0,01	0,00			0,12	0,00	0,00
121				0,12	0,02					0,14		
122				0,01						0,01		0,00
123			0,01		0,01					0,02	0,01	0,54
124										0,00		
125										0,00		
126	0,00		0,01	0,01	0,02			0,09		0,13	0,00	0,02
127										0,00		
128								0,03		0,03	0,00	0,00

No	EE 12:0 (µg/g)	EE 14:0 (µg/g)	EE 16:0 (µg/g)	EE 18:2 (µg/g)	EE 18:1 (µg/g)	EE 18:3 (µg/g)	EE 18:0 (µg/g)	EE 20:4 (µg/g)	EE 20:0 (µg/g)	Total FAEE (µg/g)	EtS (µg/g)	EtG (µg/g)
129				0,06						0,06	0,00	
130			0,00		0,00					0,01		0,02
131										0,00		
132				0,15		0,01		0,18		0,35	0,00	0,02
133			0,01				0,00			0,01		
134	0,00		0,01	0,19	0,06	0,02		0,12		0,40		0,00
135										0,00	0,00	
136										0,00		0,01
137										0,00	0,00	
138								0,22		0,22	0,01	0,02
139										0,00		
140		0,00	0,02	0,20	0,07	0,01	0,00	0,21		0,53	0,02	0,06
141		0,02	0,26	0,34	0,70	0,07	0,04	0,30	0,00	1,74	0,00	0,07
142			0,00							0,00	0,00	
143			0,00		0,02			0,05		0,07	0,00	
144			0,00	0,03	0,01					0,04	0,01	0,01
145										0,00		
146										0,00	0,03	0,00
147										0,00		
148				0,04						0,04	0,00	
149								0,25		0,25	0,00	
150										0,00	0,00	
151										0,00		
152										0,00	0,00	
153					0,01				0,00	0,01	0,00	0,00
154	0,02		0,01	0,57	0,03	0,05		0,28		0,96	0,00	0,00

No	EE 12:0 (µg/g)	EE 14:0 (µg/g)	EE 16:0 (µg/g)	EE 18:2 (µg/g)	EE 18:1 (µg/g)	EE 18:3 (µg/g)	EE 18:0 (µg/g)	EE 20:4 (µg/g)	EE 20:0 (µg/g)	Total FAEE (µg/g)	EtS (µg/g)	EtG (µg/g)
155				0,05						0,05		
156										0,00		
157			0,00							0,00		0,01
158										0,00	0,00	
159				0,01						0,01		
160										0,00		
161				0,03						0,03		
162										0,00		
163										0,00		
164										0,00	0,00	0,00
165		0,02	0,14	0,30	0,22	0,03	0,04	0,26	0,00	1,01	0,01	0,01
166										0,00		
167										0,00		
168										0,00		
169			0,03	0,01	0,06		0,02	0,12		0,24		
170			0,00	0,18	0,02	0,01		0,28		0,48	0,00	0,00
171				0,05						0,05		
172			0,00	0,71	0,02		0,00			0,73		
173										0,00	0,01	
174			0,01	0,08	0,03			0,12		0,23		
175			0,00	0,38	0,02					0,40		
176										0,00		
177										0,00	0,00	
178										0,00		
179				0,09		0,03				0,12	0,00	0,00
180				0,11						0,11	0,01	0,00

No	EE 12:0 (µg/g)	EE 14:0 (µg/g)	EE 16:0 (µg/g)	EE 18:2 (µg/g)	EE 18:1 (µg/g)	EE 18:3 (µg/g)	EE 18:0 (µg/g)	EE 20:4 (µg/g)	EE 20:0 (µg/g)	Total FAEE (µg/g)	EtS (µg/g)	EtG (µg/g)
181			0,00			0,02				0,02		
182				0,04	0,01			0,10		0,16		
183			0,00	0,01	0,00		0,00			0,02	0,00	
184			0,00		0,02	0,00		0,19		0,22		0,01
185			0,00	0,12	0,00			0,12		0,25	0,00	0,01
186										0,00		
187										0,00		0,05
188			0,00		0,00	0,00				0,01	0,00	
189										0,00	0,00	
190										0,00		
191			0,00							0,00		
192										0,00		
193										0,00		
194				0,05						0,05		
195										0,00		
196										0,00		
197								0,06		0,06		
198		0,00	0,03	0,23	0,17	0,02	0,00	0,12		0,58	0,01	
199										0,00		
200			0,01	0,12	0,07	0,01	0,00	0,03		0,25		0,02
201										0,00		
202										0,00		
203		0,01	0,05	0,28	0,43	0,05	0,00	0,35		1,16	0,00	0,01
204					0,04					0,04		
205										0,00		
206			0,01		0,02		0,00			0,03		

No	EE 12:0 (µg/g)	EE 14:0 (µg/g)	EE 16:0 (µg/g)	EE 18:2 (µg/g)	EE 18:1 (µg/g)	EE 18:3 (µg/g)	EE 18:0 (µg/g)	EE 20:4 (µg/g)	EE 20:0 (µg/g)	Total FAEE (µg/g)	EtS (µg/g)	EtG (µg/g)
207			0,01	0,34	0,03	0,01		0,01		0,41		0,01
208			0,00	0,27	0,01	0,01		0,11		0,41	0,01	0,01
209										0,00		
210			0,00	0,01	0,02		0,00			0,03	0,00	
211			0,00							0,00	0,05	0,91
212				0,01	0,01			0,24		0,27		
213										0,00		
214			0,00							0,00		
215				0,01						0,01	0,00	
216				0,11	0,00					0,11		
217			0,02		0,05		0,00			0,07		
218								0,08		0,08	0,04	0,92
219			0,22	0,35	0,04					0,62	0,00	0,00
220			0,00	0,32						0,32		0,00
221										0,00		
222										0,00	0,00	
223				0,20						0,20		0,01
224										0,00	0,00	
225										0,00		
226										0,00		0,01
227										0,00		
228										0,00		
229			0,00	0,04	0,01					0,05	0,00	0,01
230				0,11	0,00					0,11		0,01
231				0,01				0,04		0,05		0,00
232	0,00	0,05	0,46	0,55	0,84	0,11	0,06	0,11	0,02	2,21	0,02	9,38

No	EE 12:0 (µg/g)	EE 14:0 (µg/g)	EE 16:0 (µg/g)	EE 18:2 (µg/g)	EE 18:1 (µg/g)	EE 18:3 (µg/g)	EE 18:0 (µg/g)	EE 20:4 (µg/g)	EE 20:0 (µg/g)	Total FAEE (µg/g)	EtS (µg/g)	EtG (µg/g)
233										0,00		
234										0,00		
235					0,00					0,00		0,01
236	0,00		0,06	0,11	0,16	0,02	0,01	0,01		0,38	0,00	
237										0,00	0,01	
238										0,00	0,00	
239		0,00		0,66	0,02	0,01		3,85	0,01	4,55	0,00	0,03
240	0,00		0,03	0,03	0,05	0,02	0,01	0,26		0,40	0,01	0,01
241		0,00	0,02	0,19	0,05	0,01	0,00	1,37		1,64	0,00	
242										0,00	0,00	
243										0,00	0,00	
244										0,00		
245		0,00	0,04	0,13	0,27	0,02	0,00	0,22		0,68	0,00	0,04
246			0,01	0,29	0,03	0,00	0,00			0,33		
247	0,00		0,04	0,21	0,20	0,03	0,02			0,50		
248										0,00		
249										0,00	0,00	
250			0,00	0,15			0,00			0,15		
251										0,00		
252				0,50	0,02	0,01		2,07		2,60	0,03	1,11
253			0,00	0,01	0,02		0,00	0,03		0,06	0,00	
254										0,00		0,00
255			0,00				0,00			0,00	0,00	0,01
256										0,00		
257										0,00		
258			0,00	0,12	0,02					0,14		

No	EE 12:0 (µg/g)	EE 14:0 (µg/g)	EE 16:0 (µg/g)	EE 18:2 (µg/g)	EE 18:1 (µg/g)	EE 18:3 (µg/g)	EE 18:0 (µg/g)	EE 20:4 (µg/g)	EE 20:0 (µg/g)	Total FAEE (µg/g)	EtS (µg/g)	EtG (µg/g)
259			0,00	0,04	0,01		0,00			0,06	0,00	0,01
260										0,00		
261										0,00		
262	0,02	0,02	0,02	0,15	0,18	0,05	0,01			0,44	0,00	0,01
263										0,00		
264				0,03						0,03		
265				0,05						0,05		
266		0,01	0,06	0,32	0,54	0,06	0,00	0,17	0,00	1,17		
267				0,05						0,05		
268										0,00		
269			0,00							0,00	0,01	
270				0,17						0,17	0,00	0,00
271										0,00		
272								1,42		1,42	0,05	6,65
273				0,01						0,01		
274			0,01	0,25	0,05			0,82		1,13		
275				0,10						0,10		0,01
276	0,00	0,00	0,04	0,16	0,19	0,05	0,01	0,14		0,59		
277										0,00		
278										0,00		
279										0,00		
280			0,01	0,62	0,02	0,00		0,78		1,43		
281			0,00	0,06						0,07	0,00	
282			0,00	0,12	0,01	0,00		0,52		0,66		
283			0,00							0,00		
284										0,00		

No	EE 12:0 (µg/g)	EE 14:0 (µg/g)	EE 16:0 (µg/g)	EE 18:2 (µg/g)	EE 18:1 (µg/g)	EE 18:3 (µg/g)	EE 18:0 (µg/g)	EE 20:4 (µg/g)	EE 20:0 (µg/g)	Total FAEE (µg/g)	EtS (µg/g)	EtG (µg/g)
285										0,00		
286			0,00		0,00					0,01	0,01	0,01
287			0,01		0,01					0,02	0,00	
288										0,00		
289										0,00		
290		0,01	0,07	0,06	0,09	0,00	0,01	0,29		0,52	0,01	0,16
291			0,01	0,37	0,01	0,01				0,40		
292			0,00				0,00			0,00		
293										0,00	0,00	
294		0,01	0,26	0,36	0,48	0,08	0,19		0,01	1,39		
295										0,00		
296			0,00	0,21	0,01	0,01		0,19		0,43	0,00	
297			0,00							0,00		0,00
298										0,00		
299			0,00	0,09						0,09		
300		0,00		0,05						0,05		
301				0,05			0,00			0,05	0,00	
302			0,00				0,00			0,00		
303			0,00	0,25	0,00		0,00			0,26		
304							0,00			0,00		
305										0,00		
306										0,00		
307	0,00		0,00	0,24	0,00	0,02	0,00			0,27		0,01
308										0,00		
309			0,01		0,01		0,01	0,03		0,06	0,00	0,03
310			0,00	0,24	0,02	0,02				0,28		0,00

No	EE 12:0 (µg/g)	EE 14:0 (µg/g)	EE 16:0 (µg/g)	EE 18:2 (µg/g)	EE 18:1 (µg/g)	EE 18:3 (µg/g)	EE 18:0 (µg/g)	EE 20:4 (µg/g)	EE 20:0 (µg/g)	Total FAEE (µg/g)	EtS (µg/g)	EtG (µg/g)
311				0,03						0,03		0,01
312			0,00	0,01	0,01					0,02	0,00	
313			0,00							0,00		
314										0,00	0,00	
315			0,01	0,18	0,06	0,02		0,05		0,31		
316			0,00	0,04						0,04	0,00	0,09
317			0,00		0,01					0,01		
318				0,03						0,03		
319				0,03						0,03		
320			0,00	0,22	0,02	0,00	0,00			0,24		
321										0,00		
322										0,00		
323										0,00		
324	0,26	0,00	0,36	0,99	2,36	0,87	0,76		0,00	5,58	0,03	1,77
325										0,00		0,02
326										0,00		
327										0,00		
328										0,00		
329										0,00		
330										0,00		
331										0,00	0,00	0,06
332										0,00		
333										0,00		
334							0,00	0,01		0,01	0,00	
335				0,15	0,01					0,16		
336			0,00	0,21						0,21	0,00	0,00

No	EE 12:0 (µg/g)	EE 14:0 (µg/g)	EE 16:0 (µg/g)	EE 18:2 (µg/g)	EE 18:1 (µg/g)	EE 18:3 (µg/g)	EE 18:0 (µg/g)	EE 20:4 (µg/g)	EE 20:0 (µg/g)	Total FAEE (µg/g)	EtS (µg/g)	EtG (µg/g)
337										0,00	0,08	1,12
338										0,00	0,00	0,01
339			0,00				0,00	0,08		0,08	0,00	0,00
340										0,00		
341										0,00		
342			0,00				0,00			0,00		0,03
343				0,01						0,01		
344			0,00				0,00	0,01		0,01		
345	0,06	0,00		0,15	0,22	0,13		0,09		0,65	0,00	0,01
346										0,00		
347				0,01						0,01		0,00
348								0,01		0,01	0,01	0,02
349			0,01		0,02		0,00			0,03	0,01	0,01
350			0,00		0,00	0,01			0,02	0,04	0,01	0,09
351					0,01			0,01		0,02		
352			0,00	0,39		0,00				0,40		0,06
353										0,00		
354										0,00		0,00
355										0,00	0,01	
356				0,01						0,01		
357										0,00	0,00	0,01
358			0,00		0,03		0,00	0,01		0,05		0,01
359										0,00		
360			0,00	0,22	0,00	0,02				0,25	0,01	0,02
361			0,01	0,26	0,02	0,04		0,05		0,38	0,00	
362										0,00		

No	EE 12:0 (µg/g)	EE 14:0 (µg/g)	EE 16:0 (µg/g)	EE 18:2 (µg/g)	EE 18:1 (µg/g)	EE 18:3 (µg/g)	EE 18:0 (µg/g)	EE 20:4 (µg/g)	EE 20:0 (µg/g)	Total FAEE (µg/g)	EtS (µg/g)	EtG (µg/g)
363				0,01						0,01	0,00	
364										0,00	0,05	0,54
365										0,00	0,00	
366				0,19						0,19		
367				0,01						0,01		
368			0,00					0,01		0,01		0,00
369	0,02	0,04	0,35	4,84	5,20	0,83	0,07	1,34	0,01	12,68	0,00	0,03
370										0,00	0,00	
371	0,01		0,00	0,04	0,05	0,01		0,01		0,12	0,02	0,03
372										0,00		
373	0,01	0,01	0,01	0,28	0,35	0,13	0,00			0,80		
374										0,00	0,00	0,01
375										0,00		
376		0,01	0,17	0,23	0,34	0,06	0,13		0,00	0,93	0,00	
377			0,02	0,18	0,04	0,02	0,00	0,01		0,26	0,00	
378										0,00		0,00
379										0,00	0,00	
380										0,00		0,02
381										0,00	0,04	0,53
382			0,02	0,05		0,00				0,07	0,01	0,02
383										0,00	0,00	
384										0,00	0,00	
385			0,00	1,08	0,01	0,04		6,50	0,00	7,63		0,02
386			0,02	0,27	0,01	0,02	0,02		0,00	0,34	0,03	18,32
387				0,01						0,01		
388										0,00	0,00	

No	EE 12:0 (µg/g)	EE 14:0 (µg/g)	EE 16:0 (µg/g)	EE 18:2 (µg/g)	EE 18:1 (µg/g)	EE 18:3 (µg/g)	EE 18:0 (µg/g)	EE 20:4 (µg/g)	EE 20:0 (µg/g)	Total FAEE (µg/g)	EtS (µg/g)	EtG (µg/g)
389										0,00		0,01
390				0,04						0,04		0,01
391				0,11	0,01					0,12	0,01	0,00
392			0,00					0,01		0,01	0,01	0,00
393							0,02			0,02		
394										0,00	0,00	
395										0,00		
396			0,01	0,46	0,03	0,02	0,00	0,10		0,62	0,06	0,10
397			0,00		0,01					0,01		
398			0,08		0,06	0,01	0,02	0,04		0,21		0,03
399										0,00		
400										0,00		
401			0,01		0,03	0,00	0,00	0,03		0,07		0,01
402			0,00	0,20		0,01		0,76		0,98		0,01
403										0,00		
404										0,00		
405										0,00		
406										0,00	0,00	
407				0,01						0,01		
408										0,00		
409				0,14				0,10		0,25	0,00	
410			0,02		0,04		0,09	0,03	0,01	0,19	0,01	0,22
411										0,00		
412										0,00		
413				0,25		0,01				0,26	0,01	0,01
414				0,14						0,14		

No	EE 12:0 (µg/g)	EE 14:0 (µg/g)	EE 16:0 (µg/g)	EE 18:2 (µg/g)	EE 18:1 (µg/g)	EE 18:3 (µg/g)	EE 18:0 (µg/g)	EE 20:4 (µg/g)	EE 20:0 (µg/g)	Total FAEE (µg/g)	EtS (µg/g)	EtG (µg/g)
415			0,00					0,05		0,05		
416										0,00	0,00	
417										0,00		
418			0,00							0,00		
419										0,00	0,00	
420										0,00		
421	0,06	0,14	0,30	0,59	3,01	0,72	0,45	0,42	0,02	5,72	0,01	0,22
422		0,00	0,00	0,01	0,00					0,02		0,00
423				0,03						0,03		0,01
424										0,00	0,00	
425										0,00	0,00	
426		0,00	0,00							0,00	0,00	
427										0,00		
428										0,00		0,01
429			0,01		0,02			0,01		0,04		0,02
430			0,02	0,20	0,17	0,03				0,42		0,01
431										0,00		
432										0,00		
433			0,00	0,01				0,09		0,10		
434			0,00	0,18	0,00					0,18	0,00	
435			0,00	0,12	0,02	0,00		0,01		0,16	0,01	
436										0,00		0,02
437				0,06				0,01		0,07		
438			0,00							0,00		
439	0,07	0,02	0,03	0,89	0,36	0,11	0,01	0,15		1,63	0,01	0,03
440								0,04		0,04		0,03

No	EE 12:0 (µg/g)	EE 14:0 (µg/g)	EE 16:0 (µg/g)	EE 18:2 (µg/g)	EE 18:1 (µg/g)	EE 18:3 (µg/g)	EE 18:0 (µg/g)	EE 20:4 (µg/g)	EE 20:0 (µg/g)	Total FAEE (µg/g)	EtS (µg/g)	EtG (µg/g)
441										0,00		
442		0,00	0,00	0,06	0,00			0,04	0,00	0,11		0,01
443			0,00	0,17	0,02	0,00				0,19		
444		0,00	0,03	0,09	0,06	0,00	0,01	0,08		0,27		0,02
445			0,01	0,01	0,00			0,01		0,03	0,01	0,01
446										0,00		
447			0,00							0,00		
448		0,00	0,00	0,03	0,00			0,19		0,23		0,01
449			0,00					0,01		0,01	0,01	0,01
450										0,00		0,01
451				0,10				0,04		0,14		0,00
452				0,77				0,69		1,45	0,01	0,01
453										0,00	0,00	
454	0,65	0,73	2,01		2,00	4,36	0,26		0,09	10,09		0,03
455				0,14						0,14		0,00
456	0,01	0,07	0,90	0,54	0,16		0,09	0,84		2,61		0,01
457				0,03						0,03		
458										0,00		
459			0,01	0,05	0,01					0,07	0,00	0,00
460		0,01	0,14	0,09	0,02		0,02	0,15		0,43	0,01	0,05
461										0,00		
462										0,00	0,00	
463										0,00	0,00	
464								0,03		0,03	0,00	
465										0,00	0,00	
466		0,11		0,40		0,03			0,01	0,54	0,01	0,02

No	EE 12:0 (µg/g)	EE 14:0 (µg/g)	EE 16:0 (µg/g)	EE 18:2 (µg/g)	EE 18:1 (µg/g)	EE 18:3 (µg/g)	EE 18:0 (µg/g)	EE 20:4 (µg/g)	EE 20:0 (µg/g)	Total FAEE (µg/g)	EtS (µg/g)	EtG (µg/g)
467										0,00		
468	0,01	0,05	0,29	0,97	1,46	0,11	0,02	0,20	0,00	3,12	0,05	0,97
469			0,00							0,00		0,01
470			0,00				0,00			0,00	0,00	
471										0,00		
472				0,06						0,06		
473		0,01	0,03	0,34	0,02	0,02	0,01	0,35		0,77	0,00	0,00
474										0,00		
475										0,00		
476				0,11	0,01					0,12	0,01	0,00
477										0,00		
478				0,04	0,01					0,05	0,01	
