# Supplementary material for: Estimation of Prenatal Alcohol Exposure: Comparison of Retrospective Survey and Measurement of Fatty Acid Ethyl Esters, Ethyl Sulfate, and Ethyl Glucuronide Concentrations in Neonatal Meconium
Source: Toxics. 2026 Feb 4;14(2):155. doi: 10.3390/toxics14020155 (PMC12944540; doi:10.3390/toxics14020155)
Supplement: Supplementary file 1 [file toxics-14-00155-s001.zip › Table S10 chemical analysis results in mole concentration.pdf]

Table S10. Results of GC-MS-SIM and LC-ESI-MS-MS chemical analyses (in mole concentration units - nmol/g) for infant meconium (n=478) collected on the first day after birth at the Neonatology Clinic of the Medical University of Gdańsk in the Pomeranian Province between June 16, 2019, and April 24, 2020.

No	EE 12:0 (nmol/g)	EE 14:0 (nmol/g)	EE 16:0 (nmol/g)	EE 18:2 (nmol/g)	EE 18:1 (nmol/g)	EE 18:3 (nmol/g)	EE 18:0 (nmol/g)	EE 20:4 (nmol/g)	EE 20:0 (nmol/g)	Total FAEE (nmol/g)	EtS (nmol/g)	EtG (nmol/g)
1	2,38	4,37	2,99	7,83	18,92	0,79	1,57		0,20	39,05	115,00	103,10
2								0,21		0,21		0,16
3			0,04				0,01			0,05		0,03
4			0,01	1,80			0,01			1,82	0,31	3,09
5										0,00	0,02	
6										0,00	0,03	0,04
7										0,00	0,11	0,01
8										0,00		
9										0,00		0,04
10										0,00		
11			0,03	0,40						0,44		0,01
12										0,00		
13										0,00		
14										0,00		
15			0,05	0,14	0,23		0,01	0,24		0,67		0,01
16										0,00		
17										0,00		
18			0,01	0,69				1,03		1,73		0,16
19										0,00		
20				0,20						0,20	0,03	
21				0,04						0,04	0,04	
22				2,01		0,12				2,13		0,01
23			0,20	0,04						0,23		
24										0,00		0,03

No	EE 12:0 (nmol/g)	EE 14:0 (nmol/g)	EE 16:0 (nmol/g)	EE 18:2 (nmol/g)	EE 18:1 (nmol/g)	EE 18:3 (nmol/g)	EE 18:0 (nmol/g)	EE 20:4 (nmol/g)	EE 20:0 (nmol/g)	Total FAEE (nmol/g)	EtS (nmol/g)	EtG (nmol/g)
25	0,05		0,08	0,71	0,04	0,10	0,01			0,98		0,01
26				1,64						1,64	0,01	0,01
27										0,00	0,02	
28	2,85	2,83	7,05		6,45	14,24	0,83		0,26	34,50		0,16
29				0,45						0,45		0,01
30	0,04	0,29	3,16	1,74	0,52		0,30	2,52		8,58		0,06
31				0,08						0,08		
32										0,00		
33			0,04	0,16	0,04					0,24	0,01	0,01
34		0,02	0,49	0,29	0,07		0,06	0,46		1,39	0,07	0,24
35										0,00		
36										0,00		
37										0,00		
38								0,10		0,10		
39										0,00	0,02	
40		0,44		1,30		0,09			0,02	1,84	0,06	0,08
41										0,00		
42	0,02	0,20	1,03	3,14	4,71	0,37	0,06	0,61	0,01	10,16	0,36	4,35
43			0,01							0,01		0,06
44			0,01				0,01			0,01		
45										0,00		
46				0,18						0,18		
47		0,02	0,12	1,09	0,07	0,05	0,02	1,05		2,41	0,01	0,02
48										0,00		
49										0,00		
50				0,35	0,02					0,38	0,08	0,02

No	EE 12:0 (nmol/g)	EE 14:0 (nmol/g)	EE 16:0 (nmol/g)	EE 18:2 (nmol/g)	EE 18:1 (nmol/g)	EE 18:3 (nmol/g)	EE 18:0 (nmol/g)	EE 20:4 (nmol/g)	EE 20:0 (nmol/g)	Total FAEE (nmol/g)	EtS (nmol/g)	EtG (nmol/g)
51										0,00		
52				0,14	0,03					0,18	0,05	
53										0,00		
54				0,10				0,09		0,20		0,09
55	2,98	2,02	5,79		28,09	7,60	0,72		0,09	47,30	0,05	0,29
56										0,00		
57			0,04	0,30	0,04			0,17		0,56		
58				0,93				0,34		1,27		0,01
59										0,00		
60				0,95	0,01					0,96		
61			0,04	0,68			0,01	0,22		0,95		
62										0,00	0,16	0,05
63										0,00		0,01
64										0,00		
65				0,40		0,02				0,42	0,25	0,62
66				0,67						0,67		
67										0,00	0,03	2,90
68										0,00		
69										0,00	0,03	
70	0,05		0,07		0,01	0,08		0,66		0,88	0,02	
71										0,00		
72				1,72	0,13	0,04		0,30		2,19	0,01	0,01
73										0,00	0,01	
74										0,00	0,01	
75										0,00	0,02	
76										0,00		

No	EE 12:0 (nmol/g)	EE 14:0 (nmol/g)	EE 16:0 (nmol/g)	EE 18:2 (nmol/g)	EE 18:1 (nmol/g)	EE 18:3 (nmol/g)	EE 18:0 (nmol/g)	EE 20:4 (nmol/g)	EE 20:0 (nmol/g)	Total FAEE (nmol/g)	EtS (nmol/g)	EtG (nmol/g)
77				2,27	0,08			1,20		3,55		0,04
78				0,16						0,16		
79				0,11	0,01			0,08		0,20	0,15	0,05
80				0,83						0,83		
81				1,44				2,09		3,53	0,02	0,06
82										0,00		
83		0,05	0,20	1,76	0,15	0,01		1,41	0,06	3,65		0,07
84										0,00		0,02
85										0,00	0,06	0,04
86	1,98	1,73	2,46	7,40	2,24	1,22	0,11	5,58		22,71		0,03
87				0,89	0,01	0,02		0,25	0,01	1,19		0,01
88				0,25	0,03	0,01				0,29		
89										0,00		
90	0,35	0,21	0,24	6,78	0,67	0,94	0,05	1,25		10,49		
91				0,74		0,02				0,77		
92			0,25	4,31	2,81	0,55	0,03	0,86		8,82	0,06	0,06
93										0,00		
94			0,02		0,03		0,11		0,01	0,17	0,70	20,81
95					0,02					0,02	0,03	
96										0,00		
97				0,04						0,04		
98										0,00		
99										0,00	0,01	
100			0,03	0,09	0,24	0,04	0,01	0,10		0,51	0,02	0,04
101			0,04	2,16	0,10	0,07		0,03		2,41		0,05
102			0,02	0,29	0,10	0,07				0,47	0,03	

No	EE 12:0 (nmol/g)	EE 14:0 (nmol/g)	EE 16:0 (nmol/g)	EE 18:2 (nmol/g)	EE 18:1 (nmol/g)	EE 18:3 (nmol/g)	EE 18:0 (nmol/g)	EE 20:4 (nmol/g)	EE 20:0 (nmol/g)	Total FAEE (nmol/g)	EtS (nmol/g)	EtG (nmol/g)
103								0,16		0,16		0,05
104			0,17	0,76	1,32	0,11			0,01	2,36		0,01
105										0,00		
106										0,00		
107			0,01							0,01	0,02	0,03
108										0,00		
109			0,10	2,03	0,57	0,15		6,24		9,08	0,01	
110								0,52		0,52	0,04	
111				1,41	0,03			1,15		2,59		
112										0,00		
113				0,04						0,04		0,01
114				0,13				0,11		0,24		
115			0,01				0,01			0,01	0,09	0,01
116				0,68		0,04				0,71		0,07
117			0,09	0,75	0,30	0,07				1,21	0,01	
118			0,04	0,18	0,70		0,02	0,93		1,86		0,40
119			0,02	1,00	0,20			0,41		1,63	0,04	0,04
120			0,02	1,73	0,08			0,27		2,11	0,01	0,01
121										0,00		
122		0,05	0,37	1,28	1,84	0,20	0,03	0,74		4,52		0,02
123			0,01		0,03					0,03	0,03	0,03
124				0,11				0,51		0,61		
125										0,00		0,01
126										0,00		
127										0,00		0,05
128										0,00		

No	EE 12:0 (nmol/g)	EE 14:0 (nmol/g)	EE 16:0 (nmol/g)	EE 18:2 (nmol/g)	EE 18:1 (nmol/g)	EE 18:3 (nmol/g)	EE 18:0 (nmol/g)	EE 20:4 (nmol/g)	EE 20:0 (nmol/g)	Total FAEE (nmol/g)	EtS (nmol/g)	EtG (nmol/g)
129			0,19	0,96	0,77		0,01	0,72		2,65	0,05	0,03
130										0,00		
131					0,04	0,10		0,49		0,63		
132								0,77		0,77		
133										0,00	0,01	
134										0,00		
135										0,00		
136			0,12	0,24	0,34		0,03	0,45		1,19		0,01
137			0,02	0,77		0,03	0,01			0,82	0,03	
138	0,09	0,21	1,88	7,24	6,89	0,84	0,47	2,50	0,16	20,28	0,01	0,08
139		0,06	0,44	1,14	2,82	0,25	0,03	1,29	0,01	6,05		
140				0,04	0,02		0,01	0,42		0,49		0,03
141										0,00		
142										0,00		
143				0,21				2,58		2,79	0,02	
144										0,00		
145			0,02	0,11	0,07			0,12		0,33		
146			0,02	0,26	0,06	0,05	0,01			0,39		0,02
147				0,39	0,06					0,46		
148				0,04						0,04		0,01
149			0,03		0,04					0,07	0,04	2,44
150										0,00		
151										0,00		
152	0,01		0,02	0,04	0,07			0,26		0,39		0,07
153										0,00		
154								0,08		0,08	0,02	0,01

No	EE 12:0 (nmol/g)	EE 14:0 (nmol/g)	EE 16:0 (nmol/g)	EE 18:2 (nmol/g)	EE 18:1 (nmol/g)	EE 18:3 (nmol/g)	EE 18:0 (nmol/g)	EE 20:4 (nmol/g)	EE 20:0 (nmol/g)	Total FAEE (nmol/g)	EtS (nmol/g)	EtG (nmol/g)
155				0,19						0,19	0,02	
156			0,01		0,01					0,02		0,08
157										0,00		
158				0,48		0,04		0,56		1,08	0,03	0,09
159			0,02				0,01			0,03		
160	0,01		0,03	0,63	0,18	0,06		0,37		1,28		0,01
161										0,00	0,04	
162										0,00		0,05
163										0,00		
164								0,67		0,67	0,06	0,09
165										0,00		
166			0,08	0,66	0,23	0,04	0,01	0,64		1,66	0,14	0,29
167		0,09	0,93	1,10	2,25	0,23	0,14	0,90	0,01	5,64		0,31
168			0,01							0,01	0,02	
169			0,01		0,07			0,14		0,22		
170			0,01	0,09	0,03					0,13	0,05	0,04
171										0,00		
172										0,00	0,22	0,01
173										0,00		
174				0,12						0,12	0,03	
175								0,76		0,76	0,01	
176										0,00	0,01	
177										0,00		
178										0,00	0,01	
179					0,02				0,01	0,03		0,01
180	0,07		0,02	1,86	0,11	0,16		0,85		3,06	0,01	0,01

No	EE 12:0 (nmol/g)	EE 14:0 (nmol/g)	EE 16:0 (nmol/g)	EE 18:2 (nmol/g)	EE 18:1 (nmol/g)	EE 18:3 (nmol/g)	EE 18:0 (nmol/g)	EE 20:4 (nmol/g)	EE 20:0 (nmol/g)	Total FAEE (nmol/g)	EtS (nmol/g)	EtG (nmol/g)
181				0,17						0,17		
182										0,00		
183			0,01							0,01		0,06
184										0,00	0,01	
185				0,04						0,04		
186										0,00		
187				0,11						0,11		
188										0,00		
189										0,00		
190										0,00	0,01	0,02
191		0,09	0,51	0,98	0,70	0,08	0,11	0,77	0,01	3,25	0,05	0,03
192										0,00		
193										0,00		
194										0,00		
195			0,11	0,04	0,19		0,05	0,37		0,76		
196			0,01	0,57	0,07	0,03		0,83		1,50	0,03	0,02
197				0,17						0,17		
198			0,01	2,29	0,07		0,01			2,37		
199										0,00	0,05	
200			0,03	0,26	0,08			0,36		0,73		
201			0,01	1,24	0,06					1,31		
202										0,00		
203										0,00		
204										0,00		
205				0,30		0,09				0,39		0,02
206				0,35						0,35	0,05	0,01

No	EE 12:0 (nmol/g)	EE 14:0 (nmol/g)	EE 16:0 (nmol/g)	EE 18:2 (nmol/g)	EE 18:1 (nmol/g)	EE 18:3 (nmol/g)	EE 18:0 (nmol/g)	EE 20:4 (nmol/g)	EE 20:0 (nmol/g)	Total FAEE (nmol/g)	EtS (nmol/g)	EtG (nmol/g)
207			0,01			0,07				0,08		
208				0,13	0,04			0,32		0,49		
209			0,01	0,04	0,01		0,01			0,06	0,01	
210			0,01		0,06	0,01		0,58		0,66		0,04
211			0,01	0,38	0,01			0,37		0,77	0,01	0,03
212										0,00		
213										0,00		0,23
214			0,01		0,01	0,01				0,03	0,04	
215										0,00		
216										0,00		
217			0,01							0,01		
218										0,00		
219										0,00		
220				0,15						0,15		
221										0,00		
222										0,00		
223								0,17		0,17		
224			0,12	0,74	0,55	0,07	0,01	0,37		1,85	0,07	
225										0,00		
226			0,05	0,38	0,23	0,03	0,01	0,10		0,80		0,07
227										0,00		
228										0,00		
229		0,03	0,16	0,91	1,38	0,17	0,01	1,05		3,70	0,03	0,03
230					0,14					0,14		
231										0,00		
232			0,03		0,06		0,01			0,10		

No	EE 12:0 (nmol/g)	EE 14:0 (nmol/g)	EE 16:0 (nmol/g)	EE 18:2 (nmol/g)	EE 18:1 (nmol/g)	EE 18:3 (nmol/g)	EE 18:0 (nmol/g)	EE 20:4 (nmol/g)	EE 20:0 (nmol/g)	Total FAEE (nmol/g)	EtS (nmol/g)	EtG (nmol/g)
233			0,03	1,11	0,10	0,04		0,03		1,32		0,05
234			0,01	0,89	0,05	0,04		0,32		1,30	0,05	0,06
235										0,00		
236			0,01	0,04	0,05		0,01			0,10		
237			0,01							0,01	0,42	4,10
238				0,04	0,04			0,73		0,81		
239										0,00		
240			0,01							0,01		
241				0,04						0,04	0,02	
242				0,35	0,01					0,36		
243			0,07		0,16		0,01			0,24		
244								0,25		0,25	0,31	4,13
245			0,79	1,14	0,13					2,06	0,04	0,01
246			0,01	1,04						1,04		0,01
247										0,00		
248										0,00		
249				0,66						0,66		0,03
250										0,00	0,01	
251										0,00		
252										0,00		0,03
253										0,00		
254										0,00		
255			0,01	0,12	0,03					0,16	0,03	0,04
256				0,35	0,01					0,36		0,02
257				0,04				0,12		0,16		0,02
258	0,01	0,19	1,63	1,79	2,72	0,36	0,20	0,34	0,05	7,30	0,13	42,23

No	EE 12:0 (nmol/g)	EE 14:0 (nmol/g)	EE 16:0 (nmol/g)	EE 18:2 (nmol/g)	EE 18:1 (nmol/g)	EE 18:3 (nmol/g)	EE 18:0 (nmol/g)	EE 20:4 (nmol/g)	EE 20:0 (nmol/g)	Total FAEE (nmol/g)	EtS (nmol/g)	EtG (nmol/g)
259										0,00		
260										0,00		
261					0,01					0,01		0,03
262	0,01		0,22	0,35	0,53	0,07	0,03	0,03		1,25	0,02	
263										0,00	0,10	
264										0,00	0,03	
265				2,13	0,05	0,04		11,59	0,04	13,85	0,01	0,11
266	0,01		0,11	0,08	0,17	0,07	0,02	0,77		1,23	0,06	0,06
267			0,06	0,62	0,15	0,04	0,01	4,11		4,99	0,01	
268										0,00	0,01	
269										0,00	0,02	
270										0,00		
271			0,13	0,41	0,87	0,07	0,01	0,68		2,17	0,03	0,19
272			0,02	0,93	0,10	0,01	0,01			1,06		
273	0,01		0,14	0,68	0,64	0,08	0,07			1,63		
274										0,00		
275										0,00		
276			0,01	0,48			0,01			0,49		
277										0,00		
278				1,61	0,06	0,05		6,23		7,96	0,22	5,00
279			0,01	0,04	0,06		0,01	0,08		0,19	0,01	
280										0,00		0,01
281			0,01				0,01			0,01		0,03
282										0,00		
283										0,00		
284			0,01	0,39	0,05					0,45		

No	EE 12:0 (nmol/g)	EE 14:0 (nmol/g)	EE 16:0 (nmol/g)	EE 18:2 (nmol/g)	EE 18:1 (nmol/g)	EE 18:3 (nmol/g)	EE 18:0 (nmol/g)	EE 20:4 (nmol/g)	EE 20:0 (nmol/g)	Total FAEE (nmol/g)	EtS (nmol/g)	EtG (nmol/g)
285			0,01	0,14	0,04		0,01			0,19	0,03	0,03
286										0,00		
287										0,00		
288	0,09	0,07	0,09	0,48	0,57	0,16	0,02			1,46	0,04	0,03
289										0,00		
290				0,11						0,11		
291				0,17						0,17		
292		0,04	0,23	1,03	1,75	0,19	0,01	0,50	0,01	3,76		
293				0,16						0,16		
294										0,00		
295			0,01							0,01	0,05	
296				0,54						0,54		0,01
297										0,00		
298								4,28		4,28	0,41	29,95
299				0,04						0,04		
300			0,03	0,81	0,15			2,48		3,47		
301				0,31						0,31		0,03
302	0,01		0,14	0,53	0,62	0,18	0,02	0,41		1,90		
303										0,00		
304										0,00		
305										0,00		
306			0,02	2,03	0,08	0,01		2,34		4,47		
307			0,01	0,21						0,22	0,03	
308			0,01	0,39	0,04	0,01		1,58		2,03		
309			0,01							0,01		
310										0,00		

No	EE 12:0 (nmol/g)	EE 14:0 (nmol/g)	EE 16:0 (nmol/g)	EE 18:2 (nmol/g)	EE 18:1 (nmol/g)	EE 18:3 (nmol/g)	EE 18:0 (nmol/g)	EE 20:4 (nmol/g)	EE 20:0 (nmol/g)	Total FAEE (nmol/g)	EtS (nmol/g)	EtG (nmol/g)
311										0,00		
312			0,01		0,01					0,02	0,07	0,07
313			0,02		0,04					0,06		
314										0,00		
315										0,00		
316		0,03	0,24	0,18	0,29	0,01	0,03	0,87		1,64	0,07	0,74
317			0,02	1,21	0,03	0,03				1,29		
318			0,01				0,01			0,01		
319										0,00	0,01	
320		0,04	0,90	1,17	1,55	0,28	0,60		0,03	4,56		
321										0,00		
322			0,01	0,68	0,04	0,03		0,58		1,34	0,02	
323			0,01							0,01		0,01
324										0,00		
325			0,01	0,29						0,30		
326				0,17						0,17		
327				0,16			0,01			0,16	0,02	
328			0,01				0,01			0,01		
329			0,01	0,81	0,01		0,01			0,83		
330							0,01			0,01		
331										0,00		
332										0,00		
333	0,01		0,01	0,79	0,01	0,05	0,01			0,87		0,05
334										0,00		
335			0,04		0,04		0,02	0,09		0,18		0,12
336			0,01	0,77	0,07	0,06				0,91		0,01

No	EE 12:0 (nmol/g)	EE 14:0 (nmol/g)	EE 16:0 (nmol/g)	EE 18:2 (nmol/g)	EE 18:1 (nmol/g)	EE 18:3 (nmol/g)	EE 18:0 (nmol/g)	EE 20:4 (nmol/g)	EE 20:0 (nmol/g)	Total FAEE (nmol/g)	EtS (nmol/g)	EtG (nmol/g)
337				0,11						0,11		0,02
338			0,01	0,04	0,04					0,08		
339			0,01							0,01		
340										0,00	0,01	
341			0,02	0,58	0,18	0,05		0,14		0,98		
342			0,01	0,12						0,12	0,01	0,40
343			0,01		0,04					0,05		
344				0,10						0,10		
345				0,11						0,11		
346			0,01	0,72	0,05	0,01	0,01			0,79		
347										0,00		
348										0,00		
349										0,00		
350	1,12		1,25	3,20	7,61	2,83	2,42		0,01	18,43	0,26	7,97
351										0,00		0,11
352										0,00		
353										0,00		
354										0,00		
355										0,00		
356										0,00		
357										0,00		0,29
358										0,00		
359										0,00		
360							0,01	0,03		0,04	0,01	
361				0,48	0,04					0,52		
362			0,01	0,68						0,68	0,03	0,02

No	EE 12:0 (nmol/g)	EE 14:0 (nmol/g)	EE 16:0 (nmol/g)	EE 18:2 (nmol/g)	EE 18:1 (nmol/g)	EE 18:3 (nmol/g)	EE 18:0 (nmol/g)	EE 20:4 (nmol/g)	EE 20:0 (nmol/g)	Total FAEE (nmol/g)	EtS (nmol/g)	EtG (nmol/g)
363										0,00	0,65	5,06
364										0,00		0,05
365			0,01				0,01	0,23		0,24	0,02	0,01
366										0,00		
367										0,00		
368			0,01				0,01			0,01		0,15
369				0,04						0,04		
370			0,01				0,01	0,03		0,05		
371	0,27			0,49	0,71	0,43		0,26		2,15	0,01	0,05
372										0,00		
373				0,04						0,04		0,02
374								0,03		0,03	0,10	0,08
375			0,04		0,05		0,01			0,10	0,06	0,03
376			0,01		0,01	0,04			0,07	0,13	0,08	0,41
377					0,04			0,03		0,08		
378			0,01	1,28		0,01				1,30		0,26
379										0,00		
380										0,00		0,01
381										0,00	0,07	
382				0,04						0,04		
383										0,00	0,03	0,06
384			0,01		0,10		0,01	0,03		0,15		0,06
385										0,00		
386			0,01	0,72	0,01	0,07				0,81	0,07	0,07
387			0,02	0,84	0,07	0,13		0,16		1,23	0,01	
388										0,00		

No	EE 12:0 (nmol/g)	EE 14:0 (nmol/g)	EE 16:0 (nmol/g)	EE 18:2 (nmol/g)	EE 18:1 (nmol/g)	EE 18:3 (nmol/g)	EE 18:0 (nmol/g)	EE 20:4 (nmol/g)	EE 20:0 (nmol/g)	Total FAEE (nmol/g)	EtS (nmol/g)	EtG (nmol/g)
389				0,04						0,04		
390										0,00	0,42	2,45
391										0,00	0,02	
392				0,62						0,62		
393				0,04						0,04		
394			0,01					0,03		0,04		0,01
395	0,08	0,16	1,23	15,69	16,76	2,69	0,21	4,03	0,02	40,88	0,02	0,15
396										0,00	0,01	
397	0,05		0,01	0,11	0,16	0,04		0,03		0,40	0,14	0,12
398										0,00		
399	0,06	0,03	0,04	0,92	1,14	0,43	0,01			2,63		
400										0,00	0,04	0,02
401										0,00		
402		0,02	0,59	0,74	1,08	0,19	0,41		0,01	3,04	0,01	
403			0,06	0,57	0,12	0,05	0,01	0,03		0,85	0,03	
404										0,00		0,01
405										0,00	0,02	
406										0,00		0,09
407										0,00	0,33	2,39
408			0,06	0,15		0,01				0,22	0,06	0,07
409										0,00		
410										0,00	0,01	
411			0,01	3,50	0,04	0,12		19,56	0,01	23,24		0,09
412			0,08	0,88	0,02	0,05	0,05		0,01	1,11	0,22	82,47
413				0,04						0,04		
414										0,00	0,01	

No	EE 12:0 (nmol/g)	EE 14:0 (nmol/g)	EE 16:0 (nmol/g)	EE 18:2 (nmol/g)	EE 18:1 (nmol/g)	EE 18:3 (nmol/g)	EE 18:0 (nmol/g)	EE 20:4 (nmol/g)	EE 20:0 (nmol/g)	Total FAEE (nmol/g)	EtS (nmol/g)	EtG (nmol/g)
415										0,00		0,03
416				0,12						0,12		0,06
417				0,36	0,04					0,40	0,04	0,01
418			0,01					0,03		0,04	0,06	0,01
419							0,05			0,05		
420										0,00	0,02	
421										0,00		
422			0,03	1,50	0,09	0,06	0,01	0,29		1,98	0,44	0,44
423			0,01		0,02					0,02		
424			0,29		0,19	0,03	0,05	0,12		0,69		0,13
425										0,00		
426										0,00		
427			0,04		0,10	0,01	0,01	0,08		0,23		0,05
428			0,01	0,66		0,04		2,29		2,99		0,02
429										0,00		
430										0,00		
431										0,00		
432										0,00	0,02	
433				0,04						0,04		
434										0,00		
435				0,47				0,31		0,78	0,01	
436			0,08		0,12		0,29	0,10	0,02	0,62	0,06	1,00
437										0,00		
438										0,00		
439				0,81		0,04				0,85	0,06	0,06
440				0,44						0,44		

No	EE 12:0 (nmol/g)	EE 14:0 (nmol/g)	EE 16:0 (nmol/g)	EE 18:2 (nmol/g)	EE 18:1 (nmol/g)	EE 18:3 (nmol/g)	EE 18:0 (nmol/g)	EE 20:4 (nmol/g)	EE 20:0 (nmol/g)	Total FAEE (nmol/g)	EtS (nmol/g)	EtG (nmol/g)
441			0,01					0,15		0,15		
442										0,00	0,02	
443										0,00		
444			0,01							0,01		
445										0,00	0,01	
446										0,00		
447	0,27	0,56	1,06	1,92	9,71	2,34	1,45	1,26	0,05	18,62	0,05	0,99
448			0,01	0,04	0,01					0,05		0,02
449				0,11						0,11		0,06
450										0,00	0,01	
451										0,00		
452			0,01							0,01		
453										0,00		
454										0,00		0,06
455			0,03		0,07			0,03		0,14		0,07
456			0,07	0,65	0,56	0,10				1,37		0,06
457										0,00		
458										0,00		
459			0,01	0,04				0,26		0,30		
460			0,01	0,58	0,01					0,59	0,03	
461			0,01	0,39	0,06	0,01		0,03		0,50	0,08	
462										0,00		0,10
463				0,20				0,03		0,24		
464			0,01							0,01		
465	0,32	0,07	0,11	2,88	1,15	0,37	0,02	0,46		5,36	0,06	0,15
466								0,13		0,13		0,13

No	EE 12:0 (nmol/g)	EE 14:0 (nmol/g)	EE 16:0 (nmol/g)	EE 18:2 (nmol/g)	EE 18:1 (nmol/g)	EE 18:3 (nmol/g)	EE 18:0 (nmol/g)	EE 20:4 (nmol/g)	EE 20:0 (nmol/g)	Total FAEE (nmol/g)	EtS (nmol/g)	EtG (nmol/g)
467										0,00		
468			0,01	0,18	0,01			0,13	0,01	0,34		0,05
469			0,01	0,54	0,05	0,01				0,61		
470			0,12	0,30	0,18	0,01	0,03	0,23		0,87		0,08
471			0,02	0,04	0,01			0,03		0,10	0,05	0,05
472										0,00		
473			0,01							0,01		
474			0,01	0,10	0,01			0,57		0,69		0,03
475			0,01					0,03		0,04	0,04	0,03
476										0,00		0,03
477				0,33				0,11		0,44		0,02
478				2,49				2,07		4,55	0,08	0,03
