# Supplementary material for: Estimation of Prenatal Alcohol Exposure: Comparison of Retrospective Survey and Measurement of Fatty Acid Ethyl Esters, Ethyl Sulfate, and Ethyl Glucuronide Concentrations in Neonatal Meconium
Source: Toxics. 2026 Feb 4;14(2):155. doi: 10.3390/toxics14020155 (PMC12944540; doi:10.3390/toxics14020155)
Supplement: Supplementary file 1 [file toxics-14-00155-s001.zip › Table S11 results - questionnaire.pdf]

Table S11. Results of survey questionnaire (478 surveys were compiled, but a variable number of people responded to individual questions)

Question	Number of answers (%)	Comments
**1. Which pregnancy does the current baby come from?**<br>a) 1st pregnancy<br>b) 2nd pregnancy<br>c) 3rd pregnancy<br>d) 4th pregnancy<br>e) 5th or more pregnancy	a) 199 (43.4)<br>b) 142 (31.0)<br>c) 84 (18.3)<br>d) 33 (7.2)<br>e) 16 (3.5)	Total answers - 474.
**2. How many times have you given birth so far (except giving birth now)?**<br>a) 0<br>b) 1<br>c) 2<br>d) 3<br>e) 4 or more times	a) 231 (48.6)<br>b) 161 (33.9)<br>c) 54 (11.4)<br>d) 20 (4.2)<br>e) 9 (1.9)	Total answers - 475.
**3. In which week of pregnancy was the current baby born?**<br>a) At term, more than 37 weeks of gestation .<br>b) Preterm, less than 37 weeks of gestation.<br>c) 42 weeks of gestation or more.	a) 324 (67.9)<br>b) 63 (13.2)<br>c) 2 (0,4)	Total answers - 477. Instead of answers a), b) and c), women also gave numerical answers: 29 weeks – 1 (0.2%), 33 – 1 (0.2), 34 – 5 (1.0), 35 – 1 (0.2), 36 – 12 (2.5), 37 – 10 (2.1), 38 – 18 (3.8), 39 – 18 (3.8), 40 – 17 (3.6), 41 – 5 (1.0).
**4. What were the baby's measurements at birth?**	a) 477 g (99,8)	For answer a): mean – 3248 g, median – 3300 g, range

Question	Number of answers (%)	Comments
a) Birth weight ………<br>b) Head circumference ………<br>c) Body length ………	b) 409 g (85,6)<br>c) 475 g (99,4)	970–5050 g, standard deviation (SD) – 638 g.<br>For answer b): mean – 34 cm, median – 34 cm, range 26-52 cm, SD – 2.1 cm.<br>For answer c): mean – 53 cm, median – 54 cm, range 27-64 cm, SD – 4.0 cm.
**5. What was the Apgar score in the first minute of life?**<br>a) 8 - 10<br>b) 4 - 7<br>c) 0 - 3	a) 451 (94.7)<br>b) 23 (4.8)<br>c) 2 (0.4)	Total answers – 476.
**6. Have you had any chronic diseases during your current pregnancy, such as:**<br>a) pre-pregnancy hypertension<br>b) hypertension developing during pregnancy<br>c) pre-pregnancy diabetes<br>d) gestational diabetes (developing during pregnancy)<br>e) chronic kidney disease<br>f) hypothyroidism<br>g) bronchial asthma<br>h) allergy<br>i) other chronic lung diseases<br>j) other chronic diseases of the cardiovascular system<br>k) anemia<br>l) cancer<br>m) chronic infectious diseases: hepatitis B, hepatitis C, AIDS,<br>n) depression<br>o) other	Yes:<br>a) 10 (2.1)<br>b) 39 (8.2)<br>c) 11 (2.3)<br>d) 76 (15.9)<br>e) 4 (0.8)<br>f) 199 (41.7)<br>g) 7 (1.5)<br>h) 17 (3.6)<br>i) 2 (0.4)<br>j) 9 (1.9)<br>k) 44 (9.2)<br>l) 1 (0.2)<br>m) 2 (0.4)<br>n) 13 (2.7)<br>o) 30 (6.3)	Total answers – 477.
**7. Have you had a miscarriage/miscarriages?**<br>a) yes<br>b) no	a) 100 (21.1)<br>b) 375 (78.9)	Total answers – 475.
**8. Have you used any**		Total answers – 472.

Question	Number of answers (%)	Comments
**medicine during this pregnancy? If so, please list which ones**<br>a) no<br>b) yes, as follows:<br>- progesterone<br>- hypertension medicines	a) 185 (39.0)<br>b) 289 (61.0)<br>c) 47 (10.0)<br>d) 32 (6.8)	
**9. Have you colored your hair during this pregnancy?**<br>a) yes<br>b) no	a) 237 (54.9)<br>b) 195 (45.1)	Total answers – 432.
**10. Have you used sweeteners during this pregnancy?**<br>a) yes,<br>b) no.	a) 43 (10.0)<br>b) 387 (90.0)	Total answers – 430.
**11. Are you on a diet or have you been on a diet? You can select more than one answer.**<br>a) lactose-free<br>b) gluten-free<br>c) vegetarian/vegan<br>d) another	Yes:<br>a) 26 (5.5)<br>b) 13 (2.8)<br>c) 17 (3.6)<br>d) 53 (11.3)	Total answers: a) – 470, b) – 471, c) – 471, d) – 469.
**4. Have you used/taken the following medications during your current pregnancy? (you can mark several answers, you can choose not to mark any**	Yes:<br>a) 10 (2.1)<br>b) 0 (0)<br>c) 5 (1.1)<br>d) 2 (0.5)<br>e) 0 (0)	Total answers: a) – 472, b) – 470, c) – 435, d) – 433, e) – 434b, f) – 434.

Question	Number of answers (%)	Comments
**answer).**<br><br>a) sedative medicines prescribed by a doctor<br>b) sedatives available on prescription without the doctor's knowledge<br>c) over-the-counter sedatives<br>d) inhalant drugs/spliff<br>e) drugs by injection<br>f) the so-called "legal highs"	f) 0 (0)	
**5. Doctor who took care of you during your pregnancy:**<br>a) Have not talked with you about drinking alcohol during pregnancy.<br>b) Encouraged to restrain completely from drinking alcohol during pregnancy.<br>c) Allowed to drink small amounts of alcohol during pregnancy.<br>d) Encouraged to drink small amounts of alcohol during pregnancy.	a) 194 (45.2)<br>b) 233 (54.3)<br>c) 1 (0.2)<br>d) 1 (0.2)	Total answers – 429.
**6. What is, in your opinion, daily intake of alcohol which is safe in pregnancy?**<br>a) There is no safe amount of alcohol during pregnancy.<br>b) I do not know.<br>c) One glass of red wine.<br>d) One pint of beer.<br>e) One glass of cognac.	a) 410 (94.9)<br>b) 16 (3.7)<br>c) 6 (1.4)<br>d) 0 (0)<br>e) 0 (0)	Total answers – 432.
**7. Were there/are there any strong sources of electromagnetic radiation, such as: mobile phone transmitting antennas or high voltage transmission**	a) 52 (12.1)	Total answers – 431.

Question	Number of answers (%)	Comments
**lines in your work environment or in your house surroundings?**<br>f) Yes<br>g) No<br>h) I do not know	b) 281 (65.2)<br>c) 98 (22.7)	
**8. Have you had an X-RAY examination during this pregnancy?**<br>i) yes<br>j) no	a) 12 (2.8)<br>b) 418 (97/2)	Total answers – 430.
**9. Have you drunk caffeinated drinks during this pregnancy? For example: coffee, strong black or green tea, energy drinks, Coca-cola.**<br>a) yes<br>b) no	a) 364 (84.1)<br>b) 69 (15.9)	Total answers – 433.
**18. Have you smoked cigarettes during this pregnancy?**<br>a) I have never smoked (continue to question 20.).<br>b) I have stopped before pregnancy or earlier.<br>c) I have stopped during this pregnancy.<br>d) I still smoke.	a) 345 (81.9)<br>b) 76 (18.1)<br>c) 46 (10.9)<br>d) 8 (1.9)	Total answers – 421.
**19. How many cigarettes have you smoked daily:**<br>k) In the last 3 months before pregnancy? ………	0 – 282 (80.1)<br>1 – 7 (2.0)<br>2 – 7 (2.0)<br>3 – 5 (1.4)<br>4 – 2 (0.6)<br>5 – 12 (3.4)<br>6 – 1 (0.3)<br>7 – 2 (0.6)<br>8 – 2 (0.6)<br>9 – 1 (0.3)<br>10 – 14 (4.0)<br>12 – 1 (0.3)<br>14 – 1 (0.3)	Total answers – 352.

Question	Number of answers (%)	Comments
	15 – 12 (3.4)<br>20 – 3 (0.9)	
**19. How many cigarettes have you smoked daily:**<br><br>l) In the first 3 months of pregnancy? ………	0 – 327 (94.5)<br>1 – 3 (0.9)<br>2 – 5 (1.4)<br>3 – 3 (0.9)<br>4 – 4 (1.2)<br>20 – 1 (0.3)	Total answers – 346.
**19. How many cigarettes have you smoked daily:**<br><br>m) In the last 3 months of pregnancy? ………	0 – 282 (98.8)<br>3 – 1 (0.3)<br>5 – 1 (0.3)<br>15 – 1 (0.3)<br>20 – 1 (0.3)	Total answers – 344.
**19. How many cigarettes have you smoked daily:**<br><br>n) I still smoke around ……… cigarettes daily.	0 – 344 (99.1)<br>10 – 2 (0.6)<br>20 – 3 (0.3)	Total answers – 347.
**20. How often have you consumed beverages containing alcohol before pregnancy?**<br><br>a) Never<br>b) 1 per month or less<br>c) 2 to 4 times per month<br>d) 2 or 3 times per week<br>e) 4 times per week or more	a) 56 (12,6)<br>b) 198 (44,5)<br>c) 162 (36,4)<br>d) 26 (5,8)<br>e) 3 (0,7)	Total answers – 445.
**21. How often did you consume beverages containing alcohol during pregnancy?**<br><br>a) Never (continue to question 25.)<br>b) 1 per month or less<br>c) 2 to 4 times per month	a) 454 (95,8)<br>b) 15 (3,2)<br>c) 5 (1,1)<br>d) 0 (0)<br>e) 0 (0)	Total answers – 474.

Question	Number of answers (%)	Comments
d) 2 or 3 times per week<br>e) 4 times per week or more		
**22. What kind of alcohol beverages did you consume during pregnancy? You can choose more than 1 answer.**<br>a) wine<br>b) beer<br>c) high-proof alcohol	a) 14 (3.0)<br>b) 8 (1.7)<br>c) 1 (0.2)	Total answers – 474. Due to the fact that more than one answer could be given to this question, the actual number of people who answered is lower (n=19):<br>- woman no. 54 declared that she consumed all three alcoholic beverages (answers a, b, c)<br>- women no. 186 and 237 declared that they consumed beer and wine (answers a, b)
**23. In which trimester did you consume alcohol beverages during pregnancy? You can choose more than 1 answer.**<br>a) 1st trimester (1st - 3rd month)<br>b) 2nd trimester (4th - 6th month)<br>c) 3rd trimester (7th - 9th month)	a) 8 (1,7)<br>b) 7 (1.5)<br>c) 10 (2.1)	Total answers – 474. Due to the fact that more than one answer could be given to this question, the actual number of respondents is lower (n=19):<br>- women no. 42 and 363 declared that they consumed alcoholic beverages in all trimesters (answers a, b, c)<br>- women numbered 4 and 244 declared that they consumed alcohol in the 2nd and 3rd trimesters (answers b, c)
**24. What was the greatest amount of alcohol you drank at once during pregnancy?**<br>a) 1 small beer, or 1 glass of wine, or 1 glass of vodka, or 1 cocktail<br>b) 2 small beers, or 2 glasses of wine, or 2 glasses of vodka, or 2 cocktails<br>c) 3 small beers, or 3 glasses of wine, or 3 glasses of vodka, or 3	a) 14 (3.0)<br>b) 0 (0)<br>c) 2 (0.4)	Total answers – 474.

Question	Number of answers (%)	Comments
cocktails		
**25. Can you rate your knowledge on positive and negative factors affecting the course of pregnancy?**<br>a) High<br>b) Moderate<br>c) Low<br>d) No knowledge	a) 146 (34.2)<br>b) 275 (64.4)<br>c) 5 (1.2)<br>d) 1 (0.2)<br>e) 0 (0)	Total answers – 427.
**26. Where did you look for information on substances toxic to the fetus? You can choose more than 1 answer.**<br>a) internet<br>b) media (press, radio, TV)<br>c) doctor<br>d) midwife<br>e) birthing classes<br>f) family<br>g) friends<br>h) other.	a) 391 (90.5)<br>b) 138 (31.9)<br>c) 279 (64.6)<br>d) 155 (35.9)<br>e) 160 (37.0)<br>f) 182 (42.1)<br>g) 147 (34.0)<br>h) 59 (13.7)	Total answers – 432.
**MATERNAL CHARACTERISTICS**		
**1. How old are you?**<br>a) less than 18 years old<br>b) 18-24 years old<br>c) 25-30 years old<br>d) 31-35 years old<br>e) more than 35 years old	a) 2 (0.4)<br>b) 39 (8.2)<br>c) 188 (39.5)<br>d) 151 (31.7)<br>e) 96 (20.2)	Total answers – 476.
**2. What is your education?**<br>a) Primary school<br>b) Middle school<br>c) Basic vocational education	a) 3 (0.7)<br>b) 9 (2.0)<br>c) 29 (6.3)<br>d) 46 (10.0)<br>e) 49 (10.6)	Total answers – 461.

Question	Number of answers (%)	Comments
d) Secondary vocational education<br>e) High school diploma/general secondary schools<br>f) Bachelor degree/Engineer<br>g) Master's degree	f) 51 (11.1)<br>g) 274 (59.4)	
**3. What is your marital status?**<br>a) Single<br>b) Married<br>c) Informal relationship<br>d) Separated<br>e) Divorced<br>f) Widow	a) 61 (12.8)<br>b) 346 (72.8)<br>c) 61 (12.8)<br>d) 3 (0.6)<br>e) 4 (0.8)<br>f) 0 (0)	Total answers – 475.
**4. Where do you live?**<br>a) Countryside<br>b) City < 100.000 inhabitants<br>c) City >100.000 and <100.000 inhabitants<br>d) City > 400.000 inhabitants	a) 103 (21.7)<br>b) 69 (14.5)<br>c) 73 (15.4)<br>d) 230 (48.4)	Total answers – 475.
**5. Do you work? What is the type of your profession?**<br>a) Office/administrative work.<br>b) Physical work.<br>c) I am a business owner.<br>d) I do not work.<br>e) I am a student.	a) 272 (58.4)<br>b) 60 (12.9)<br>c) 47 (10.1)<br>d) 68 (14.6)<br>e) 19 (4.1)	Total answers – 466.
**6. Can you rate your social and living conditions?**<br>a) Very good	a) 244 (56.5)<br>b) 169 (39.1)<br>c) 18 (4.2)	Total answers – 432.

Question	Number of answers (%)	Comments
b) Good<br>c) Middle<br>d) Low	d) 1 (0.2)	
**7. Who lives with you at one household? You can choose more than 1 answer.**<br>a) Husband<br>b) Partner<br>c) Child/children<br>d) Parents<br>e) Siblings<br>f) Other people, who are they? ………	a) 343 (73.1)<br>b) 120 (25.6)<br>c) 245 (52.2)<br>d) 41 (8.7)<br>e) 11 (2.3)<br>f) 9 (1.9)	Total answers – 469.
